# Solvent and catalyst-free bromofunctionalization of olefins using a mechanochemical approach†

**DOI:** 10.1039/d1ra01816g

**Published:** 2021-04-12

**Authors:** Jonathan Wong, Ying-Yeung Yeung

**Affiliations:** Department of Chemistry, State Key Laboratory of Synthetic Chemistry, The Chinese University of Hong Kong Shatin, NT Hong Kong China yyyeung@cuhk.edu.hk

## Abstract

Bromofunctionalizations of olefins are an important class of chemical transformations. *N*-Bromoimide reagents are commonly used in these reactions but catalysts and chlorinated solvents are often employed to achieve a reasonable reaction rate. In this report, we present a solvent and catalyst-free bromofunctionalization of olefins using mechanical force.

## Abstract 2

Efficient bromofunctionalization of olefins including bromolactonization, bromocycloetherification, and intermolecular bromoesterification were achieved under solvent and catalyst-free conditions using a mechanochemical approach.

## Graphical abstract

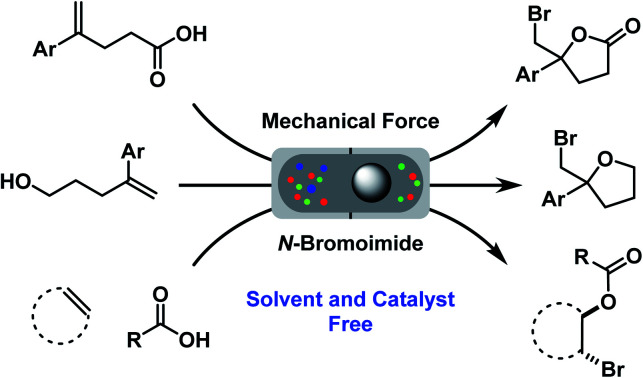

Bromofunctionalization of olefinic substrates represents one of the most useful classes of halogenation reactions. It allows for the simultaneous addition of a bromine atom and another functional group across the alkene C–C double bond.^1^ Further modification of the bromine handle can be easily done using conventional methods. These reactions are fundamentally important in industrial chemical synthetic processes where environmental sustainability should be taken into serious consideration.^2^ Although molecular bromine is an inexpensive halogen source, using it in halogenation processes has proven problematic due to its corrosive and toxic nature.^3^ Oxidative bromination, which involves the *in situ* generation of bromine *via* the oxidation of a bromide anion, provides a greener alternative and can avoid the use of stoichiometric amounts of molecular bromine. However, these methods are not suitable for the co-halogenation of olefins (*e.g.* bromocyclization) because dibromination readily occurs as the major side reaction.^4^ Thus, milder and more user-friendly halogen sources such as *N*-bromosuccinimide (NBS) and 1,3-dibromo-5,5-dimethylhydantoin (DBDMH) are frequently employed in many bromofunctionalization reactions.^5^ Well reported examples include bromocyclization reactions such as bromolactonization^6–15^ and bromoetherification^16–19^ as well as the more challenging intermolecular bromoesterification.^20–26^ Because of the high polarity of the *N*-bromoimide reagents, polar solvents such as *N*,*N*-dimethylformamide and acetonitrile are often required for good solvation which poses difficulties in the purification process. The use of relatively less polar solvents such as dichloromethane and chloroform are also commonly reported. However, various catalysts (*e.g.* Lewis bases to activate the electrophilic Br) or additives (*e.g.* Brønsted bases to deprotonate the pronucleophiles such as carboxylic acids) are often required to achieve high reactivity. Furthermore, the use of chlorinated solvents in industrial settings is strongly regulated due to its ability to cause ozone depletion as well as its biological carcinogenicity.^27,28^ Our previous work in this area include the use of lipophilic indole catalysts as a solid-to-liquid phase bromine shuttle for efficient bromination with *N*-bromoimide reagents in environmentally benign lipophilic solvent.^29–31^ Despite these precedent efforts on establishing greener bromofunctionalization of olefins, recycling solvents at a large scale is still highly energy-consuming and is undesirable at industrial sectors. In view of the ever-increasing demand for green chemical protocols, the development of more sustainable bromofunctionalization processes is still highly desired.

Mechanochemistry has re-emerged as a tool in various chemical transformations.^32^ This strategy uses mechanical energy to induce various reactions. In many occasions, solvents are not required and the mechanochemically activated reactions can often result in higher efficiency and selectivity. Herein, we report a comprehensive study on the halo-*O*-cyclization of olefinic substrates using mechanical force under solvent and catalyst-free conditions. The protocol has also been applied to the three-component intermolecular bromoesterification of olefins. Using a Retsch mixer mill we have successfully achieved the solvent, catalyst and additive-free bromolactonization, bromoetherification and intermolecular bromoesterification reactions with *N*-bromoimide reagents (Scheme 1, eqn (1)–(3)). These reactions proceeded efficiently with near equimolar quantities of all reagents at ambient conditions. The products can be purified by column-free filtration and the bromine carrier byproducts (*e.g.* succinimide of NBS) can be recovered effectively for recycling.

**Scheme 1:**
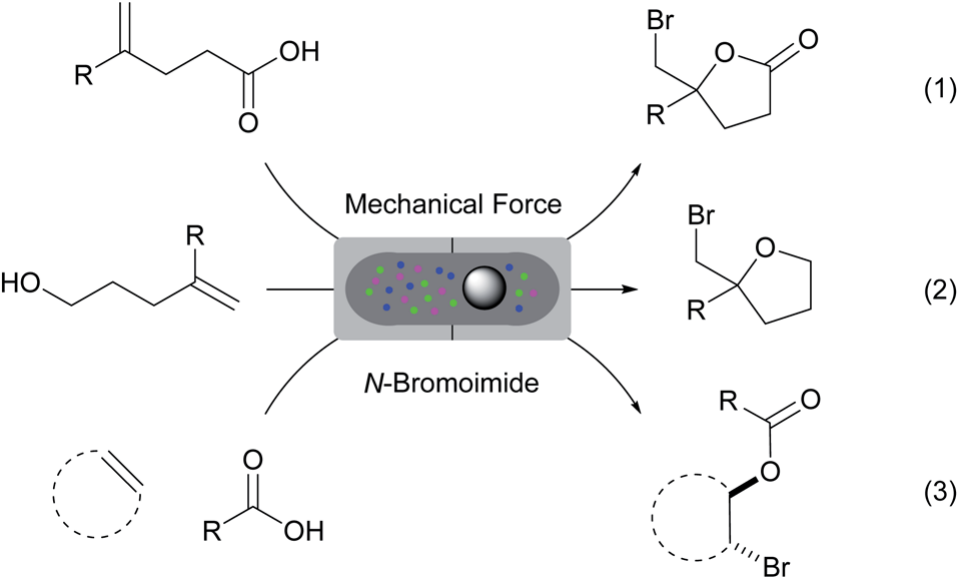
Solvent, catalyst and additive-free bromofunctionalizations of olefinic substrates by mechanical force.

We began our investigation using the bromolactonization of various 1,1-disubstituted alkenoic acids to produce γ-lactones. Though this type of reaction is well-documented, reported examples often rely on the use of high boiling point polar or chlorinated solvents in combination with various catalyst systems.^1^ A recent example was reported by Tungen and co-workers, where the organoselenium catalyst, named DECAD, was found to catalyze the efficient bromolactonization of various olefinic acids (Scheme 2, eqn (1)).^6^ However, this protocol requires the use of acetonitrile as solvent, which is not trivial to remove due to its relatively high boiling point and water miscibility. Furthermore, the reaction system is water sensitive and molecular sieves are needed. Another recent report by Kumar and co-workers showcased the use of a *C*_2_-symmetric sulfide catalyst carrying two dihydroquinine chiral scaffolds in various asymmetric bromolactonization reactions (Scheme 2, eqn (2)).^33^ In this example, a solvent blend with chloroform was required to dissolve the various reaction components in order to maintain efficient reactivity.

**Scheme 2:**
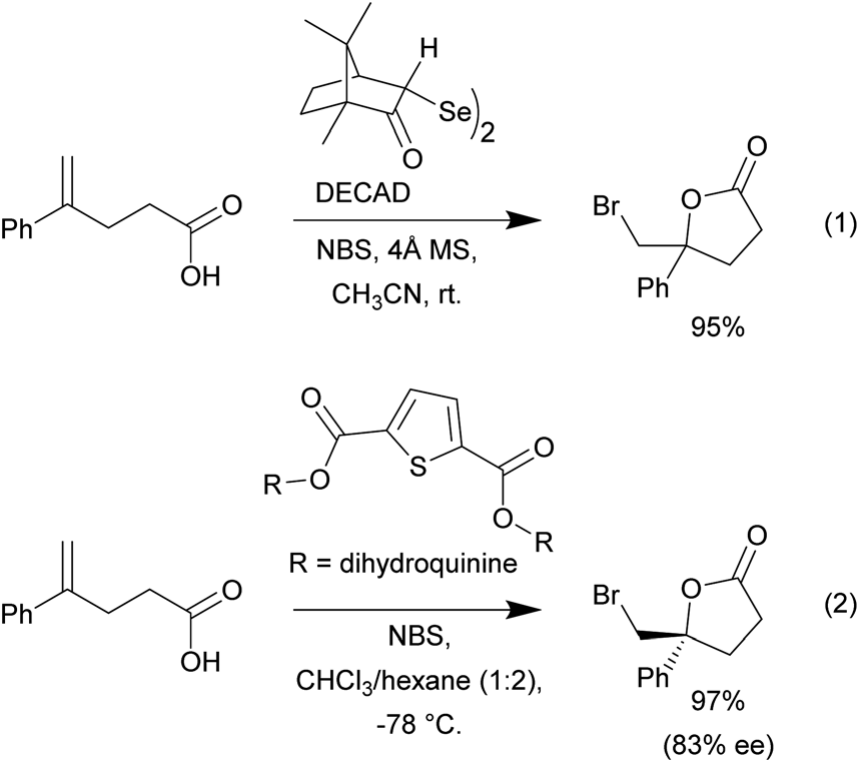
Selected recent literature examples of bromocyclization reactions.

Using a mixer mill at an oscillation frequency of 20 Hz with solid samples of alkenoic acid 1a and NBS, 14% of lactone 2a was produced in 10 minutes (Table 1, entry 1). When the reaction was conducted in dichloromethane, only 10% of 2a was detected even after 2 hours (entry 2). No reaction was observed when the solids of 1a and NBS were mixed using a magnetic stirrer bar (entry 3). These results highlight the crucial effect of mechanical force on the bromolactonization. At an oscillation frequency of 30 Hz and with a longer reaction time, gradual improvement in reaction conversion was observed leading to complete reaction after 2 hours (entries 4–6). Bromolactonization using the more reactive halogen source DBDMH gave near quantitative yields of 2a in a shorter time period (entry 7). NBS is known to readily decompose through light-activated radical pathways, causing faster reaction rates in the solution phase (as indicated by a rapid change from a colorless to a red/brown solution). Since the reaction was carried out in a stainless-steel milling chamber, the enclosed system is completely shielded from light; thereby minimizing the undesirable NBS radical decomposition.

**Table 1:** Conditions optimization for the bromolactonization

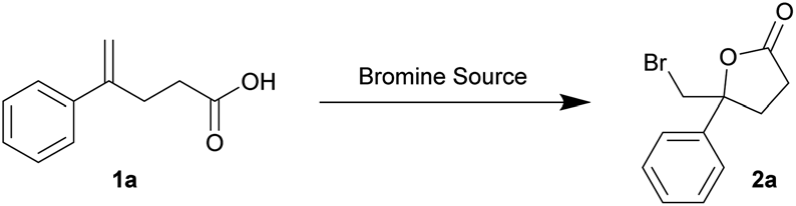				
Entry^a^	Bromine source	Frequency	Time	Yield (%)^b^
1	NBS	20 Hz	10 min	14
2^c^	NBS	—	120 min	10
3^d^	NBS	—	120 min	0
4	NBS	30 Hz	30 min	20
5	NBS	30 Hz	60 min	33
6	NBS	30 Hz	120 min	99 (97)^e^
7	DBDMH	20 Hz	10 min	99

a Reactions were conducted using a Retsch mixer mill (MM 400) in a 10 mL zirconium oxide chambers with alkenoic acid 1a (0.2 mmol) and bromine source (0.22 mmol) at ambient temperature in the absence of light.

b Determined by NMR spectroscopy with dibromomethane as the internal standard.

c Reaction conducted in dichloromethane (0.2 M).

d Reaction was conducted under neat conditions and the solid samples were mixed by a magnetic stirrer bar.

e Isolated yield.

Alkenoic acids carrying various substituents were examined in order to evaluate the influence of electronic effects on the reaction efficiency. The relatively electron rich 4-methyl and 4-methoxy phenyl alkenoic acids (1b and 1c) afforded lactones 2b and 2c in 98 and 87% yields, respectively. Relatively electron-deficient olefins are typically less reactive towards electrophilic halogenations; due to a lower availability of the π-electrons for formation of a haliranium intermediate. Nonetheless, 4-chloro, 4-fluoro and 4-trifluoromethyl phenyl olefinic acids readily cyclized to give lactones 2d, 2e, and 2f in 98, 90 and 98% yields, respectively, in one hour. The acylated alkenoic acid 1g was also well tolerated under the mild conditions, yielding lactone 2g in 81% yield. This method was also compatible with the 1,2-disubstituted *trans*-olefinic acid 3, providing 4 in good yield and diastereoselectivity (Table 2).

**Table 2:** Substrate scope of bromolactonization^a^

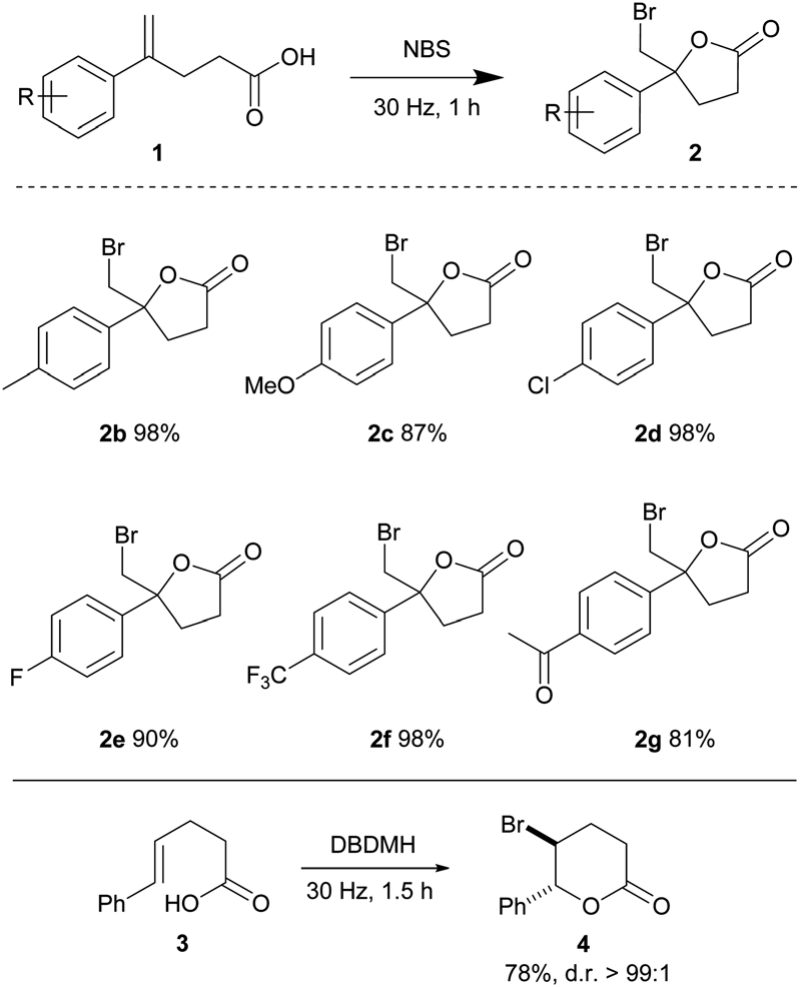

a Reactions were conducted using a Retsch mixer mill (MM 400) in a 10 mL zirconium oxide chamber with alkenoic acid 1 (0.2 mmol) and NBS (0.22 mmol) at ambient temperature. The yields are isolated yields.

In view of the high efficiency, low operational complexity and environmental sustainability of this mechanical force-driven bromocyclization; we then sought to test its compatibility with less reactive substrates. The bromocycloetherifications of olefinic alcohols 5 were chosen because they are known to be less efficient when compared to the analogous bromolactonization reactions.^17,18,34^ Using a mixer mill set at 15 Hz and NBS as the brominating reagent, substrate 5a yielded 58% of the bromoether 6a in only 10 minutes (Table 3, entry 1). The reaction efficiency was far superior to that of the solvated reaction in dichloromethane (entry 2). Increasing the oscillation frequency gave higher yields within the same time period (entry 3) and full conversion was realized after 1 hour (entries 4–5).

**Table 3:** Conditions optimization for the bromocycloetherification

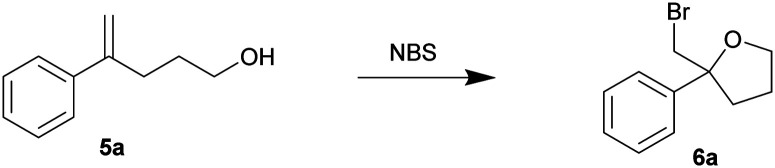			
Entry^a^	Frequency	Time	Yield (%)^b^
1	15 Hz	10 min	58
2^c^	—	10 min	2
3	30 Hz	10 min	69
4	30 Hz	30 min	85
5	30 Hz	60 min	99 (98)^d^

a Reactions were conducted using a Retsch mixer mill (MM 400) in a 10 mL zirconium oxide chambers with olefinic alcohol 5a (0.2 mmol) and NBS (0.22 mmol) at ambient temperature in the absence of light.

b Yields were acquired by NMR spectroscopy with dibromomethane as the internal standard.

c Reaction was conducted in CH_2_Cl_2_ (0.2 M).

d Isolated yield.

Using our optimized conditions, we continued to evaluate the reaction using olefinic alcohols of varied electronic properties (Table 4). The relatively electron-rich *p*-tolyl olefinic alcohol 5b gave near quantitative yield of the product tetrahydrofuran 6b. Good yields were also obtained when the relatively electron-rich and electron-deficient olefinic alcohols were subjected to the same conditions. 4-Methoxy (5c), 4-chloro (5d), 4-fluoro (5e), 4-trifluoromethyl (5f) and 3,5-bistrifluoromethyl (5g) phenyl substituted olefinic alcohols gave products 6c–6g in 76–98% yield.

**Table 4:** Substrate scope of bromocycloetherification^a^

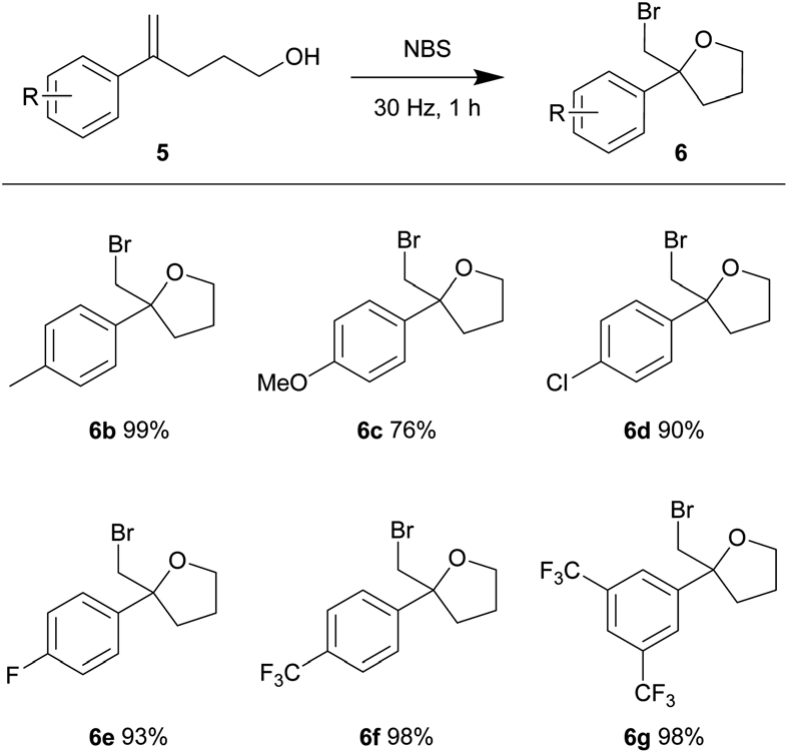

a Reactions were conducted using a Retsch mixer mill (MM 400) in a 10 mL zirconium oxide chamber with olefinic alcohol 5 (0.2 mmol) and NBS (0.22 mmol) at ambient temperature. The yields are isolated yields.

To further explore the scope of this mechanochemical bromination protocol, the intermolecular bromoesterifications of alkenes and carboxylic acids was chosen as our next target. These reactions are intrinsically sluggish when compared to their intramolecular counterparts due to a higher reaction entropy.^34^ This low reactivity is often overcome using either a super-stoichiometric amount of the acid partner or directly as the solvent; resulting in poor atom-economy. For example, in two separate publications by Braddock *et al.* the tetramethylguanidine (TMG) and iso-amarine were used to catalyze the bromoacetoxylation of styrene (Scheme 3a).^35,36^ In these examples, chlorinated solvent and a large excess of acetic acid was required to effectively promote the reaction. More recently Pimenta *et al.* has reported the use of DABCO as a catalyst in the bromoacetoxylation of various alkenes (Scheme 3b).^22^ In this example two equivalents of acetic acid were required when the reaction was conducted in dichloromethane. The authors then chose to use an excess of acetic acid as a replacement for the environmentally hazardous dichloromethane solvent. In view of the undesired conditions required for this type of reaction, we sought to optimize our mechanochemical bromofunctionalization protocol to achieve a more efficient and environmentally sustainable alternative.

**Scheme 3:**
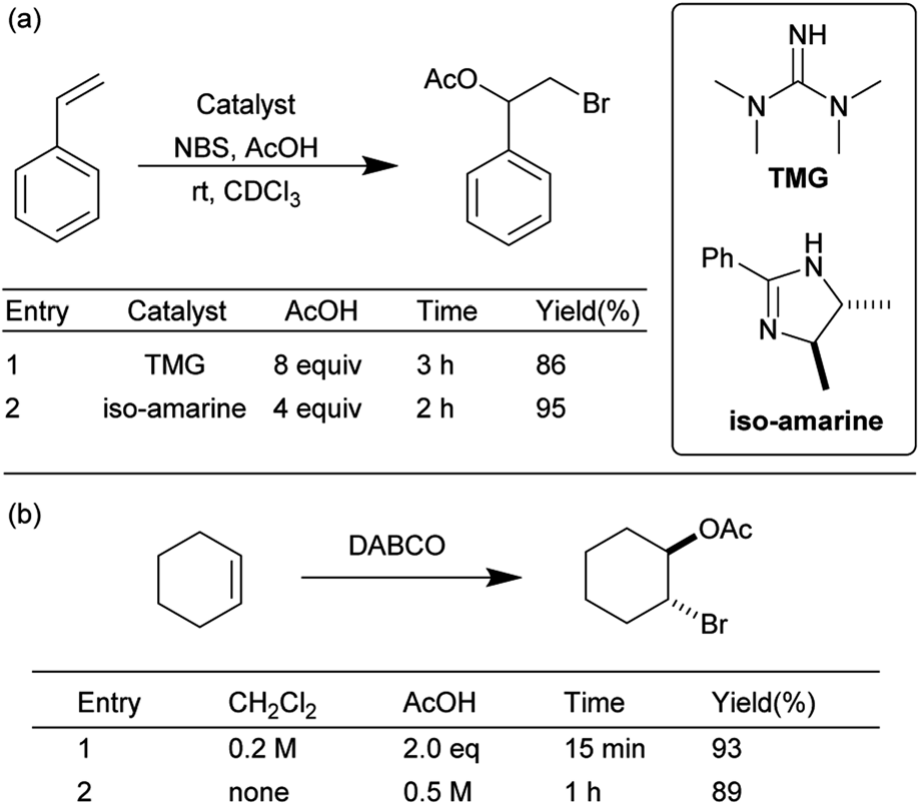
Selected literature examples of intermolecular bromoesterification.

In our initial substrate optimization, benzoic acid 7a and styrene 8a were used as the reacting partners with a near equimolar ratio. When NBS was used as the bromine source, the reaction did not proceed (Table 5, entries 1 and 2). Using DBDMH as the bromine source, full conversion was reached after 10 minutes at an oscillation frequency of 30 Hz (entries 3 and 4). In contrast, very poor yields were obtained when the reaction was conducted neat or under full solvation in dichloromethane (entries 5 and 6, respectively). We also compared the source of mechanical force. It was found that the reaction using a mixer mill is more efficient than that of a planetary mill (entry 7), although the planetary mill is more suitable for scaled up processes.

**Table 5:** Conditions optimization for the intermolecular bromoesterification

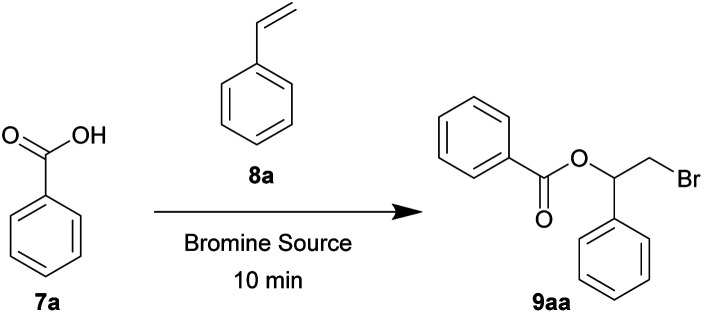			
Entry^a^	Bromine source	Frequency	Yield (%)^b^
1	NBS	20 Hz	0
2	NBS	30 Hz	0
3	DBDMH	20 Hz	74
4	DBDMH	30 Hz	94 (92)^c^
5^d^	DBDMH	—	6
6^e^	DBDMH	—	4
7^f^	DBDMH	30 Hz	78

a Reactions were conducted using a Retsch mixer mill (MM 400) in a 10 mL zirconium oxide chambers with benzoic acid 7a (0.2 mmol), styrene 8a (0.22 mmol) and bromine source (0.22 mmol) at ambient temperature.

b Determined by NMR spectroscopy with dibromomethane as the internal standard.

c Isolated yield.

d Reaction was conducted in dichloromethane (0.2 M).

e Reaction was conducted under neat condition and the samples were mixed by a magnetic stirrer bar.

f A planetary mill was used.

With the optimized conditions in hand, we investigated the substrate scope using different combinations of alkene and carboxylic acid derivatives. Here, we observed that some substrates required a longer time to achieve full conversion and a reaction time of one hour was used in general. First, we tested various alkene partners while using benzoic acid 7a as the nucleophile (Table 6). Styrene derivatives carrying electron-donating aryl substituents gave excellent yields of the resulting bromoesters. 4-Methyl (8b), 2,4,6-trimethyl (8c) and 4-*tert-*butyl (8d) styrene afforded the corresponding bromoesters (9ab), (9ac) and (9ad) in 93%, 99%, and 90% yields, respectively. The steric bulk of styrene 8c and 8d were well-tolerated. The relatively electron-deficient 4-bromo (8e), 4-chloro (8f) and 4-fluoro (8g) styrene derivatives also reacted efficiently to give bromoesters 9ae, 9af, and 9ag in excellent yields, respectively. The cyclic aliphatic olefin, cyclohexene 8h, reacted smoothly to give bromoester 9ah in 95% yield.

**Table 6:** Substrate scope of intermolecular bromoesterification^a^

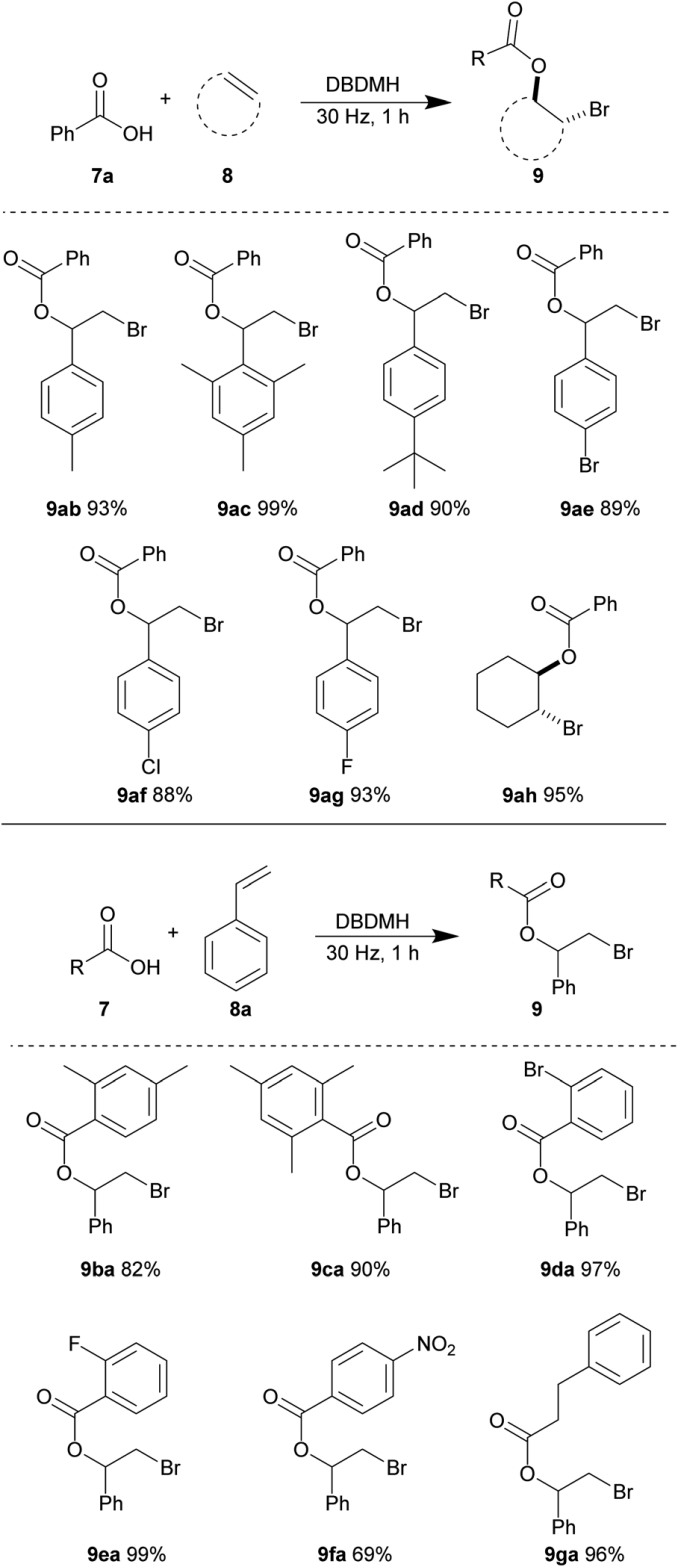

a Reactions were conducted using a Retsch mixer mill (MM 400) in 10 mL zirconium oxide chambers with carboxylic acid 7 (0.2 mmol), alkene 8 (0.22 mmol) and DBDMH (0.22 mmol) at ambient temperature. Yields shown are isolated yields.

Next, various benzoic acid derivatives 7 were tested using styrene 8a as the reaction partner. The sterically bulky and relatively electron-rich benzoic acid derivatives 2,4-dimethyl (7b) and 2,4,6-trimethyl benzoic acid (7c) reacted efficiently with styrene 8a to produce bromoesters 9ba and 9ca in 82% and 90% yields, respectively. The relatively electron-deficient benzoic acid derivatives carrying 2-bromo (7d) and 2-fluoro (7e) substituents also reacted smoothly, giving nearly quantitative yields of bromoesters 9da and 9ea. A moderate decrease in yield of ester 9fa was observed when 4-nitrobenzoic acid 7f was subjected to the same reaction conditions. We believe that the strong electron-withdrawing ability of the nitro substitution may reduce the nucleophilicity for the carboxylate group. The aliphatic acid derivative, 3-phenylpropionic acid 7g was also well-tolerated, yielding bromoester 9ga in 96% yield.

To demonstrate the column-free product isolation and bromine carrier recyclability of this method, a scaled-up filtration/recovery experiment was conducted. When the bromolactonization of 1a was conducted in a reaction chamber of the same size but at 1 mmol scale. At an oscillation frequency of 30 Hz, the reaction reached completion after 1 hour (Scheme 4A). Pure hexane was used to rinse the contents of the mill chamber into a sintered funnel, where the solid remains of the bromine carrier was removed from the product. This gave a 95% isolated yield of lactone 2a in high purity (see ESI, Section G†). The residue was quenched with saturated sodium thiosulfate solution and extracted with ethyl acetate. This returned a 98% recovery of the 5,5-dimethylhydantoin which can be recycled for the synthesis of DBDMH. Next, the bromocycloetherification of 5a was scaled up to 1 mmol scale. At an oscillation frequency of 30 Hz, the reaction reached completion after 3 hours (Scheme 4B). The sample was purified by filtration using an analogous approach as in the bromolactonization example. This protocol gave product 6a in 93% and succinimide in 99% (see ESI, Section G†). A column-free filtration purification of the bromoester product and recovery of 5,5-dimethylhydantoin was also demonstrated. Using cyclohexene 8h and benzoic acid 7a as the reacting partners with DBDMH as the bromine source, complete reaction was achieved after 3 hours under an oscillation frequency of 30 Hz (Scheme 4C). Extraction of the milling chamber with hexane and filtration over sintered glass, gave the bromoester 9ah in 99% yield together with 95% recovery of 5,5-dimethylhydantoin with high purity (see ESI, Section G†).

**Scheme 4:**
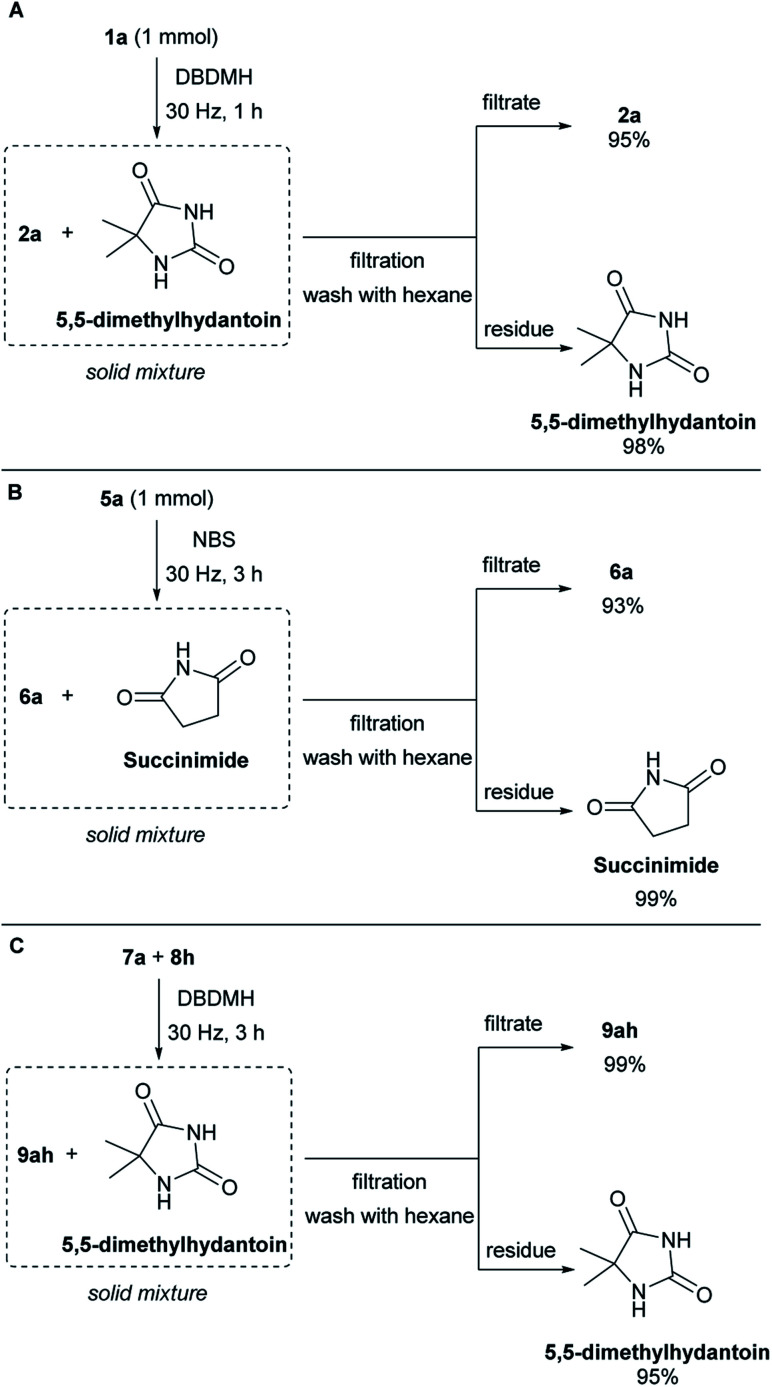
Efficient product isolation and imide recovery.

## Conclusions

In summary, with a mechanochemical approach we have successfully achieved high efficiency in various bromofunctionalization reactions of olefins using *N*-haloimides as the bromine source. This method allows for the reactions to proceed under solvent, catalyst and additive-free conditions. Avoiding the need for environmentally hazardous chlorinated solvents commonly employed to provide good solvation of the *N*-haloimides. Reaction efficiency was also maintained in the absence of any catalyst or additives, allowing the reaction to proceed in a mild manner within a short reaction period. The substrate scope for bromolactonization, bromoetherification and bromoesterification have shown great compatibility with varied steric and electronic substrate properties. With near equimolar amounts of substrates and excellent recyclability of the bromine source, this method offers superior atom economy when compared to existing protocols. Furthermore, hassle-free filtration purification circumvents the need for flash-column chromatography. Together with the ease of operation we believe that mechanochemical methods can provide both time and energy saving alternatives to the traditionally solvated reaction protocols of bromofunctionalizations of olefins.

## Conflicts of interest

There are no conflicts to declare.

## Supplementary Material

RA-011-D1RA01816G-s001
