# Supplementary material for: Solvent and catalyst-free bromofunctionalization of olefins using a mechanochemical approach
Source: RSC Adv. 2021 Apr 12;11(22):13564–70. doi: 10.1039/d1ra01816g (PMC8697521; doi:10.1039/d1ra01816g)
Supplement: RA-011-D1RA01816G-s001 [file RA-011-D1RA01816G-s001.pdf]

Electronic Supplementary Material (ESI) for RSC Advances.
This journal is © The Royal Society of Chemistry 2021

Electronic Supplementary Information

Solvent and Catalyst-Free Bromofunctionalization of Olefins Using a Mechanochemical Approach

Jonathan Wong, Ying-Yeung Yeung*

Department of Chemistry and State Key Laboratory of Synthetic Chemistry, The Chinese University of Hong Kong, Shatin, NT, Hong Kong, China

E-mail: yyyeung@cuhk.edu.hk; Tel: +852-3943-6377

**Page**	**Content**
**S2**	**General Information**
**S3-S27**	**Experimental procedures and physical data**
**S28-S121**	**^{1}H and ^{13}C NMR spectra**

(A) General Information

Unless otherwise specified, commercially available reagents were used directly without further purification. All reactions requiring anhydrous conditions were conducted by standard procedures under nitrogen atmosphere. The solvents were dried over a solvent purification system from Innovative Technology. Melting points were determined on a Buchi B-540b melting point apparatus. ^{1}H NMR and $^{13}C\{^{1}H\}$ NMR spectra were recorded on a Bruker AMX500 (500 MHz) spectrometer or a Bruker AMX400 (400 MHz) spectrometer. Proton and carbon chemical shifts are reported in parts per million (ppm) values downfield from TMS (δ 0.00) and referenced to residual protons in NMR solvents ($CDCl_3$ at δ 7.26, CD_2Cl_2 at δ 5.36) or carbon signals in NMR solvent ($CDCl_3$ at δ 77.16, CD_2Cl_2 at δ 55.42). ^{1}H NMR data were reported as follows: chemical shift, multiplicity, coupling constants (Hz), and integration. High resolution mass spectra were obtained on a Thermo Finnigan MAT95XL Magnetic Sector mass spectrometer (ionization mode: EI) or a Thermo Q Exactive Hybrid Quadrupole-Orbitrap mass spectrometer (ESI). Analytical thin layer chromatography (TLC) was performed with Merck precoated TLC plates, silica gel 60F-254, layer thickness 0.25 mm. Flash chromatography separations were performed on Merck 60 (0.040−0.063 mm) mesh silica gel.

(B) Mechanochemical Bromolactonization Reactions

General Procedure. Alkenoic acid **1** (0.2 mmol) and NBS (39.2 mg, 0.22 mmol) were added into a 10 mL zirconium oxide chamber. The milling chamber was then securely fastened into a Retsch mixer mill (MM 400) and set to oscillate at 30 Hz for 1 hour. On completion of reaction, the mill chamber was extracted with hexane or cyclohexane and the solution was filtered through a thin plug of celite. The filtrate was concentrated under reduced pressure to give the desired product **2** with high purity. No column chromatography was necessary.

5-(bromomethyl)-5-phenyldihydrofuran-2(3H)-one (**2a**)

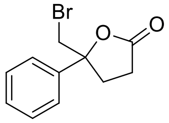

Yield: 97% (49.5 mg, colorless oil)

^{1}H NMR (500 MHz, $CDCl_3$): δ 7.41 (d, *J* = 4.3 Hz, 4H), 7.33-7.39 (m, 1H), 3.74 (d, *J* = 11.4 Hz, 1H), 3.69 (d, *J* = 11.4, 1H), 2.77-2.86 (m, 2H), 2.49-2.61 (m, 2H).

$^{13}C\{^{1}H\}$ NMR (125 MHz, $CDCl_3$): δ 175.7, 140.8, 129.0, 128.8, 125.0, 86.6, 41.2, 32.5, 29.2.

HRMS (ESI-Q-orbitrap) m/z: $[M + Na]^+$ calcd for $C_{11}H_{11}BrO_2$ 276.98346, found 276.98365.

The data are in full accordance with the literature (Chen, T.; Foo, T. J. Y.; Yeung, Y.-Y. *ACS Catal.* **2015**, *5* (8), 4751–4755).

5-(bromomethyl)-5-(p-tolyl)dihydrofuran-2(3H)-one (**2b**)

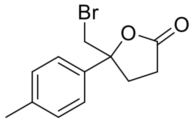

Yield: 98% (52.7 mg, colorless oil)

^{1}H NMR (500 MHz, $CDCl_3$): δ 7.29 (d, *J* = 7.5 Hz, 2H), 7.21 (d, *J* = 7.5 Hz, 2H), 3.73 (d, *J* = 11.1 Hz, 1H), 3.67 (d, *J* = 11.1, 1H), 2.74-2.84 (m, 2H), 2.48-2.58 (m, 2H), 2.36 (s, 3H).

$^{13}C\{^{1}H\}$ NMR (125 MHz, $CDCl_3$): δ 175.8, 138.7, 137.8, 129.6, 125.0, 86.6, 41.2, 32.4, 29.2, 21.2.

HRMS (ESI-Q-orbitrap) m/z: $[M + Na]^+$ calcd for $C_{12}H_{13}BrO_2$ 290.99911, found 290.99917.

The data are in full accordance with the literature (Chen, T.; Foo, T. J. Y.; Yeung, Y.-Y. *ACS Catal.* **2015**, *5* (8), 4751–4755).

5-(bromomethyl)-5-(4-methoxyphenyl)dihydrofuran-2(3H)-one (**2c**)

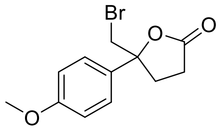

Yield: 87% (49.6 mg, colorless oil)

^{1}H NMR (500 MHz, $CDCl_3$): δ 7.33 (d, *J* = 8.9 Hz, 2H), 6.91 (d, *J* = 8.9 Hz, 2H), 3.81 (s, 3H), 3.71 (d, *J* = 11.3 Hz, 1H), 3.65 (d, *J* = 11.3 Hz, 1H), 2.75-2.83 (m, 2H), 2.50-2.58 (m, 2H).

$^{13}C\{^{1}H\}$ NMR (125 MHz, $CDCl_3$): δ 175.8, 159.8, 132.6, 126.4, 114.3, 86.5, 55.5, 41.3, 32.3, 29.3.

HRMS (ESI-Q-orbitrap) m/z: $[M + Na]^+$ calcd for $C_{12}H_{13}BrO_3$ 306.99403, found 306.99378.

The data are in full accordance with the literature (Zhou, L.; Tan, C. K.; Jiang, X.; Chen, F.; Yeung, Y.-Y. *J. Am. Chem. Soc.* **2010**, *132* (44), 15474–15476.).

5-(bromomethyl)-5-(4-chlorophenyl)dihydrofuran-2(3H)-one (**2d**)

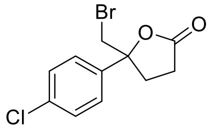

Yield: 98% (56.7 mg, colorless oil)

^{1}H NMR (500 MHz, $CDCl_3$): δ 7.39 (d, *J* = 8.6 Hz, 2H), 7.35 (d, *J* = 8.6 Hz, 2H), 3.70 (d, *J* = 11.3 Hz, 1H), 3.65 (d, *J* = 11.3 Hz, 1H), 2.75-2.86 (m, 2H), 2.48-2.59 (m, 2H).

$^{13}C\{^{1}H\}$ NMR (125 MHz, $CDCl_3$): δ 175.3, 139.4, 134.9, 129.2, 126.6, 86.1, 40.7, 32.5, 29.1.

HRMS (ESI-Q-orbitrap) m/z: $[M + Na]^+$ calcd for $C_{11}H_{10}BrClO_2$ 312.94236, found 312.94231.

The data are in full accordance with the literature (Chen, T.; Foo, T. J. Y.; Yeung, Y.-Y. *ACS Catal.* **2015**, *5* (8), 4751–4755).

5-(bromomethyl)-5-(4-fluorophenyl)dihydrofuran-2(3H)-one (**2e**)

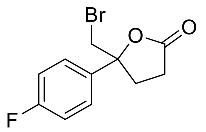

Yield: 90% (49.2 mg, colorless oil)

^{1}H NMR (500 MHz, $CDCl_3$): δ 7.40 (d, *J* = 8.7 Hz, 1H), 7.39 (d, *J* = 8.7 Hz, 1H), 7.10 (t, *J* = 8.7 Hz, 2 H), 3.70 (d, *J* = 11.3 Hz, 1H), 3.65 (d, *J* = 11.3 Hz, 1H), 2.76-2.87 (m, 2H), 2.49-2.60 (m, 2H).

^{13}C{^{1}H} NMR (125 MHz, $CDCl_3$): δ 175.4, 163.8, 161.8, 136.7, 136.6, 127.1, 127.0, 116.0, 115.9, 86.2, 41.0, 32.5, 29.2.

HRMS (ESI-Q-orbitrap) m/z: $[M + Na]^+$ calcd for $C_{11}H_{10}BrFO_2$ 294.97404, found 294.97435.

The data are in full accordance with the literature (Zhou, L.; Tan, C. K.; Jiang, X.; Chen, F.; Yeung, Y.-Y. *J. Am. Chem. Soc.* **2010**, *132* (44), 15474–15476.).

5-(bromomethyl)-5-(4-(trifluoromethyl)phenyl)dihydrofuran-2(3H)-one (**2f**)

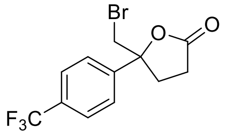

Yield: 98% (63.3 mg, colorless oil)

^{1}H NMR (500 MHz, $CDCl_3$): δ 7.67 (d, *J* = 8.2 Hz, 2H), 7.55 (d, *J* = 8.2 Hz, 2H), 3.72 (d, *J* = 11.5 Hz, 1H), 3.70 (d, *J* = 11.5 Hz, 1H), 2.78-2.88 (m, 2H), 2.50-2.60 (m, 2H).

^{13}C{^{1}H} NMR (125 MHz, $CDCl_3$): δ 175.1, 144.8, 131.0 (q, *J* = 33 Hz), 126.0 (q, *J* = 4 Hz), 125.6, 123.9 (q, *J* = 272 Hz), 86.0, 40.4, 32.6, 29.0.

HRMS (ESI-Q-orbitrap) m/z: $[M + Na]^+$ calcd for $C_{12}H_{10}BrF_3O_2$ 344.97085, found 344.97095.

The data are in full accordance with the literature (Zhou, L.; Tan, C. K.; Jiang, X.; Chen, F.; Yeung, Y.-Y. *J. Am. Chem. Soc.* **2010**, *132* (44), 15474–15476.).

5-(4-acetylphenyl)-5-(bromomethyl)dihydrofuran-2(3H)-one (**2g**)

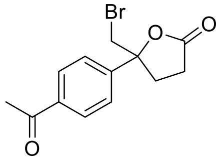

Yield: 81% (48.1 mg, white solid).

IR (KBr): 3020, 1785, 1737, 1685, 1270, 1216, 1162, 760, 668 cm^{-1}.

^{1}H NMR (500 MHz, $CDCl_3$): δ 8.00 (d, *J* = 8.6 Hz, 2H), 7.52 (d, *J* = 8.6 Hz, 2H), 3.73 (d, *J* = 11.4 Hz, 1H), 3.70 (d, *J* = 11.4 Hz, 1H), 2.76-2.89 (m, 2H), 2.62 (s, 3H), 2.50-2.60 (m, 2H).

^{13}C{^{1}H} NMR (125 MHz, $CDCl_3$): δ 197.4, 175.2, 145.8, 137.4, 129.0, 125.4, 86.3, 40.5, 32.6, 29.1, 26.8.

HRMS (ESI-Q-orbitrap) m/z: $[M + Na]^+$ calcd for $C_{13}H_{13}BrO_3$ 318.99403, found 318.99413.

5-bromo-6-phenyltetrahydro-*2H*-pyran-2-one **2h**

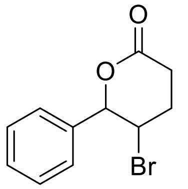

Yield: 78%, d.r. > 99:1 (39.7 mg, white solid)

^{1}H NMR (500 MHz, $CDCl_3$): δ 7.36-7.43 (m, 3H), 7.32 (d, *J* = 6.9 Hz, 2H), 5.56 (d, *J* = 6.4 Hz, 1H), 4.39 (dd, *J* = 11.0, 6.4 Hz, 1H), 2.95 (dt, *J* = 18.2, 8.2 Hz, 1H), 2.72 (dt, *J* = 18.2, 6.2 Hz, 1H), 2.38-2.46 (m, 1H), 2.23-2.31 (m, 1H).

^{13}C{^{1}H} NMR (125 MHz, $CDCl_3$): δ 169.2, 137.4, 129.2, 128.9, 126.5, 85.7, 47.3, 28.5, 27.7.

HRMS (ESI-Q-orbitrap) m/z: $[M + Na]^+$ calcd for $C_{11}H_{11}BrO_2$ 276.98346, found 276.98327.

The data are in full accordance with the literature (Zhou, L.; Tan, C. K.; Jiang, X.; Chen, F.; Yeung, Y.-Y. *J. Am. Chem. Soc.* **2010**, *132* (44), 15474–15476.).

(C) Mechanochemical Bromocycloetherification Reactions

General Procedure. Olefinic mono-alcohol **5** (0.2 mmol) and NBS (39.2 mg, 0.22 mmol) were added into a 10 mL zirconium oxide chamber. The milling chamber was then securely fastened into a Retsch mixer mill (MM 400) and set to oscillate at 30 Hz, for 1 hour. On completion of reaction, the ball mill chamber was extracted with hexane and the solution was filtered through a thin plug of celite. The filtrate was concentrated under reduced pressure to give the desired product **6** in high purity. No column chromatography was necessary.

2-(bromomethyl)-2-phenyltetrahydrofuran (**6a**)

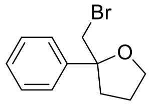

Yield: 98% (47.2 mg, colorless oil).

^{1}H NMR (500 MHz, $CDCl_3$): δ 7.40-7.44 (m, 2H), 7.38-7.38 (m, 2H), 7.26-7.30 (m, 1H), 4.06-4.12 (m, 1H), 3.92-3.97 (m, 1H), 3.66 (s, 2H), 2.40-2.46 (m, 1H), 2.23-2.30 (m, 1H), 2.02-2.10 (m, 1H), 1.80-1.89 (m, 1H).

^{13}C{^{1}H} NMR (125 MHz, $CDCl_3$): δ 144.1, 128.4, 127.5, 125.7, 85.4, 68.8, 42.3, 36.6, 26.3.

The data are in full accordance with the literature (Greb, M.; Hartung, J.; Köhler, F.; Špehar, K.; Kluge, R.; Csuk, R. *European J. Org. Chem.* **2004**, *2004* (18), 3799–3812.).

2-(bromomethyl)-2-(p-tolyl)tetrahydrofuran (**6b**)

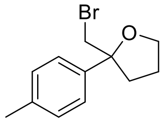

Yield: 99% (50.5 mg, colorless oil).

IR (KBr): 3017, 1216, 750 cm^{-1}.

^{1}H NMR (500 MHz, $CDCl_3$): δ 7.31 (d, *J* = 7.5 Hz, 2H), 7.17 (d, *J* = 7.5 Hz, 2H), 4.08 (dd, *J* = 14.5, 7.3 Hz, 1H), 3.93 (dd, *J* = 14.5, 7.3 Hz, 1H), 3.64 (s, 2H), 2.37-2.44 (m, 1H), 2.35 (s, 3H), 2.21-2.28 (m, 1H), 2.00-2.09 (m, 1H), 1.80-1.89 (m, 1H).

$^{13}C\{^{1}H\}$ NMR (125 MHz, $CDCl_3$): δ 141.1, 137.2, 129.1, 125.6, 85.3, 68.7, 42.4, 36.5, 26.3, 21.2.

HRMS (ESI-Q-orbitrap) m/z: $[M-H_2O+H]^+$ calcd for $C_{12}H_{15}BrO$ 237.02734, found 237.02724.

2-(bromomethyl)-2-(4-methoxyphenyl)tetrahydrofuran (**6c**)

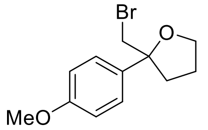

Yield: 76% (41.2 mg, colorless oil).

IR (KBr): 3019, 1510, 1249, 1216, 763, 669 cm^{-1}.

^{1}H NMR (500 MHz, $CDCl_3$): δ 7.33 (d, *J* = 8.7 Hz, 2H), 6.88 (d, *J* = 8.7 Hz, 2H), 4.07 (dd, *J* = 14.7, 7.4 Hz, 1H), 3.92 (dd, *J* = 14.7, 7.4 Hz, 1H), 3.81 (s, 3H), 3.62 (s, 2H), 2.36-2.43 (m, 1H), 2.20-2.67 (m, 1H), 2.00-2.09 (m, 1H), 1.809-1.89 (m, 1H).

$^{13}C\{^{1}H\}$ NMR (125 MHz, $CDCl_3$): δ 159.0, 136.0, 126.9, 113.7, 85.1, 68.7, 55.4, 42.5, 36.4, 26.3.

HRMS (ESI-Q-orbitrap) m/z: $[M-H_2O+H]^+$ calcd for $C_{12}H_{15}BrO_2$ 253.02225, found 253.02216.

2-(bromomethyl)-2-(4-chlorophenyl)tetrahydrofuran (**6d**)

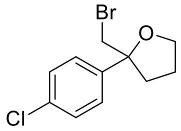

Yield: 90% (49.6 mg, colorless oil).

IR (KBr): 3018, 1490, 1216, 1052, 752 cm^{-1}.

^{1}H NMR (500 MHz, $CDCl_3$): δ 7.35 (d, *J* = 8.0 Hz, 2H), 7.32 (d, *J* = 8.0 Hz, 2H), 4.08 (dd, *J* = 14.5, 7.2 Hz, 1H), 3.92 (dd, *J* = 14.5, 7.2 Hz, 1H), 3.60 (s, 2H), 2.36-2.43 (m, 1H), 2.16-2.25 (m, 1H), 2.01-2.10 (m, 1H), 1.79-1.89 (m, 1H).

$^{13}C\{^1H\}$ NMR (125 MHz, $CDCl_3$): δ 142.6, 133.4, 128.5, 127.2, 85.1, 68.9, 41.9, 36.7, 26.2.

HRMS (ESI-Q-orbitrap) m/z: $[M+H]^+$ calcd for $C_{11}H_{12}BrClO$ 276.98107, found 276.98107.

2-(bromomethyl)-2-(4-fluorophenyl)tetrahydrofuran (**6e**)

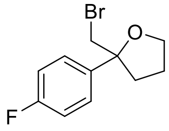

Yield: 93% (48.2 mg, colorless oil).

IR (KBr): 3017, 2981, 1604, 1508, 1210, 1052, 838, 756 cm^{-1}.

^1H NMR (500 MHz, $CDCl_3$): δ 7.38 (m, 2H), 7.03 (t, J = 8.3 Hz, 2H), 4.08 (dd, J = 14.5, 7.4 Hz, 1H), 3.92 (dd, J = 14.5, 7.4 Hz, 1H), 3.61 (s, 2H), 2.37-2.44 (m, 1H), 2.19-2.26 (m, 1H), 2.01-2.10 (m, 1H), 1.80-1.89 (m, 1H).

$^{13}C\{^1H\}$ NMR (125 MHz, $CDCl_3$): δ 163.2, 161.2, 139.8, 139.7, 127.5, 127.4, 115.3, 115.1, 85.1, 68.8, 42.1, 36.7, 26.2.

HRMS (ESI-Q-orbitrap) m/z: $[M+H]^+$ calcd for $C_{11}H_{12}BrFO$ 259.01283, found 259.01287.

2-(bromomethyl)-2-(4-(trifluoromethyl)phenyl)tetrahydrofuran (**6f**)

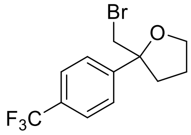

Yield: 98% (60.6 mg, colorless oil).

^1H NMR (500 MHz, $CDCl_3$): δ 7.61 (d, J = 8.3 Hz, 2H), 7.54 (d, J = 8.3 Hz, 2H), 4.08-4.13 (m, 1H), 3.92-3.97 (m, 1H), 3.63 (d, J = 0.9 Hz, 2H), 2.41-2.47 (m, 1H), 2.21-2.28 (m, 1H), 2.04-2.12 (m, 1H), 1.80-1.90 (m, 1H).

$^{13}C\{^1H\}$ NMR (125 MHz, $CDCl_3$): δ 148.2, 129.8 (q, J = 32 Hz), 126.2, 125.4 (q, J = 4 Hz), 124.4 (q, J = 271 Hz), 85.3, 69.0, 41.6, 36.9, 26.2.

The data are in full accordance with the literature (Greb, M.; Hartung, J.; Köhler, F.; Špehar, K.; Kluge, R.; Csuk, R. *European J. Org. Chem.* **2004**, *2004* (18), 3799–3812.).

2-(3,5-bis(trifluoromethyl)phenyl)-2-(bromomethyl)tetrahydrofuran (**6g**)

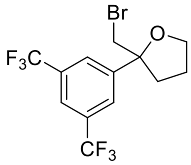

Yield: 98% (73.9 mg, colorless oil).

IR (KBr): 3019, 1377, 1280, 1216, 1180, 1139, 1055, 900, 844, 757, 669 cm^{-1}.

^{1}H NMR (500 MHz, $CDCl_3$): δ 7.89 (s, 2H), 7.81 (s, 1H), 4.11-4.16 (m, 1H), 3.95-4.00 (m, 1H), 3.63 (d, *J* = 10.8 Hz, 1 H), 3.59 (d, *J* = 10.8, 1H), 2.47-2.54 (m, 1H), 2.24-2.30 (m, 1H), 2.08-2.17 (m, 1H), 1.84-1.93 (m, 1H).

^{13}C{^{1}H} NMR (125 MHz, $CDCl_3$): δ 147.0, 131.7 (q, *J* = 33 Hz), 126.2, 123.4 (q, *J* = 273 Hz), 121.6-121.8 (m), 85.0, 69.2, 41.0, 37.1, 26.3.

(D) Mechanochemical Intermolecular Bromoesterification Reactions

General Procedure. Carboxylic acid **7** (0.2 mmol), alkene **8** (0.22 mmol) and DBDMH (62.9 mg, 0.22 mmol) were added into a 10 mL zirconium oxide chamber. The milling chamber was then securely fastened into a Retsch mixer mill (MM 400) and set to oscillate at 30 Hz, for 1 hour. On completion of reaction, the ball mill chamber was extracted with hexane and the solution was filtered through a thin plug of celite. The filtrate was concentrated under reduced pressure to give the desired product **9**. Purification using flash chromatography through silica gel (hexane/ethyl acetate 40:1) was used to obtain the final product.

2-bromo-1-phenylethyl benzoate (**9aa**)

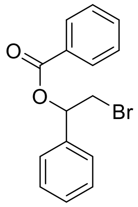

Yield: 92% (56.2 mg, colorless oil, R_f = 0.47, hexane/ethyl acetate = 10:1).

^{1}H NMR (500 MHz, $CDCl_3$): δ 8.12-8.16 (m, 2H), 7.60 (tt, *J* = 7.4, 1.2 Hz, 1H), 7.44-7.51 (m, 4H), 7.34-7.43 (m, 3H), 6.24 (dd, *J* = 8.0, 4.5 Hz, 1H), 3.83 (dd, *J* = 10.9, 8.0 Hz, 1H), 3.75 (dd, *J* = 10.9, 4.5 Hz, 1H).

^{13}C{^{1}H} NMR (125 MHz, $CDCl_3$): δ 165.5, 137.9, 133.4, 129.9, 129.8, 128.6, 126.6, 75.4, 34.6.

HRMS (ESI-Q-orbitrap) m/z: $[M + Na]^+$ calcd for $C_{15}H_{13}BrO_2$ 326.99911, found 326.99884.

The data are in full accordance with the literature (Ng, W.-H.; Hu, R.-B.; Lam, Y.-P.; Yeung, Y.-Y. *Org. Lett.* **2020**, *22* (14), 5572–5576.).

2-bromo-1-(p-tolyl)ethyl benzoate (**9ab**)

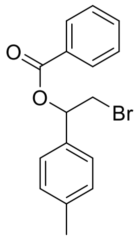

Yield: 93% (59.3 mg, colorless oil, R_f = 0.30, hexane/dichloromethane = 2:1).

^{1}H NMR (500 MHz, $CDCl_3$): δ 8.11 (d, *J* = 7.5 Hz, 2H), 7.58 (t, *J* = 7.6 Hz, 1H), 7.46 (t, *J* = 7.6 Hz, 2H), 7.34 (d, *J* = 7.8 Hz, 2H), 7.20 (d, *J* = 7.8 Hz, 2H), 6.18 (dd, *J* = 8.0, 4.5 Hz, 1H), 3.81 (dd, *J* = 10.8, 8.3 Hz, 1H), 3.71 (dd, *J* = 10.8, 4.5 Hz, 1H), 2.35 (s, 1H).

^{13}C{^{1}H} NMR (125 MHz, $CDCl_3$): δ 165.6, 138.9, 134.9, 133.4, 130.0, 129.6, 128.6, 126.6, 75.5, 34.6, 21.4.

HRMS (ESI-Q-orbitrap) m/z: [M + Na]$^+$ calcd for $C_{16}H_{15}BrO_2$ 341.01476, found 341.01511.

The data are in full accordance with the literature (Ng, W.-H.; Hu, R.-B.; Lam, Y.-P.; Yeung, Y.-Y. *Org. Lett.* **2020**, *22* (14), 5572–5576.).

2-bromo-1-mesitylethyl benzoate (**9ac**)

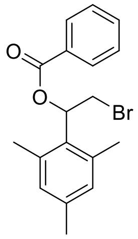

Yield: 99% (68.5 mg, colorless oil, R_f = 0.40, hexane/ethyl acetate = 20:1).

^{1}H NMR (500 MHz, $CDCl_3$): δ 8.11-8.14 (m, 2H), 7.57-7.61 (m, 1H), 7.46-7.50 (m, 2H), 6.87 (s, 2H), 6.62 (dd, *J* = 10.1, 4.6 Hz, 1H), 4.07 (dd, *J* = 11.0, 10.1 Hz, 1H), 3.69 (dd, *J* = 11.0, 4.6 Hz, 1H), 2.55 (s, 6H), 2.27 (s, 3H).

^{13}C{^{1}H} NMR (125 MHz, $CDCl_3$): δ 165.6, 138.4, 136.8, 133.2, 130.6, 130.4, 129.9, 129.8, 128.6, 73.5, 32.0, 21.0, 20.8.

HRMS (ESI-Q-orbitrap) m/z: [M + Na]$^+$ calcd for $C_{18}H_{19}BrO_2$ 369.04606, found 369.04625.

The data are in full accordance with the literature (Ng, W.-H.; Hu, R.-B.; Lam, Y.-P.; Yeung, Y.-Y. *Org. Lett.* **2020**, *22* (14), 5572–5576.).

2-bromo-1-(4-(tert-butyl)phenyl)ethyl benzoate (**9ad**)

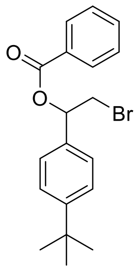

Yield: 90% (65.0 mg, colorless oil, R_f = 0.37, hexane/dichloromethane = 2:1).

^{1}H NMR (500 MHz, $CDCl_3$): δ 8.13 (d, *J* = 7.6 Hz, 2H), 7.58 (t, *J* = 7.4 Hz, 1H), 7.47 (t, *J* = 7.6 Hz, 2H), 7.36-7.43 (m, 4H), 6.21 (dd, *J* = 8.3, 4.3 Hz, 1H), 3.82 (dd, *J* = 10.9, 8.6 Hz, 1H), 3.73 (dd, *J* = 10.9, 4.4 Hz, 1H), 1.31 (s, 9H).

^{13}C{^{1}H} NMR (125 MHz, $CDCl_3$): δ 165.6, 152.0, 134.8, 133.4, 130.0, 128.6, 126.4, 125.8, 75.4, 34.8, 34.6, 31.4.

HRMS (ESI-Q-orbitrap) m/z: $[M + Na]^+$ calcd for $C_{19}H_{21}BrO_2$ 383.06171, found 383.06263.

The data are in full accordance with the literature (Ng, W.-H.; Hu, R.-B.; Lam, Y.-P.; Yeung, Y.-Y. *Org. Lett.* **2020**, *22* (14), 5572–5576.).

2-bromo-1-(4-bromophenyl)ethyl benzoate (**9ae**)

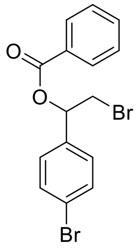

Yield: 89% (68.3 mg, colorless oil, R_f = 0.30, hexane/dichloromethane = 2:1).

^{1}H NMR (500 MHz, $CDCl_3$): δ 8.09-8.13 (m, 2H), 7.58-7.62 (m, 1H), 7.51-7.54 (m, 2H), 7.46-7.50 (m, 2H), 7.31-7.35 (m, 2H), 6.16 (dd, *J* = 7.5, 4.9 Hz, 1H), 3.78 (dd, *J* = 10.9, 7.5 Hz, 1H), 3.71 (dd, *J* = 10.9, 4.9 Hz, 1H).

^{13}C{^{1}H} NMR (125 MHz, $CDCl_3$): δ 165.4, 136.9, 133.6, 132.1, 129.9, 129.6, 128.7, 128.4, 123.0, 74.7, 34.1.

HRMS (ESI-Q-orbitrap) m/z: $[M + Na]^+$ calcd for $C_{15}H_{12}Br_2O_2$ 406.90765, found 406.90751.

The data are in full accordance with the literature (Ng, W.-H.; Hu, R.-B.; Lam, Y.-P.; Yeung, Y.-Y. *Org. Lett.* **2020**, *22* (14), 5572–5576.).

2-bromo-1-(4-chlorophenyl)ethyl benzoate (**9af**)

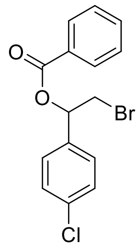

Yield: 88% (59.8 mg, colorless oil, R_f = 0.27, hexane/dichloromethane = 2:1).

^{1}H NMR (500 MHz, $CDCl_3$): δ 8.10-8.14 (m, 2H), 7.58-7.62 (m, 1H), 7.46-7.50 (m, 2H), 7.35-7.41 (m, 4H), 6.19 (dd, *J* = 7.5, 4.9 Hz, 1H), 3.79 (dd, *J* = 10.9, 7.5 Hz, 1H), 3.72 (dd, *J* = 10.9, 4.9 Hz, 1H).

$^{13}C\{^1H\}$ NMR (125 MHz, $CDCl_3$): δ 165.4, 136.3, 134.8, 133.6, 129.9, 129.6, 129.1, 128.6, 128.1, 74.7, 34.2.

HRMS (ESI-Q-orbitrap) m/z: $[M + Na]^+$ calcd for $C_{15}H_{12}BrClO_2$ 362.95798, found 362.95726.

The data are in full accordance with the literature (Ng, W.-H.; Hu, R.-B.; Lam, Y.-P.; Yeung, Y.-Y. *Org. Lett.* **2020**, *22* (14), 5572–5576.).

2-bromo-1-(4-fluorophenyl)ethyl benzoate (**9ag**)

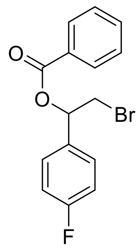

Yield: 93% (60.1 mg, colorless oil, R_f = 0.24, hexane/dichloromethane = 2:1).

IR (KBr): 3020, 1722, 1511, 1268, 1216, 1109, 757, 669 cm^{-1}.

^{1}H NMR (500 MHz, $CDCl_3$): δ 8.09-8.12 (m, 2H), 7.58-7.62 (m, 1H), 7.41-7.50 (m, 4H), 7.05-7.11 (m, 2H), 6.19 (dd, *J* = 7.6, 4.8 Hz, 1H), 3.79 (dd, *J* = 10.9, 7.6 Hz, 1H), 3.71 (dd, *J* = 10.9, 4.8 Hz, 1H).

^{13}C{^{1}H} NMR (125 MHz, $CDCl_3$): δ 165.5, 164.0, 162.0, 133.8, 133.7, 133.6, 130.0, 129.7, 128.7, 128.6, 128.5, 116.0, 115.8, 74.8, 34.4.

HRMS (ESI-Q-orbitrap) m/z: $[M + Na]^+$ calcd for $C_{15}H_{12}BrFO_2$ 344.98969, found 344.98986.

2-bromocyclohexyl benzoate (**9ah**)

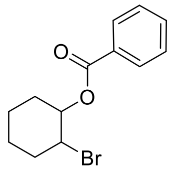

Yield: 86% (48.6 mg, colorless oil, R_f = 0.17, pure hexane).

^{1}H NMR (500 MHz, $CDCl_3$): δ 8.07 (d, *J* = 7.5 Hz, 2H), 7.57 (t, *J* = 7.4 Hz, 1H), 7.45 (t, *J* = 7.7 Hz, 2H), 5.10-5.16 (m, 1H), 4.13-4.19 (m, 1H), 2.38-2.45 (m, 1H), 2.24-2.33 (m, 1H), 1.90-2.00 (m, 1H), 1.74-1.87 (m, 2H), 1.47-1.57 (m, 2H), 1.35-1.45 (m, 1H).

^{13}C{^{1}H} NMR (125 MHz, $CDCl_3$): δ 165.7, 133.2, 130.3, 129.9, 128.5, 76.5, 52.8, 35.7, 31.2, 25.6, 23.4.

HRMS (ESI-Q-orbitrap) m/z: $[M + Na]^+$ calcd for $C_{13}H_{15}BrO_2$ 305.01476, found 305.01457.

The data are in full accordance with the literature (Ng, W.-H.; Hu, R.-B.; Lam, Y.-P.; Yeung, Y.-Y. *Org. Lett.* **2020**, *22* (14), 5572–5576.).

2-bromo-1-phenylethyl 2,4-dimethylbenzoate (**9ba**)

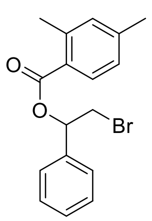

Yield: 82% (54.6 mg, colorless oil, R_f = 0.57, hexane/dichloromethane = 1:1).

IR (KBr): 3019, 1717, 1615, 1256, 1216, 756 cm^{-1}.

^{1}H NMR (500 MHz, $CDCl_3$): δ 7.99 (d, *J* = 7.8 Hz, 1H), 7.33-7.47 (m, 5H), 7.10 (d, *J* = 8.8 Hz, 2H), 6.20 (dd, *J* = 8.0, 4.6 Hz, 1H), 3.80 (dd, *J* = 10.9, 8.0 Hz, 1H), 3.73 (10.9, 4.6 Hz, 1H), 2.60 (s, 3H), 2.38 (s, 3H).

^{13}C{^{1}H} NMR (125 MHz, $CDCl_3$): δ 166.2, 143.1, 141.0, 138.1, 132.7, 131.2, 128.9, 126.7, 126.2, 75.2, 34.8, 22.1, 21.6.

HRMS (ESI-Q-orbitrap) m/z: $[M + Na]^+$ calcd for $C_{17}H_{17}BrO_2$ 355.03041, found 355.03042.

2-bromo-1-phenylethyl 2,4,6-trimethylbenzoate (**9ca**)

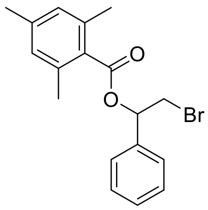

Yield: 90% (62.5 mg, colorless oil, R_f = 0.43, hexane/ethyl acetate = 20:1).

IR (KBr): 3020, 1726, 1612, 1262, 1216, 1167, 1079, 756, 669 cm^{-1}.

^{1}H NMR (500 MHz, $CDCl_3$): δ 7.42-7.46 (m, 2H), 7.35-7.42 (m, 3H), 6.86 (s, 2H), 6.25 (dd, *J* = 8.7, 4.2 Hz, 1H), 3.78 (dd, *J* = 11.0, 8.7 Hz, 1H), 3.67 (dd, *J* = 11.0, 4.2 Hz, 1H), 2.29 (s, 3H), 2.24 (s, 6H).

^{13}C{^{1}H} NMR (125 MHz, $CDCl_3$): δ 169.2, 139.6, 137.7, 135.3, 130.6, 129.1, 128.9, 128.5, 127.1, 76.1, 34.0, 21.3, 19.9.

HRMS (ESI-Q-orbitrap) m/z: $[M + Na]^+$ calcd for $C_{18}H_{19}BrO_2$ 369.04606, found 369.04633.

2-bromo-1-phenylethyl 2-bromobenzoate (**9da**)

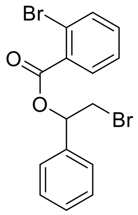

Yield: 90% (69.1 mg, colorless oil, R_f = 0.32, hexane/dichloromethane = 2:1).

IR (KBr): 3019, 1735, 1249, 1215, 755, 669 cm^{-1}.

^{1}H NMR (500 MHz, $CDCl_3$): δ 7.92 (dd, *J* = 7.6, 1.6 Hz, 1H), 7.68 (d, *J* = 7.9 Hz, 1H), 7.46 (d, *J* = 7.6 Hz, 2H), 7.33-7.43 (m, 5H), 6.23 (dd, *J* = 8.0, 4.6 Hz, 1H), 3.82 (dd, *J* = 11.0, 8.0 Hz, 1H), 3.73 (dd, *J* = 11.0, 4.6 Hz, 1H).

$^{13}C\{^{1}H\}$ NMR (125 MHz, $CDCl_3$): δ 164.9, 137.4, 134.7, 133.0, 131.8, 131.6, 129.1, 128.9, 127.4, 126.9, 122.1, 76.3, 34.3.

HRMS (ESI-Q-orbitrap) m/z: $[M + Na]^+$ calcd for $C_{15}H_{12}Br_2O_2$ 406.90765, found 406.90749.

2-bromo-1-phenylethyl 2-fluorobenzoate (**9ea**)

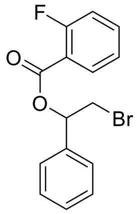

Yield: 99% (63.9 mg, colorless oil, R_f = 0.24, hexane/dichloromethane = 2:1).

IR (KBr): 3020, 1718, 1615, 1216, 756 cm^{-1}.

^{1}H NMR (500 MHz, $CDCl_3$): δ 8.02 (td, *J* = 7.6, 1.8 Hz, 1H), 7.52-7.57 (m, 1H), 7.45-7.49 (m, 2H), 7.34-7.42 (m, 3H), 7.23 (td, *J* = 7.8, 1.1 Hz, 1H), 7.16 (ddd, *J* = 10.8, 8.4, 0.9 Hz, 1H), 6.25 (dd, *J* = 8.1, 4.5 Hz, 1H), 3.81 (dd, *J* = 10.9, 8.1 Hz, 1H), 3.72 (dd, *J* = 10.9, 4.5 Hz, 1H).

$^{13}C\{^{1}H\}$ NMR (125 MHz, $CDCl_3$): δ 163.3, 163.3, 163.2, 161.2, 137.6, 135.0, 134.9, 132.4, 129.0, 128.9, 128.9, 126.8, 124.2, 124.1, 118.4, 118.3, 117.3,117.1, 75.9, 34.5.

HRMS (ESI-Q-orbitrap) m/z: $[M + Na]^+$ calcd for $C_{15}H_{12}BrFO_2$ 344.98969, found 344.98977.

2-bromo-1-phenylethyl 4-nitrobenzoate (**9fa**)

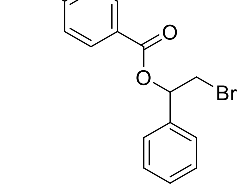

Yield: 69% (48.3 mg, colorless oil, R_f = 0.20, hexane/dichloromethane = 2:1).

^{1}H NMR (500 MHz, $CDCl_3$): δ 8.26-8.33 (m, 4H), 7.36-7.46 (m, 5H), 6.24 (dd, *J* = 8.4, 4.3 Hz, 1H), 3.84 (dd, *J* = 11.1, 8.4 Hz, 1H), 3.75 (dd, *J* = 11.1, 4.3 Hz, 1H).

^{13}C{^{1}H} NMR (125 MHz, $CDCl_3$): δ 163.7, 150.9, 137.2, 135.2, 131.1, 129.4, 129.1, 126.7, 123.8, 76.6, 34.1.

HRMS (ESI-Q-orbitrap) m/z: $[M + Na]^+$ calcd for $C_{15}H_{12}BrNO_4$ 371.98419, found 371.98425.

The data are in full accordance with the literature (Ng, W.-H.; Hu, R.-B.; Lam, Y.-P.; Yeung, Y.-Y. *Org. Lett.* **2020**, *22* (14), 5572–5576.).

2-bromo-1-phenylethyl 3-phenylpropanoate (**9ga**)

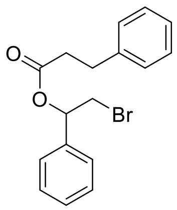

Yield: 96% (64.0 mg, yellow oil, R_f = 0.33, hexane/ethyl acetate = 20:1).

^{1}H NMR (500 MHz, $CDCl_3$): δ 7.36-7.41 (m, 3H), 7.28-7.35 (m, 4H), 7.20-7.25 (m, 3H), 6.03 (dd, *J* = 8.2, 4.7 Hz, 1H), 3.65 (dd, *J* =10.8, 8.2 Hz, 1H), 3.59 (dd, *J* = 10.8, 4.7 Hz, 1H), 2.97-3.07 (m, 2H), 2.70-2.83 (m, 2H).

^{13}C{^{1}H} NMR (125 MHz, $CDCl_3$): δ 171.8, 140.3, 137.7, 128.9, 128.8, 128.6, 128.4, 126.6, 126.4, 75.0, 35.9, 34.3, 30.9.

HRMS (ESI-Q-orbitrap) m/z: $[M + Na]^+$ calcd for $C_{17}H_{17}BrO_2$ 355.03041, found 355.03031.

The data are in full accordance with the literature (Shi, Y.; Wong, J.; Ke, Z.; Yeung, Y.-Y. *J. Org. Chem.* **2019**, *84* (7), 4017–4024.).

(E) Synthesis of olefinic acid substrates 1

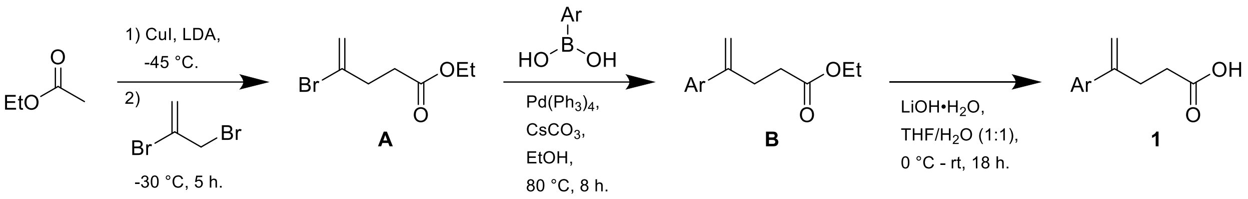

General procedure.

1. Preparation of LDA solution: n-Butyllithium solution 1.58 M in hexanes (47.5 mL, 75.0 mmol) was added to a solution of diisopropylamine (10.5 mL, 75.0 mmol) in THF (25.0 mL) at -78 °C. The resultant mixture was allowed to warm to room temperature over 2 hours.

2. Enolate alkylation: The LDA solution was added to a suspension of ethyl acetate (7.3 mL, 75.0 mmol) and copper (I) iodide (28.6 g, 150.0 mmol) in THF (75.0 mL) at -45 °C. The resultant solution was warmed to -30 °C. A solution of 2,3-dibromopropene (3.7 mL, 37.5 mmol) in THF (37.5 mL) was then added dropwise. The resultant mixture was stirred for an additional 5 hours at -30 °C. The reaction was then quenched using a saturated aqueous ammonium chloride solution (20.0 mL), acidified to pH 2 with 1 M aqueous hydrogen chloride solution, and extracted with diethyl ether (3 x 20.0 mL). The organic extracts were then washed with 28% aqueous ammonia solution (3 x 10.0 mL), dried over anhydrous sodium sulfate, filtereted, and concentrated *in vacuo* to give the intermediate **A**; which was used in the next step without further purification.

3. Suzuki coupling: A mixture of intermediate **A** (2.1 g, 10.0 mmol), aryl boronic acid (11.0 mmol), tetrakis(triphenylphosphine)-palladium(0) (0.6 g, 0.5 mmol), cesium carbonate (4.9 g, 15.0 mmol), in ethanol (50 mL) was degassed with nitrogen in a resealable tube. The sealed system was then heated to 80 °C for 8 hours. The resultant reaction mixture was filtered, concentrated *in vacuo* and purification by flash column chromatography in silica gel (hexane/ethyl acetate, 20:1) to give the intermediate **B**.

4. Ester hydrolysis: To a suspension of intermediate **B** (6.0 mmol), in a 1:1 mixture of THF and water (60.0 mL) at 0 °C, lithium hydroxide monohydrate (1.3 g, 30.0 mmol) was added in portions. The resultant mixture was warmed to room temperature and stirred for 18 hours. The product mixture was then acidified to pH < 4 with a 1 M hydrogen chloride aqueous solution, extracted with diethyl ether (3 x 10.0 mL) and washed with brine (3 x 5.0 mL). The organic fractions were concentrated in vacuo and recrystallized from a mixture of diethyl ether and hexane to give the alkenoic acid **1**.

4-phenylpent-4-enoic acid (**1a**)

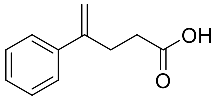

White solid.

^{1}H NMR (500 MHz, $CDCl_3$): δ 10.50 (br. s, 1H), 7.41 (d, *J* = 7.3 Hz, 2H), 7.34 (t, *J* = 7.3 Hz, 2H), 7.27-7.31 (m, 1H), 5.33 (s, 1H), 5.12 (s, 1H), 2.86 (t, *J* = 7.7 Hz, 2H), 2.54 (t, *J* = 7.7 Hz, 2H).

$^{13}C\{^{1}H\}$ NMR (125 MHz, $CDCl_3$): δ 179.4, 146.6, 140.5, 128.6, 127.8, 126.2, 113.1, 33.1, 30.3.

HRMS (ESI-Q-orbitrap) m/z: $[M - H]^-$ calcd for $C_{11}H_{12}O_2$ 175.07645, found 175.07630.

The data are in full accordance with the literature (Zhou, L.; Tan, C. K.; Jiang, X.; Chen, F.; Yeung, Y.-Y. *J. Am. Chem. Soc.* **2010**, *132* (44), 15474–15476.).

4-(p-tolyl)pent-4-enoic acid (**1b**)

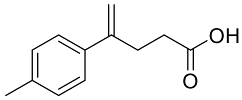

White solid.

^{1}H NMR (500 MHz, $CDCl_3$): δ 10.91 (br. s, 1H), 7.32 (d, *J* = 7.4 Hz, 2H), 7.16 (d, *J* = 7.4 Hz, 2H), 5.31 (s, 1H), 5.08 (s, 1H), 2.85 (t, *J* = 7.5 Hz, 2H), 2.55 (t, *J* = 7.5 Hz, 2H), 2.36 (s, 3H).

$^{13}C\{^{1}H\}$ NMR (125 MHz, $CDCl_3$): δ 179.8, 146.4, 137.6, 137.5, 129.2, 126.1, 112.3, 33.2, 30.2, 21.2.

HRMS (ESI-Q-orbitrap) m/z: $[M - H]^-$ calcd for $C_{12}H_{14}O_2$ 189.09210, found 189.09202.

The data are in full accordance with the literature (Zhou, L.; Tan, C. K.; Jiang, X.; Chen, F.; Yeung, Y.-Y. *J. Am. Chem. Soc.* **2010**, *132* (44), 15474–15476.).

4-(4-methoxyphenyl)pent-4-enoic acid (**1c**)

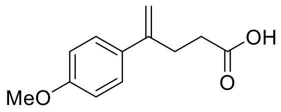

White solid.

^{1}H NMR (500 MHz, $CDCl_3$): δ 7.34 (d, *J* = 7.6 Hz, 2H), 6.87 (d, *J* = 7.6 Hz, 2H), 5.25 (s, 1H), 5.02 (s, 1H), 3.81 (s, 3H), 2.82 (t, *J* = 7.7 Hz, 2H), 2.52 (t, *J* = 7.7 Hz, 2H).

^{13}C{^{1}H} NMR (125 MHz, $(CD_3)_2SO$): δ 174.1, 159.0, 145.8, 132.4, 127.1, 114.0, 110.8, 55.3, 32.9, 29.8.

HRMS (ESI-Q-orbitrap) m/z: $[M+Na]^+$ calcd for $C_{12}H_{14}O_3$ 229.08352, found 229.08351.

The data are in full accordance with the literature (Zhou, L.; Tan, C. K.; Jiang, X.; Chen, F.; Yeung, Y.-Y. *J. Am. Chem. Soc.* **2010**, *132* (44), 15474–15476.).

4-(4-chlorophenyl)pent-4-enoic acid (**1d**)

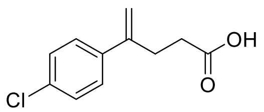

White solid.

^{1}H NMR (500 MHz, $CDCl_3$): δ 9.25 (br. s, 1H), 7.28-7.35 (m, 4H), 5.31 (s, 1H), 5.12 (m, 1H), 2.81 (t, *J* = 7.5 Hz, 2H), 2.52 (t, *J* = 7.5 Hz, 2H).

^{13}C{^{1}H} NMR (125 MHz, $CDCl_3$): δ 179.1, 145.5, 139.0, 133.6, 128.7, 127.5, 113.7, 32.9, 30.1.

HRMS (ESI-Q-orbitrap) m/z: $[M - H]^-$ calcd for $C_{11}H_{11}ClO_2$ 209.03748, found 209.03744.

The data are in full accordance with the literature (Zhou, L.; Tan, C. K.; Jiang, X.; Chen, F.; Yeung, Y.-Y. *J. Am. Chem. Soc.* **2010**, *132* (44), 15474–15476.).

4-(4-fluorophenyl)pent-4-enoic acid (**1e**)

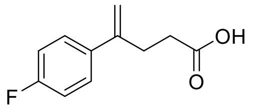

^{1}H NMR (500 MHz, $CDCl_3$): δ 11.02 (br. s, 1H), 7.37 (t, *J* = 6.3 Hz, 2H), 7.03 (t, *J* = 7.9 Hz, 2H), 5.28 (s, 1H), 5.10 (s, 1H), 2.82 (t, *J* = 7.5 Hz, 2H), 2.53 (t, *J* = 7.5 Hz, 2H).

^{13}C{^{1}H} NMR (125 MHz, $CDCl_3$): δ 179.8, 163.5, 161.6, 145.6, 136.6, 136.6, 127.9, 127.8, 115.5, 115.3, 113.1, 33.0, 30.3.

HRMS (ESI-Q-orbitrap) m/z: [M - H]$^-$ calcd for $C_{11}H_{11}FO_2$ 193.06703, found 193.06691.

4-(4-(trifluoromethyl)phenyl)pent-4-enoic acid (**1f**)

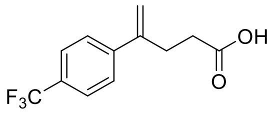

White solid.

^{1}H NMR (500 MHz, $CDCl_3$): δ 7.59 (d, *J* = 7.9 Hz, 2H), 7.50 (d, *J* = 7.9 Hz, 2H), 5.39 (s, 1H), 5.21 (s, 1H), 2.85 (t, *J* = 7.6 Hz, 2H), 2.53 (t, *J* = 7.6 Hz, 2H).

^{13}C{^{1}H} NMR (125 MHz, $CDCl_3$): δ 178.7, 145.6, 144.2, 129.8 (q, *J* = 32 Hz), 126.6, 125.6 (q, *J* = 3.6 Hz), 115.1, 32.8, 30.1.

HRMS (ESI-Q-orbitrap) m/z: [M - H]$^-$ calcd for $C_{12}H_{12}F_3O_2$ 243.06384, found 243.06371.

The data are in full accordance with the literature (Zhou, L.; Tan, C. K.; Jiang, X.; Chen, F.; Yeung, Y.-Y. *J. Am. Chem. Soc.* **2010**, *132* (44), 15474–15476.).

4-(4-acetylphenyl)pent-4-enoic acid (**1g**)

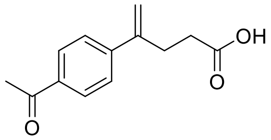

White solid.

^{1}H NMR (500 MHz, $CDCl_3$): δ 7.93 (d, *J* = 7.6 Hz, 2H), 7.49 (d, *J* = 7.4 Hz, 2H), 5.43 (s, 1H), 5.23 (s, 1H), 2.87 (t, *J* = 7.6 Hz, 2H), 2.61 (s, 3H), 2.54 (t, *J* = 7.6 Hz, 2H).

^{13}C{^{1}H} NMR (125 MHz, $CDCl_3$): δ 197.8, 177.6, 145.8, 145.3, 136.4, 128.7, 126.4, 115.2, 32.7, 30.0, 26.8.

HRMS (ESI-Q-orbitrap) m/z: [M+Na]$^+$ calcd for $C_{13}H_{14}O_3$ 241.08345, found 241.08342.

(*E*)-5-phenylpent-4-enoic acid **1h**

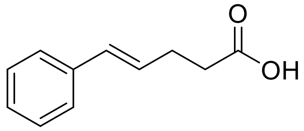

White solid.

^{1}H NMR (500 MHz, $CDCl_3$): δ 7.35 (d, *J* = 7.7 Hz, 2H), 7.30 (t, *J* = 7.7 Hz, 2H), 7.21 (t, *J* = 7.1 Hz, 1H), 6.45 (d, *J* = 15.9 Hz, 1H), 6.18-6.25 (m, 1H), 2.54-2.57 (m, 4H).

^{13}C{^{1}H} NMR (125 MHz, $CDCl_3$): δ 179.1, 137.4, 131.3, 128.7, 128.1, 127.4, 126.2, 33.9, 28.0.

HRMS (ESI-Q-orbitrap) m/z: $[M-H]^-$ calcd for $C_{11}H_{12}O_2$ 175.07645, found 175.07651.

The data are in full accordance with the literature (Zhou, L.; Tan, C. K.; Jiang, X.; Chen, F.; Yeung, Y.-Y. *J. Am. Chem. Soc.* **2010**, *132* (44), 15474–15476.).

Synthesis of olefinic mono-alcohol 5

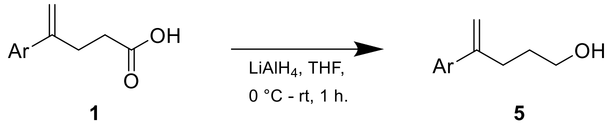

General procedure. To a solution of **1** (3.0 mmol) in anhydrous THF (6.0 mL) at 0 ̊C lithium aluminum hydride (0.2 g, 6.0 mmol) was added in portions. The resultant suspension was warmed to room temperature and stirred for 1 hour. The reaction was then quenched with a 2 M aqueous solution of sodium hydroxide (5.0 mL), filtered through a thin pad of celite, and extracted with diethyl ether (3 x 5 ml). The organic fractions were dried with anhydrous sodium sulfate, filtered, and concentrated *in vacuo*. Purification by flash column chromatography through silica gel yielded the alkenoic alcohol **5**.

4-phenylpent-4-en-1-ol (**5a**)

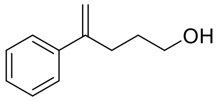

Colorless oil.

^{1}H NMR (500 MHz, $CDCl_3$): δ 7.42 (d, *J* = 7.8 Hz, 2H), 7.34 (t, *J* = 7.2 Hz, 2H), 7.25-7.30 (m, 1H), 5.31 (s, 1H), 5.11 (s, 1H), 3.65 (t, *J* = 6.5 Hz, 2H), 2.61 (t, *J* = 7.5 Hz, 2H), 1.68-1.77 (m, 3H).

^{13}C{^{1}H} NMR (125 MHz, $CDCl_3$): δ 148.02, 141.1, 128.4, 127.5, 126.2, 112.7, 62.4, 31.6, 31.2.

HRMS (ESI-Q-orbitrap) m/z: $[M+Na]^+$ calcd for $C_{11}H_{14}O$ 185.09369, found 185.09375.

The data are in full accordance with the literature (Hornback, J. M.; Proehl, G. S. *J. Am. Chem. Soc.* **1979**, *101* (24), 7367–7373.)

4-(p-tolyl)pent-4-en-1-ol (**5b**)

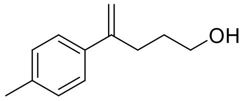

Yellow oil.

^{1}H NMR (500 MHz, $CDCl_3$): δ 7.33 (d, J = 7.5 Hz, 2H), 7.15 (d, J = 7.5 Hz, 2H), 5.29 (s, 1H), 5.07 (s, 1H), 3.66 (t, J = 6.4 Hz, 2H), 2.60 (t, J = 7.5 Hz, 2H), 2.36 (s, 3H), 1.69-1.77 (m, 2H), 1.67 (br. s, 1H).

$^{13}C\{^{1}H\}$ NMR (125 MHz, $CDCl_3$): δ 147.8, 138.1, 137.3, 129.1, 126.1, 111.9, 62.5, 31.6, 31.2, 21.2.

HRMS (ESI-Q-orbitrap) m/z: $[M+Na]^+$ calcd for $C_{12}H_{16}O$ 199.10934, found 199.10952.

The data are in full accordance with the literature (Rösner, C.; Hennecke, U. *Org. Lett.* **2015**, *17* (13), 3226–3229.).

4-(4-methoxyphenyl)pent-4-en-1-ol (**5c**)

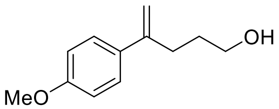

Yellow oil.

^{1}H NMR (500 MHz, $CDCl_3$): δ 7.36 (d, J = 8.7 Hz, 2H), 6.86 (d, J = 8.7 Hz, 2H), 5.23 (s, 1H), 5.01 (s, 1H), 3.81 (s, 3H), 3.65 (t, J = 6.4 Hz, 2H), 2.57 (t, J = 7.5 Hz, 2H), 1.639-1.75 (m, 2H), 1.59 (br. s, 1H).

$^{13}C\{^{1}H\}$ NMR (125 MHz, $CDCl_3$): δ 159.1, 147.29, 133.4, 127.3, 113.8, 111.1, 62.5, 55.4, 31.7, 31.3.

HRMS (ESI-Q-orbitrap) m/z: $[M+H]^+$ calcd for $C_{12}H_{16}O_2$ 193.12231, found 193.12252.

The data are in full accordance with the literature (Rösner, C.; Hennecke, U. *Org. Lett.* **2015**, *17* (13), 3226–3229.).

4-(4-chlorophenyl)pent-4-en-1-ol (**5d**)

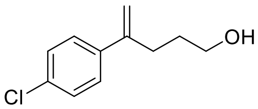

Colorless oil.

^{1}H NMR (500 MHz, $CDCl_3$): δ 7.34 (d, J = 7.7 Hz, 2H), 7.29 (d, J = 7.7 Hz, 2H), 5.28 (s, 1H), 5.11 (s, 1H), 3.66 (t, J = 6.3 Hz, 2H), 2.58 (t, J = 7.5 Hz, 2H), 1.67-1.74 (m, 2H), 1.41 (br. s, 1H).

$^{13}C\{^{1}H\}$ NMR (125 MHz, $CDCl_3$): δ 146.9, 139.5, 133.3, 128.6, 127.5, 113.2, 62.4, 31.6, 31.1.

HRMS (ESI-Q-orbitrap) m/z: $[M+H]^+$ calcd for $C_{12}H_{13}ClO$ 197.07277, found 197.07285.

The data are in full accordance with the literature (Tsuji, N.; Kennemur, J. L.; Buyck, T.; Lee, S.; Prévost, S.; Kaib, P. S. J.; Bykov, D.; Farès, C.; List, B. *Science.* **2018**, *359* (6383), 1501–1505.).

4-(4-fluorophenyl)pent-4-en-1-ol (**5e**)

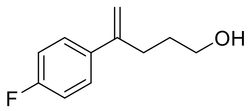

Yellow oil.

^{1}H NMR (500 MHz, $CDCl_3$): δ 7.34-7.39 (m, 2H), 7.00 (t, *J* = 8.0 Hz, 2H), 5.24 (s, 1H), 5.07 (s, 1H), 3.64 (t, *J* = 6.3, 2H), 2.57 (t, *J* = 7.5 Hz, 2H), 1.80 (br. s, 1H), 1.70 (m, 2H).

^{13}C{^{1}H} NMR (125 MHz, $CDCl_3$): δ 163.3, 161.4, 137.1, 137.1, 127.8, 127.7, 115.3, 115.1, 112.6, 62.3, 31.7, 31.1.

HRMS (ESI-Q-orbitrap) m/z: $[M+H]^+$ calcd for $C_{12}H_{13}FO$ 181.10232, found 181.10239.

The data are in full accordance with the literature (Rösner, C.; Hennecke, U. *Org. Lett.* **2015**, *17* (13), 3226–3229.).

4-(4-(trifluoromethyl)phenyl)pent-4-en-1-ol (**5f**)

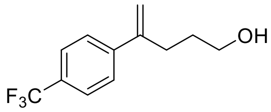

Colorless oil.

^{1}H NMR (500 MHz, $CDCl_3$): δ 7.58 (d, *J* = 8.4 Hz, 2H), 7.50 (d, *J* = 8.4 Hz, 2H), 5.36 (s, 1H), 5.20 (s, 1H), 3.67 (t, *J* = 6.4 Hz, 2H), 2.62 (t, *J* = 7.2 Hz, 2H), 1.68-1.75 (m, 2H), 1.50 (br. s, 1H).

^{13}C{^{1}H} NMR (125 MHz, $CDCl_3$): δ 147.0, 144.8, 129.5 (q, *J* = 32 Hz), 126.5, 125.4 (q, *J* = 4 Hz), 114.6, 62.3, 31.5, 31.1.

HRMS (ESI-Q-orbitrap) m/z: $[M-H]^-$ calcd for $C_{12}H_{13}F_3O$ 229.08457, found 229.08467.

4-(3,5-bis(trifluoromethyl)phenyl)pent-4-en-1-ol (**5g**)

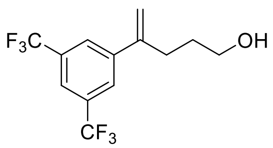

Colorless oil.

^{1}H NMR (500 MHz, $CDCl_3$): δ 7.83 (s, 2H), 7.77 (s, 1H), 5.42 (s, 1H), 5.28 (s, 1H), 3.69 (t, *J* = 6.4 Hz, 2H), 2.63 (t, *J* = 7.6 Hz, 2H), 1.68-1.78 (m, 3H).

$^{13}C\{^{1}H\}$ NMR (125 MHz, $CDCl_3$): δ 145.7, 134.5, 131.8 (q, *J* = 33 Hz), 126.3, 123.5 (q, *J* = 271 Hz), 121.2 (m), 115.8, 62.1, 31.2, 30.9.

HRMS (ESI-Q-orbitrap) m/z: $[M-H]^-$ calcd for $C_{13}H_{12}F_6O$ 297.07196, found 297.07153.

(F) ^{1}H NMR of filter purified products and recovered imide by-products

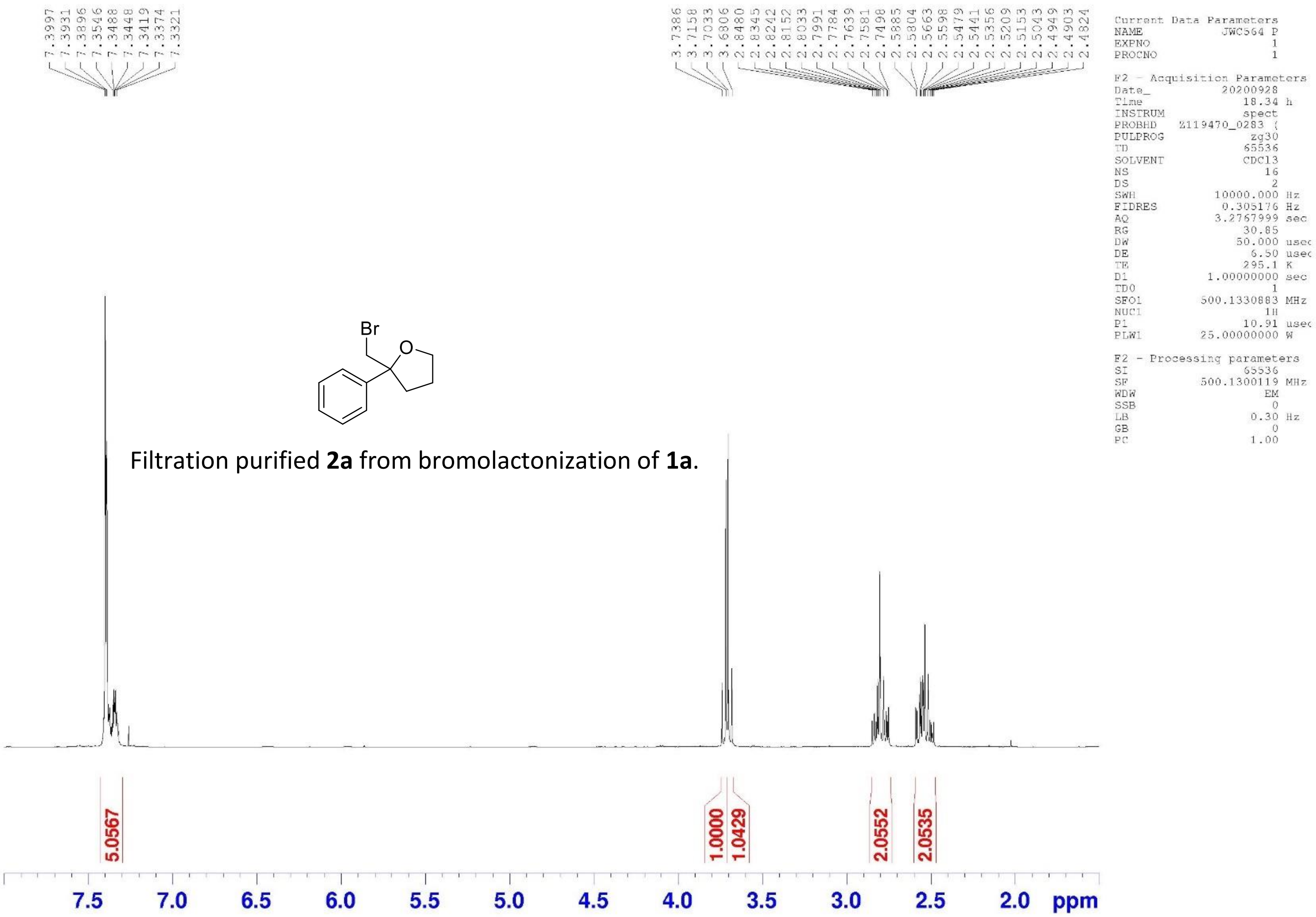

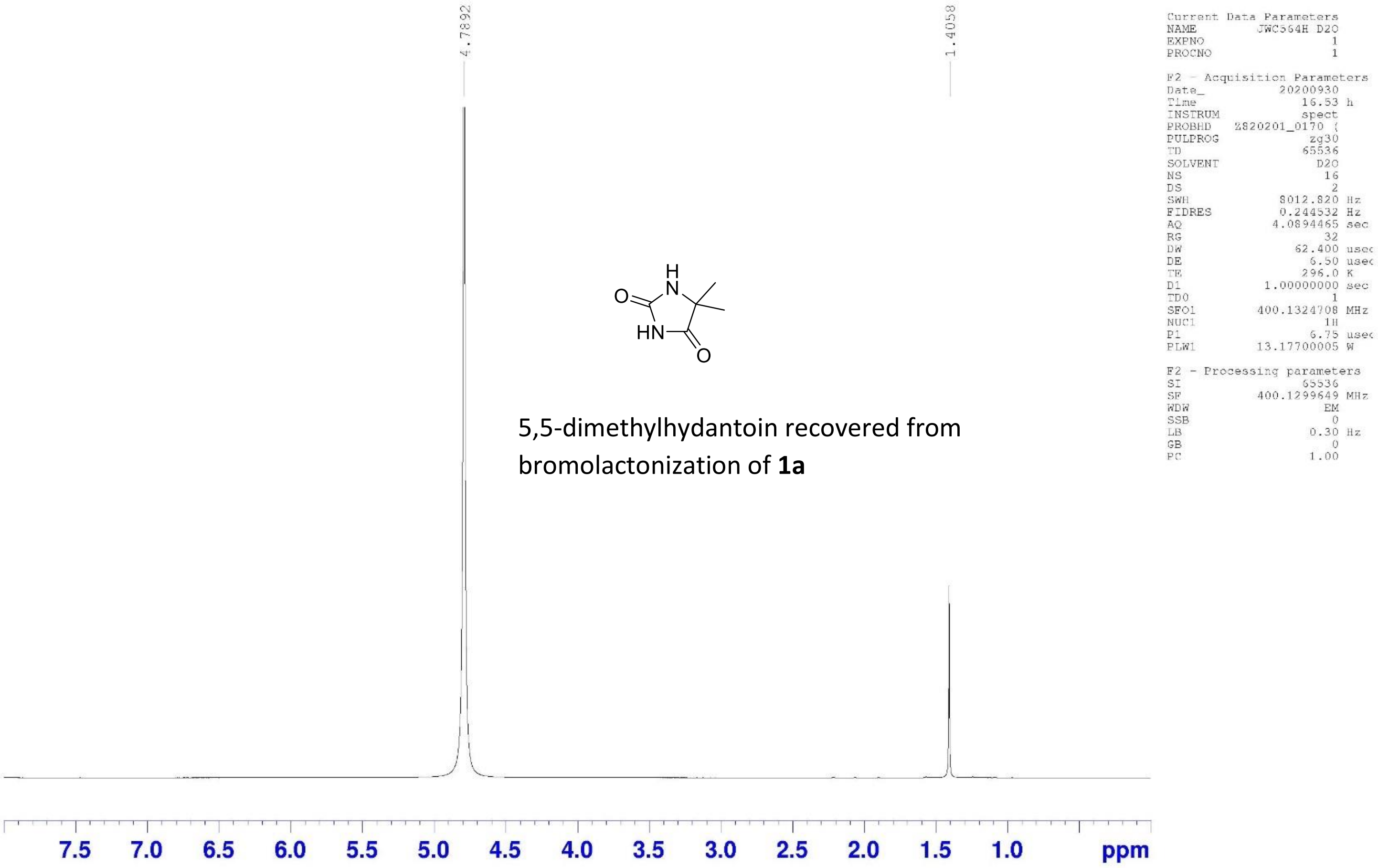

5,5-dimethylhydantoin recovered from bromolactonization of **1a**

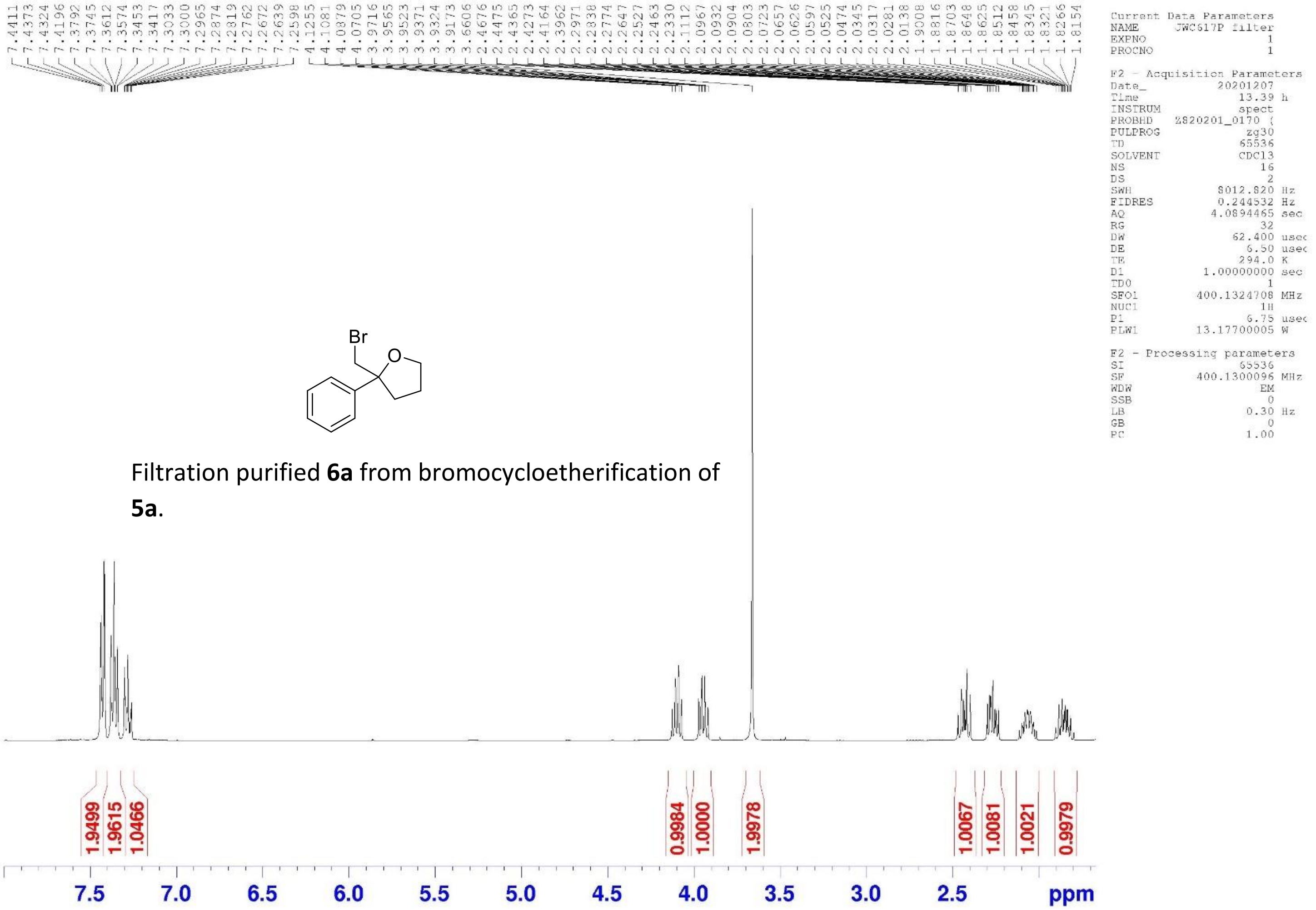

Filtration purified **6a** from bromocycloetherification of **5a**.

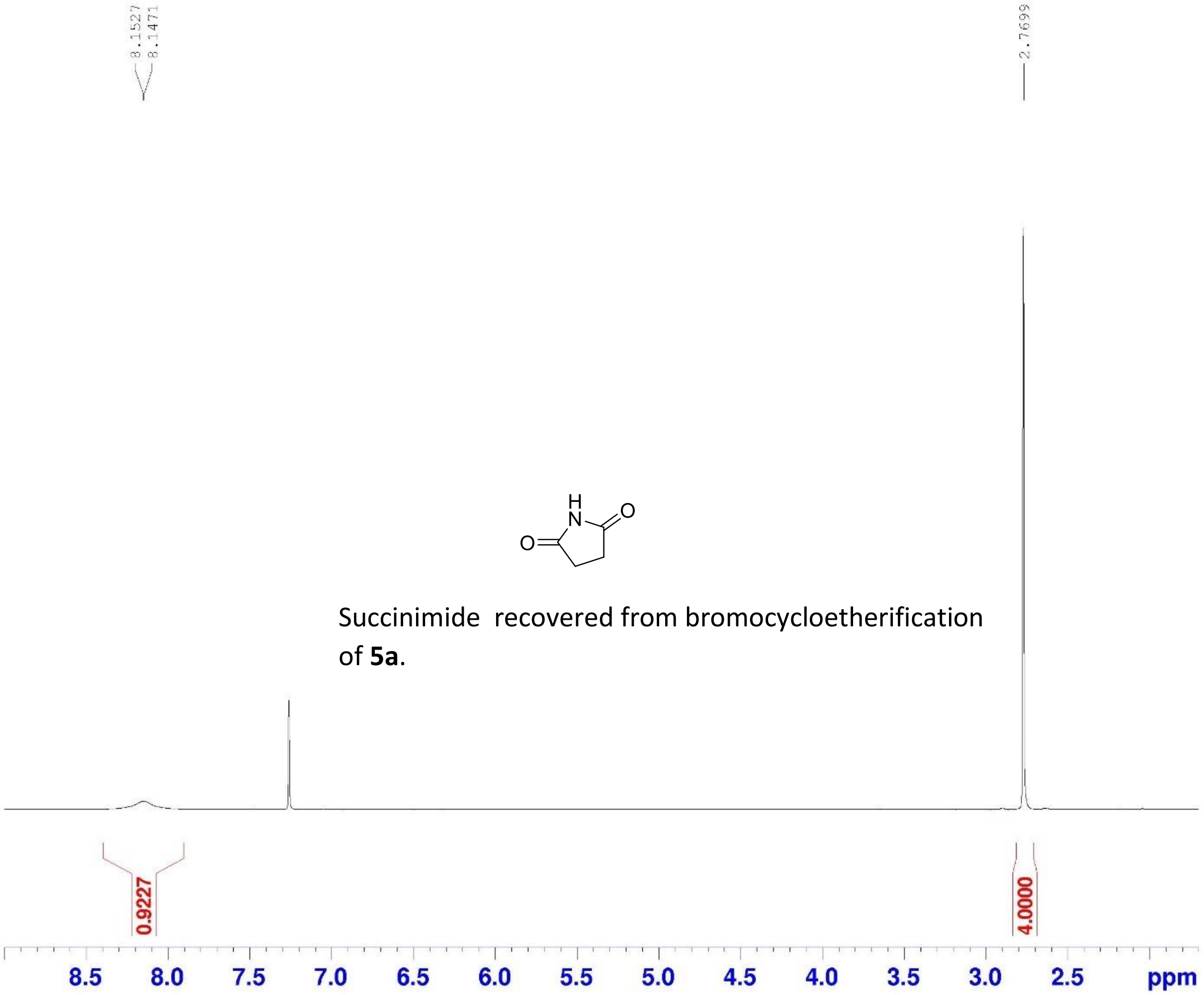

Succinimide recovered from bromocycloetherification of **5a**.

```
Current Data Parameters
NAME            JWC617 Sx
EXPNO                   1
PROCNO                  1

F2 - Acquisition Parameters
Date_           20201214
Time               18.37 h
INSTRUM            spect
PROBHD   Z119470_0283 (
PULPROG             zg30
TD                 65536
SOLVENT            CDCl3
NS                    16
DS                     2
SWH            10000.000 Hz
FIDRES          0.305176 Hz
AQ             3.2767999 sec
RG                 142.5
DW                50.000 usec
DE                  6.50 usec
TE                 295.1 K
D1            1.00000000 sec
TD0                    1
SFO1         500.1330883 MHz
NUC1                  1H
P1                 10.91 usec
PLW1         25.00000000 W

F2 - Processing parameters
SI                 65536
SF           500.1300121 MHz
WDW                   EM
SSB                    0
LB                  0.30 Hz
GB                     0
PC                  1.00
```

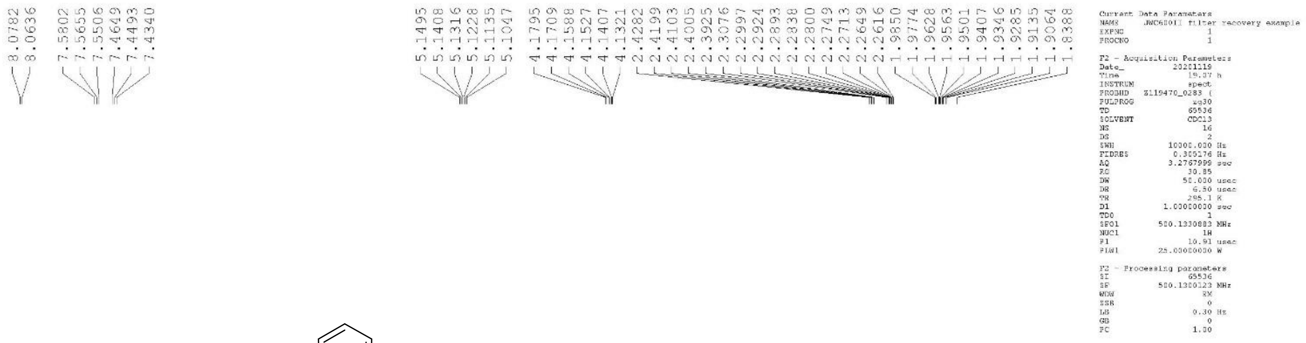

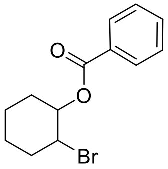

Filtration purified **9ah** from bromoesterification of **7a** and **8h**..

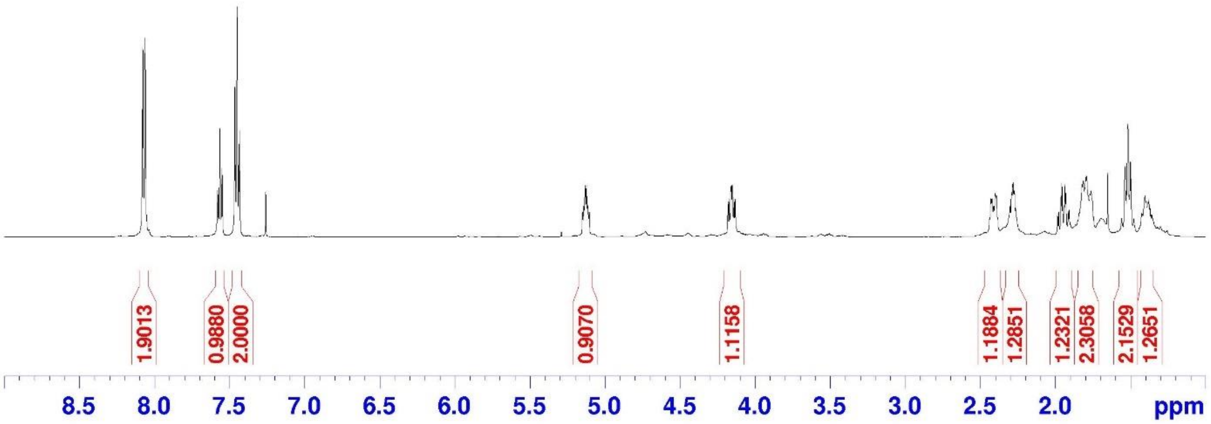

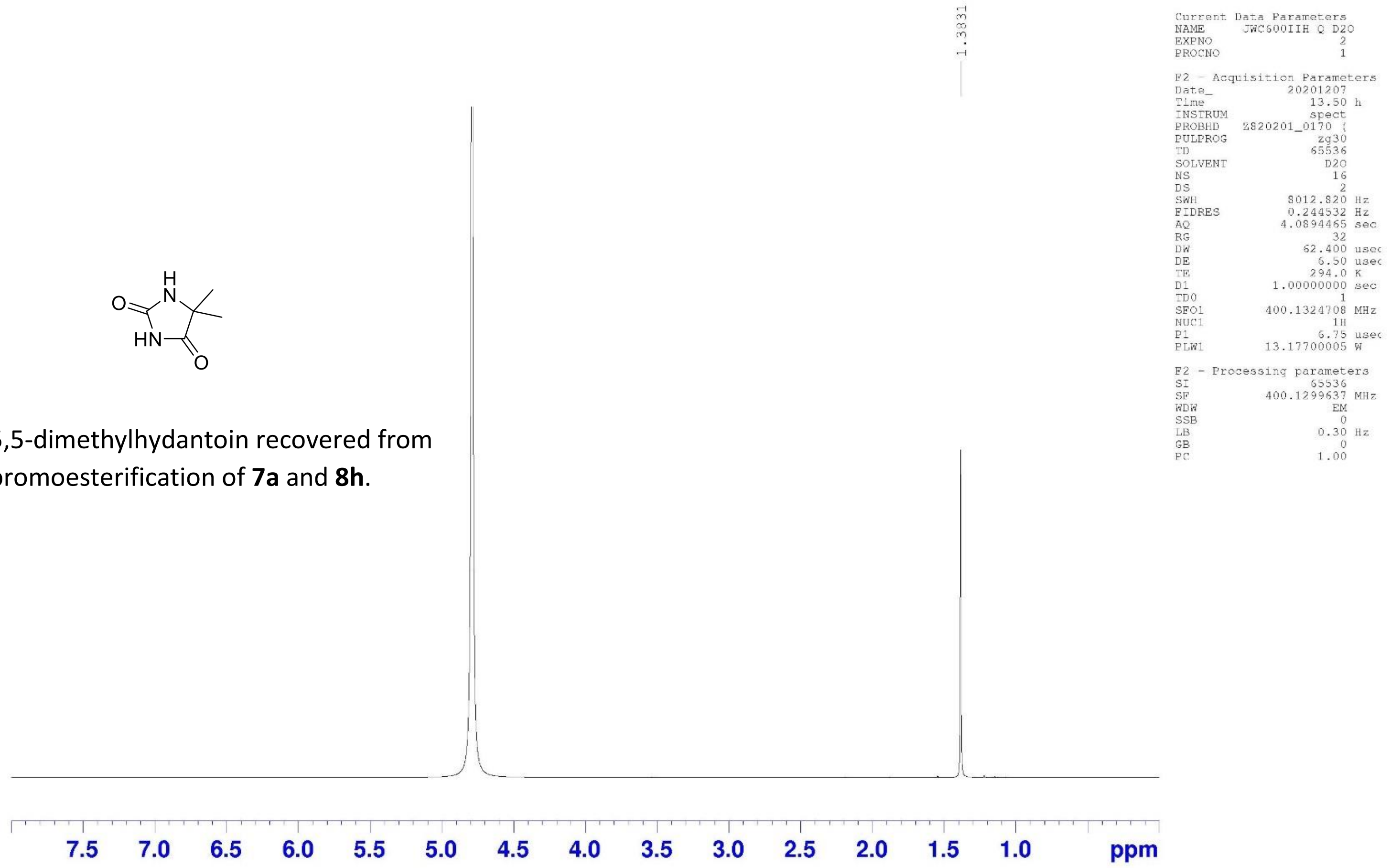

5,5-dimethylhydantoin recovered from bromoesterification of **7a** and **8h**.

(G) ^{1}H and ^{13}C NMR Spectra

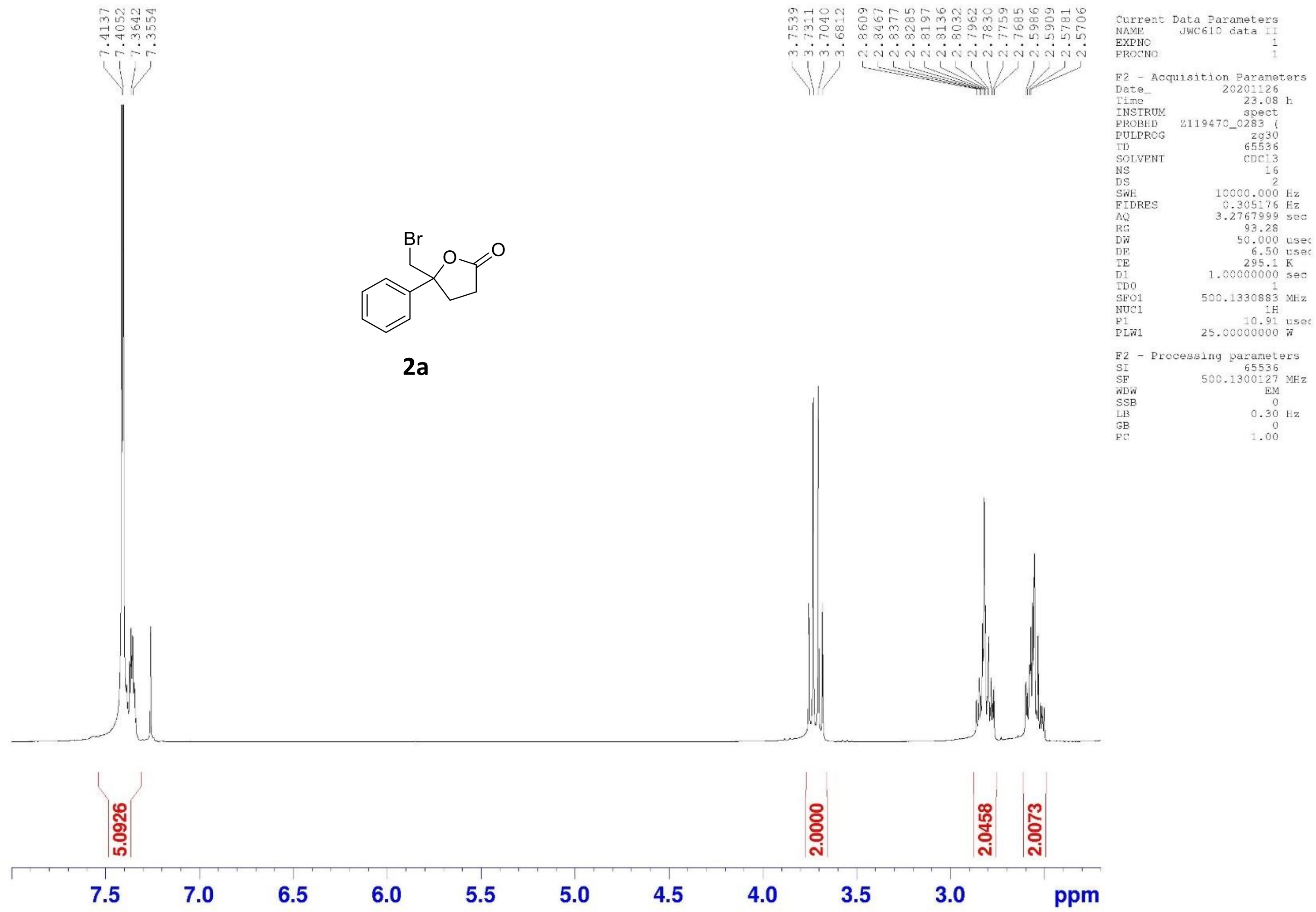

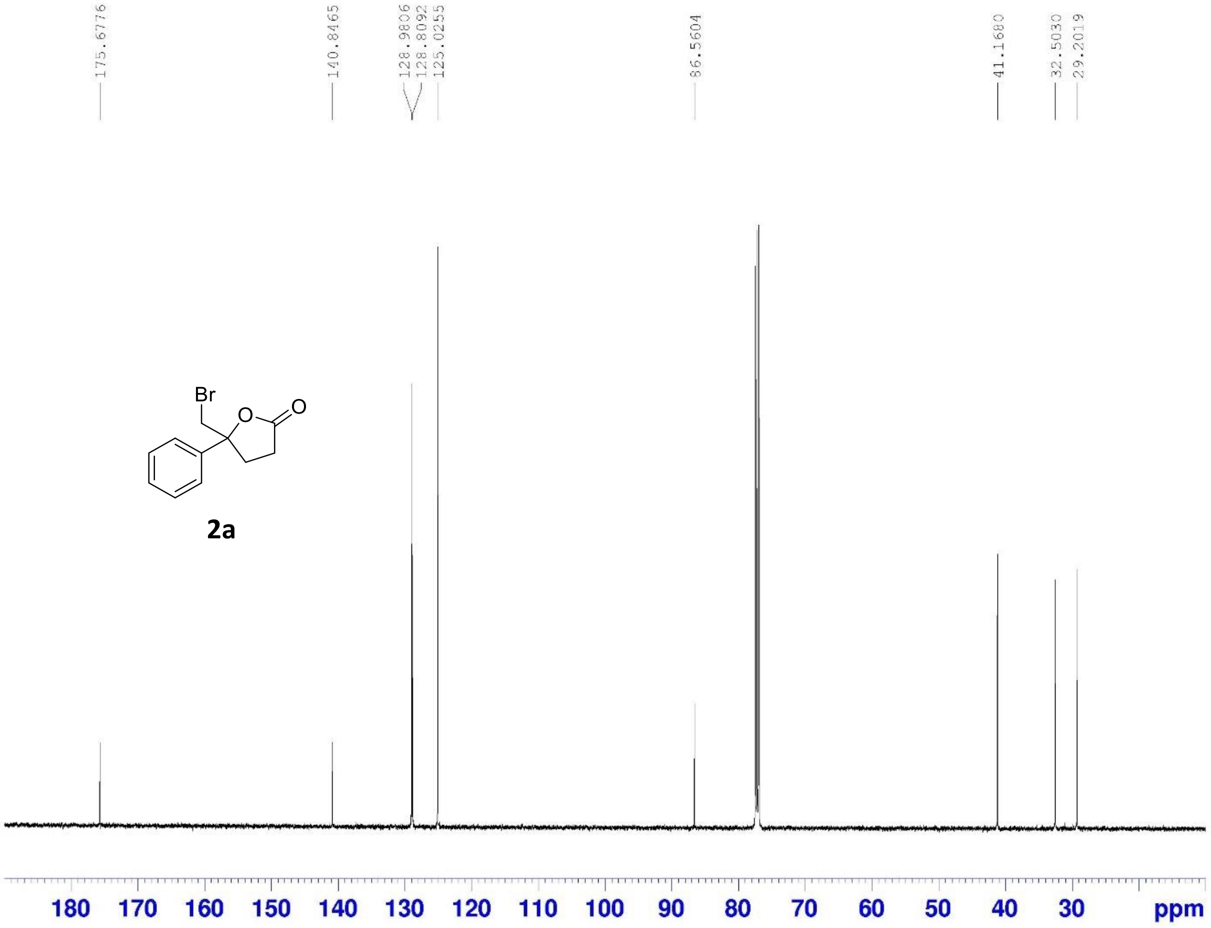

2a

Current Data Parameters
NAME JWC610 data II
EXPNO 2
PROCNO 1

F2 - Acquisition Parameters
Date_ 20201126
Time 23.51 h
INSTRUM spect
PROBHD Z119470_0283 (
PULPROG zgpg30
TD 65536
SOLVENT CDCl3
NS 800
DS 4
SWH 29761.904 Hz
FIDRES 0.908261 Hz
AQ 1.1010048 sec
RG 206.72
DW 16.800 usec
DE 6.50 usec
TE 295.2 K
D1 2.00000000 sec
D11 0.03000000 sec
TD0 1
SFO1 125.7703643 MHz
NUC1 13C
P1 9.75 usec
PLW1 94.00000000 W
SFO2 500.1320005 MHz
NUC2 1H
CPDPRG[2 waltz16
PCPD2 80.00 usec
PLW2 25.00000000 W
PLW12 0.46495000 W
PLW13 0.23387000 W

F2 - Processing parameters
SI 32768
SF 125.7577762 MHz
WDW EM
SSB 0
LB 1.00 Hz
GB 0
PC 1.40

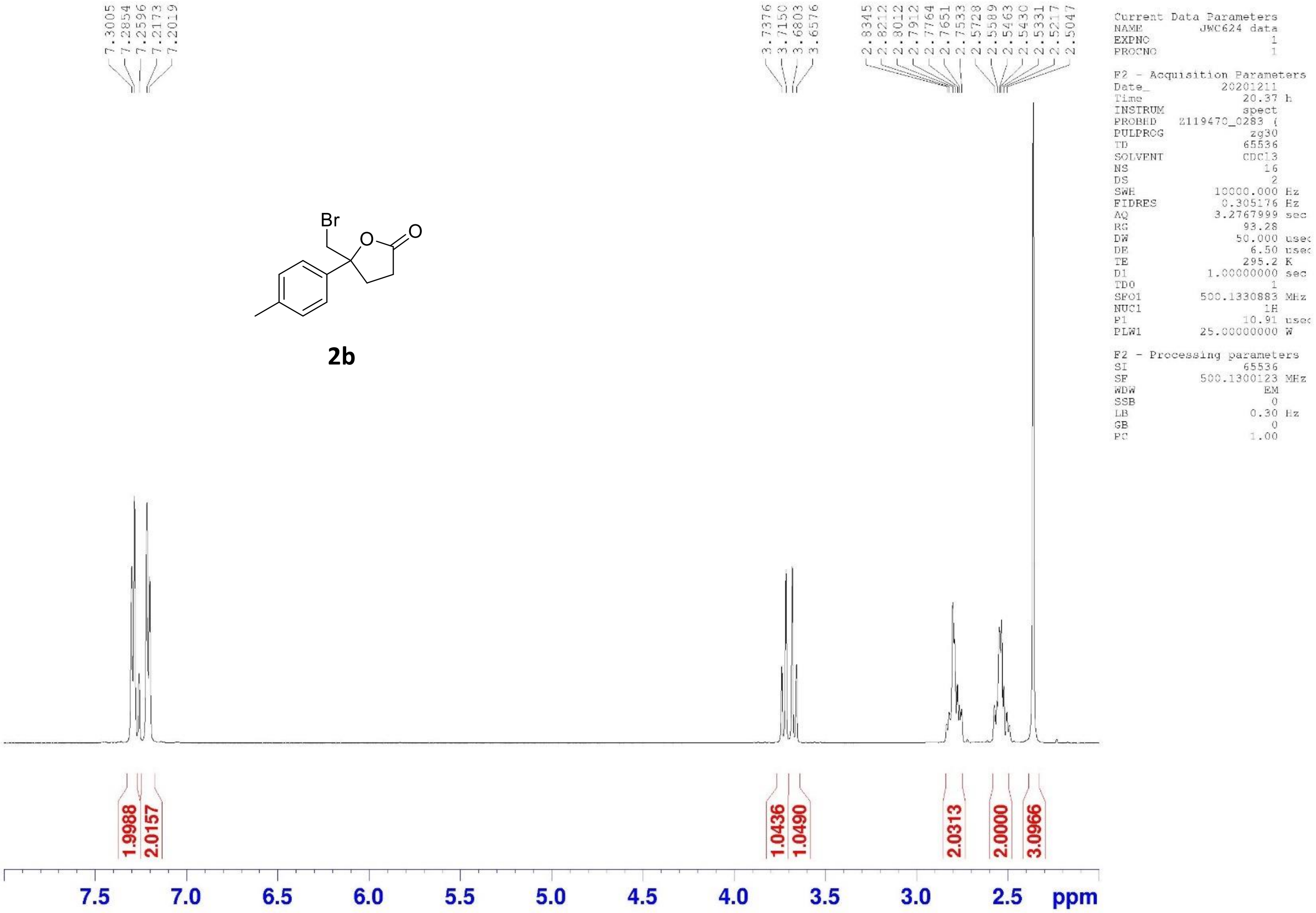

7.3005
7.2854
7.2596
7.2173
7.2019
3.7376
3.7150
3.6803
3.6576
2.8345
2.8212
2.8012
2.7912
2.7764
2.7651
2.7533
2.5728
2.5589
2.5463
2.5430
2.5331
2.5217
2.5047
Br
O
O
2b
1.9988
2.0157
1.0436
1.0490
2.0313
2.0000
3.0966
7.5
7.0
6.5
6.0
5.5
5.0
4.5
4.0
3.5
3.0
2.5
ppm
Current Data Parameters
NAME JWC624 data
EXPNO 1
PROCNO 1
F2 - Acquisition Parameters
Date_ 20201211
Time 20.37 h
INSTRUM spect
PROBHD Z119470_0283 (
PULPROG zg30
TD 65536
SOLVENT CDCl3
NS 16
DS 2
SWH 10000.000 Hz
FIDRES 0.305176 Hz
AQ 3.2767999 sec
RG 93.28
DW 50.000 usec
DE 6.50 usec
TE 295.2 K
D1 1.00000000 sec
TD0 1
SFO1 500.1330883 MHz
NUC1 1H
P1 10.91 usec
PLW1 25.00000000 W
F2 - Processing parameters
SI 65536
SF 500.1300123 MHz
WDW EM
SSB 0
LB 0.30 Hz
GB 0
PC 1.00

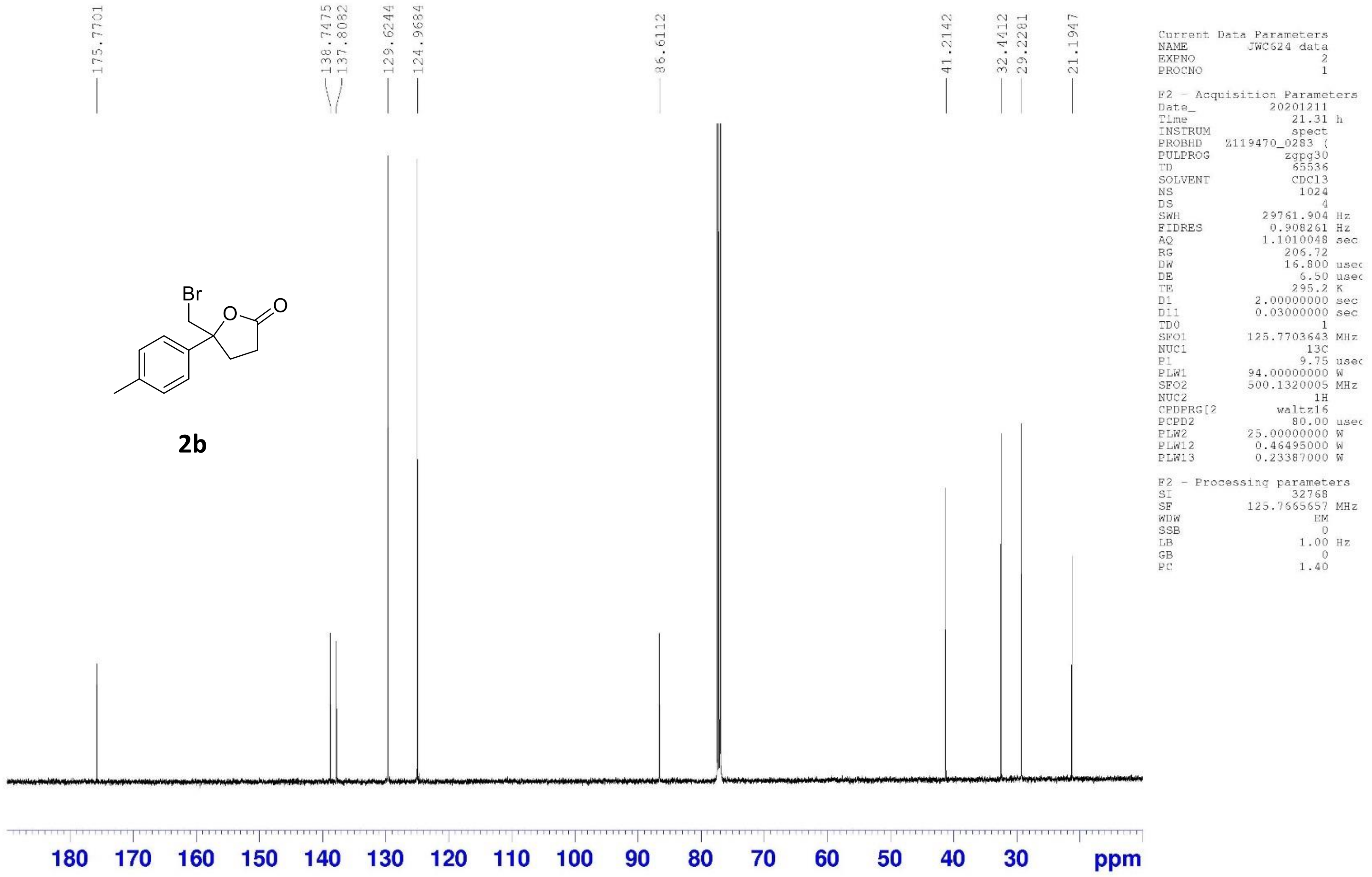
175.7701
138.7475
137.8082
129.6244
124.9684
86.6112
41.2142
32.4412
29.2281
21.1947
180 170 160 150 140 130 120 110 100 90 80 70 60 50 40 30 ppm
Current Data Parameters
NAME JWC624 data
EXPNO 2
PROCNO 1
F2 - Acquisition Parameters
Date_ 20201211
Time 21.31 h
INSTRUM spect
PROBHD Z119470_0283 (
PULPROG zgpg30
TD 65536
SOLVENT CDCl3
NS 1024
DS 4
SWH 29761.904 Hz
FIDRES 0.908261 Hz
AQ 1.1010048 sec
RG 206.72
DW 16.800 usec
DE 6.50 usec
TE 295.2 K
D1 2.00000000 sec
D11 0.03000000 sec
TD0 1
SFO1 125.7703643 MHz
NUC1 13C
P1 9.75 usec
PLW1 94.00000000 W
SFO2 500.1320005 MHz
NUC2 1H
CPDPRG[2 waltz16
PCPD2 80.00 usec
PLW2 25.00000000 W
PLW12 0.46495000 W
PLW13 0.23387000 W
F2 - Processing parameters
SI 32768
SF 125.7665657 MHz
WDW EM
SSB 0
LB 1.00 Hz
GB 0
PC 1.40
Br
O
O

2b

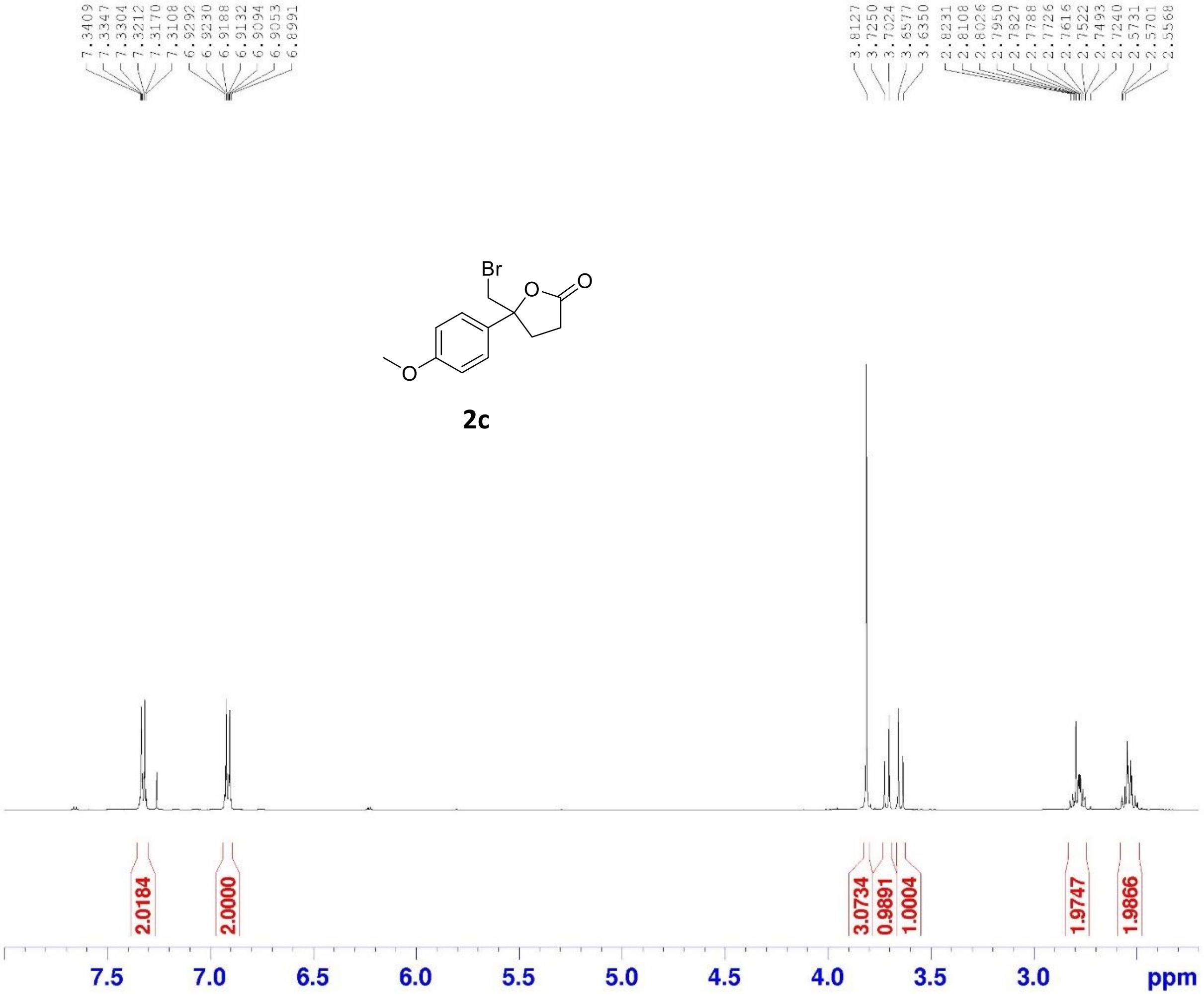

Current Data Parameters
NAME JWC468 data
EXPNO 1
PROCNO 1

F2 - Acquisition Parameters
Date_ 20201116
Time 18.22 h
INSTRUM spect
PROBHD Z119470_0283 (
PULPROG zg30
TD 65536
SOLVENT CDCl3
NS 16
DS 2
SWH 10000.000 Hz
FIDRES 0.305176 Hz
AQ 3.2767999 sec
RG 83.35
DW 50.000 usec
DE 6.50 usec
TE 295.2 K
D1 1.00000000 sec
TD0 1
SFO1 500.1330883 MHz
NUC1 1H
P1 10.91 usec
PLW1 25.00000000 W

F2 - Processing parameters
SI 65536
SF 500.1300125 MHz
WDW EM
SSB 0
LB 0.30 Hz
GB 0
PC 1.00

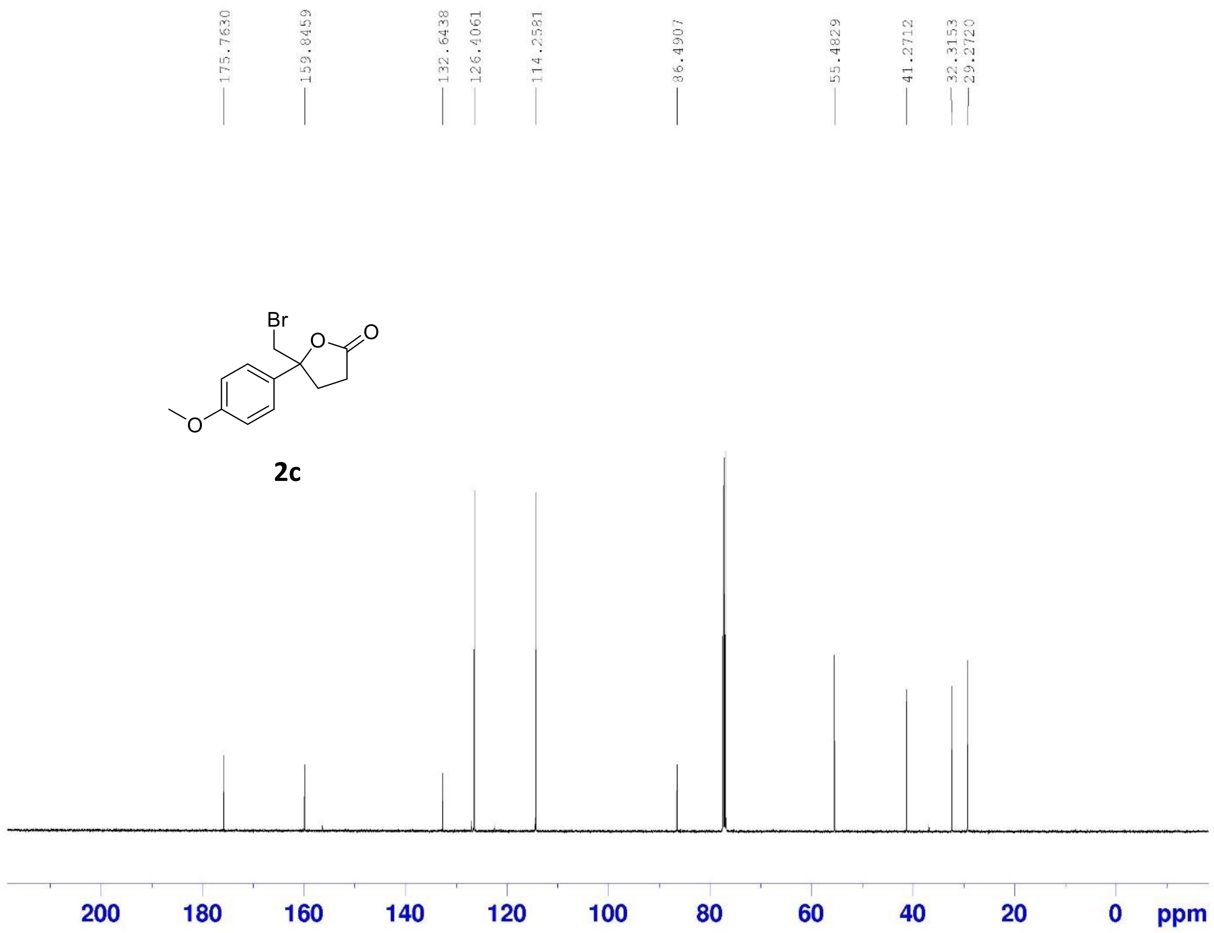

2c

Current Data Parameters		
NAME	JWC468 data	
EXPNO	2	
PROCNO	1	
F2 - Acquisition Parameters		
Date_	20201116	
Time	21.10	h
INSTRUM	spect	
PROBHD	Z119470_0283 (	
PULPROG	zgpg30	
TD	65536	
SOLVENT	CDCl3	
NS	700	
DS	4	
SWH	29761.904	Hz
FIDRES	0.908261	Hz
AQ	1.1010048	sec
RG	206.72	
DW	16.800	usec
DE	6.50	usec
TE	295.2	K
D1	2.00000000	sec
D11	0.03000000	sec
TD0	1	
SFO1	125.7703643	MHz
NUC1	13C	
P1	9.75	usec
PLW1	94.00000000	W
SFO2	500.1320005	MHz
NUC2	1H	
CPDPRG[2	waltz16	
PCPD2	80.00	usec
PLW2	25.00000000	W
PLW12	0.46495000	W
PLW13	0.23387000	W
F2 - Processing parameters		
SI	32768	
SF	125.7577761	MHz
WDW	EM	
SSB	0	
LB	1.00	Hz
GB	0	
PC	1.40	

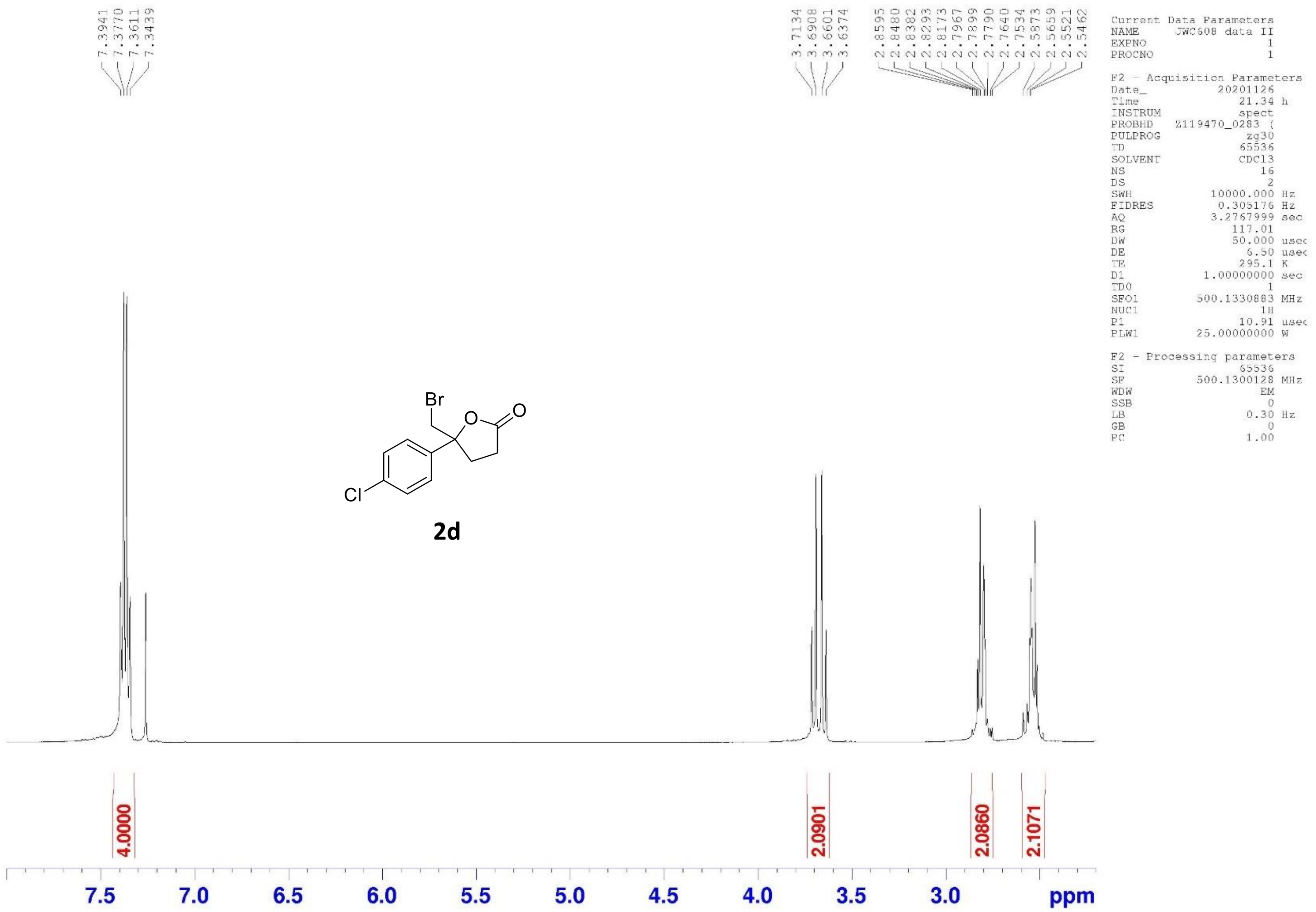
7.3941
7.3770
7.3611
7.3439
3.7134
3.6908
3.6601
3.6374
2.8595
2.8480
2.8382
2.8293
2.8173
2.7967
2.7899
2.7790
2.7640
2.7534
2.5873
2.5659
2.5521
2.5462
Current Data Parameters
NAME JWC608 data II
EXPNO 1
PROCNO 1
F2 - Acquisition Parameters
Date_ 20201126
Time 21.34 h
INSTRUM spect
PROBHD Z119470_0283 (
PULPROG zg30
TD 65536
SOLVENT CDCl3
NS 16
DS 2
SWH 10000.000 Hz
FIDRES 0.305176 Hz
AQ 3.2767999 sec
RG 117.01
DW 50.000 usec
DE 6.50 usec
TE 295.1 K
D1 1.00000000 sec
TD0 1
SFO1 500.1330883 MHz
NUC1 1H
P1 10.91 usec
PLW1 25.00000000 W
F2 - Processing parameters
SI 65536
SF 500.1300128 MHz
WDW EM
SSB 0
LB 0.30 Hz
GB 0
PC 1.00
Br
O
O
Cl
2d
4.0000
2.0901
2.0860
2.1071
7.5
7.0
6.5
6.0
5.5
5.0
4.5
4.0
3.5
3.0
ppm

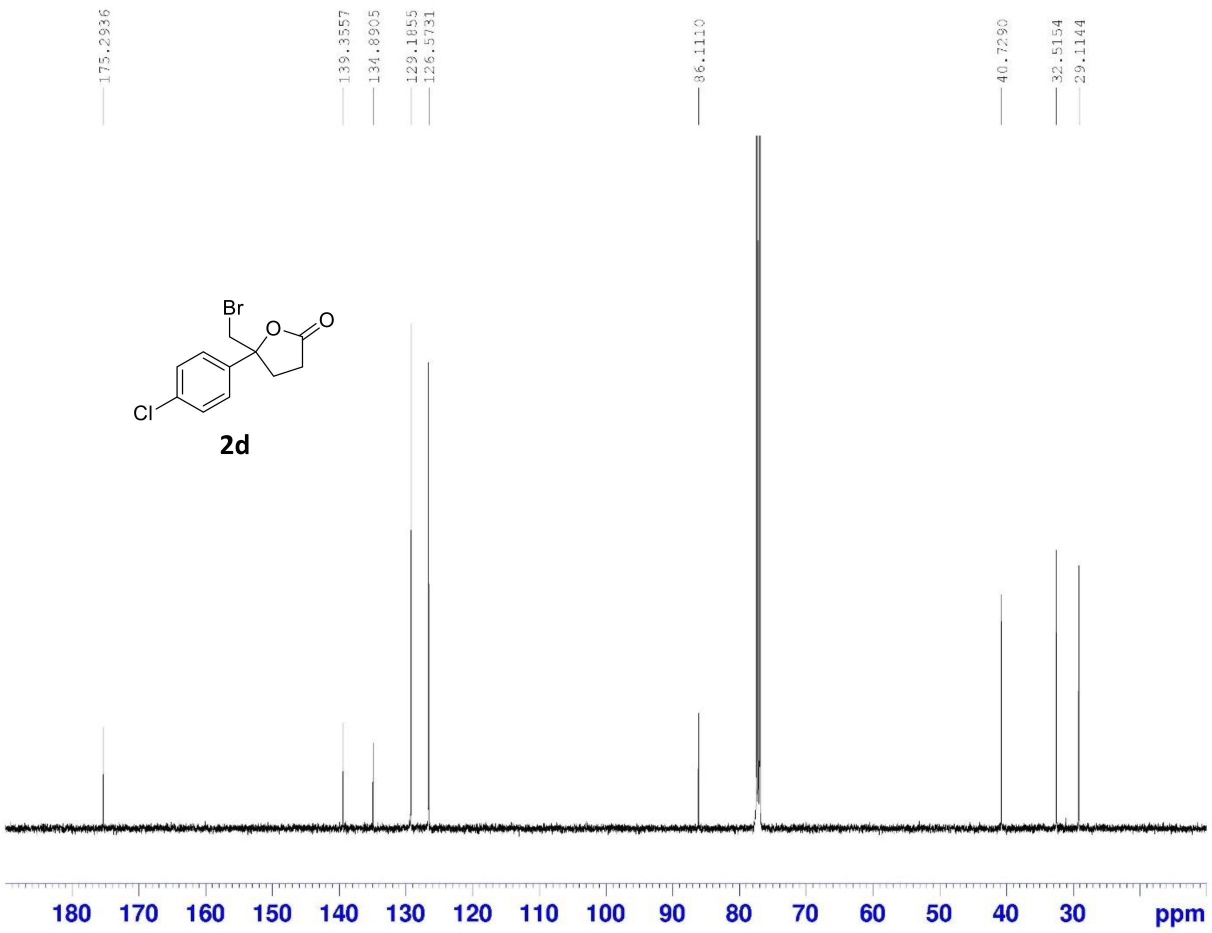

Current Data Parameters

NAME	JWC608 data II	
EXPNO	2	
PROCNO	1	

F2 - Acquisition Parameters

Date_	20201126	
Time	22.17	h
INSTRUM	spect	
PROBHD	Z119470_0283 (	
PULPROG	zgpg30	
TD	65536	
SOLVENT	CDCl3	
NS	800	
DS	4	
SWH	29761.904	Hz
FIDRES	0.908261	Hz
AQ	1.1010048	sec
RG	206.72	
DW	16.800	usec
DE	6.50	usec
TE	295.2	K
D1	2.00000000	sec
D11	0.03000000	sec
TD0	1	
SFO1	125.7703643	MHz
NUC1	13C	
P1	9.75	usec
PLW1	94.00000000	W
SFO2	500.1320005	MHz
NUC2	1H	
CPDPRG[2	waltz16	
PCPD2	80.00	usec
PLW2	25.00000000	W
PLW12	0.46495000	W
PLW13	0.23387000	W

F2 - Processing parameters

SI	32768	
SF	125.7577744	MHz
WDW	EM	
SSB	0	
LB	1.00	Hz
GB	0	
PC	1.40	

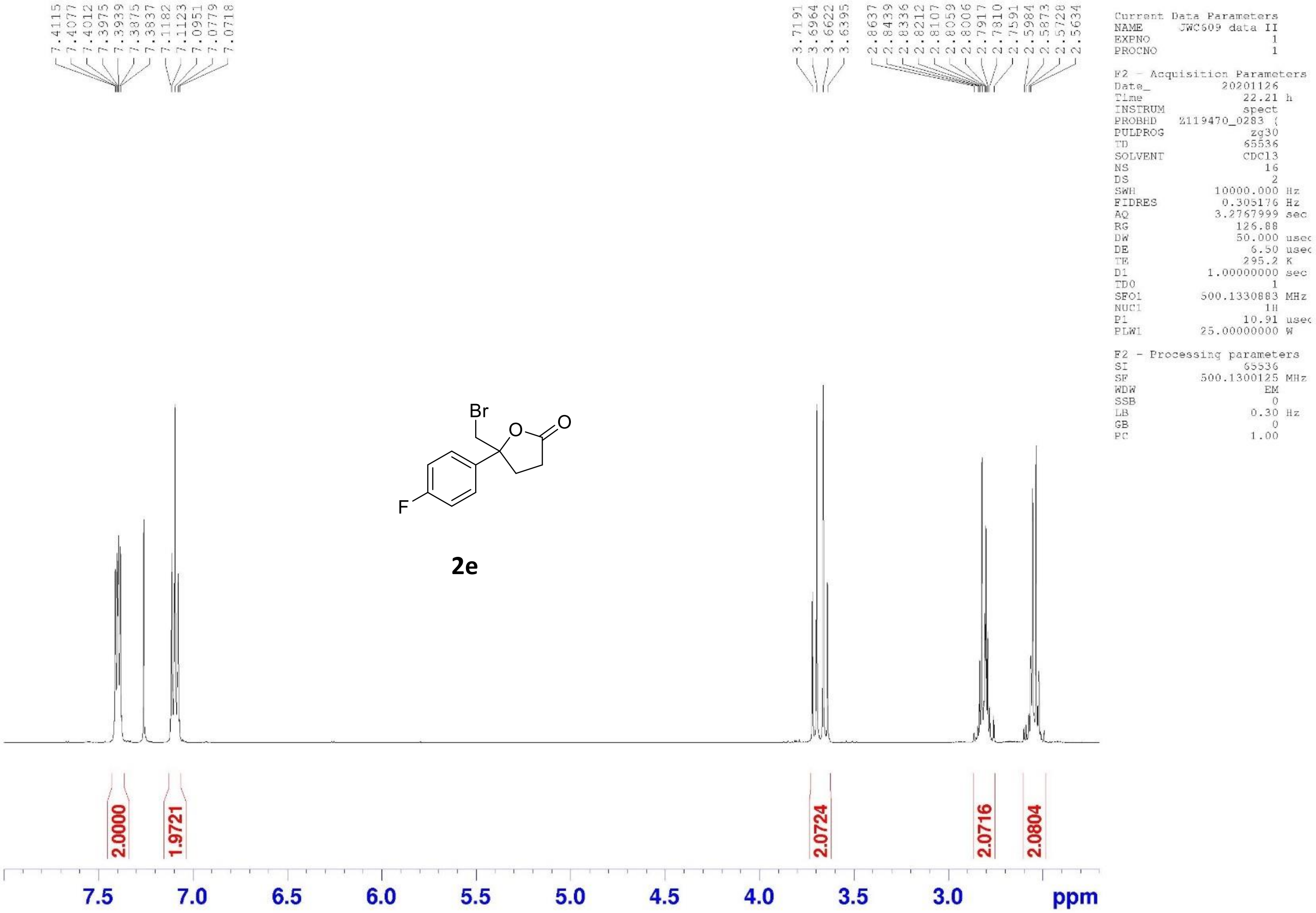
7.4115
7.4077
7.4012
7.3975
7.3939
7.3875
7.3837
7.1182
7.1123
7.0951
7.0779
7.0718
3.7191
3.6964
3.6622
3.6395
2.8637
2.8439
2.8336
2.8212
2.8107
2.8059
2.8006
2.7917
2.7810
2.7591
2.5984
2.5873
2.5728
2.5634
Current Data Parameters
NAME JWC609 data II
EXPNO 1
PROCNO 1
F2 - Acquisition Parameters
Date_ 20201126
Time 22.21 h
INSTRUM spect
PROBHD Z119470_0283 (
PULPROG zg30
TD 65536
SOLVENT CDCl3
NS 16
DS 2
SWH 10000.000 Hz
FIDRES 0.305176 Hz
AQ 3.2767999 sec
RG 126.88
DW 50.000 usec
DE 6.50 usec
TE 295.2 K
D1 1.00000000 sec
TD0 1
SFO1 500.1330883 MHz
NUC1 1H
P1 10.91 usec
PLW1 25.00000000 W
F2 - Processing parameters
SI 65536
SF 500.1300125 MHz
WDW EM
SSB 0
LB 0.30 Hz
GB 0
PC 1.00
Br
O
O
F
2e
2.0000
1.9721
2.0724
2.0716
2.0804
7.5
7.0
6.5
6.0
5.5
5.0
4.5
4.0
3.5
3.0
ppm

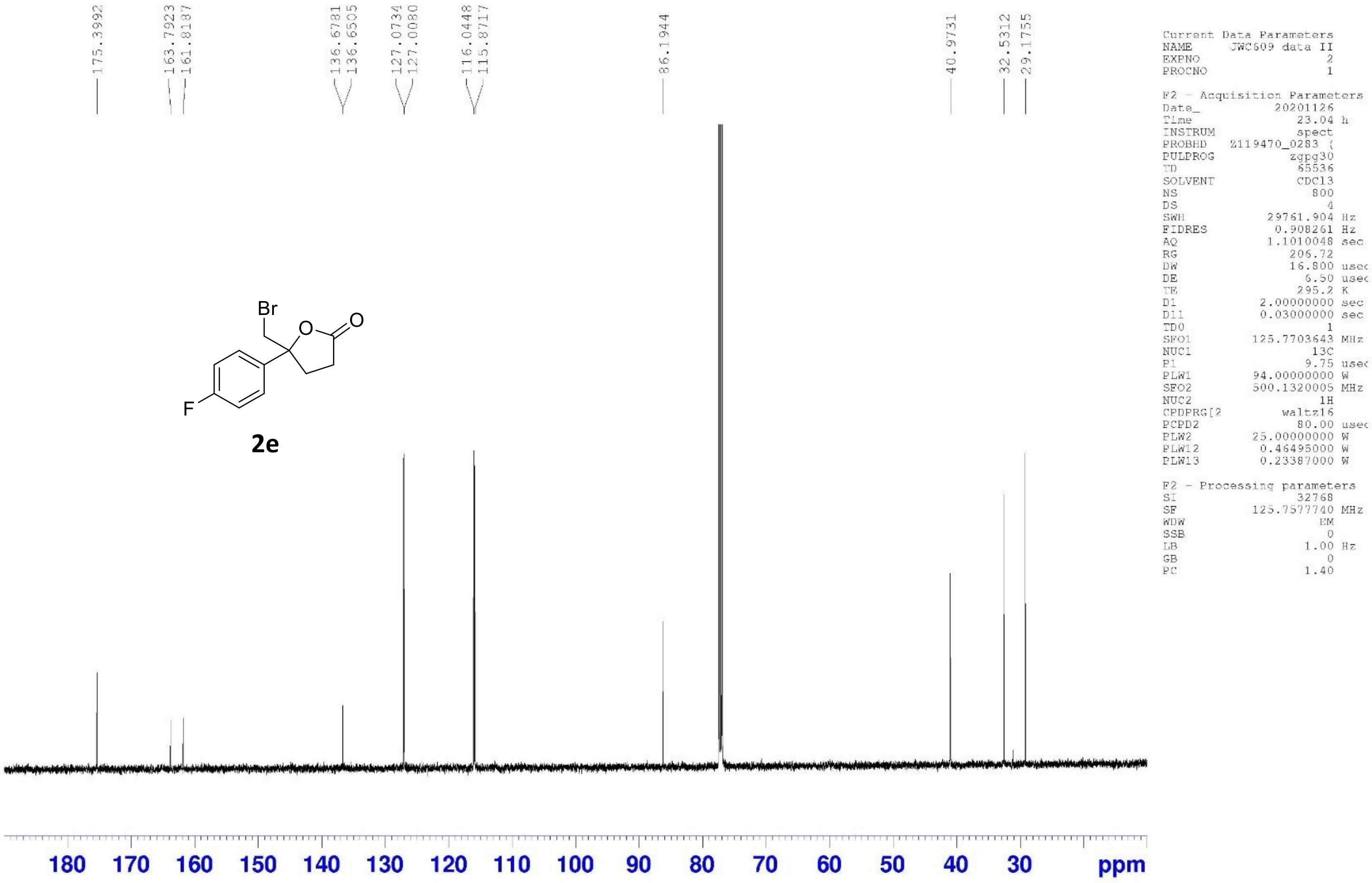
175.3992
163.7923
161.8187
136.6781
136.6505
127.0734
127.0080
116.0448
115.8717
86.1944
40.9731
32.5312
29.1755
Br
O
O
F
2e
180 170 160 150 140 130 120 110 100 90 80 70 60 50 40 30 ppm
Current Data Parameters
NAME JWC609 data II
EXPNO 2
PROCNO 1
F2 - Acquisition Parameters
Date_ 20201126
Time 23.04 h
INSTRUM spect
PROBHD Z119470_0283 (
PULPROG zgpg30
TD 65536
SOLVENT CDCl3
NS 800
DS 4
SWH 29761.904 Hz
FIDRES 0.908261 Hz
AQ 1.1010048 sec
RG 206.72
DW 16.800 usec
DE 6.50 usec
TE 295.2 K
D1 2.00000000 sec
D11 0.03000000 sec
TD0 1
SFO1 125.7703643 MHz
NUC1 13C
P1 9.75 usec
PLW1 94.00000000 W
SFO2 500.1320005 MHz
NUC2 1H
CPDPRG[2 waltz16
PCPD2 80.00 usec
PLW2 25.00000000 W
PLW12 0.46495000 W
PLW13 0.23387000 W
F2 - Processing parameters
SI 32768
SF 125.7577740 MHz
WDW EM
SSB 0
LB 1.00 Hz
GB 0
PC 1.40

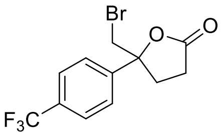

2f

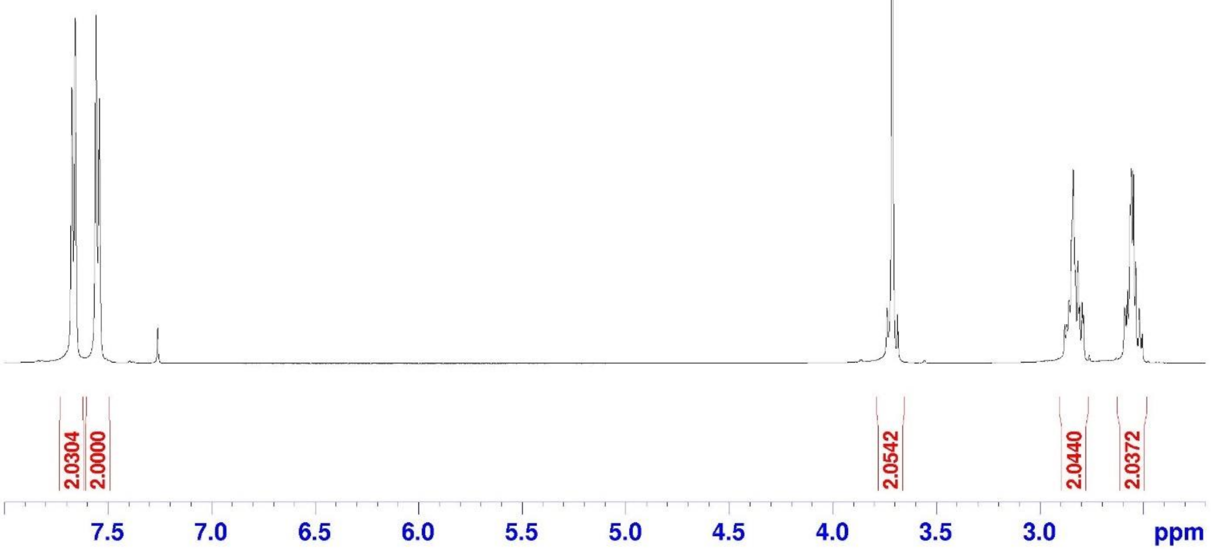
7.6745
7.6581
7.5580
7.5417
3.7372
3.7141
3.7093
3.6863
2.8790
2.8691
2.8583
2.8473
2.8386
2.8281
2.8155
2.8082
2.7955
2.7889
2.5902
2.5858
2.5762
2.5576
Current Data Parameters
NAME JWC472 data
EXPNO 2
PROCNO 1
F2 - Acquisition Parameters
Date_ 20201116
Time 18.27 h
INSTRUM spect
PROBHD Z119470_0283 (
PULPROG zg30
TD 65536
SOLVENT CDCl3
NS 16
DS 2
SWH 10000.000 Hz
FIDRES 0.305176 Hz
AQ 3.2767999 sec
RG 30.85
DW 50.000 usec
DE 6.50 usec
TE 295.2 K
D1 1.00000000 sec
TD0 1
SFO1 500.1330883 MHz
NUC1 1H
P1 10.91 usec
PLW1 25.00000000 W
F2 - Processing parameters
SI 65536
SF 500.1300128 MHz
WDW EM
SSB 0
LB 0.30 Hz
GB 0
PC 1.00
2.0304
2.0000
2.0542
2.0440
2.0372
7.5
7.0
6.5
6.0
5.5
5.0
4.5
4.0
3.5
3.0
ppm

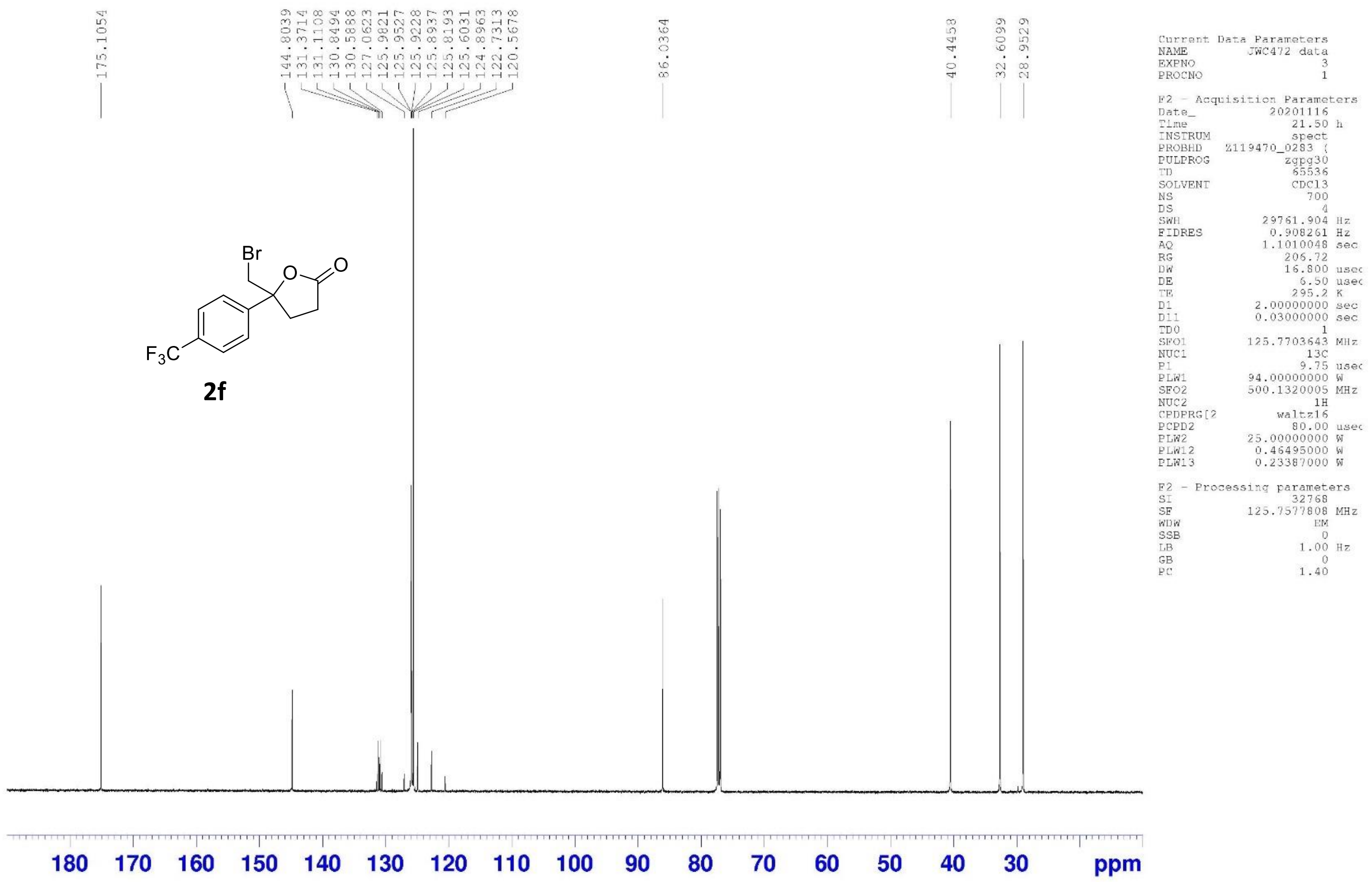
175.1054
144.8039
131.3714
131.1108
130.8494
130.5888
127.0623
125.9821
125.9527
125.9228
125.8937
125.8193
125.6031
124.8963
122.7313
120.5678
86.0364
40.4453
32.6099
28.9529
Br
O
O
F3C
2f
Current Data Parameters
NAME JWC472 data
EXPNO 3
PROCNO 1
F2 - Acquisition Parameters
Date_ 20201116
Time 21.50 h
INSTRUM spect
PROBHD Z119470_0283 (
PULPROG zgpg30
TD 65536
SOLVENT CDCl3
NS 700
DS 4
SWH 29761.904 Hz
FIDRES 0.908261 Hz
AQ 1.1010048 sec
RG 206.72
DW 16.800 usec
DE 6.50 usec
TE 295.2 K
D1 2.00000000 sec
D11 0.03000000 sec
TD0 1
SFO1 125.7703643 MHz
NUC1 13C
P1 9.75 usec
PLW1 94.00000000 W
SFO2 500.1320005 MHz
NUC2 1H
CPDPRG[2 waltz16
PCPD2 80.00 usec
PLW2 25.00000000 W
PLW12 0.46495000 W
PLW13 0.23387000 W
F2 - Processing parameters
SI 32768
SF 125.7577808 MHz
WDW EM
SSB 0
LB 1.00 Hz
GB 0
PC 1.40
180 170 160 150 140 130 120 110 100 90 80 70 60 50 40 30 ppm

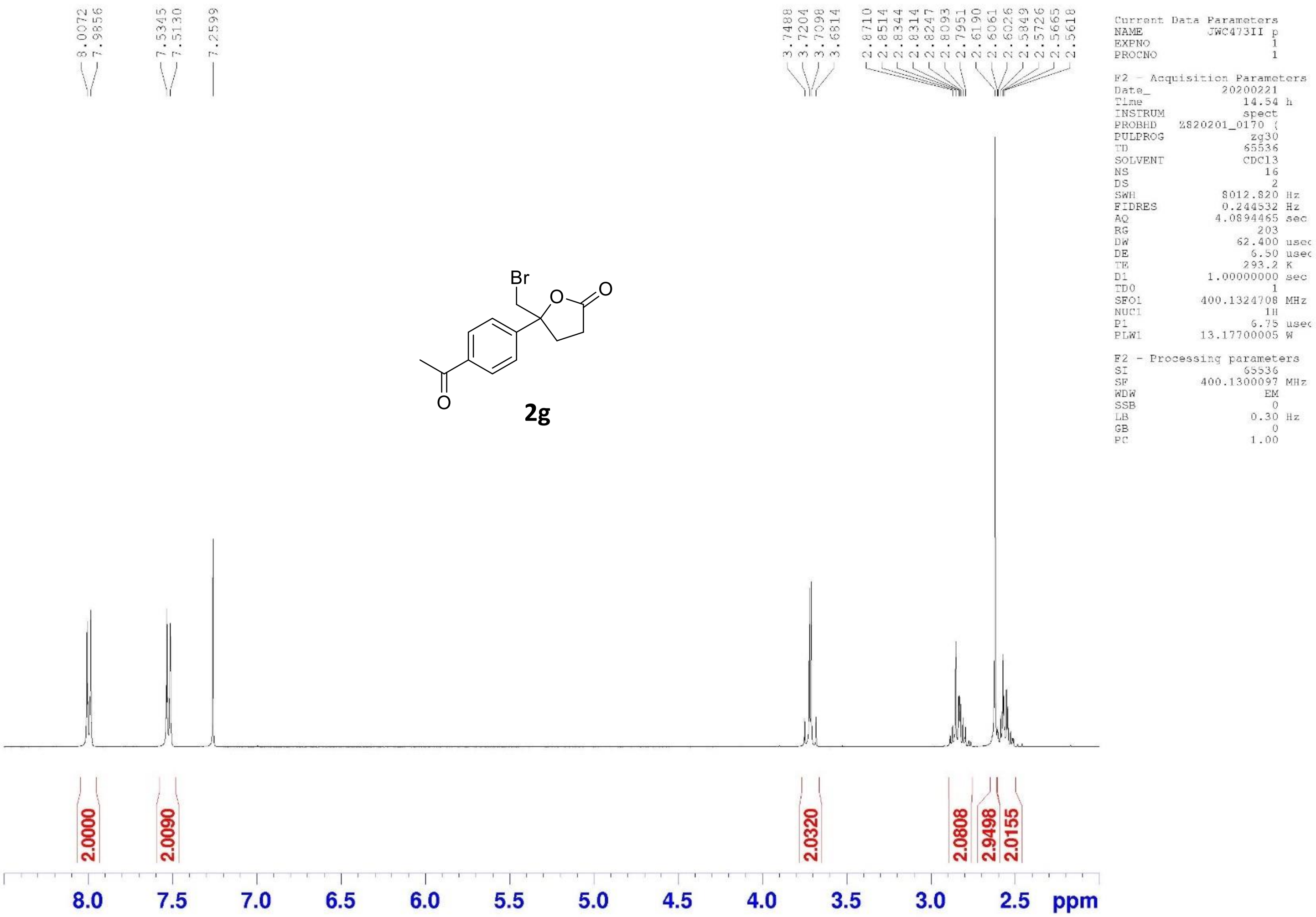

8.0072
7.9856
7.5345
7.5130
7.2599
3.7488
3.7204
3.7098
3.6814
2.8710
2.8514
2.8344
2.8314
2.8247
2.8093
2.7951
2.6190
2.6061
2.6026
2.5849
2.5726
2.5665
2.5618
Br
O
O
O
2g
Current Data Parameters
NAME JWC473II p
EXPNO 1
PROCNO 1
F2 - Acquisition Parameters
Date_ 20200221
Time 14.54 h
INSTRUM spect
PROBHD Z820201_0170 (
PULPROG zg30
TD 65536
SOLVENT CDCl3
NS 16
DS 2
SWH 8012.820 Hz
FIDRES 0.244532 Hz
AQ 4.0894465 sec
RG 203
DW 62.400 usec
DE 6.50 usec
TE 293.2 K
D1 1.00000000 sec
TD0 1
SFO1 400.1324708 MHz
NUC1 1H
P1 6.75 usec
PLW1 13.17700005 W
F2 - Processing parameters
SI 65536
SF 400.1300097 MHz
WDW EM
SSB 0
LB 0.30 Hz
GB 0
PC 1.00
2.0000
2.0090
2.0320
2.0808
2.9498
2.0155
8.0
7.5
7.0
6.5
6.0
5.5
5.0
4.5
4.0
3.5
3.0
2.5
ppm

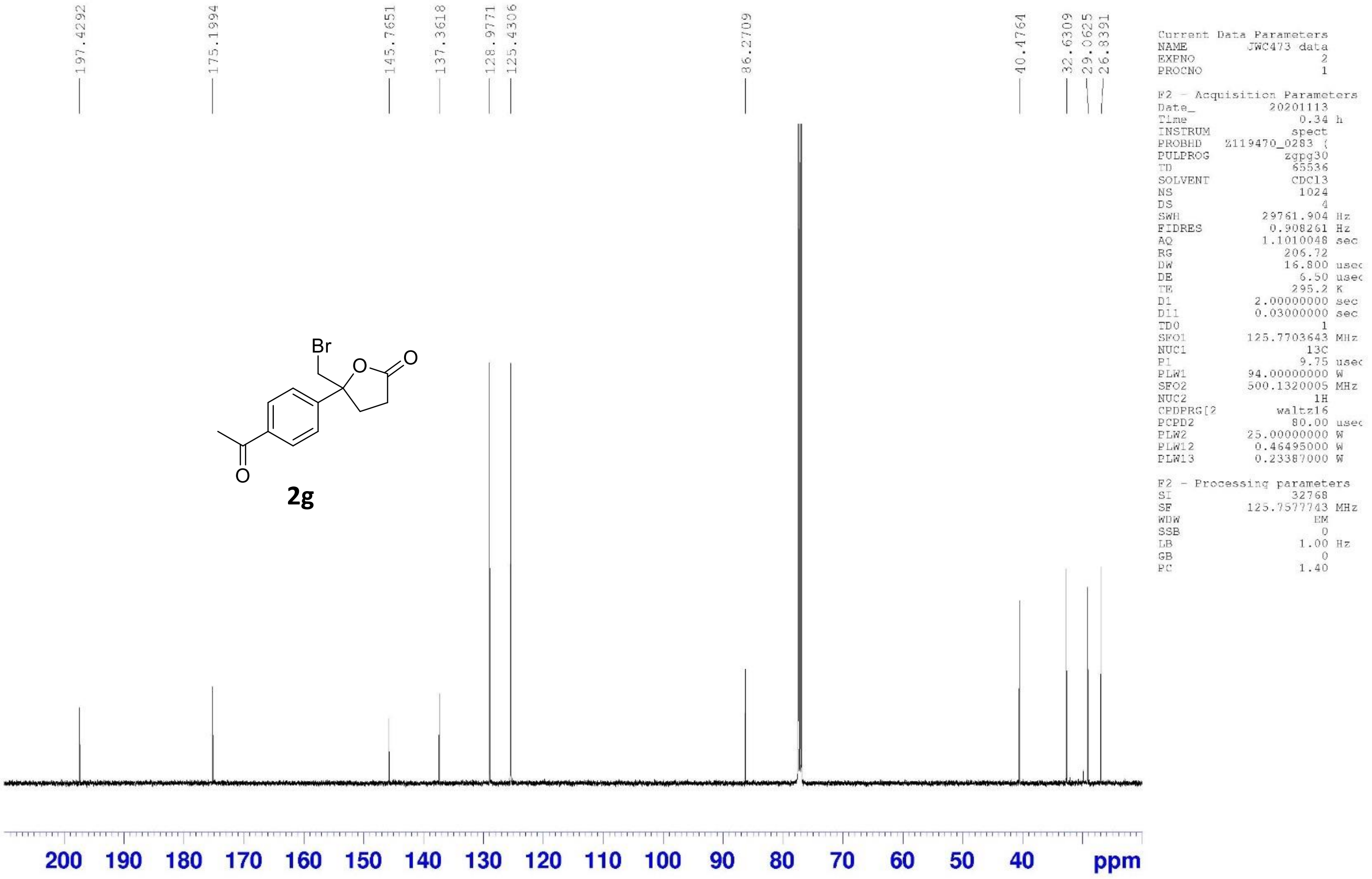
197.4292
175.1994
145.7651
137.3618
128.9771
125.4306
86.2709
40.4764
32.6309
29.0625
26.8391
Br
O
O
O
2g
200 190 180 170 160 150 140 130 120 110 100 90 80 70 60 50 40 ppm
Current Data Parameters
NAME JWC473 data
EXPNO 2
PROCNO 1
F2 - Acquisition Parameters
Date_ 20201113
Time 0.34 h
INSTRUM spect
PROBHD Z119470_0283 (
PULPROG zgpg30
TD 65536
SOLVENT CDCl3
NS 1024
DS 4
SWH 29761.904 Hz
FIDRES 0.908261 Hz
AQ 1.1010048 sec
RG 206.72
DW 16.800 usec
DE 6.50 usec
TE 295.2 K
D1 2.00000000 sec
D11 0.03000000 sec
TD0 1
SFO1 125.7703643 MHz
NUC1 13C
P1 9.75 usec
PLW1 94.00000000 W
SFO2 500.1320005 MHz
NUC2 1H
CPDPRG[2 waltz16
PCPD2 80.00 usec
PLW2 25.00000000 W
PLW12 0.46495000 W
PLW13 0.23387000 W
F2 - Processing parameters
SI 32768
SF 125.7577743 MHz
WDW EM
SSB 0
LB 1.00 Hz
GB 0
PC 1.40

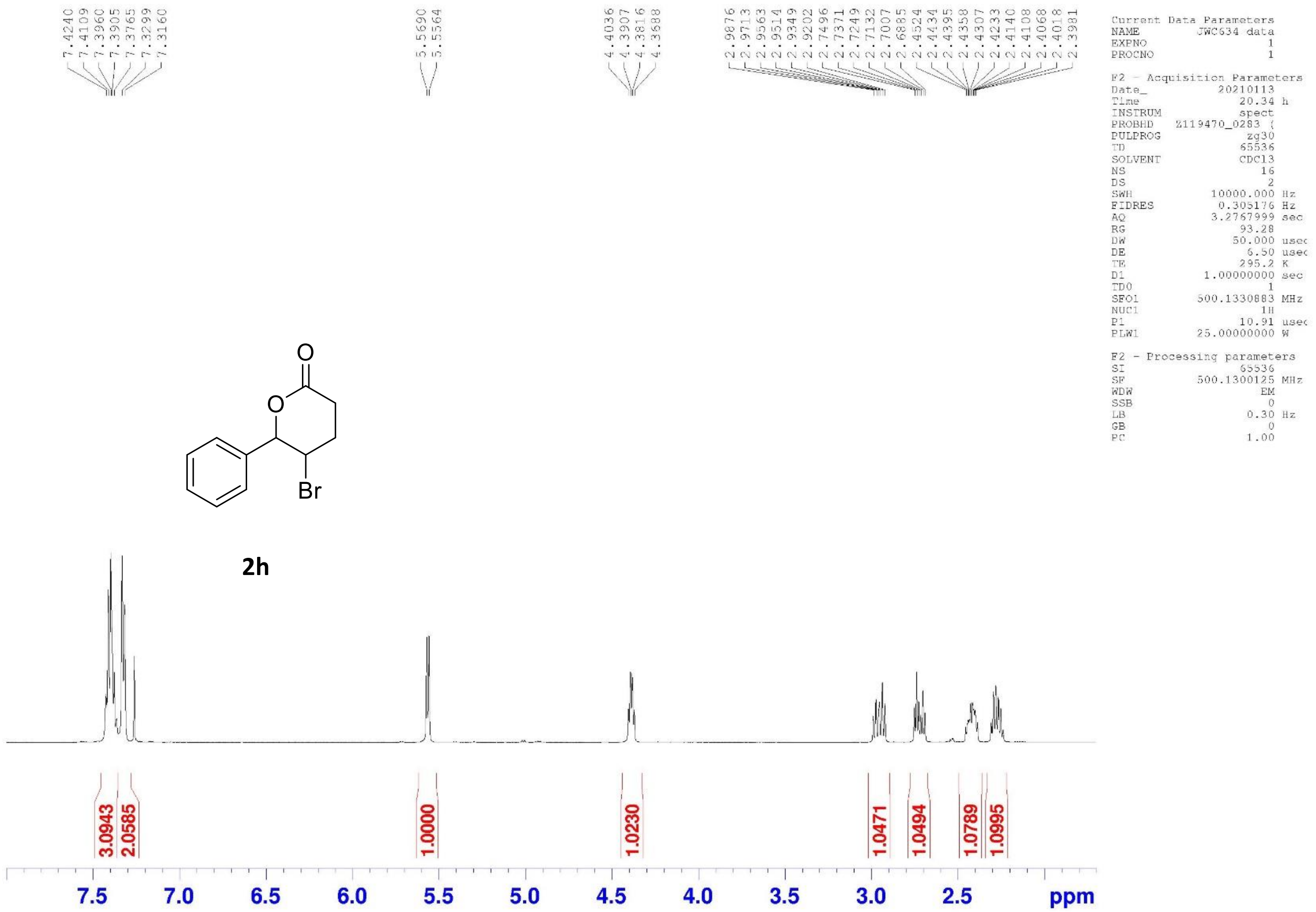
Current Data Parameters
NAME JWC634 data
EXPNO 1
PROCNO 1
F2 - Acquisition Parameters
Date_ 20210113
Time 20.34 h
INSTRUM spect
PULPROG zg30
TD 65536
SOLVENT CDCl3
NS 16
DS 2
SWH 10000.000 Hz
FIDRES 0.305176 Hz
AQ 3.2767999 sec
RG 93.28
DW 50.000 usec
DE 6.50 usec
TE 295.2 K
D1 1.00000000 sec
TD0 1
SFO1 500.1330883 MHz
NUC1 1H
P1 10.91 usec
PLW1 25.00000000 W
F2 - Processing parameters
SI 65536
SF 500.1300125 MHz
WDW EM
SSB 0
LB 0.30 Hz
GB 0
PC 1.00
O
O
Br
ppm

2h

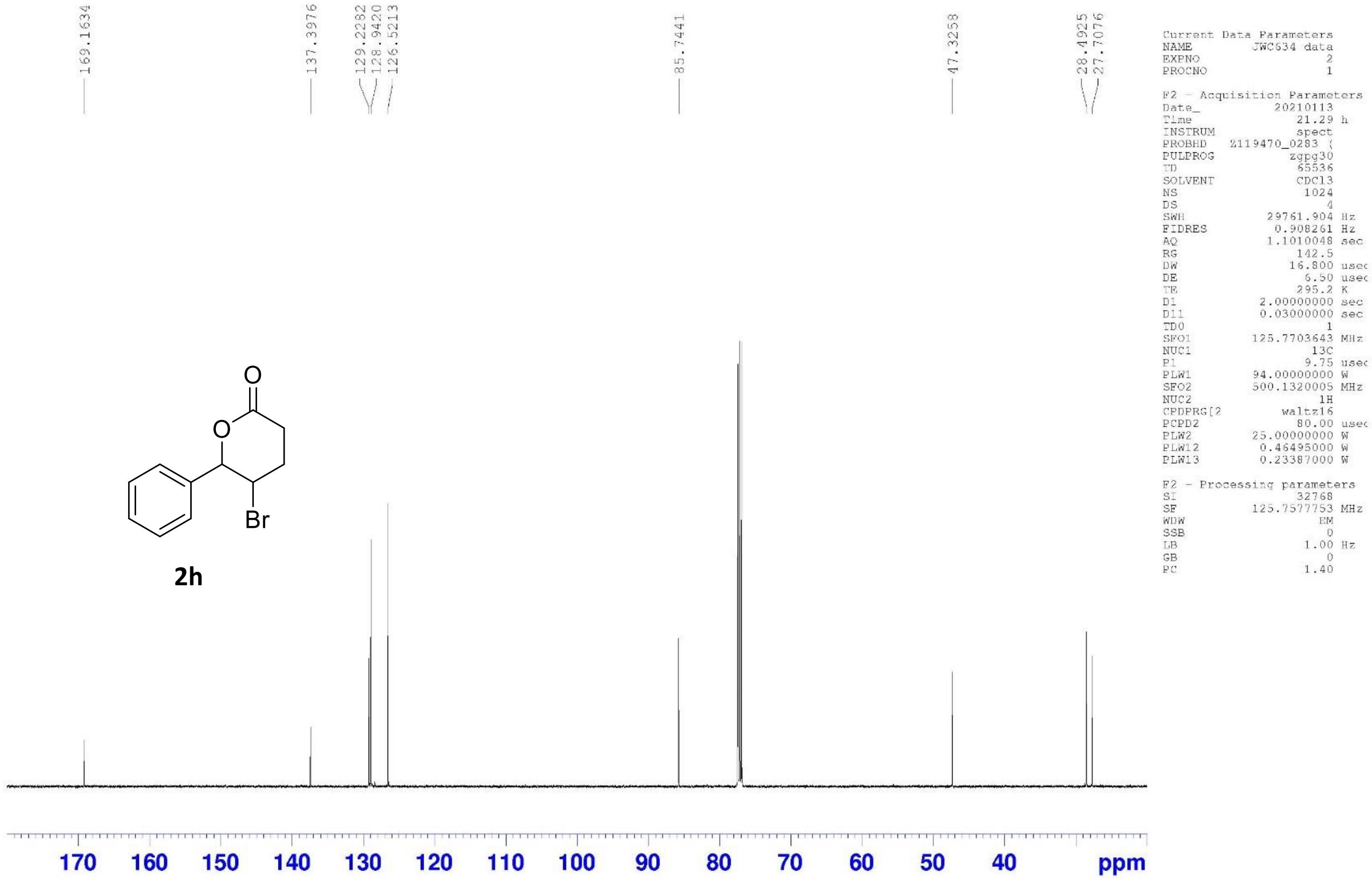

169.1634
137.3976
129.2282
128.9420
126.5213
85.7441
47.3258
28.4925
27.7076
O
O
Br
2h
170
160
150
140
130
120
110
100
90
80
70
60
50
40
ppm
Current Data Parameters
NAME JWC634 data
EXPNO 2
PROCNO 1
F2 - Acquisition Parameters
Date_ 20210113
Time 21.29 h
INSTRUM spect
PROBHD Z119470_0283 (
PULPROG zgpg30
TD 65536
SOLVENT CDCl3
NS 1024
DS 4
SWH 29761.904 Hz
FIDRES 0.908261 Hz
AQ 1.1010048 sec
RG 142.5
DW 16.800 usec
DE 6.50 usec
TE 295.2 K
D1 2.00000000 sec
D11 0.03000000 sec
TD0 1
SFO1 125.7703643 MHz
NUC1 13C
P1 9.75 usec
PLW1 94.00000000 W
SFO2 500.1320005 MHz
NUC2 1H
CPDPRG[2 waltz16
PCPD2 80.00 usec
PLW2 25.00000000 W
PLW12 0.46495000 W
PLW13 0.23387000 W
F2 - Processing parameters
SI 32768
SF 125.7577753 MHz
WDW EM
SSB 0
LB 1.00 Hz
GB 0
PC 1.40

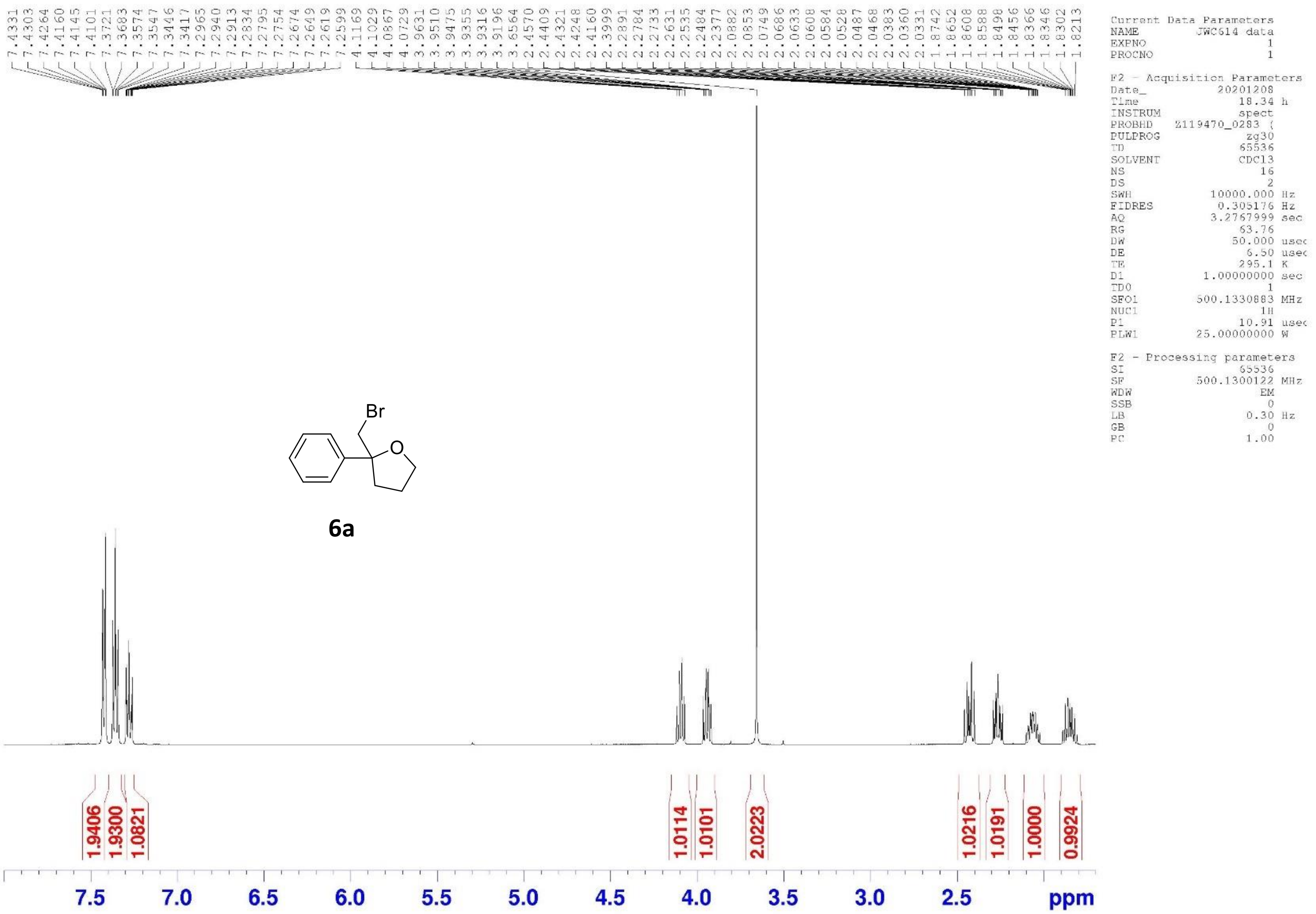
7.4331 7.4303 7.4264 7.4160 7.4145 7.4101 7.3721 7.3683 7.3574 7.3547 7.3446 7.3417 7.2965 7.2940 7.2913 7.2834 7.2795 7.2754 7.2674 7.2649 7.2619 7.2599 4.1169 4.1029 4.0867 4.0729 3.9631 3.9510 3.9475 3.9355 3.9316 3.9196 3.6564 2.4570 2.4409 2.4321 2.4248 2.4160 2.3999 2.2891 2.2784 2.2733 2.2631 2.2535 2.2484 2.2377 2.0882 2.0853 2.0749 2.0686 2.0633 2.0608 2.0584 2.0528 2.0487 2.0468 2.0383 2.0360 2.0331 1.8742 1.8652 1.8608 1.8588 1.8498 1.8456 1.8366 1.8346 1.8302 1.8213
Current Data Parameters
NAME JWC614 data
EXPNO 1
PROCNO 1
F2 - Acquisition Parameters
Date_ 20201208
Time 18.34 h
INSTRUM spect
PROBHD Z119470_0283 (
PULPROG zg30
TD 65536
SOLVENT CDCl3
NS 16
DS 2
SWH 10000.000 Hz
FIDRES 0.305176 Hz
AQ 3.2767999 sec
RG 63.76
DW 50.000 usec
DE 6.50 usec
TE 295.1 K
D1 1.00000000 sec
TD0 1
SFO1 500.1330883 MHz
NUC1 1H
P1 10.91 usec
PLW1 25.00000000 W
F2 - Processing parameters
SI 65536
SF 500.1300122 MHz
WDW EM
SSB 0
LB 0.30 Hz
GB 0
PC 1.00
Br
O
6a
1.9406 1.9300 1.0821 1.0114 1.0101 2.0223 1.0216 1.0191 1.0000 0.9924
7.5 7.0 6.5 6.0 5.5 5.0 4.5 4.0 3.5 3.0 2.5 ppm

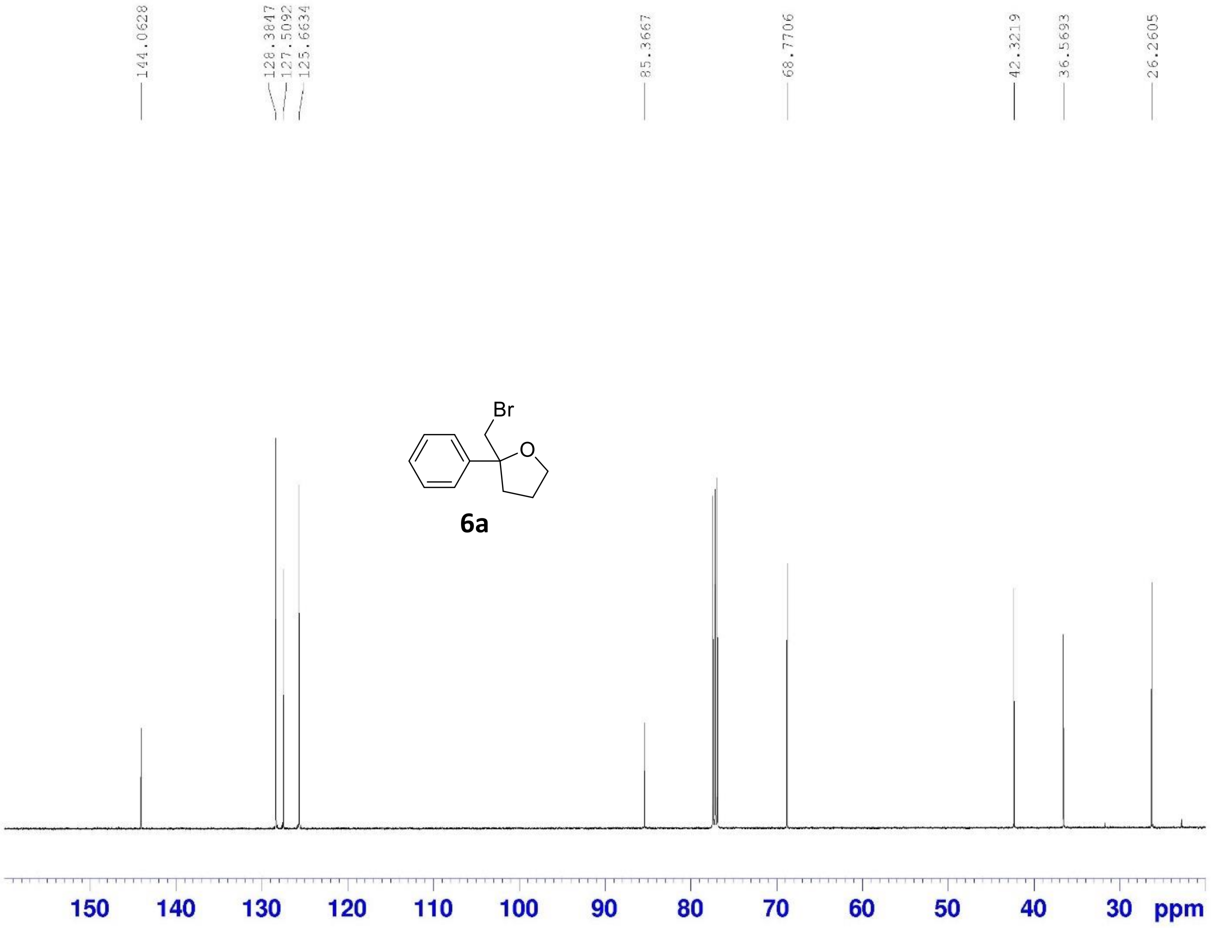


```
Current Data Parameters
NAME          JWC614 data
EXPNO                   2
PROCNO                  1

F2 - Acquisition Parameters
Date_            20201208
Time                21.27 h
INSTRUM             spect
PROBHD   Z119470_0283 (
PULPROG           zgpg30
TD                 65536
SOLVENT            CDCl3
NS                  1024
DS                     4
SWH            29761.904 Hz
FIDRES          0.908261 Hz
AQ             1.1010048 sec
RG                206.72
DW                16.800 usec
DE                  6.50 usec
TE                 295.1 K
D1            2.00000000 sec
D11           0.03000000 sec
TD0                    1
SFO1         125.7703643 MHz
NUC1                 13C
P1                  9.75 usec
PLW1         94.00000000 W
SFO2         500.1320005 MHz
NUC2                  1H
CPDPRG[2         waltz16
PCPD2              80.00 usec
PLW2         25.00000000 W
PLW12         0.46495000 W
PLW13         0.23387000 W

F2 - Processing parameters
SI                 32768
SF           125.7577772 MHz
WDW                   EM
SSB                    0
LB                  1.00 Hz
GB                     0
PC                  1.40
```

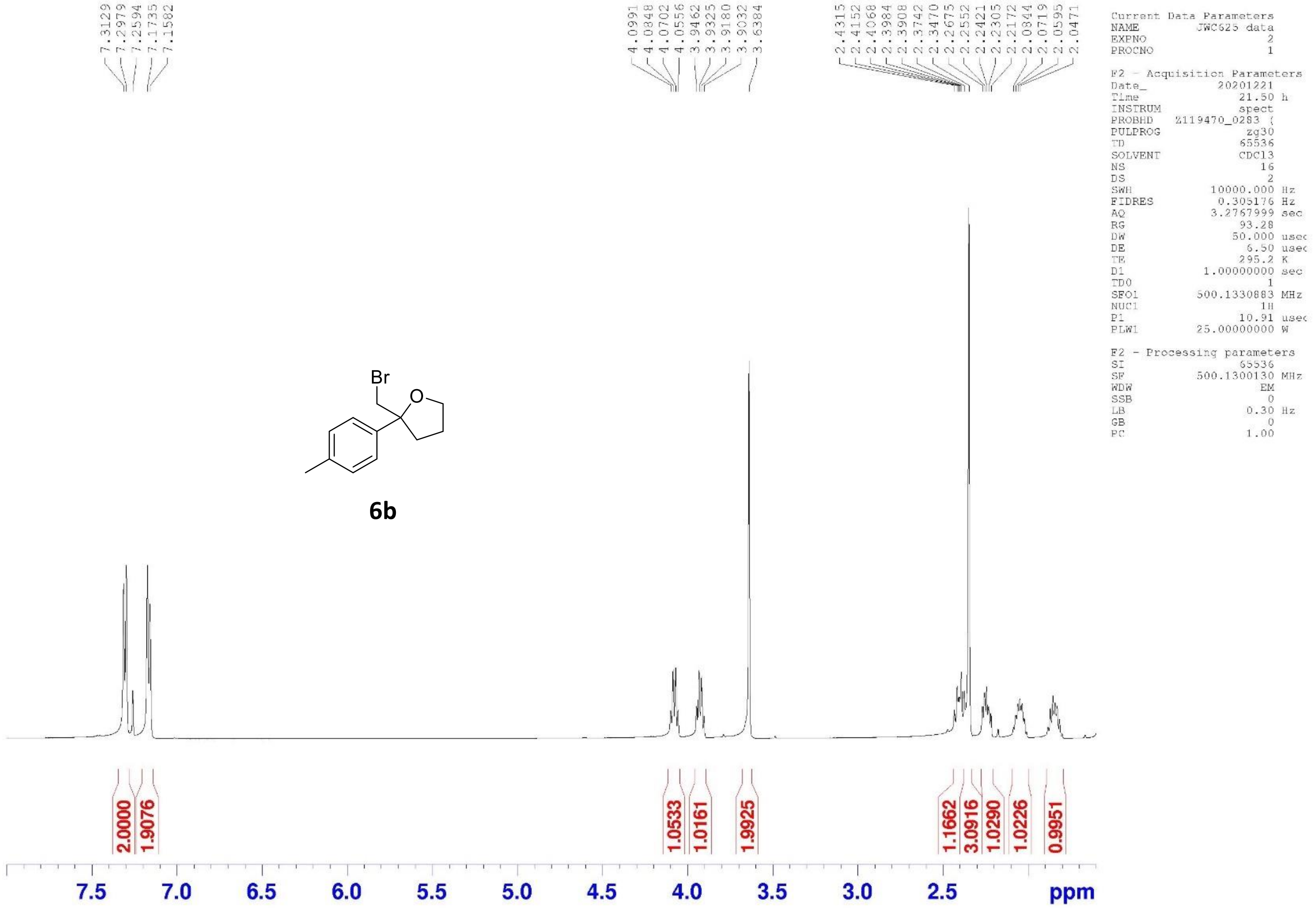
7.3129
7.2979
7.2594
7.1735
7.1582
4.0991
4.0848
4.0702
4.0556
3.9462
3.9325
3.9180
3.9032
3.6384
2.4315
2.4152
2.4068
2.3984
2.3908
2.3742
2.3470
2.2675
2.2552
2.2421
2.2305
2.2172
2.0844
2.0719
2.0595
2.0471
Current Data Parameters
NAME JWC625 data
EXPNO 2
PROCNO 1
F2 - Acquisition Parameters
Date_ 20201221
Time 21.50 h
INSTRUM spect
PROBHD Z119470_0283 (
PULPROG zg30
TD 65536
SOLVENT CDCl3
NS 16
DS 2
SWH 10000.000 Hz
FIDRES 0.305176 Hz
AQ 3.2767999 sec
RG 93.28
DW 50.000 usec
DE 6.50 usec
TE 295.2 K
D1 1.00000000 sec
TD0 1
SFO1 500.1330883 MHz
NUC1 1H
P1 10.91 usec
PLW1 25.00000000 W
F2 - Processing parameters
SI 65536
SF 500.1300130 MHz
WDW EM
SSB 0
LB 0.30 Hz
GB 0
PC 1.00
Br
O
6b
2.0000
1.9076
1.0533
1.0161
1.9925
1.1662
3.0916
1.0290
1.0226
0.9951
7.5
7.0
6.5
6.0
5.5
5.0
4.5
4.0
3.5
3.0
2.5
ppm

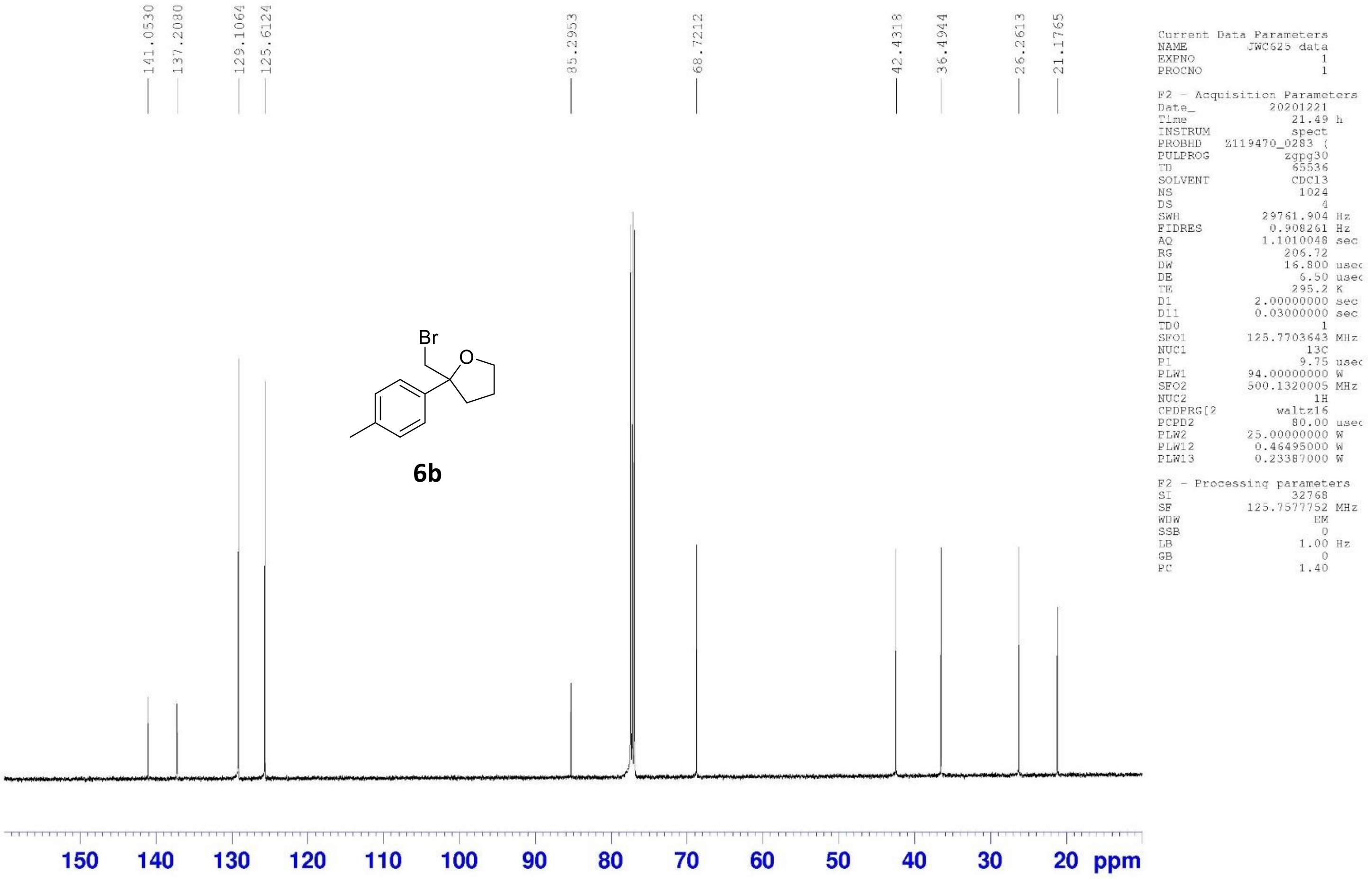
141.0530
137.2080
129.1064
125.6124
85.2953
68.7212
42.4318
36.4944
26.2613
21.1765
Br
O
6b
150
140
130
120
110
100
90
80
70
60
50
40
30
20
ppm
Current Data Parameters
NAME JWC625 data
EXPNO 1
PROCNO 1
F2 - Acquisition Parameters
Date_ 20201221
Time 21.49 h
INSTRUM spect
PROBHD Z119470_0283 (
PULPROG zgpg30
TD 65536
SOLVENT CDCl3
NS 1024
DS 4
SWH 29761.904 Hz
FIDRES 0.908261 Hz
AQ 1.1010048 sec
RG 206.72
DW 16.800 usec
DE 6.50 usec
TE 295.2 K
D1 2.00000000 sec
D11 0.03000000 sec
TD0 1
SFO1 125.7703643 MHz
NUC1 13C
P1 9.75 usec
PLW1 94.00000000 W
SFO2 500.1320005 MHz
NUC2 1H
CPDPRG[2 waltz16
PCPD2 80.00 usec
PLW2 25.00000000 W
PLW12 0.46495000 W
PLW13 0.23387000 W
F2 - Processing parameters
SI 32768
SF 125.7577752 MHz
WDW EM
SSB 0
LB 1.00 Hz
GB 0
PC 1.40

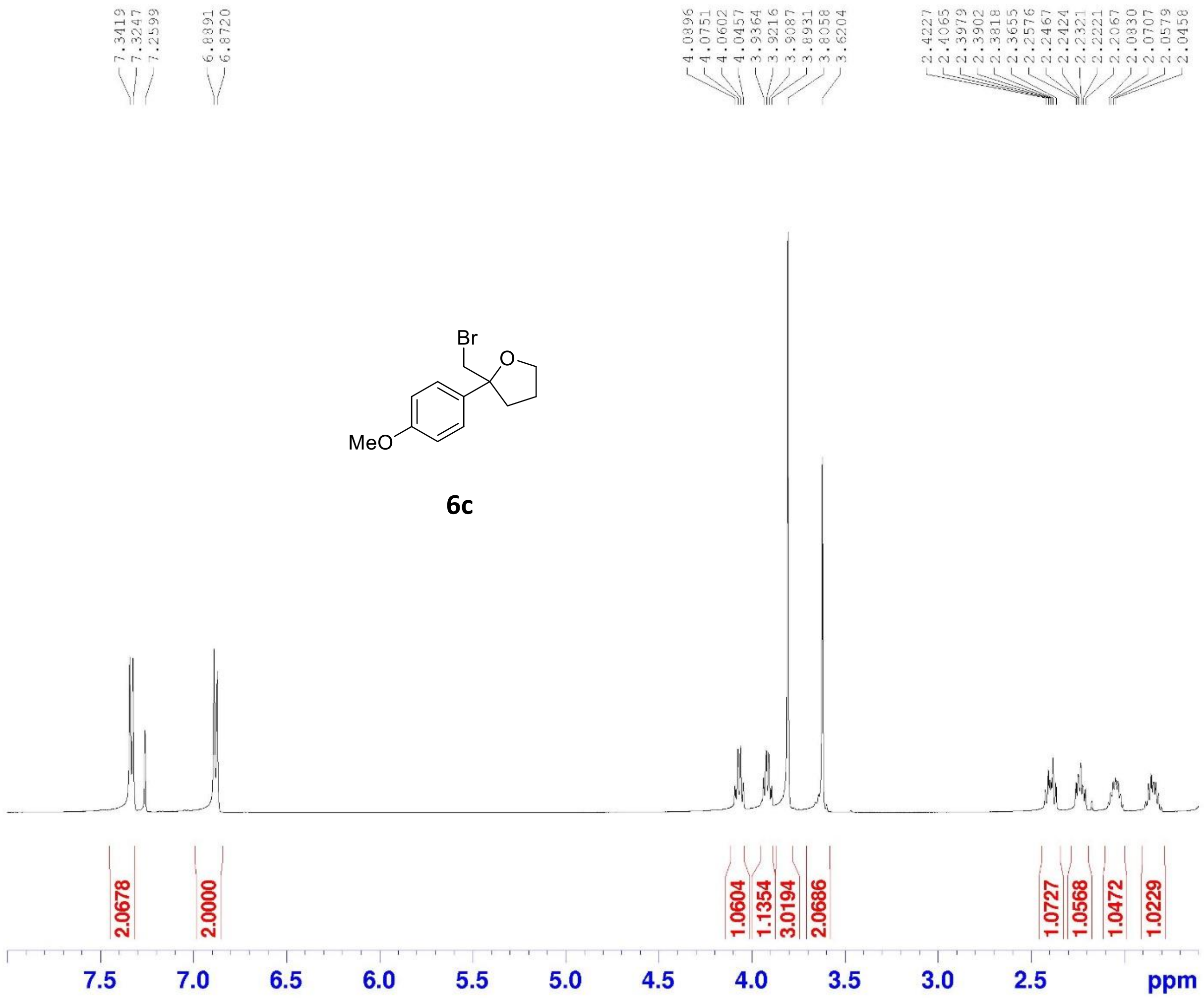

6c

Current Data Parameters
NAME JWC626 data
EXPNO 2
PROCNO 1

F2 - Acquisition Parameters
Date_ 20201221
Time 22.28 h
INSTRUM spect
PROBHD Z119470_0283 (
PULPROG zg30
TD 65536
SOLVENT CDCl3
NS 16
DS 2
SWH 10000.000 Hz
FIDRES 0.305176 Hz
AQ 3.2767999 sec
RG 117.01
DW 50.000 usec
DE 6.50 usec
TE 295.2 K
D1 1.00000000 sec
TD0 1
SFO1 500.1330883 MHz
NUC1 1H
P1 10.91 usec
PLW1 25.00000000 W

F2 - Processing parameters
SI 65536
SF 500.1300127 MHz
WDW EM
SSB 0
LB 0.30 Hz
GB 0
PC 1.00

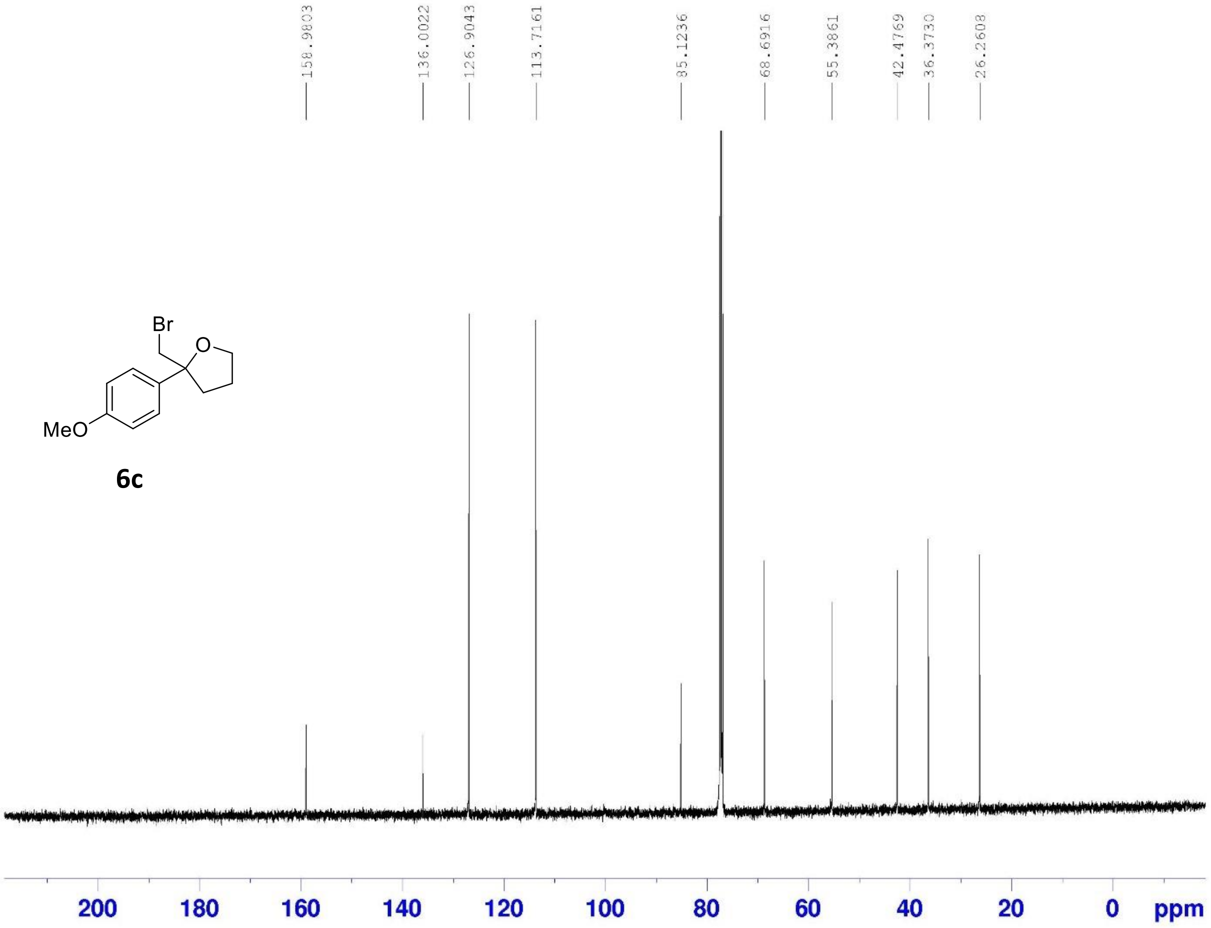

6c


```
Current Data Parameters
NAME          JWC626 data
EXPNO                   1
PROCNO                  1

F2 - Acquisition Parameters
Date_            20201221
Time                22.26 h
INSTRUM             spect
PROBHD   Z119470_0283 (
PULPROG            zgpg30
TD                  65536
SOLVENT             CDCl3
NS                    625
DS                      4
SWH             29761.904 Hz
FIDRES           0.908261 Hz
AQ              1.1010048 sec
RG                 206.72
DW                 16.800 usec
DE                   6.50 usec
TE                  295.1 K
D1             2.00000000 sec
D11            0.03000000 sec
TD0                     1
SFO1          125.7703643 MHz
NUC1                  13C
P1                   9.75 usec
PLW1          94.00000000 W
SFO2          500.1320005 MHz
NUC2                   1H
CPDPRG[2          waltz16
PCPD2               80.00 usec
PLW2          25.00000000 W
PLW12          0.46495000 W
PLW13          0.23387000 W

F2 - Processing parameters
SI                  32768
SF            125.7577743 MHz
WDW                    EM
SSB                     0
LB                   1.00 Hz
GB                      0
PC                   1.40
```

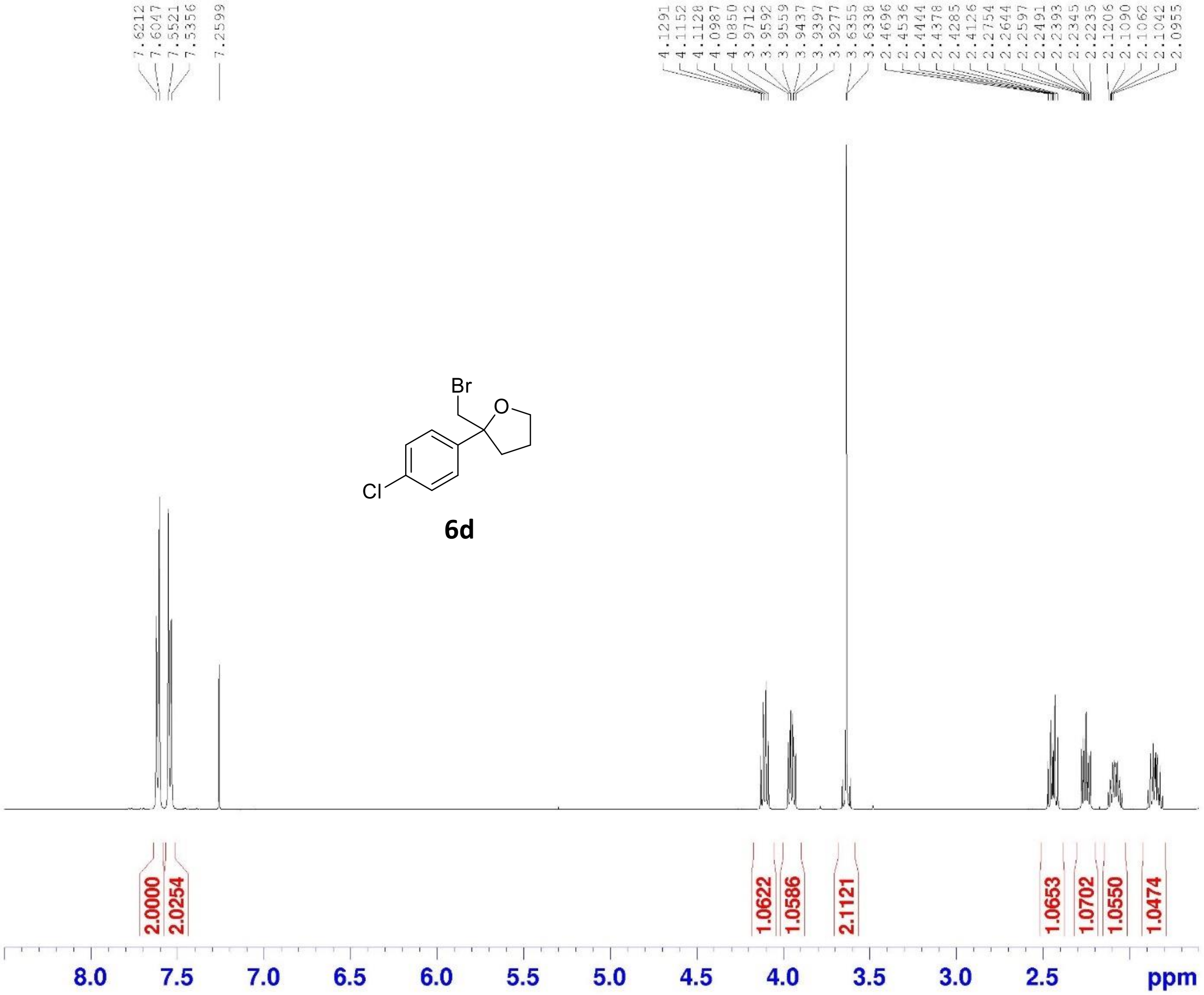

```
Current Data Parameters
NAME          JWC619 data
EXPNO                  1
PROCNO                 1

F2 - Acquisition Parameters
Date_           20201208
Time               18.38 h
INSTRUM            spect
PROBHD   Z119470_0283 (
PULPROG             zg30
TD                 65536
SOLVENT            CDCl3
NS                    16
DS                     2
SWH            10000.000 Hz
FIDRES          0.305176 Hz
AQ             3.2767999 sec
RG                 83.35
DW                50.000 usec
DE                  6.50 usec
TE                 295.2 K
D1            1.00000000 sec
TD0                    1
SFO1         500.1330883 MHz
NUC1                  1H
P1                 10.91 usec
PLW1         25.00000000 W

F2 - Processing parameters
SI                 65536
SF           500.1300122 MHz
WDW                   EM
SSB                    0
LB                  0.30 Hz
GB                     0
PC                  1.00
```

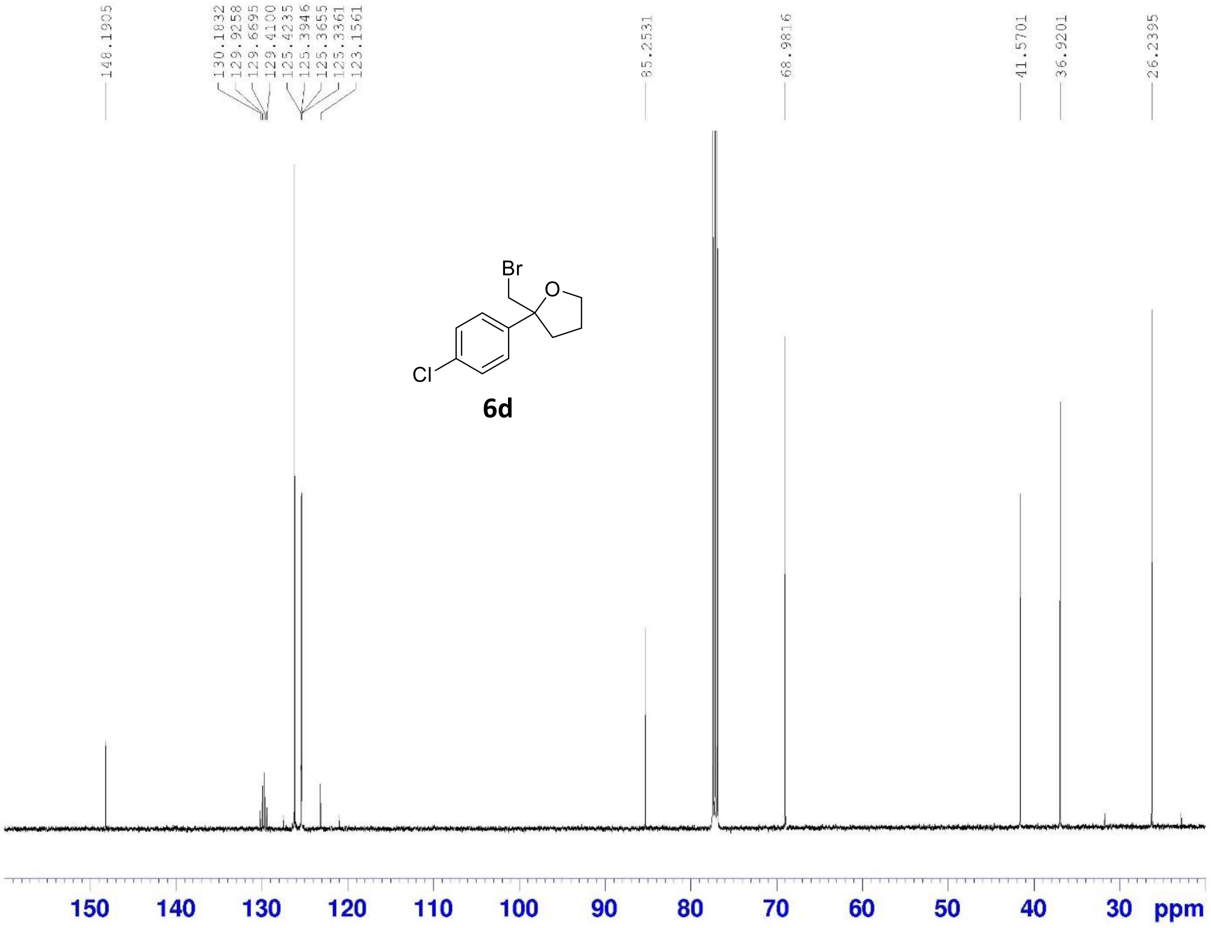


```
Current Data Parameters
NAME          JWC619 data
EXPNO                   2
PROCNO                  1

F2 - Acquisition Parameters
Date_            20201208
Time                22.23 h
INSTRUM             spect
PROBHD   Z119470_0283 (
PULPROG           zgpg30
TD                 65536
SOLVENT            CDCl3
NS                  1024
DS                     4
SWH            29761.904 Hz
FIDRES          0.908261 Hz
AQ             1.1010048 sec
RG                206.72
DW                16.800 usec
DE                  6.50 usec
TE                 295.1 K
D1            2.00000000 sec
D11           0.03000000 sec
TD0                    1
SFO1         125.7703643 MHz
NUC1                 13C
P1                  9.75 usec
PLW1         94.00000000 W
SFO2         500.1320005 MHz
NUC2                  1H
CPDPRG[2         waltz16
PCPD2              80.00 usec
PLW2         25.00000000 W
PLW12         0.46495000 W
PLW13         0.23387000 W

F2 - Processing parameters
SI                 32768
SF           125.7577734 MHz
WDW                   EM
SSB                    0
LB                  1.00 Hz
GB                     0
PC                  1.40
```

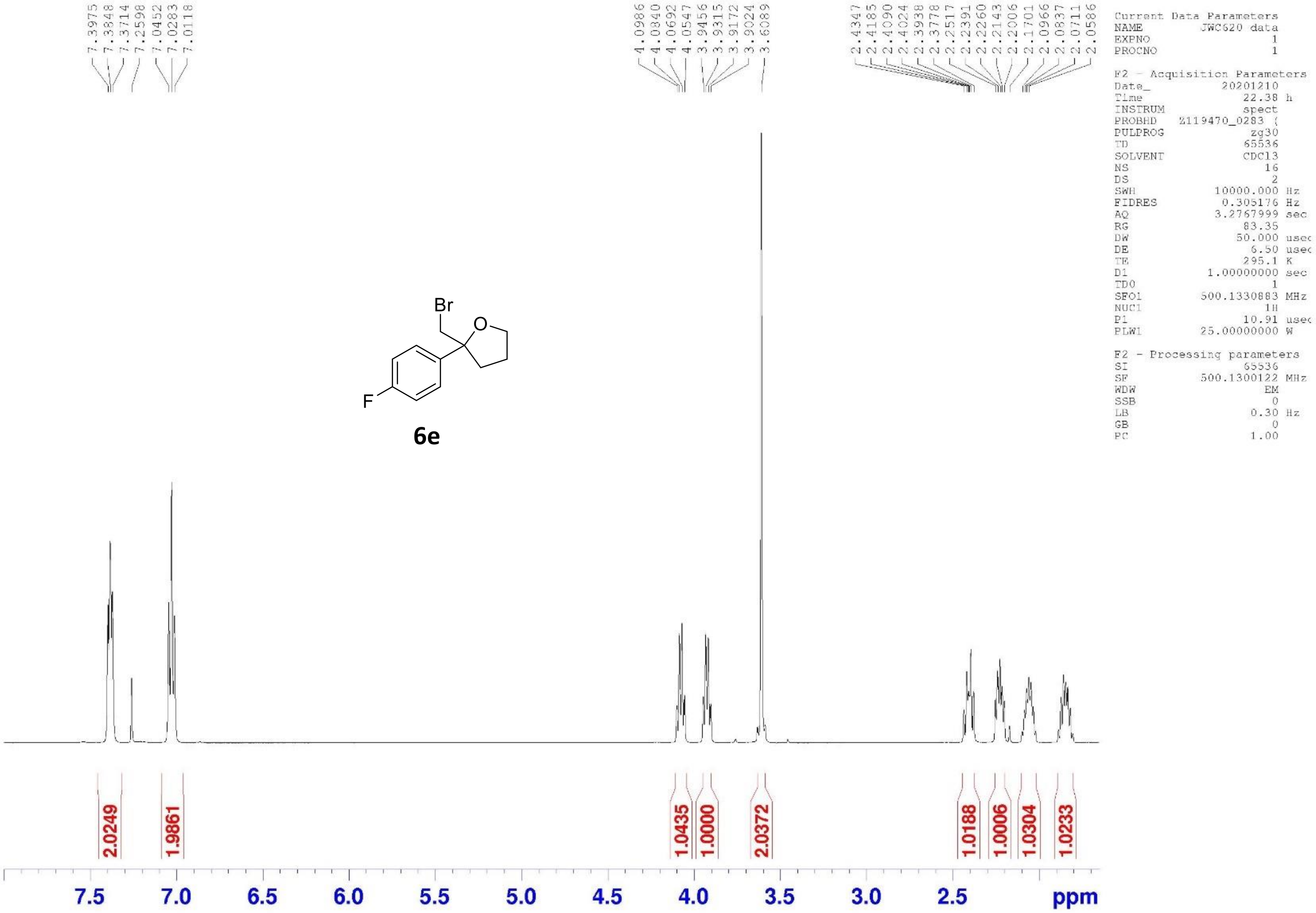
7.3975
7.3848
7.3714
7.2598
7.0452
7.0283
7.0118
4.0986
4.0840
4.0692
4.0547
3.9456
3.9315
3.9172
3.9024
3.6089
2.4347
2.4185
2.4090
2.4024
2.3938
2.3778
2.2517
2.2391
2.2260
2.2143
2.2006
2.1701
2.0966
2.0837
2.0711
2.0586
Br
O
F
6e
2.0249
1.9861
1.0435
1.0000
2.0372
1.0188
1.0006
1.0304
1.0233
7.5
7.0
6.5
6.0
5.5
5.0
4.5
4.0
3.5
3.0
2.5
ppm
Current Data Parameters
NAME JWC620 data
EXPNO 1
PROCNO 1
F2 - Acquisition Parameters
Date_ 20201210
Time 22.38 h
INSTRUM spect
PROBHD Z119470_0283 (
PULPROG zg30
TD 65536
SOLVENT CDCl3
NS 16
DS 2
SWH 10000.000 Hz
FIDRES 0.305176 Hz
AQ 3.2767999 sec
RG 83.35
DW 50.000 usec
DE 6.50 usec
TE 295.1 K
D1 1.00000000 sec
TD0 1
SFO1 500.1330883 MHz
NUC1 1H
P1 10.91 usec
PLW1 25.00000000 W
F2 - Processing parameters
SI 65536
SF 500.1300122 MHz
WDW EM
SSB 0
LB 0.30 Hz
GB 0
PC 1.00

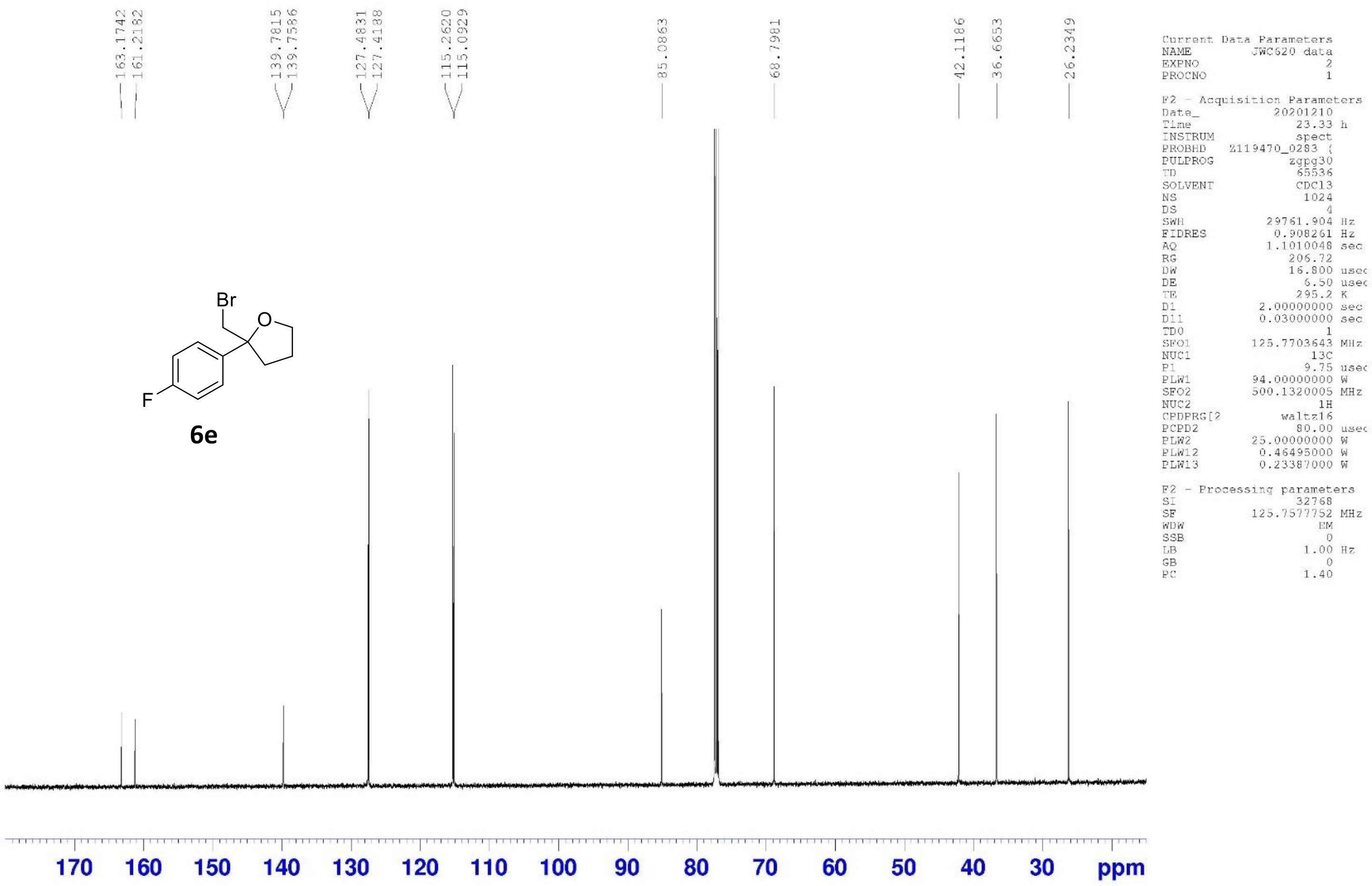
163.1742
161.2182
139.7815
139.7586
127.4831
127.4188
115.2620
115.0929
85.0863
68.7981
42.1186
36.6653
26.2349
Br
O
F
6e
170 160 150 140 130 120 110 100 90 80 70 60 50 40 30 ppm
Current Data Parameters
NAME JWC620 data
EXPNO 2
PROCNO 1
F2 - Acquisition Parameters
Date_ 20201210
Time 23.33 h
INSTRUM spect
PROBHD Z119470_0283 (
PULPROG zgpg30
TD 65536
SOLVENT CDCl3
NS 1024
DS 4
SWH 29761.904 Hz
FIDRES 0.908261 Hz
AQ 1.1010048 sec
RG 206.72
DW 16.800 usec
DE 6.50 usec
TE 295.2 K
D1 2.00000000 sec
D11 0.03000000 sec
TD0 1
SFO1 125.7703643 MHz
NUC1 13C
P1 9.75 usec
PLW1 94.00000000 W
SFO2 500.1320005 MHz
NUC2 1H
CPDPRG[2 waltz16
PCPD2 80.00 usec
PLW2 25.00000000 W
PLW12 0.46495000 W
PLW13 0.23387000 W
F2 - Processing parameters
SI 32768
SF 125.7577752 MHz
WDW EM
SSB 0
LB 1.00 Hz
GB 0
PC 1.40

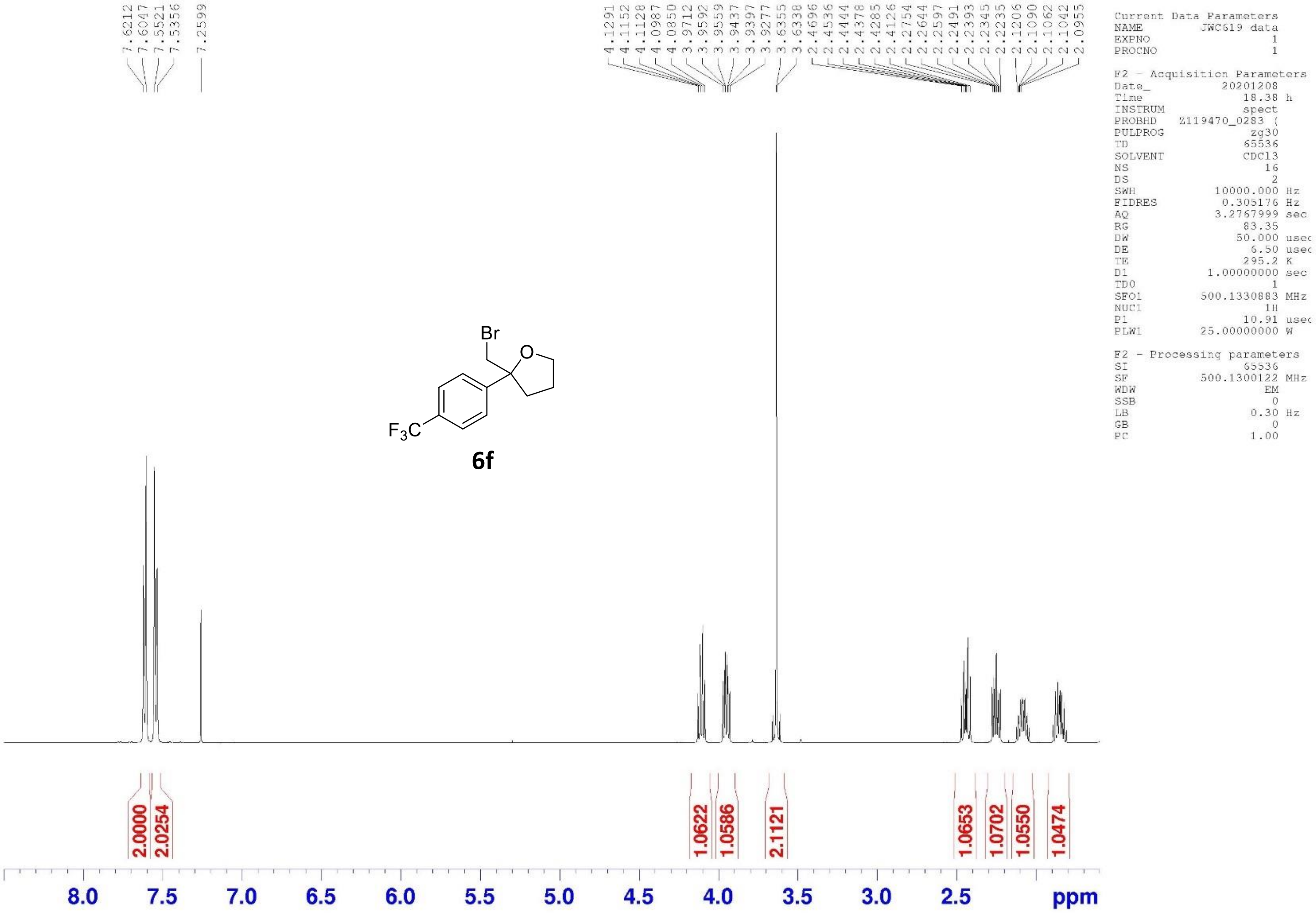

6f

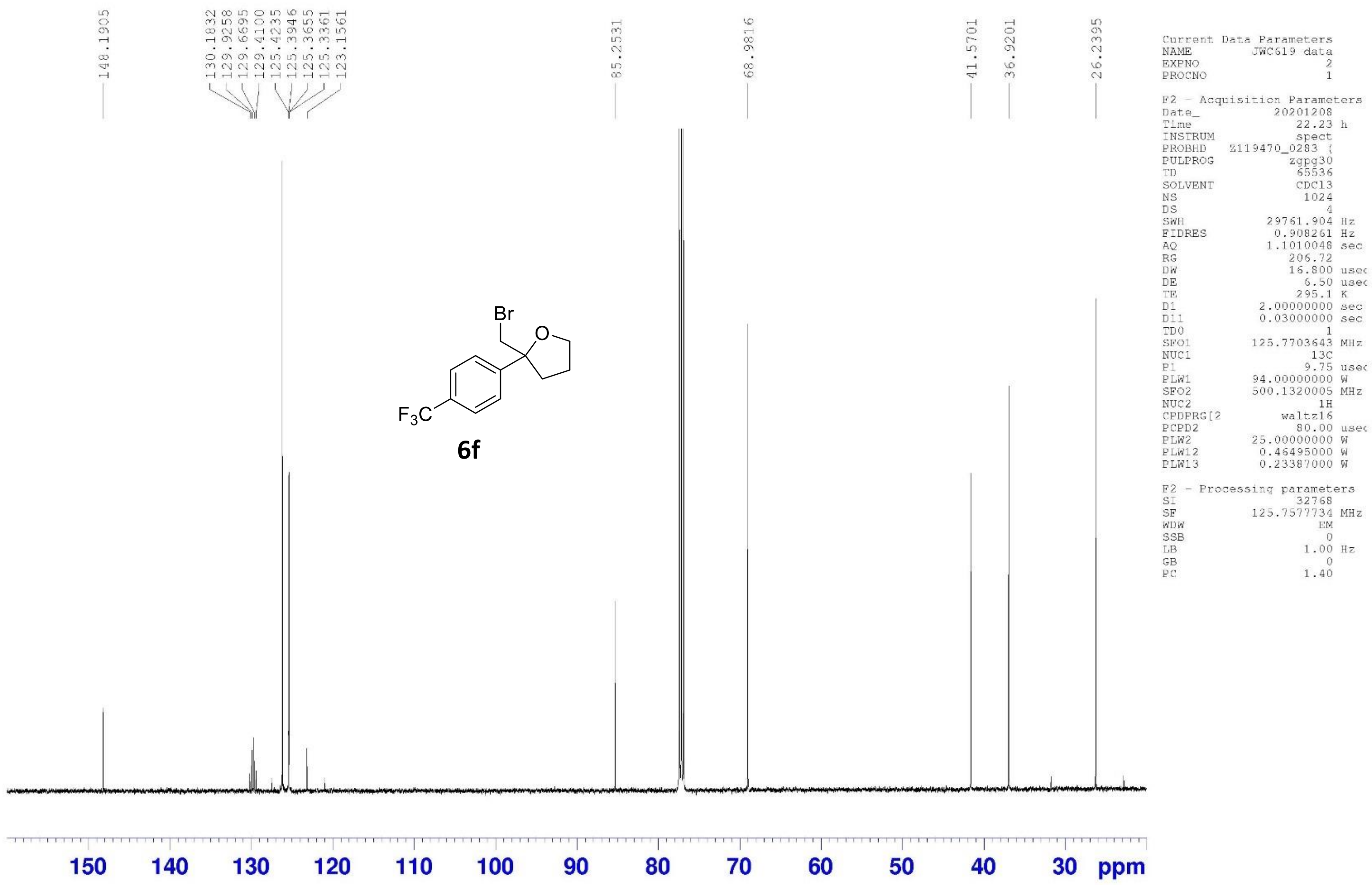

148.1905
130.1832
129.9258
129.6695
129.4100
125.4235
125.3946
125.3655
125.3361
123.1561
85.2531
68.9816
41.5701
36.9201
26.2395
Br
O
F3C
6f
150
140
130
120
110
100
90
80
70
60
50
40
30
ppm
Current Data Parameters
NAME JWC619 data
EXPNO 2
PROCNO 1
F2 - Acquisition Parameters
Date_ 20201208
Time 22.23 h
INSTRUM spect
PROBHD Z119470_0283 (
PULPROG zgpg30
TD 65536
SOLVENT CDCl3
NS 1024
DS 4
SWH 29761.904 Hz
FIDRES 0.908261 Hz
AQ 1.1010048 sec
RG 206.72
DW 16.800 usec
DE 6.50 usec
TE 295.1 K
D1 2.00000000 sec
D11 0.03000000 sec
TD0 1
SFO1 125.7703643 MHz
NUC1 13C
P1 9.75 usec
PLW1 94.00000000 W
SFO2 500.1320005 MHz
NUC2 1H
CPDPRG[2 waltz16
PCPD2 80.00 usec
PLW2 25.00000000 W
PLW12 0.46495000 W
PLW13 0.23387000 W
F2 - Processing parameters
SI 32768
SF 125.7577734 MHz
WDW EM
SSB 0
LB 1.00 Hz
GB 0
PC 1.40

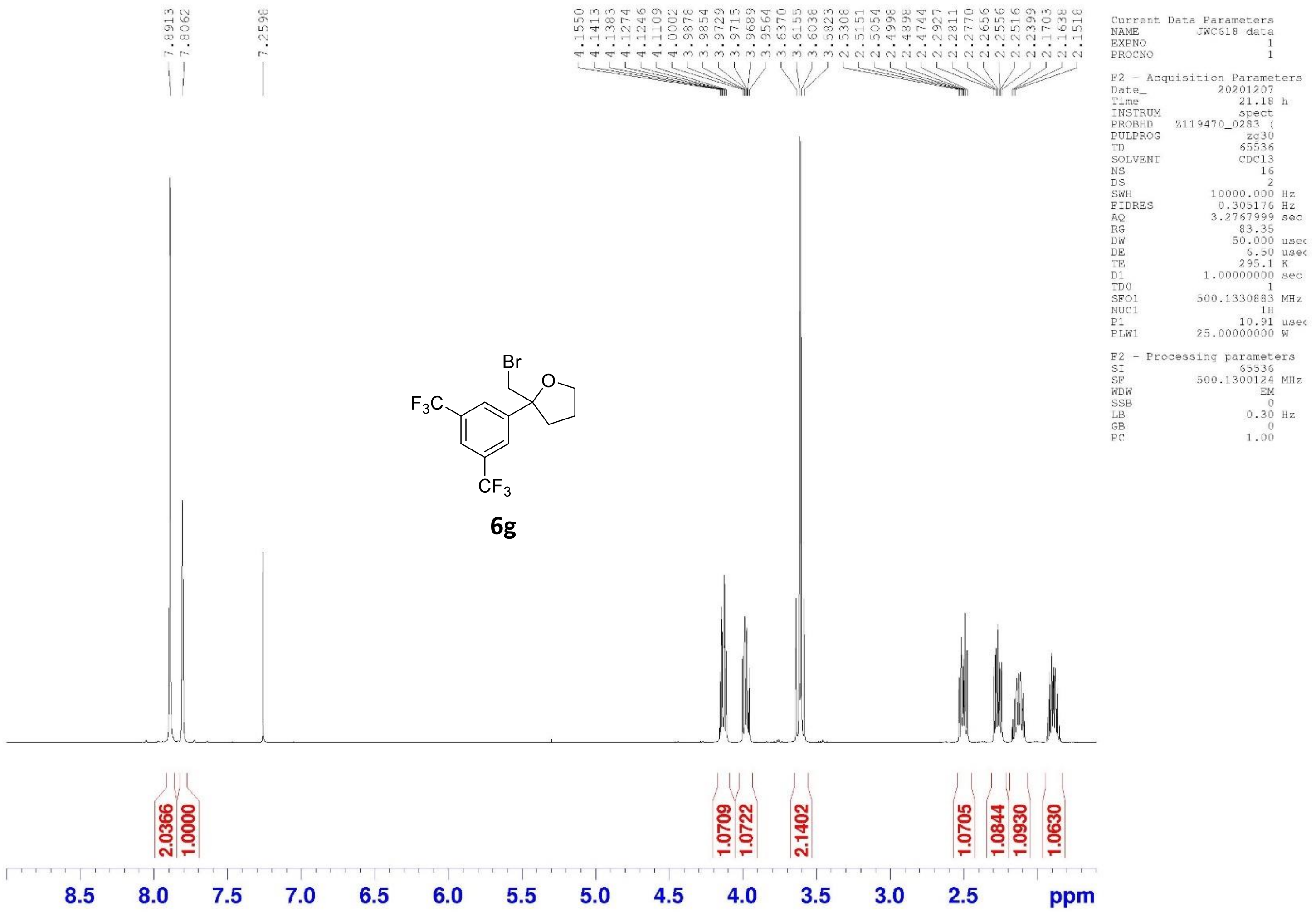

6g

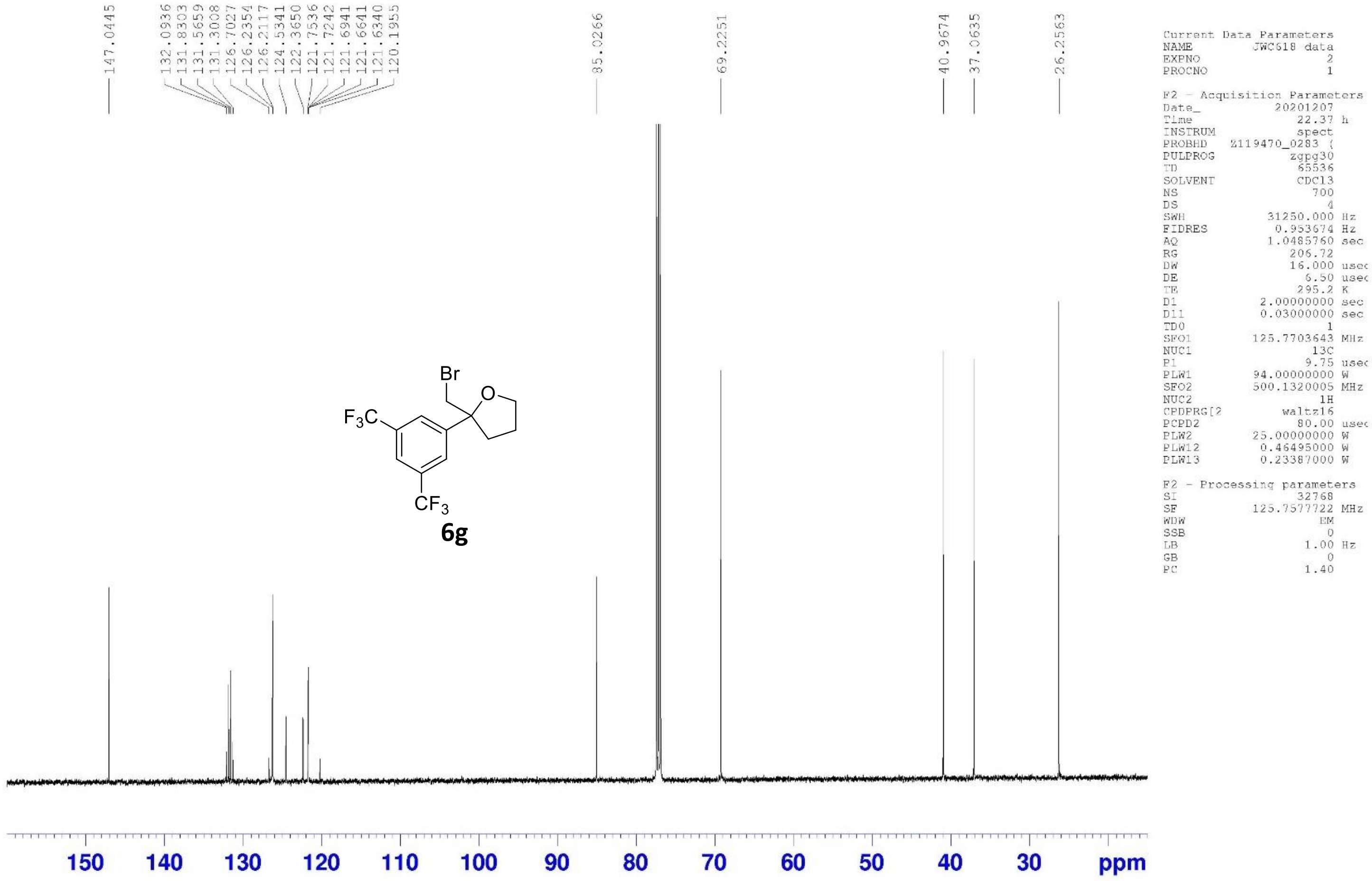
147.0445
132.0936
131.8303
131.5659
131.3008
126.7027
126.2354
126.2117
124.5341
122.3650
121.7536
121.7242
121.6941
121.6641
121.6340
120.1955
85.0266
69.2251
40.9674
37.0635
26.2563
Br
O
F3C
CF3
6g
150 140 130 120 110 100 90 80 70 60 50 40 30 ppm
Current Data Parameters
NAME JWC618 data
EXPNO 2
PROCNO 1
F2 - Acquisition Parameters
Date_ 20201207
Time 22.37 h
INSTRUM spect
PROBHD Z119470_0283 (
PULPROG zgpg30
TD 65536
SOLVENT CDCl3
NS 700
DS 4
SWH 31250.000 Hz
FIDRES 0.953674 Hz
AQ 1.0485760 sec
RG 206.72
DW 16.000 usec
DE 6.50 usec
TE 295.2 K
D1 2.00000000 sec
D11 0.03000000 sec
TD0 1
SFO1 125.7703643 MHz
NUC1 13C
P1 9.75 usec
PLW1 94.00000000 W
SFO2 500.1320005 MHz
NUC2 1H
CPDPRG[2 waltz16
PCPD2 80.00 usec
PLW2 25.00000000 W
PLW12 0.46495000 W
PLW13 0.23387000 W
F2 - Processing parameters
SI 32768
SF 125.7577722 MHz
WDW EM
SSB 0
LB 1.00 Hz
GB 0
PC 1.40

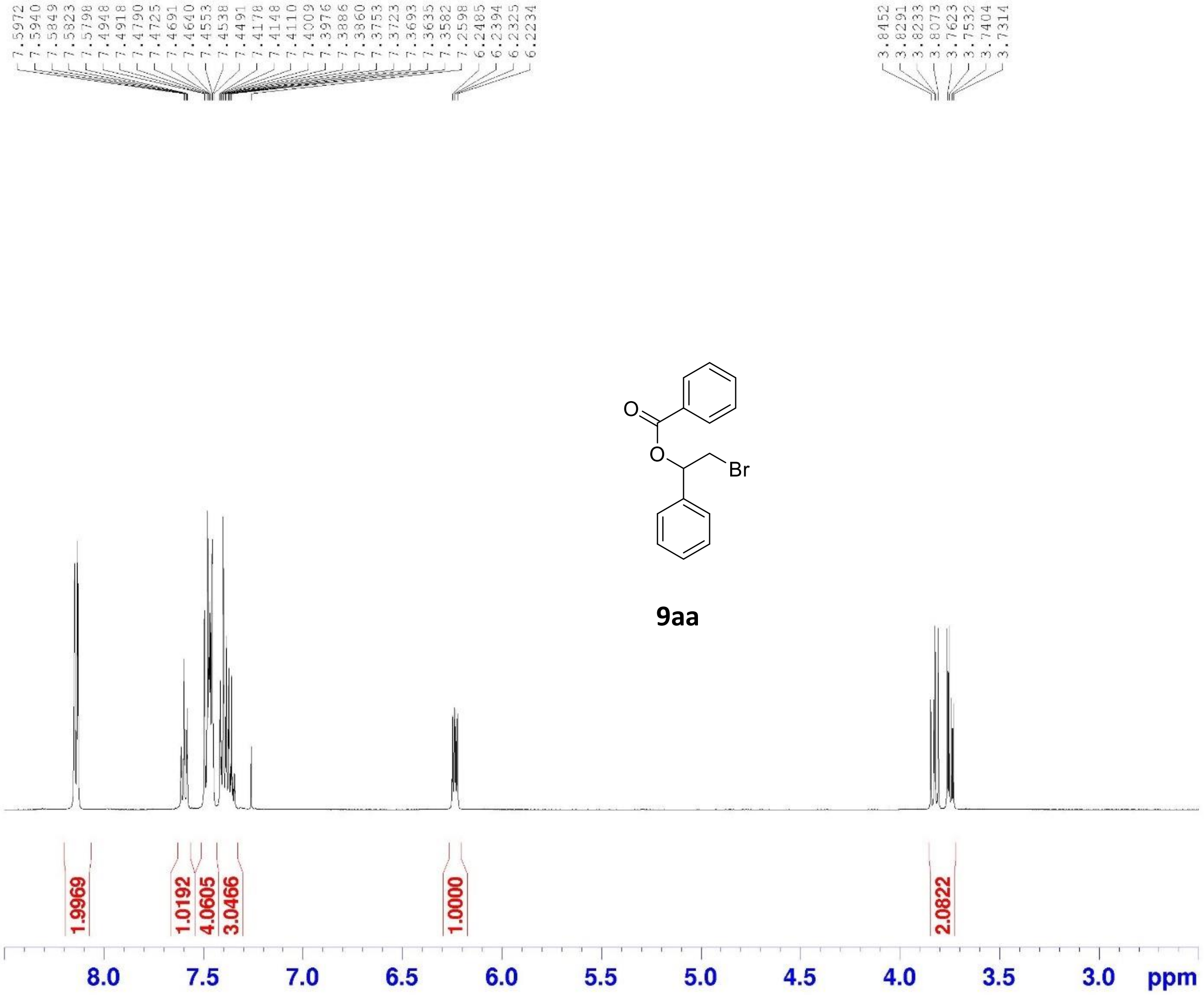

Current Data Parameters
NAME JWC395pm A pure
EXPNO 1
PROCNO 1

F2 - Acquisition Parameters
Date_ 20190704
Time 23.20 h
INSTRUM spect
PROBHD Z149001_0010 (
PULPROG zg30
TD 65536
SOLVENT CDCl3
NS 16
DS 2
SWH 10000.000 Hz
FIDRES 0.305176 Hz
AQ 3.2767999 sec
RG 30.85
DW 50.000 usec
DE 10.00 usec
TE 298.0 K
D1 1.00000000 sec
TD0 1
SFO1 500.1330883 MHz
NUC1 1H
P1 11.25 usec
PLW1 15.00000000 W

F2 - Processing parameters
SI 65536
SF 500.1300123 MHz
WDW EM
SSB 0
LB 0.30 Hz
GB 0
PC 1.00

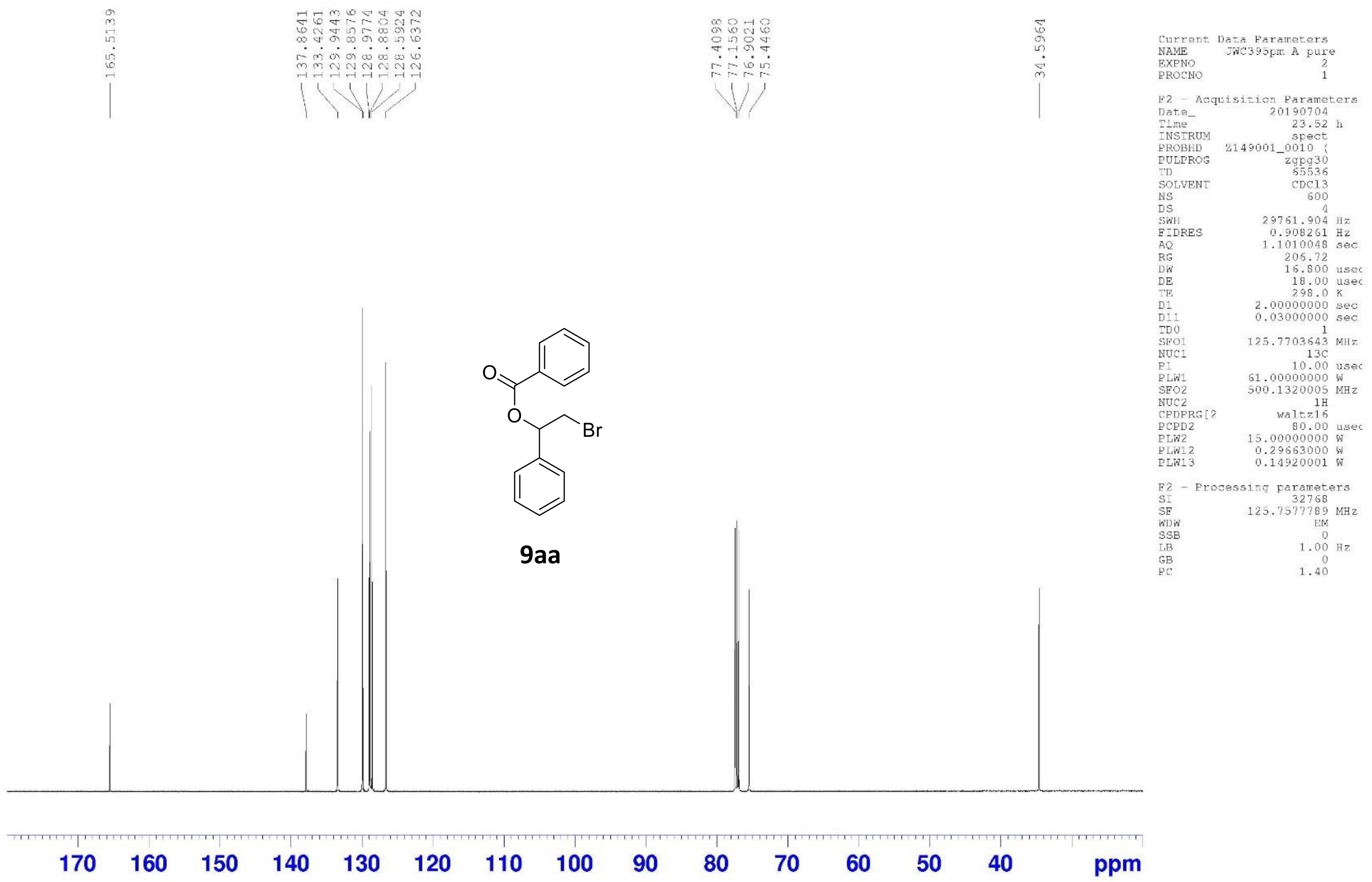
165.5139
137.8641
133.4261
129.9443
129.8576
128.9774
128.8804
128.5924
126.6372
77.4098
77.1560
76.9021
75.4460
34.5964
O
O
Br
9aa
170
160
150
140
130
120
110
100
90
80
70
60
50
40
ppm
Current Data Parameters
NAME JWC395pm A pure
EXPNO 2
PROCNO 1
F2 - Acquisition Parameters
Date_ 20190704
Time 23.52 h
INSTRUM spect
PROBHD Z149001_0010 (
PULPROG zgpg30
TD 65536
SOLVENT CDCl3
NS 600
DS 4
SWH 29761.904 Hz
FIDRES 0.908261 Hz
AQ 1.1010048 sec
RG 206.72
DW 16.800 usec
DE 18.00 usec
TE 298.0 K
D1 2.00000000 sec
D11 0.03000000 sec
TD0 1
SFO1 125.7703643 MHz
NUC1 13C
P1 10.00 usec
PLW1 61.00000000 W
SFO2 500.1320005 MHz
NUC2 1H
CPDPRG[2 waltz16
PCPD2 80.00 usec
PLW2 15.00000000 W
PLW12 0.29663000 W
PLW13 0.14920001 W
F2 - Processing parameters
SI 32768
SF 125.7577789 MHz
WDW EM
SSB 0
LB 1.00 Hz
GB 0
PC 1.40

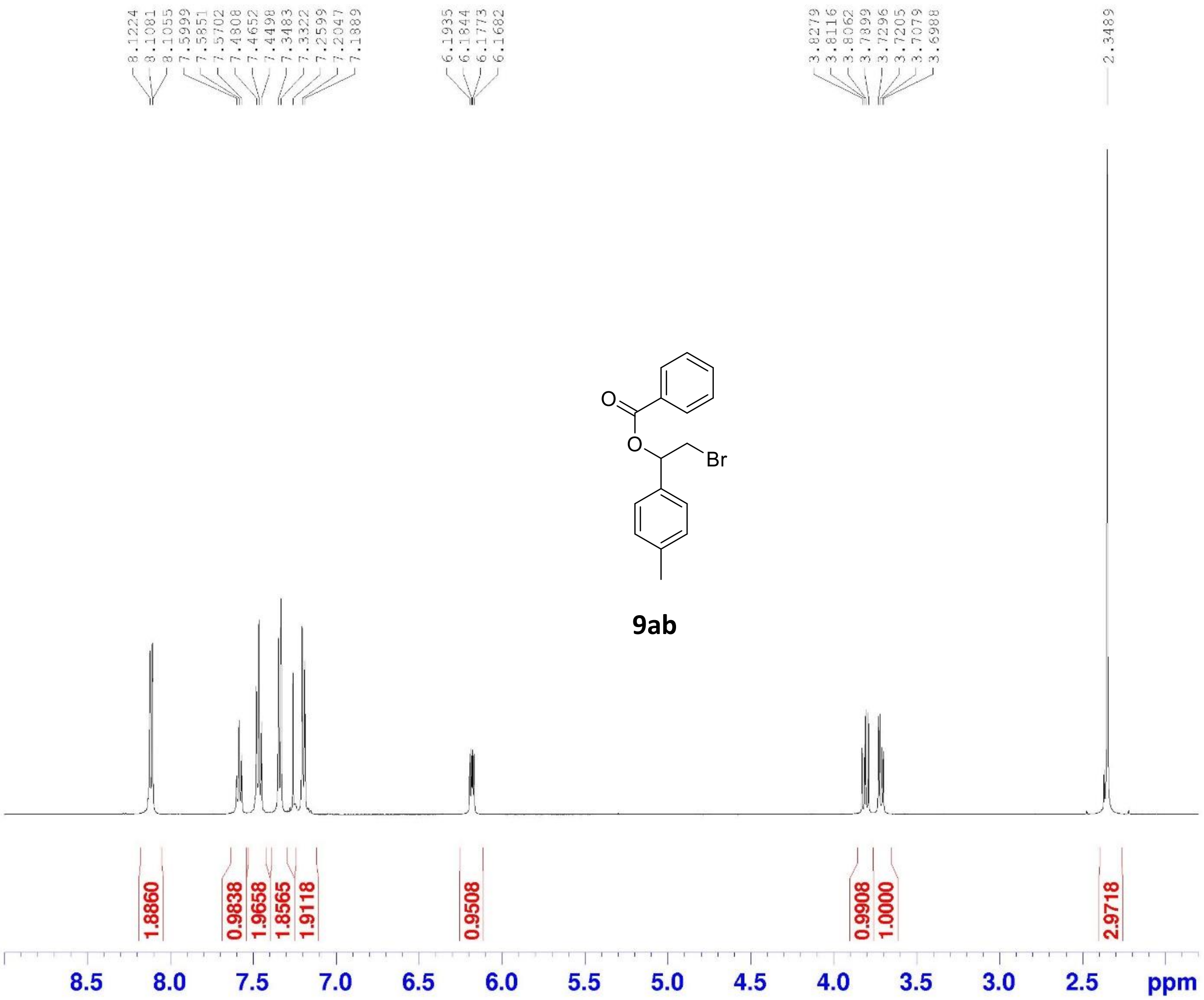


```
Current Data Parameters
NAME          JWC525 p3
EXPNO                 1
PROCNO                1

F2 - Acquisition Parameters
Date_          20200722
Time              18.45 h
INSTRUM           spect
PROBHD   Z119470_0283 (
PULPROG            zg30
TD                65536
SOLVENT           CDCl3
NS                   16
DS                    2
SWH           10000.000 Hz
FIDRES         0.305176 Hz
AQ            3.2767999 sec
RG                102.6
DW               50.000 usec
DE                 6.50 usec
TE                295.1 K
D1           1.00000000 sec
TD0                   1
SFO1        500.1330883 MHz
NUC1                 1H
P1                10.00 usec
PLW1        25.00000000 W

F2 - Processing parameters
SI                65536
SF          500.1300125 MHz
WDW                  EM
SSB                   0
LB                 0.30 Hz
GB                    0
PC                 1.00
```

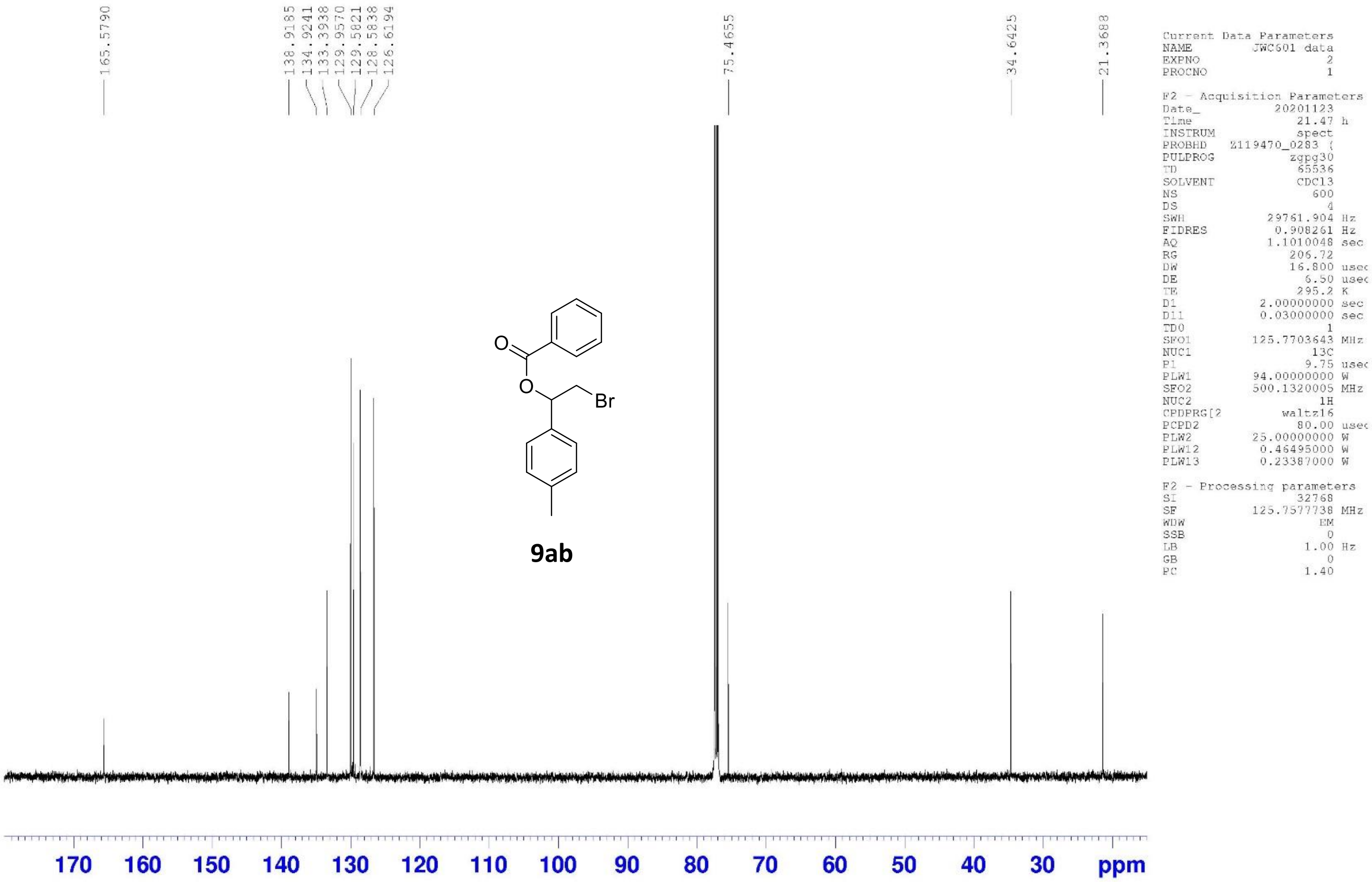

165.5790
138.9185
134.9241
133.3938
129.9570
129.5821
128.5838
126.6194
75.4655
34.6425
21.3688
O
O
Br
9ab
170 160 150 140 130 120 110 100 90 80 70 60 50 40 30 ppm
Current Data Parameters
NAME JWC601 data
EXPNO 2
PROCNO 1
F2 - Acquisition Parameters
Date_ 20201123
Time 21.47 h
INSTRUM spect
PROBHD Z119470_0283 (
PULPROG zgpg30
TD 65536
SOLVENT CDCl3
NS 600
DS 4
SWH 29761.904 Hz
FIDRES 0.908261 Hz
AQ 1.1010048 sec
RG 206.72
DW 16.800 usec
DE 6.50 usec
TE 295.2 K
D1 2.00000000 sec
D11 0.03000000 sec
TD0 1
SFO1 125.7703643 MHz
NUC1 13C
P1 9.75 usec
PLW1 94.00000000 W
SFO2 500.1320005 MHz
NUC2 1H
CPDPRG[2 waltz16
PCPD2 80.00 usec
PLW2 25.00000000 W
PLW12 0.46495000 W
PLW13 0.23387000 W
F2 - Processing parameters
SI 32768
SF 125.7577738 MHz
WDW EM
SSB 0
LB 1.00 Hz
GB 0
PC 1.40

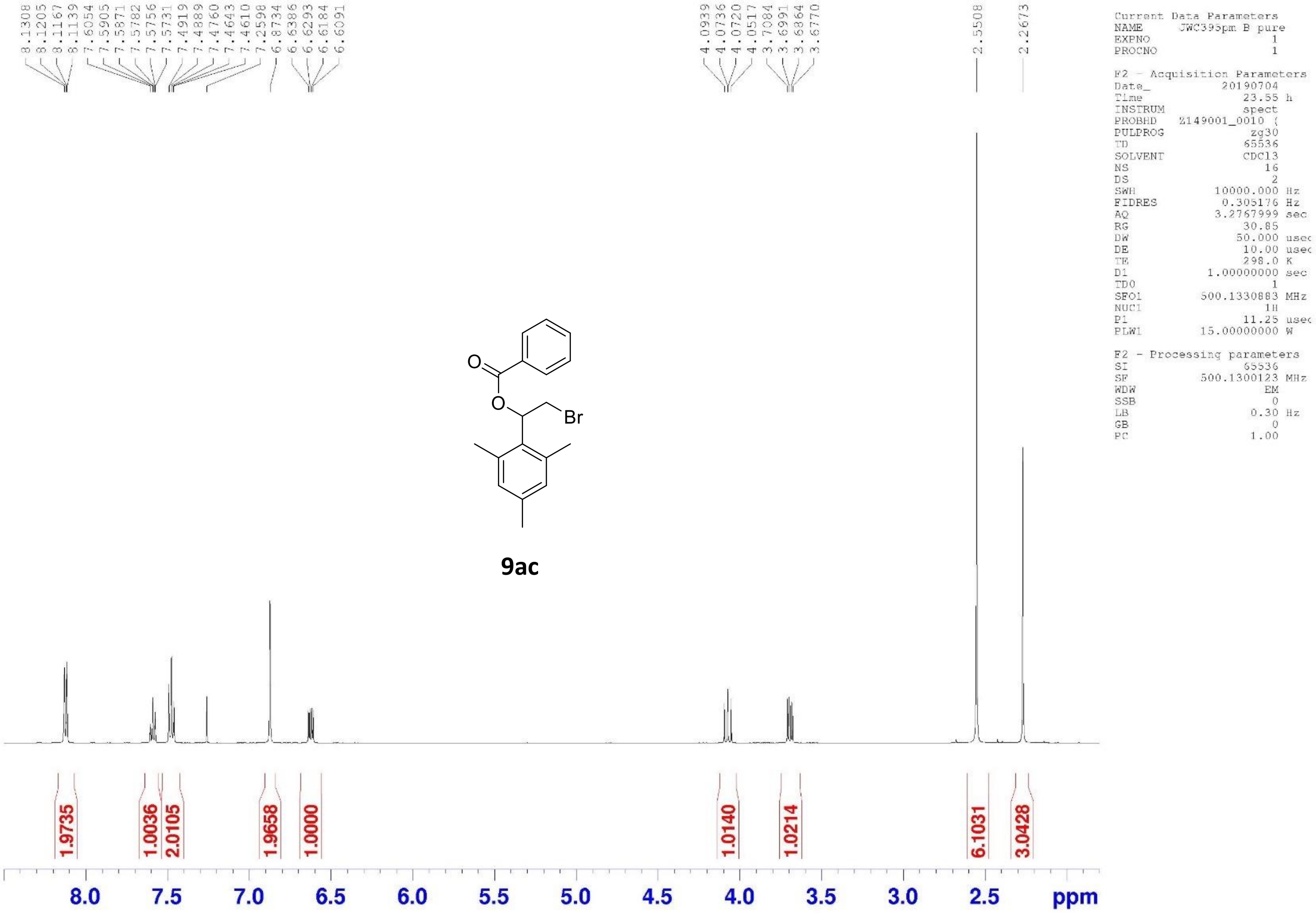
8.1308
8.1205
8.1167
8.1139
7.6054
7.5905
7.5871
7.5782
7.5756
7.5731
7.4919
7.4889
7.4760
7.4643
7.4610
7.2598
6.8734
6.6386
6.6293
6.6184
6.6091
4.0939
4.0736
4.0720
4.0517
3.7084
3.6991
3.6864
3.6770
2.5508
2.2673
O
O
Br
9ac
1.9735
1.0036
2.0105
1.9658
1.0000
1.0140
1.0214
6.1031
3.0428
8.0
7.5
7.0
6.5
6.0
5.5
5.0
4.5
4.0
3.5
3.0
2.5
ppm
Current Data Parameters
NAME JWC395pm B pure
EXPNO 1
PROCNO 1
F2 - Acquisition Parameters
Date_ 20190704
Time 23.55 h
INSTRUM spect
PROBHD Z149001_0010 (
PULPROG zg30
TD 65536
SOLVENT CDCl3
NS 16
DS 2
SWH 10000.000 Hz
FIDRES 0.305176 Hz
AQ 3.2767999 sec
RG 30.85
DW 50.000 usec
DE 10.00 usec
TE 298.0 K
D1 1.00000000 sec
TD0 1
SFO1 500.1330883 MHz
NUC1 1H
P1 11.25 usec
PLW1 15.00000000 W
F2 - Processing parameters
SI 65536
SF 500.1300123 MHz
WDW EM
SSB 0
LB 0.30 Hz
GB 0
PC 1.00

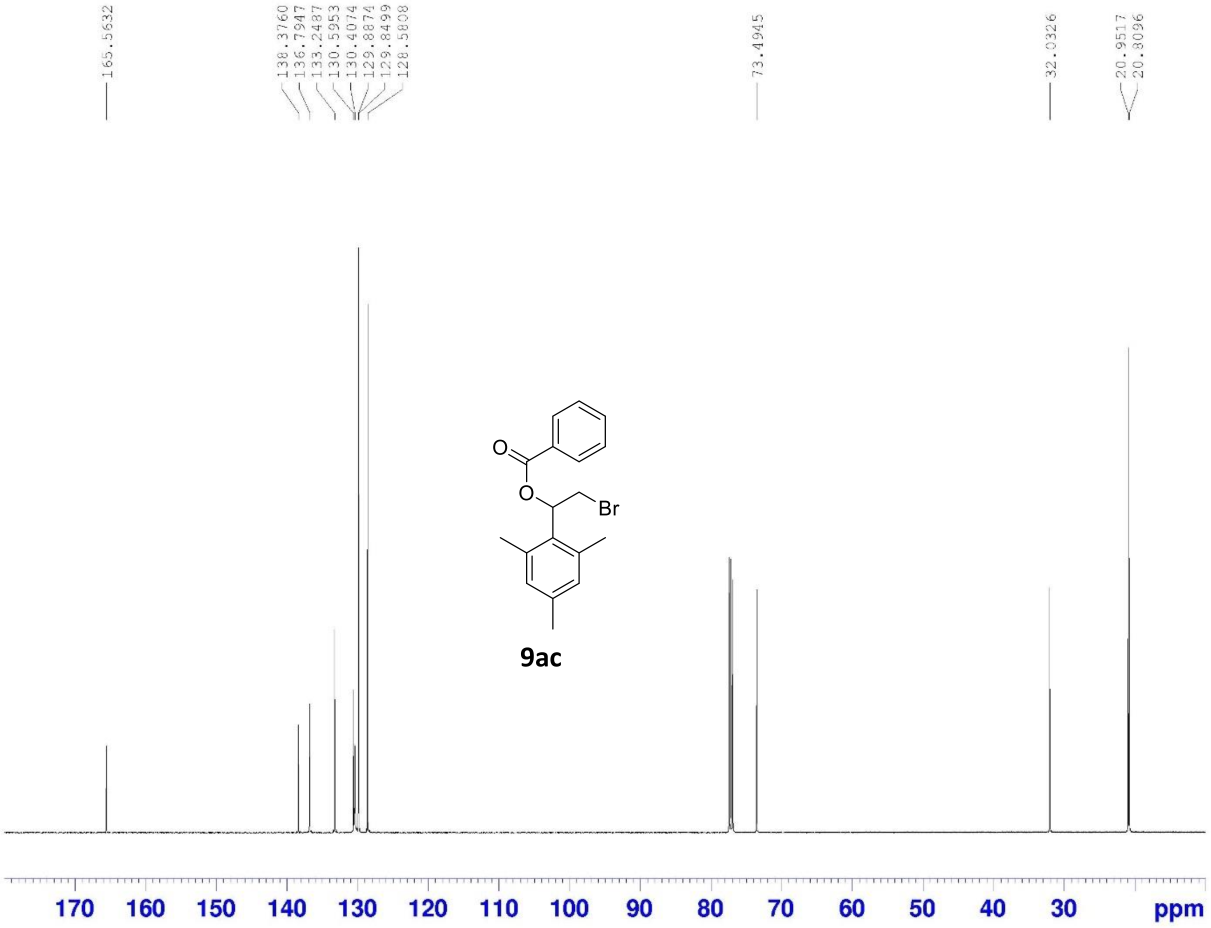

9ac

Current Data Parameters
NAME JWC395pm B pure
EXPNO 2
PROCNO 1

F2 - Acquisition Parameters
Date_ 20190705
Time 0.27 h
INSTRUM spect
PROBHD Z149001_0010 (
PULPROG zgpg30
TD 65536
SOLVENT CDCl3
NS 600
DS 4
SWH 29761.904 Hz
FIDRES 0.908261 Hz
AQ 1.1010048 sec
RG 206.72
DW 16.800 usec
DE 18.00 usec
TE 298.0 K
D1 2.00000000 sec
D11 0.03000000 sec
TD0 1
SFO1 125.7703643 MHz
NUC1 13C
P1 10.00 usec
PLW1 61.00000000 W
SFO2 500.1320005 MHz
NUC2 1H
CPDPRG[2 waltz16
PCPD2 80.00 usec
PLW2 15.00000000 W
PLW12 0.29663000 W
PLW13 0.14920001 W

F2 - Processing parameters
SI 32768
SF 125.7577815 MHz
WDW EM
SSB 0
LB 1.00 Hz
GB 0
PC 1.40

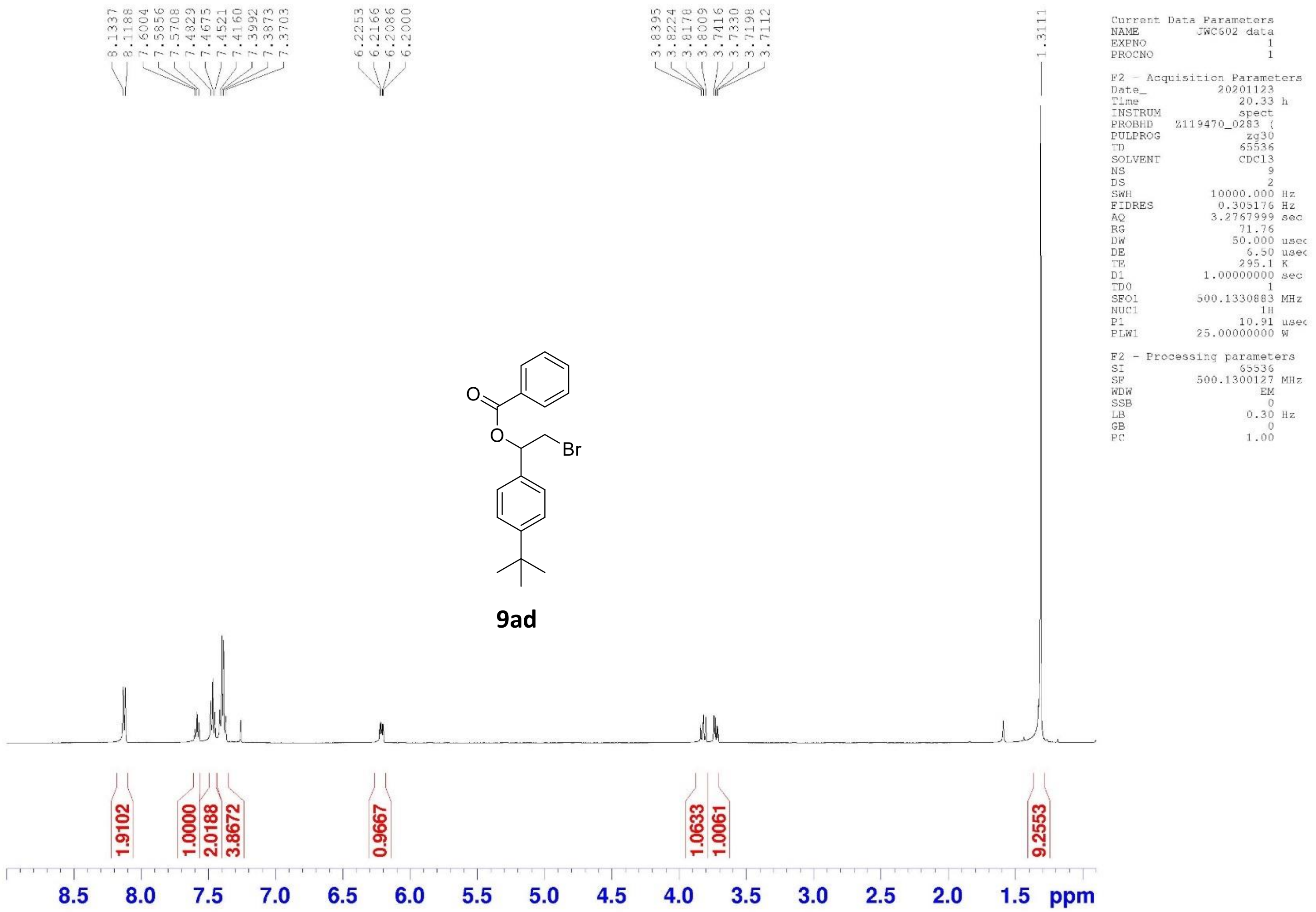
8.1337
8.1188
7.6004
7.5856
7.5708
7.4829
7.4675
7.4521
7.4160
7.3992
7.3873
7.3703
6.2253
6.2166
6.2086
6.2000
3.8395
3.8224
3.8178
3.8009
3.7416
3.7330
3.7198
3.7112
1.3111
1.9102
1.0000
2.0188
3.8672
0.9667
1.0633
1.0061
9.2553
8.5
8.0
7.5
7.0
6.5
6.0
5.5
5.0
4.5
4.0
3.5
3.0
2.5
2.0
1.5
ppm
O
O
Br
Current Data Parameters
NAME JWC602 data
EXPNO 1
PROCNO 1
F2 - Acquisition Parameters
Date_ 20201123
Time 20.33 h
INSTRUM spect
PROBHD Z119470_0283 (
PULPROG zg30
TD 65536
SOLVENT CDCl3
NS 9
DS 2
SWH 10000.000 Hz
FIDRES 0.305176 Hz
AQ 3.2767999 sec
RG 71.76
DW 50.000 usec
DE 6.50 usec
TE 295.1 K
D1 1.00000000 sec
TD0 1
SFO1 500.1330883 MHz
NUC1 1H
P1 10.91 usec
PLW1 25.00000000 W
F2 - Processing parameters
SI 65536
SF 500.1300127 MHz
WDW EM
SSB 0
LB 0.30 Hz
GB 0
PC 1.00

9ad

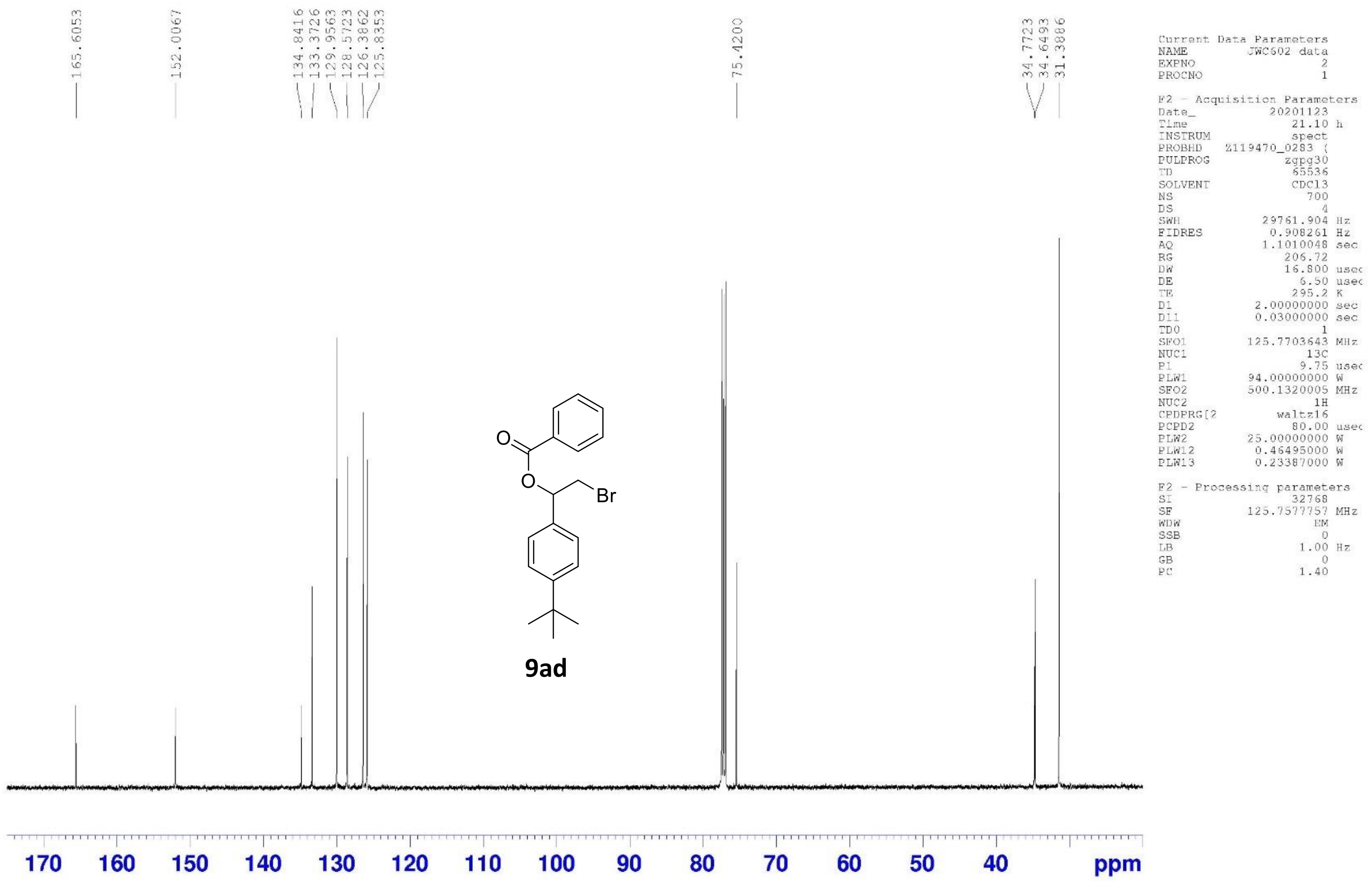
165.6053
152.0067
134.8416
133.3726
129.9563
128.5723
126.3862
125.8353
75.4200
34.7723
34.6493
31.3886
O
O
Br
9ad
170 160 150 140 130 120 110 100 90 80 70 60 50 40 ppm
Current Data Parameters
NAME JWC602 data
EXPNO 2
PROCNO 1
F2 - Acquisition Parameters
Date_ 20201123
Time 21.10 h
INSTRUM spect
PROBHD Z119470_0283 (
PULPROG zgpg30
TD 65536
SOLVENT CDCl3
NS 700
DS 4
SWH 29761.904 Hz
FIDRES 0.908261 Hz
AQ 1.1010048 sec
RG 206.72
DW 16.800 usec
DE 6.50 usec
TE 295.2 K
D1 2.00000000 sec
D11 0.03000000 sec
TD0 1
SFO1 125.7703643 MHz
NUC1 13C
P1 9.75 usec
PLW1 94.00000000 W
SFO2 500.1320005 MHz
NUC2 1H
CPDPRG[2 waltz16
PCPD2 80.00 usec
PLW2 25.00000000 W
PLW12 0.46495000 W
PLW13 0.23387000 W
F2 - Processing parameters
SI 32768
SF 125.7577757 MHz
WDW EM
SSB 0
LB 1.00 Hz
GB 0
PC 1.40

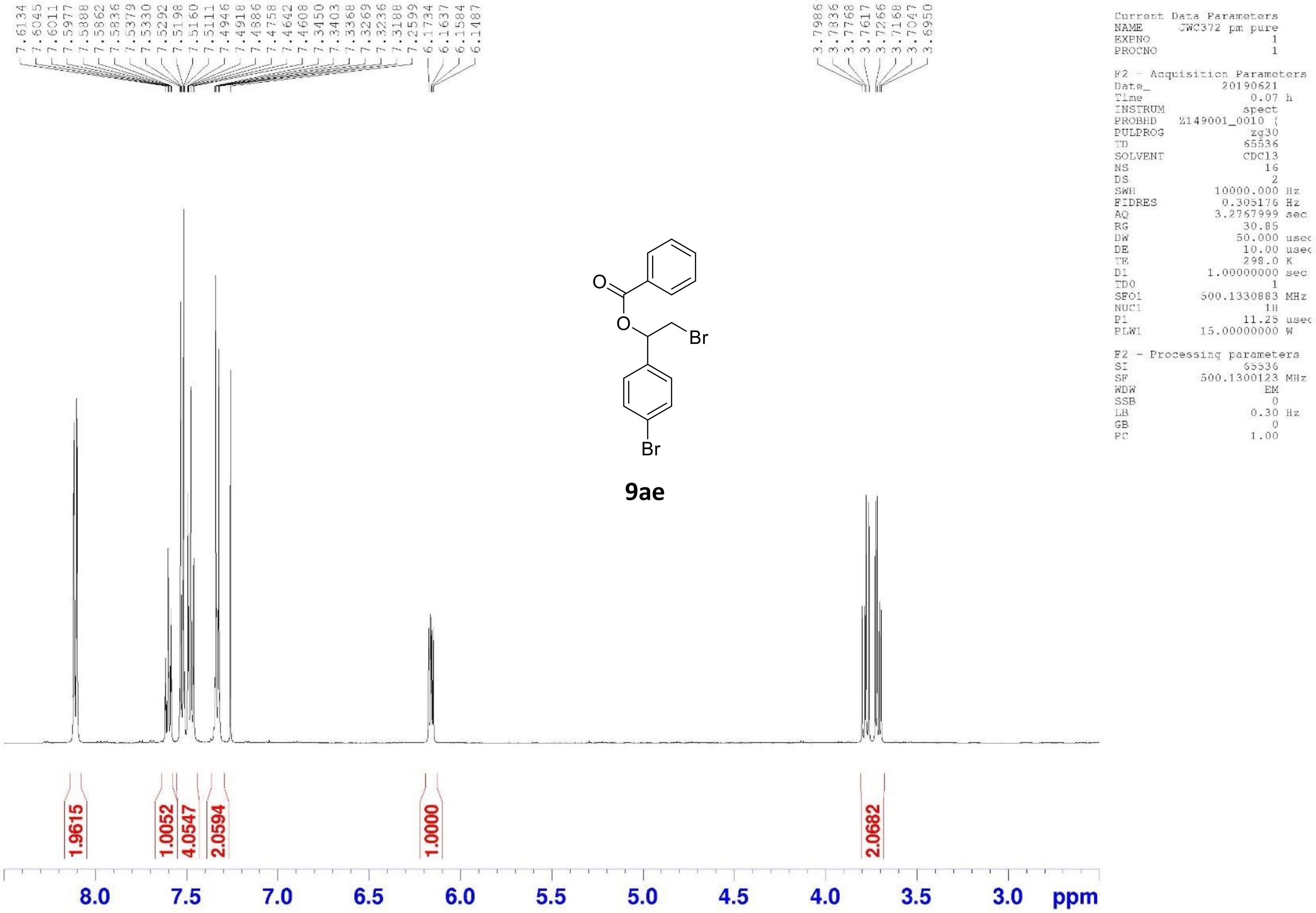
7.6134
7.6045
7.6011
7.5977
7.5888
7.5862
7.5836
7.5379
7.5330
7.5292
7.5198
7.5160
7.5111
7.4946
7.4918
7.4886
7.4758
7.4642
7.4608
7.3450
7.3403
7.3368
7.3269
7.3236
7.3188
7.2599
6.1734
6.1637
6.1584
6.1487
3.7986
3.7836
3.7768
3.7617
3.7266
3.7168
3.7047
3.6950
O
O
Br
Br
9ae
1.9615
1.0052
4.0547
2.0594
1.0000
2.0682
8.0
7.5
7.0
6.5
6.0
5.5
5.0
4.5
4.0
3.5
3.0
ppm
Current Data Parameters
NAME JWC372 pm pure
EXPNO 1
PROCNO 1
F2 - Acquisition Parameters
Date_ 20190621
Time 0.07 h
INSTRUM spect
PROBHD Z149001_0010 (
PULPROG zg30
TD 65536
SOLVENT CDCl3
NS 16
DS 2
SWH 10000.000 Hz
FIDRES 0.305176 Hz
AQ 3.2767999 sec
RG 30.85
DW 50.000 usec
DE 10.00 usec
TE 298.0 K
D1 1.00000000 sec
TD0 1
SFO1 500.1330883 MHz
NUC1 1H
P1 11.25 usec
PLW1 15.00000000 W
F2 - Processing parameters
SI 65536
SF 500.1300123 MHz
WDW EM
SSB 0
LB 0.30 Hz
GB 0
PC 1.00

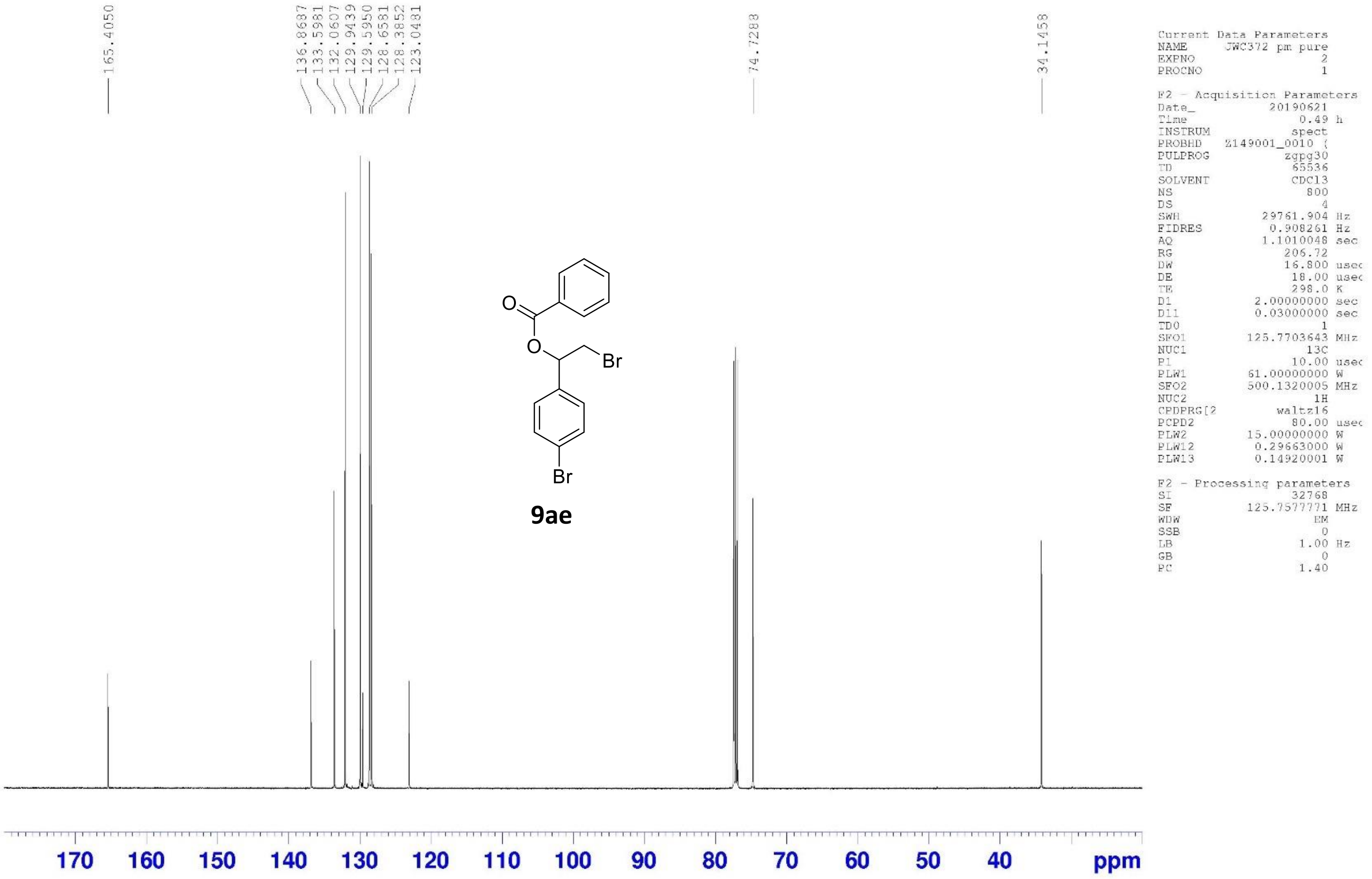

165.4050
136.8637
133.5981
132.0607
129.9439
129.5950
128.6581
128.3852
123.0431
74.7283
34.1458
O
O
Br
Br
9ae
170 160 150 140 130 120 110 100 90 80 70 60 50 40 ppm
Current Data Parameters
NAME JWC372 pm pure
EXPNO 2
PROCNO 1
F2 - Acquisition Parameters
Date_ 20190621
Time 0.49 h
INSTRUM spect
PROBHD Z149001_0010 (
PULPROG zgpg30
TD 65536
SOLVENT CDCl3
NS 800
DS 4
SWH 29761.904 Hz
FIDRES 0.908261 Hz
AQ 1.1010048 sec
RG 206.72
DW 16.800 usec
DE 18.00 usec
TE 298.0 K
D1 2.00000000 sec
D11 0.03000000 sec
TD0 1
SFO1 125.7703643 MHz
NUC1 13C
P1 10.00 usec
PLW1 61.00000000 W
SFO2 500.1320005 MHz
NUC2 1H
CPDPRG[2 waltz16
PCPD2 80.00 usec
PLW2 15.00000000 W
PLW12 0.29663000 W
PLW13 0.14920001 W
F2 - Processing parameters
SI 32768
SF 125.7577771 MHz
WDW EM
SSB 0
LB 1.00 Hz
GB 0
PC 1.40

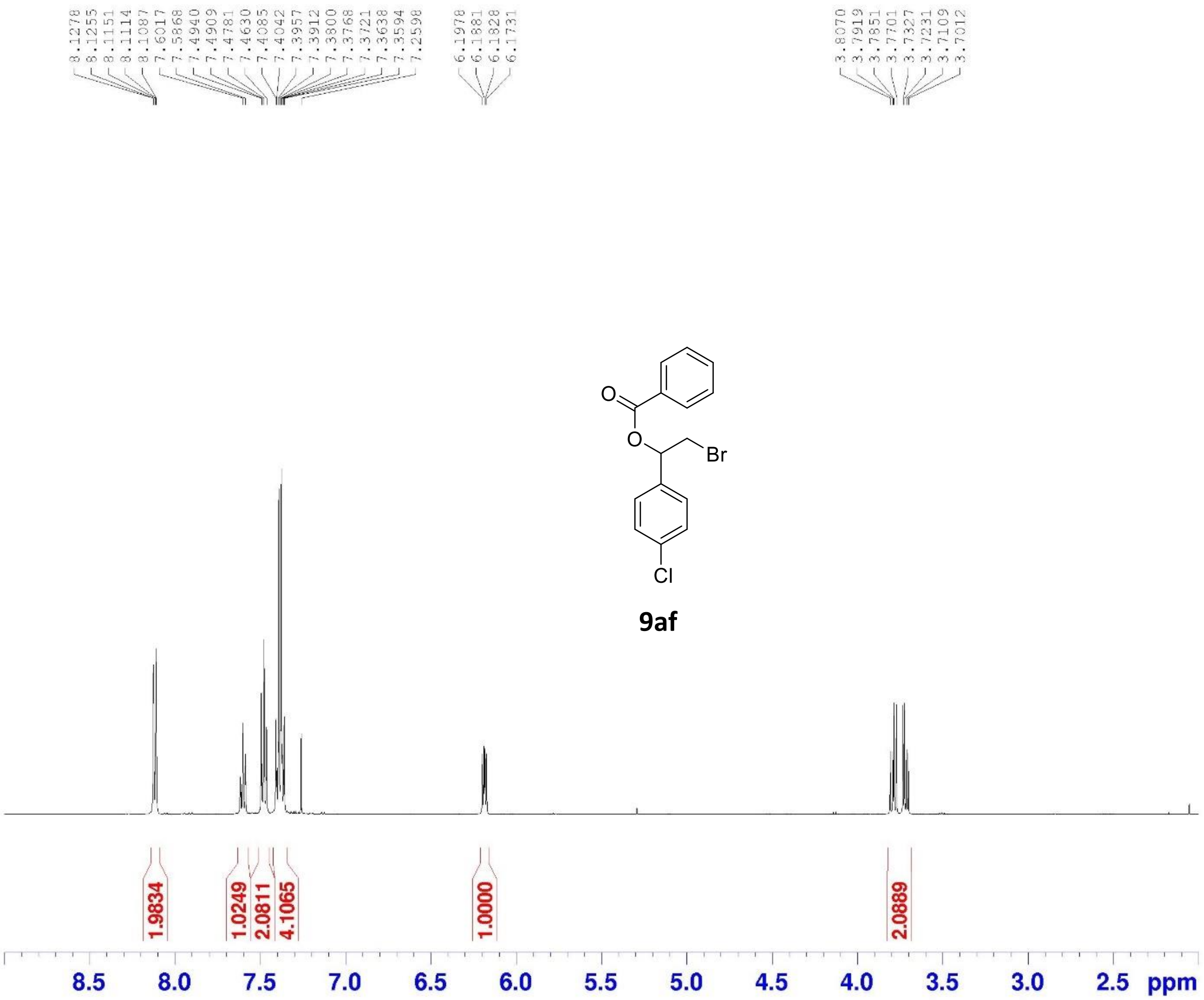

9af

Current Data Parameters		
NAME	JWC370 pm pure	
EXPNO	1	
PROCNO	1	
F2 - Acquisition Parameters		
Date_	20190620	
Time	23.21	h
INSTRUM	spect	
PROBHD	Z149001_0010 (	
PULPROG	zg30	
TD	65536	
SOLVENT	CDCl3	
NS	16	
DS	2	
SWH	10000.000	Hz
FIDRES	0.305176	Hz
AQ	3.2767999	sec
RG	30.85	
DW	50.000	usec
DE	10.00	usec
TE	298.0	K
D1	1.00000000	sec
TD0	1	
SFO1	500.1330883	MHz
NUC1	1H	
P1	11.25	usec
PLW1	15.00000000	W
F2 - Processing parameters		
SI	65536	
SF	500.1300123	MHz
WDW	EM	
SSB	0	
LB	0.30	Hz
GB	0	
PC	1.00	

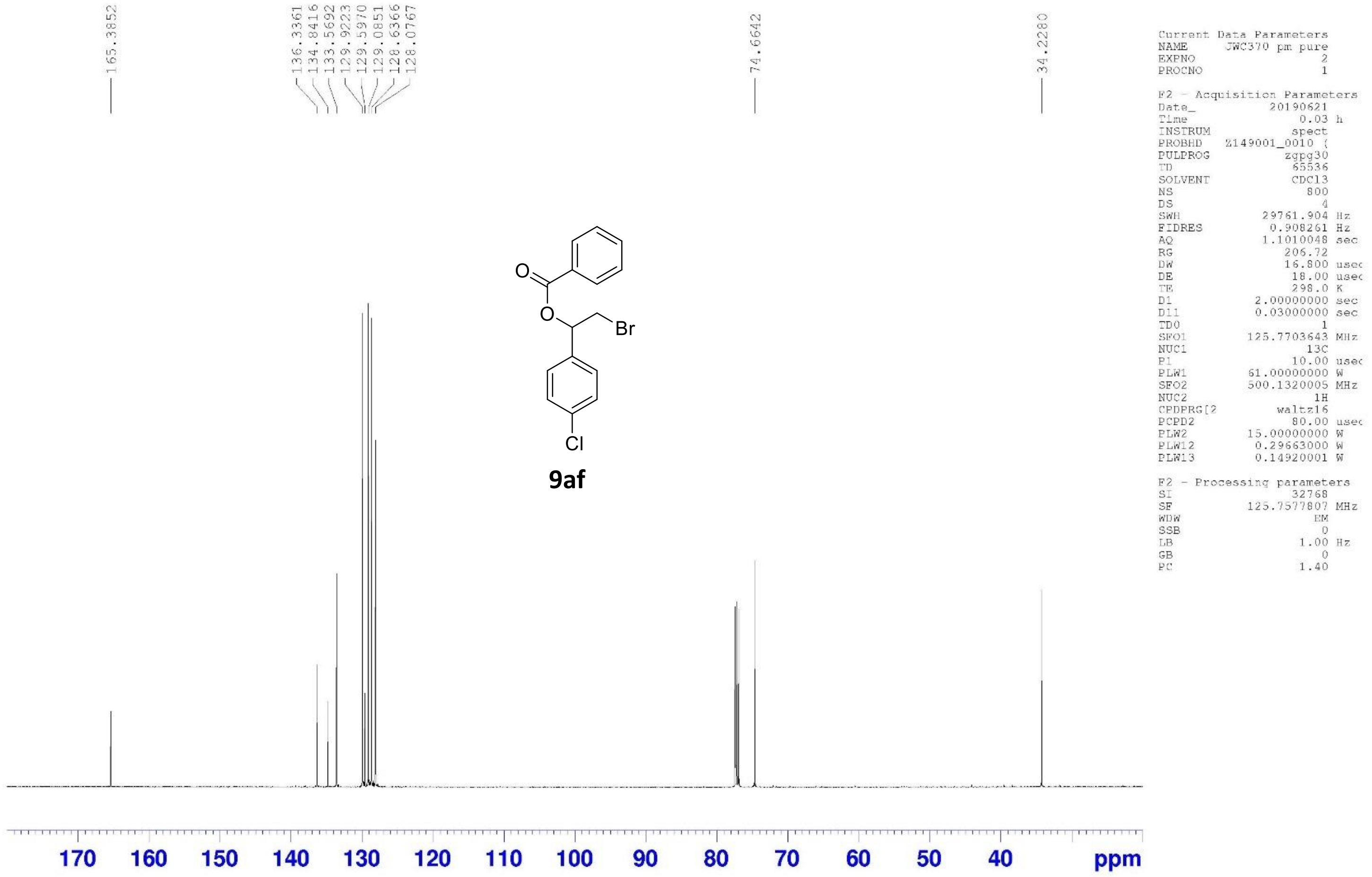
165.3852
136.3361
134.8416
133.5692
129.9223
129.5970
129.0851
128.6366
128.0767
74.6642
34.2280
O
O
Br
Cl
9af
170 160 150 140 130 120 110 100 90 80 70 60 50 40 ppm
Current Data Parameters
NAME JWC370 pm pure
EXPNO 2
PROCNO 1
F2 - Acquisition Parameters
Date_ 20190621
Time 0.03 h
INSTRUM spect
PROBHD Z149001_0010 (
PULPROG zgpg30
TD 65536
SOLVENT CDCl3
NS 800
DS 4
SWH 29761.904 Hz
FIDRES 0.908261 Hz
AQ 1.1010048 sec
RG 206.72
DW 16.800 usec
DE 18.00 usec
TE 298.0 K
D1 2.00000000 sec
D11 0.03000000 sec
TD0 1
SFO1 125.7703643 MHz
NUC1 13C
P1 10.00 usec
PLW1 61.00000000 W
SFO2 500.1320005 MHz
NUC2 1H
CPDPRG[2 waltz16
PCPD2 80.00 usec
PLW2 15.00000000 W
PLW12 0.29663000 W
PLW13 0.14920001 W
F2 - Processing parameters
SI 32768
SF 125.7577807 MHz
WDW EM
SSB 0
LB 1.00 Hz
GB 0
PC 1.40

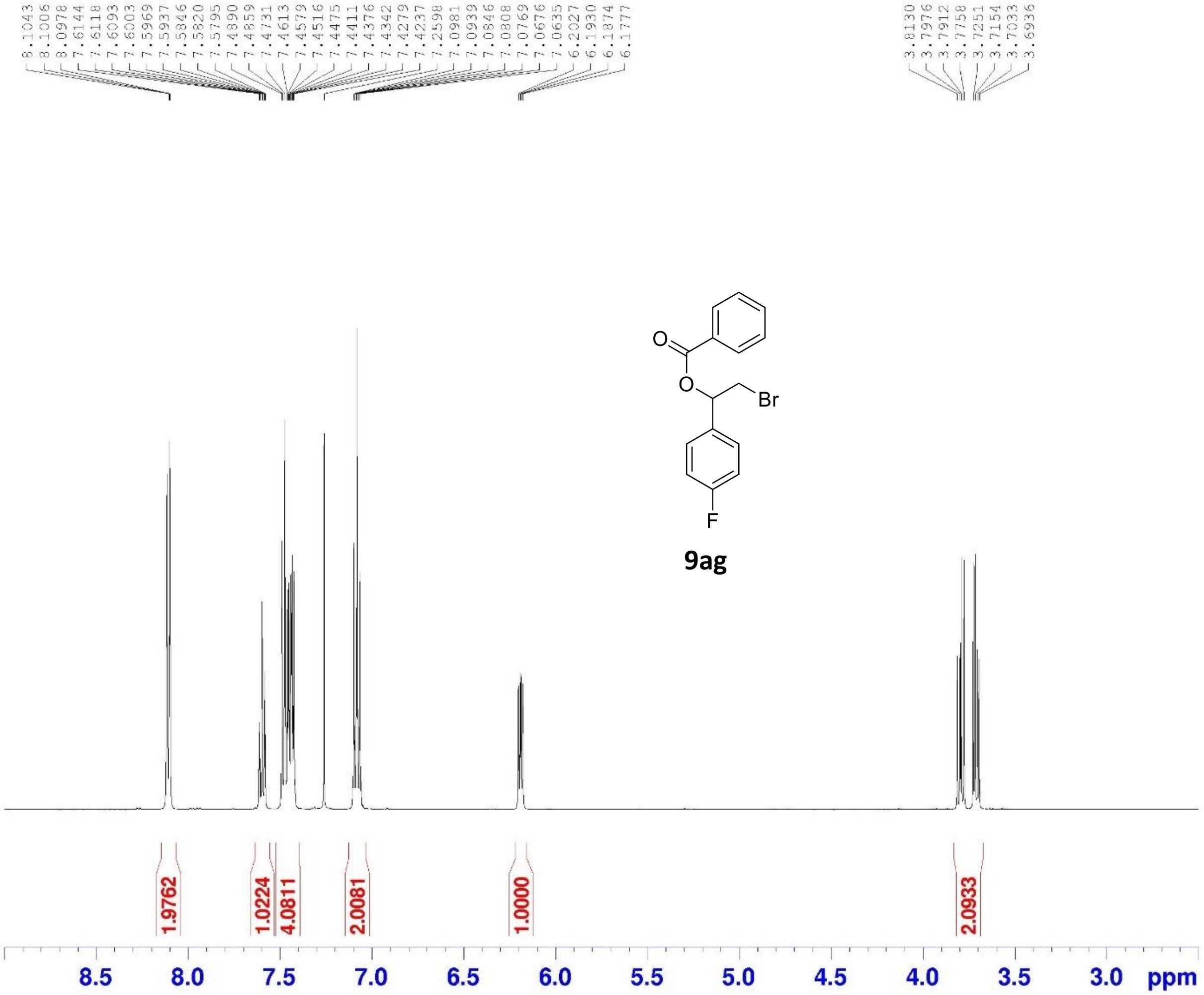

9ag

Current Data Parameters	
NAME	JWC378 pm pure
EXPNO	1
PROCNO	1
F2 - Acquisition Parameters	
Date_	20190621
Time	2.25 h
INSTRUM	spect
PROBHD	Z149001_0010 (
PULPROG	zg30
TD	65536
SOLVENT	CDCl3
NS	16
DS	2
SWH	10000.000 Hz
FIDRES	0.305176 Hz
AQ	3.2767999 sec
RG	50.6
DW	50.000 usec
DE	10.00 usec
TE	298.0 K
D1	1.00000000 sec
TD0	1
SFO1	500.1330883 MHz
NUC1	1H
P1	11.25 usec
PLW1	15.00000000 W
F2 - Processing parameters	
SI	65536
SF	500.1300124 MHz
WDW	EM
SSB	0
LB	0.30 Hz
GB	0
PC	1.00

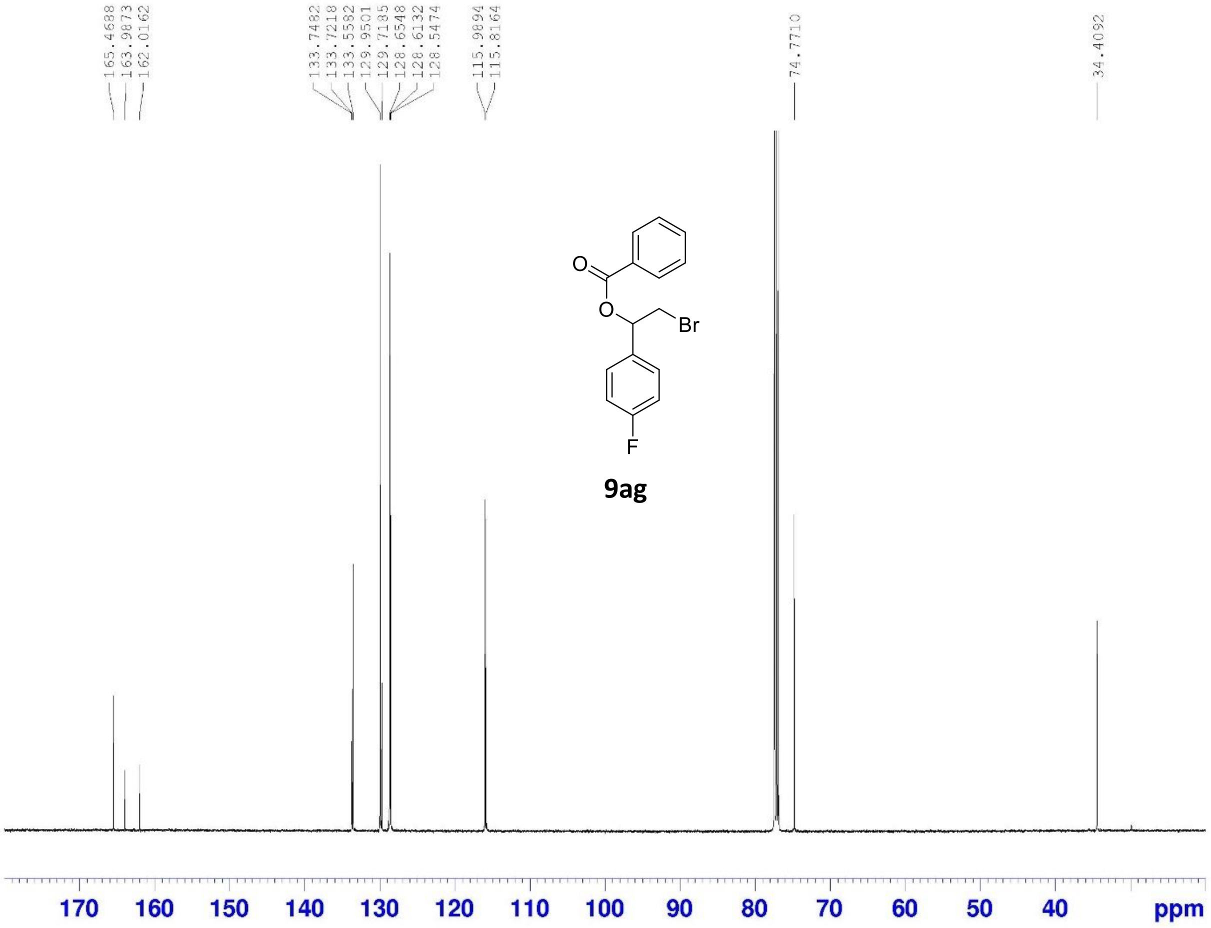

Current Data Parameters
NAME JWC378 pm pure
EXPNO 2
PROCNO 1

F2 - Acquisition Parameters
Date_ 20190621
Time 3.07 h
INSTRUM spect
PROBHD Z149001_0010 (
PULPROG zgpg30
TD 65536
SOLVENT CDCl3
NS 800
DS 4
SWH 29761.904 Hz
FIDRES 0.908261 Hz
AQ 1.1010048 sec
RG 206.72
DW 16.800 usec
DE 18.00 usec
TE 298.0 K
D1 2.00000000 sec
D11 0.03000000 sec
TD0 1
SFO1 125.7703643 MHz
NUC1 13C
P1 10.00 usec
PLW1 61.00000000 W
SFO2 500.1320005 MHz
NUC2 1H
CPDPRG[2 waltz16
PCPD2 80.00 usec
PLW2 15.00000000 W
PLW12 0.29663000 W
PLW13 0.14920001 W

F2 - Processing parameters
SI 32768
SF 125.7577737 MHz
WDW EM
SSB 0
LB 1.00 Hz
GB 0
PC 1.40

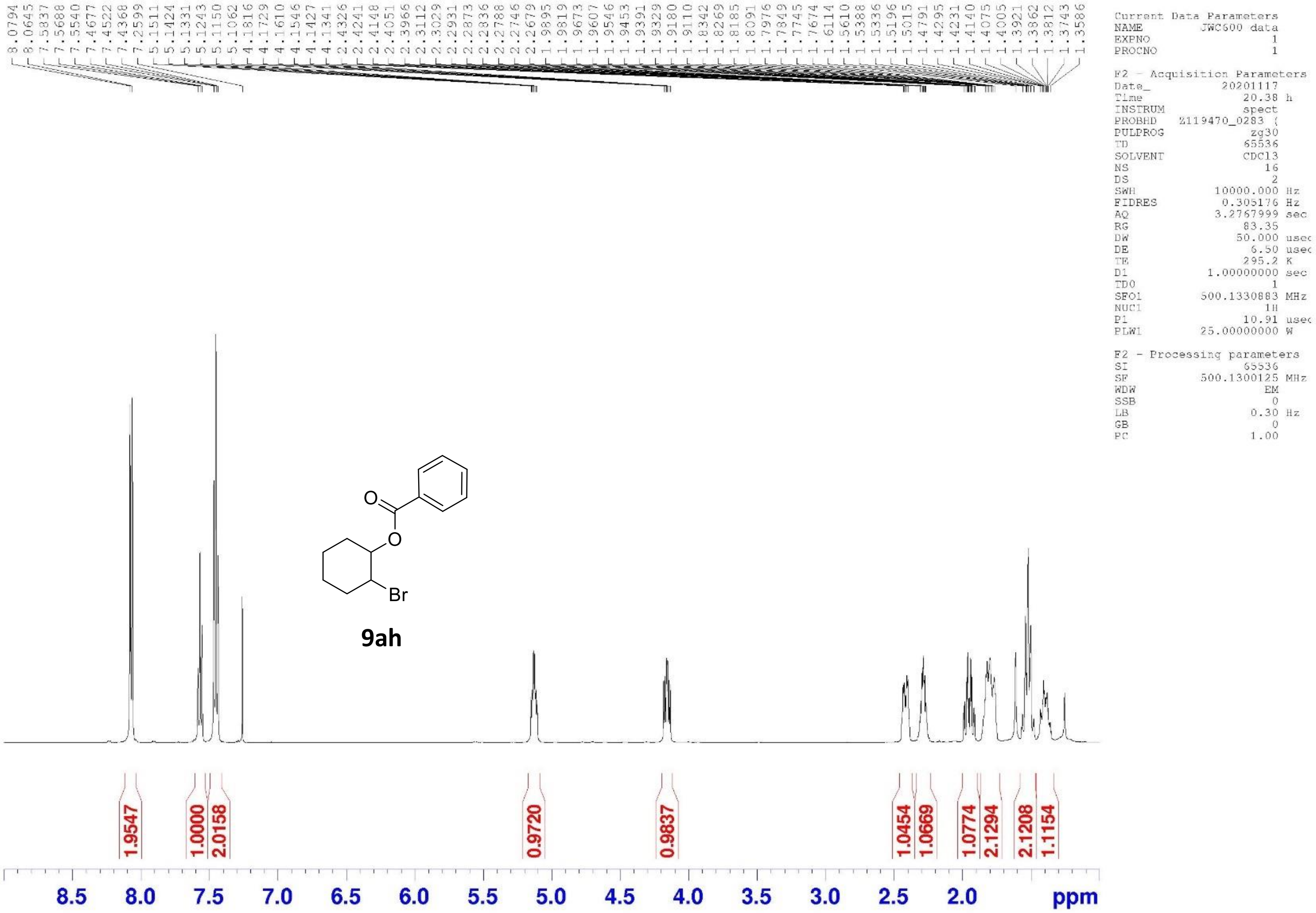
8.0794
8.0645
7.5837
7.5688
7.5540
7.4677
7.4522
7.4368
7.2599
5.1511
5.1424
5.1331
5.1243
5.1150
5.1062
4.1816
4.1729
4.1610
4.1546
4.1427
4.1341
2.4326
2.4241
2.4148
2.4051
2.3966
2.3112
2.3029
2.2931
2.2873
2.2836
2.2788
2.2746
2.2679
1.9895
1.9819
1.9673
1.9607
1.9546
1.9453
1.9391
1.9329
1.9180
1.9110
1.8342
1.8269
1.8185
1.8091
1.7976
1.7849
1.7745
1.7674
1.6114
1.5610
1.5388
1.5336
1.5196
1.5015
1.4791
1.4295
1.4231
1.4140
1.4075
1.4005
1.3921
1.3862
1.3812
1.3743
1.3586
Current Data Parameters
NAME JWC500 data
EXPNO 1
PROCNO 1
F2 - Acquisition Parameters
Date_ 20201117
Time 20.38 h
INSTRUM spect
PROBHD Z119470_0283 (
PULPROG zg30
TD 65536
SOLVENT CDCl3
NS 16
DS 2
SWH 10000.000 Hz
FIDRES 0.305176 Hz
AQ 3.2767999 sec
RG 83.35
DW 50.000 usec
DE 6.50 usec
TE 295.2 K
D1 1.00000000 sec
TD0 1
SFO1 500.1330883 MHz
NUC1 1H
P1 10.91 usec
PLW1 25.00000000 W
F2 - Processing parameters
SI 65536
SF 500.1300125 MHz
WDW EM
SSB 0
LB 0.30 Hz
GB 0
PC 1.00
O
O
Br
9ah
1.9547
1.0000
2.0158
0.9720
0.9837
1.0454
1.0669
1.0774
2.1294
2.1208
1.1154
8.5
8.0
7.5
7.0
6.5
6.0
5.5
5.0
4.5
4.0
3.5
3.0
2.5
2.0
ppm

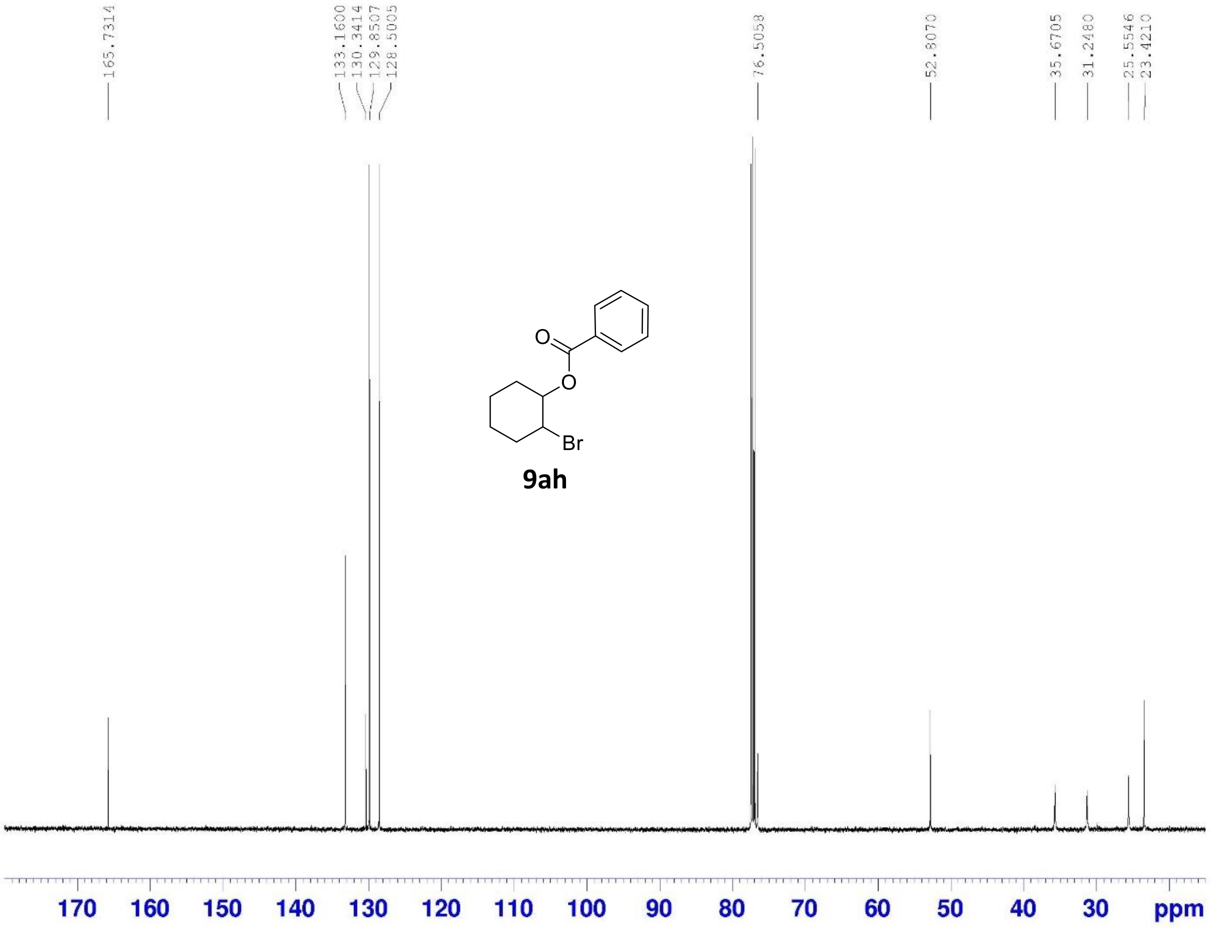

Current Data Parameters		
NAME	JWC600 data	
EXPNO	2	
PROCNO	1	
F2 - Acquisition Parameters		
Date_	20201117	
Time	21.16	h
INSTRUM	spect	
PROBHD	Z119470_0283 (	
PULPROG	zgpg30	
TD	65536	
SOLVENT	CDCl3	
NS	700	
DS	4	
SWH	29761.904	Hz
FIDRES	0.908261	Hz
AQ	1.1010048	sec
RG	206.72	
DW	16.800	usec
DE	6.50	usec
TE	295.1	K
D1	2.00000000	sec
D11	0.03000000	sec
TD0	1	
SFO1	125.7703643	MHz
NUC1	13C	
P1	9.75	usec
PLW1	94.00000000	W
SFO2	500.1320005	MHz
NUC2	1H	
CPDPRG[2	waltz16	
PCPD2	80.00	usec
PLW2	25.00000000	W
PLW12	0.46495000	W
PLW13	0.23387000	W
F2 - Processing parameters		
SI	32768	
SF	125.7577752	MHz
WDW	EM	
SSB	0	
LB	1.00	Hz
GB	0	
PC	1.40	

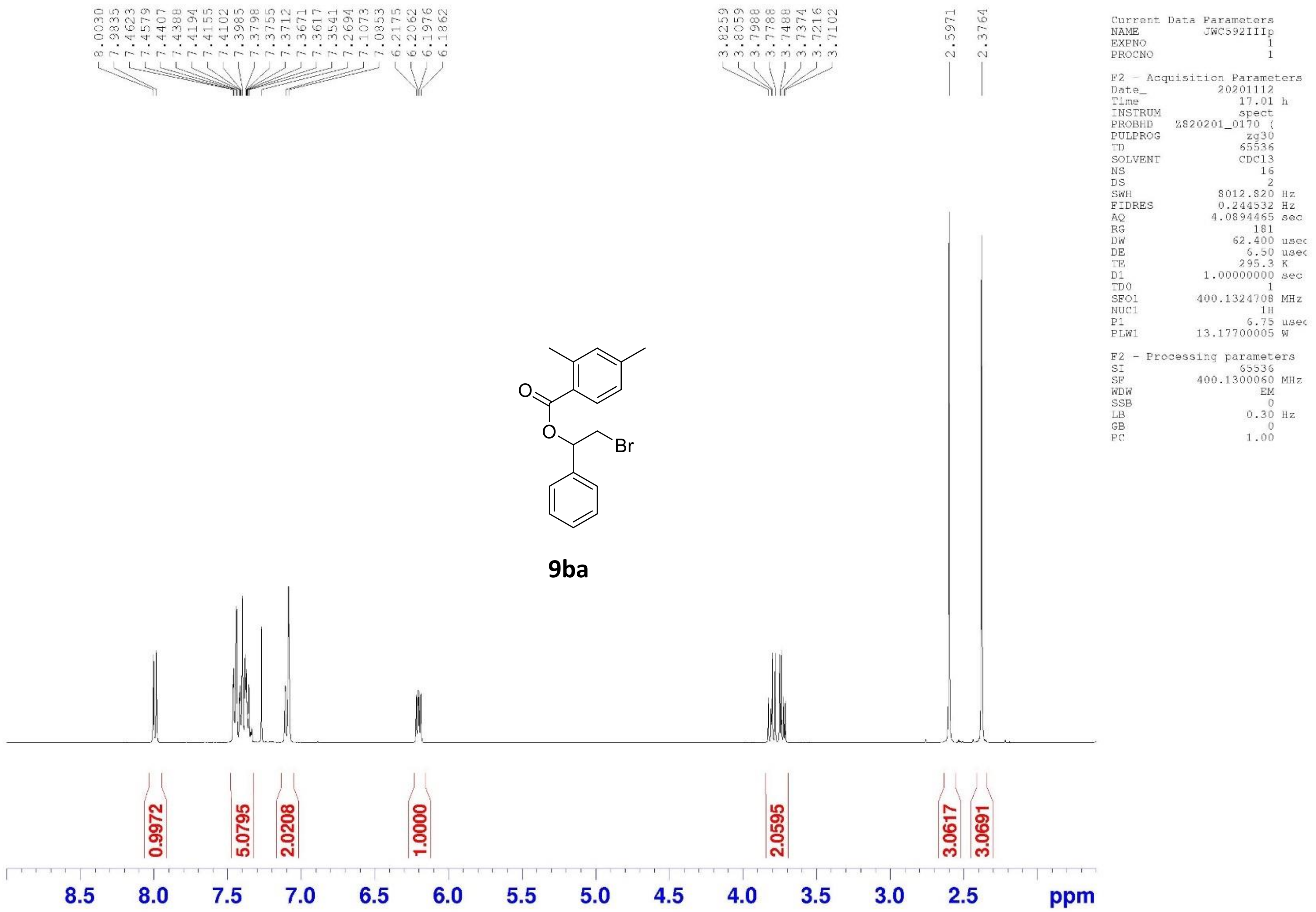
8.0030
7.9835
7.4623
7.4579
7.4407
7.4388
7.4194
7.4155
7.4102
7.3985
7.3798
7.3755
7.3712
7.3671
7.3617
7.3541
7.2694
7.1073
7.0853
6.2175
6.2062
6.1976
6.1862
3.8259
3.8059
3.7988
3.7788
3.7488
3.7374
3.7216
3.7102
2.5971
2.3764
O
O
Br
0.9972
5.0795
2.0208
1.0000
2.0595
3.0617
3.0691
8.5
8.0
7.5
7.0
6.5
6.0
5.5
5.0
4.5
4.0
3.5
3.0
2.5
ppm
Current Data Parameters
NAME JWC592IIIp
EXPNO 1
PROCNO 1
F2 - Acquisition Parameters
Date_ 20201112
Time 17.01 h
INSTRUM spect
PROBHD Z820201_0170 (
PULPROG zg30
TD 65536
SOLVENT CDCl3
NS 16
DS 2
SWH 8012.820 Hz
FIDRES 0.244532 Hz
AQ 4.0894465 sec
RG 181
DW 62.400 usec
DE 6.50 usec
TE 295.3 K
D1 1.00000000 sec
TD0 1
SFO1 400.1324708 MHz
NUC1 1H
P1 6.75 usec
PLW1 13.17700005 W
F2 - Processing parameters
SI 65536
SF 400.1300060 MHz
WDW EM
SSB 0
LB 0.30 Hz
GB 0
PC 1.00

9ba

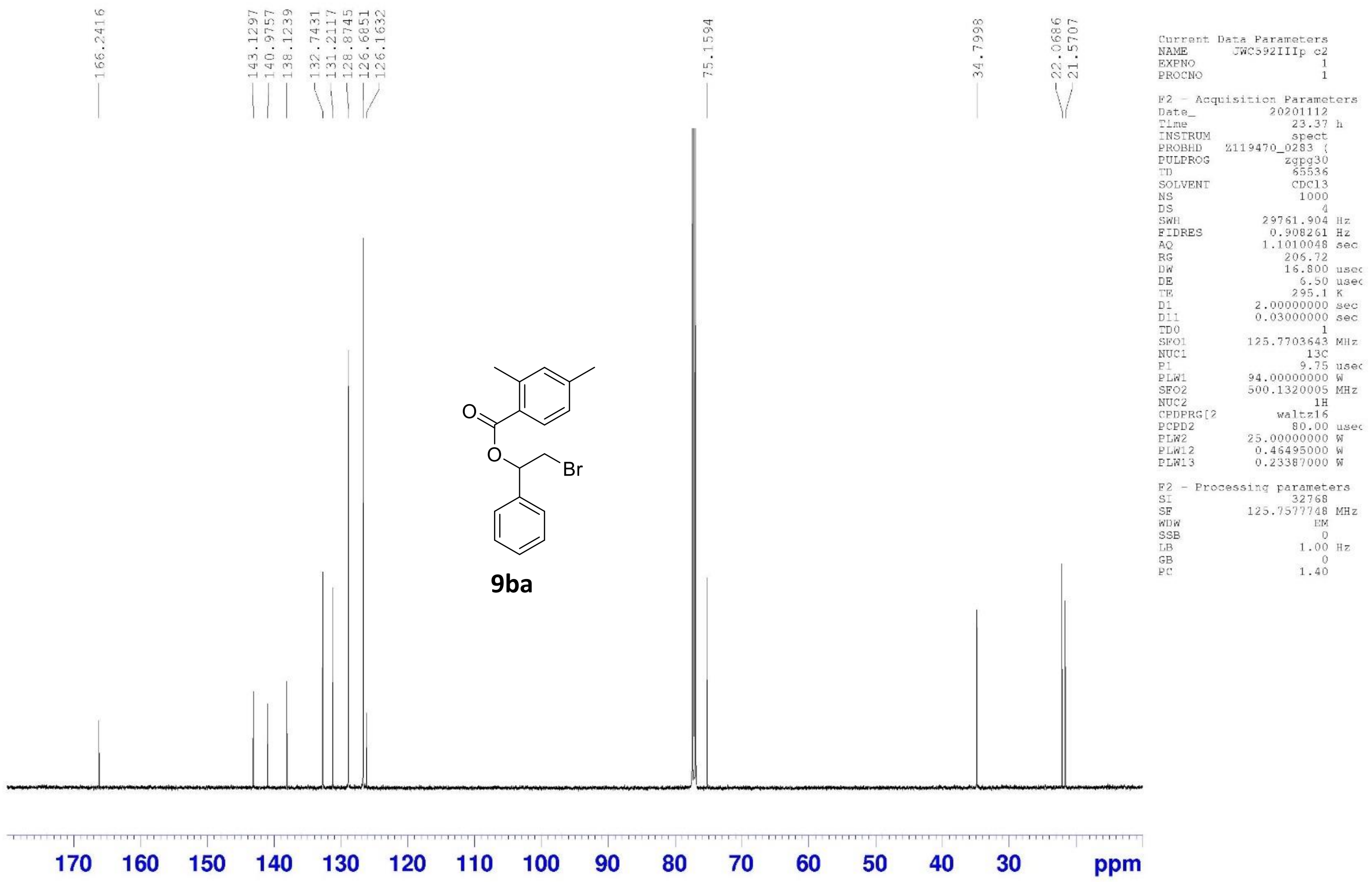
166.2416
143.1297
140.9757
138.1239
132.7431
131.2117
128.8745
126.6851
126.1632
75.1594
34.7998
22.0686
21.5707
O
O
Br
9ba
170 160 150 140 130 120 110 100 90 80 70 60 50 40 30 ppm
Current Data Parameters
NAME JWC592IIIp c2
EXPNO 1
PROCNO 1
F2 - Acquisition Parameters
Date_ 20201112
Time 23.37 h
INSTRUM spect
PROBHD Z119470_0283 (
PULPROG zgpg30
TD 65536
SOLVENT CDCl3
NS 1000
DS 4
SWH 29761.904 Hz
FIDRES 0.908261 Hz
AQ 1.1010048 sec
RG 206.72
DW 16.800 usec
DE 6.50 usec
TE 295.1 K
D1 2.00000000 sec
D11 0.03000000 sec
TD0 1
SFO1 125.7703643 MHz
NUC1 13C
P1 9.75 usec
PLW1 94.00000000 W
SFO2 500.1320005 MHz
NUC2 1H
CPDPRG[2 waltz16
PCPD2 80.00 usec
PLW2 25.00000000 W
PLW12 0.46495000 W
PLW13 0.23387000 W
F2 - Processing parameters
SI 32768
SF 125.7577748 MHz
WDW EM
SSB 0
LB 1.00 Hz
GB 0
PC 1.40

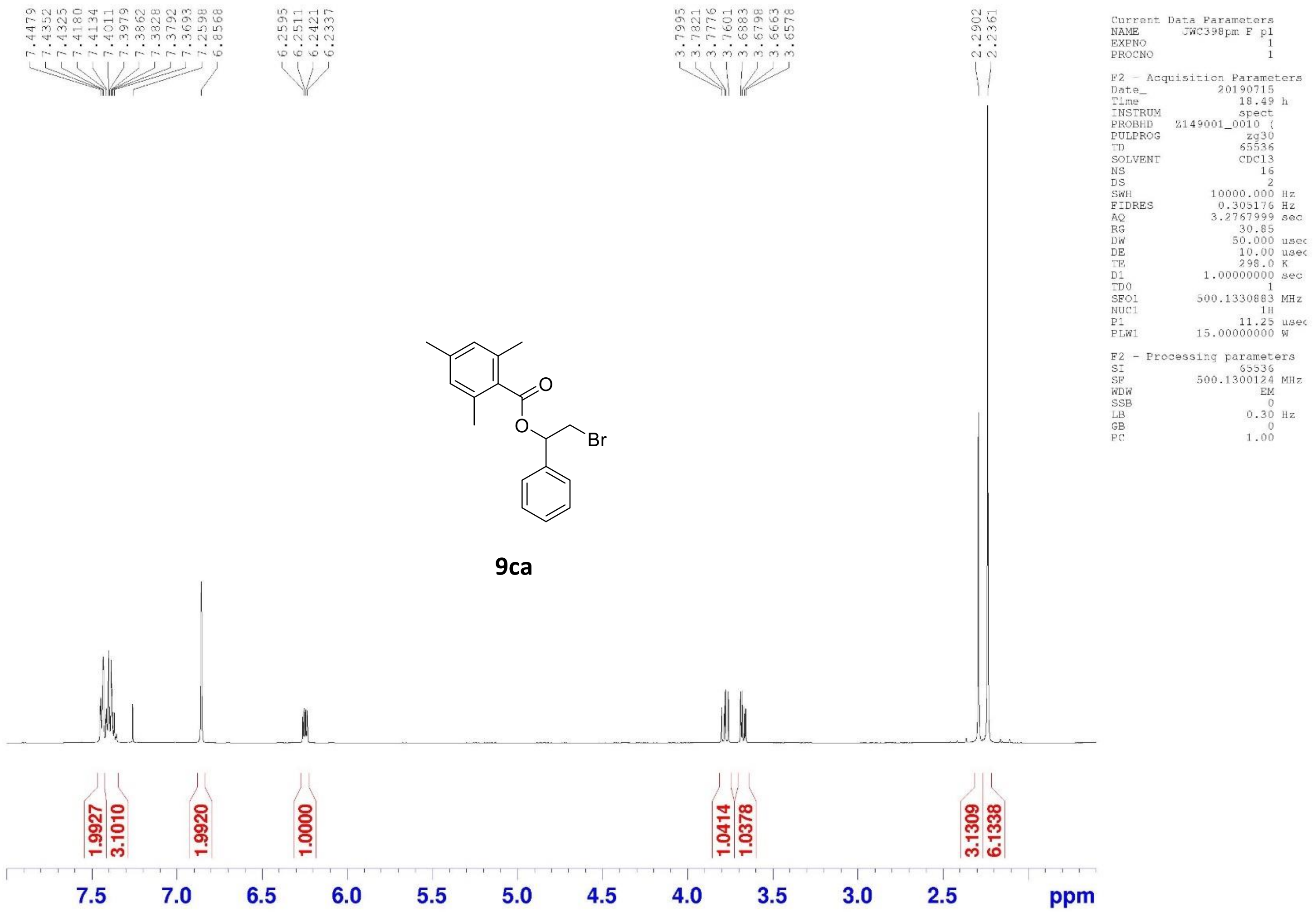

9ca

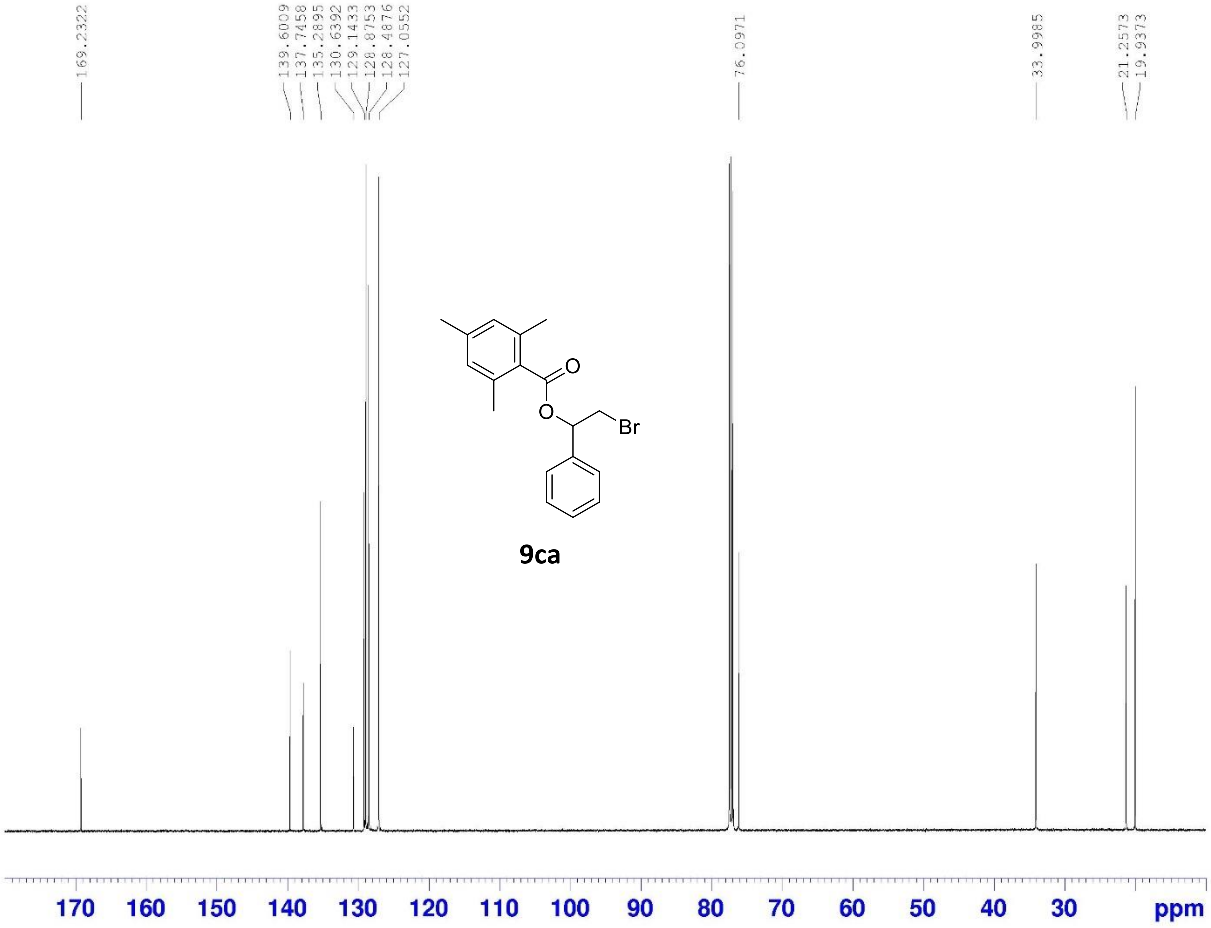

9ca

Current Data Parameters
NAME JWC398pm F p1
EXPNO 2
PROCNO 1

F2 - Acquisition Parameters
Date_ 20190715
Time 19.18 h
INSTRUM spect
PROBHD Z149001_0010 (
PULPROG zgpg30
TD 65536
SOLVENT CDCl3
NS 500
DS 4
SWH 29761.904 Hz
FIDRES 0.908261 Hz
AQ 1.1010048 sec
RG 206.72
DW 16.800 usec
DE 18.00 usec
TE 298.0 K
D1 2.00000000 sec
D11 0.03000000 sec
TD0 1
SFO1 125.7703643 MHz
NUC1 13C
P1 10.00 usec
PLW1 61.00000000 W
SFO2 500.1320005 MHz
NUC2 1H
CPDPRG[2 waltz16
PCPD2 80.00 usec
PLW2 15.00000000 W
PLW12 0.29663000 W
PLW13 0.14920001 W

F2 - Processing parameters
SI 32768
SF 125.7577761 MHz
WDW EM
SSB 0
LB 1.00 Hz
GB 0
PC 1.40

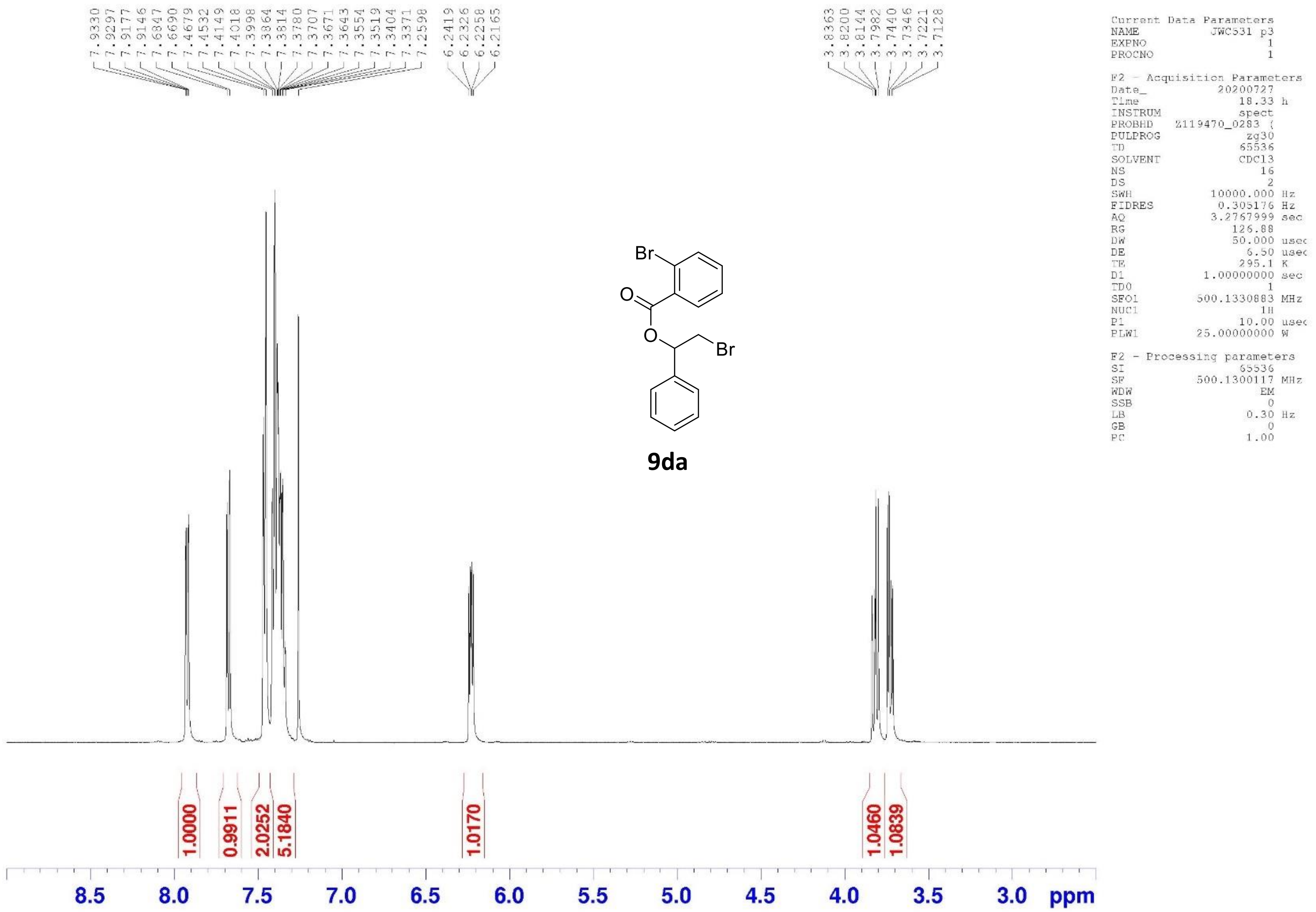
7.9330
7.9297
7.9177
7.9146
7.6847
7.6690
7.4679
7.4532
7.4149
7.4018
7.3998
7.3864
7.3814
7.3780
7.3707
7.3671
7.3643
7.3554
7.3519
7.3404
7.3371
7.2598
6.2419
6.2326
6.2258
6.2165
3.8363
3.8200
3.8144
3.7982
3.7440
3.7346
3.7221
3.7128
Current Data Parameters
NAME JWC531 p3
EXPNO 1
PROCNO 1
F2 - Acquisition Parameters
Date_ 20200727
Time 18.33 h
INSTRUM spect
PROBHD Z119470_0283 (
PULPROG zg30
TD 65536
SOLVENT CDCl3
NS 16
DS 2
SWH 10000.000 Hz
FIDRES 0.305176 Hz
AQ 3.2767999 sec
RG 126.88
DW 50.000 usec
DE 6.50 usec
TE 295.1 K
D1 1.00000000 sec
TD0 1
SFO1 500.1330883 MHz
NUC1 1H
P1 10.00 usec
PLW1 25.00000000 W
F2 - Processing parameters
SI 65536
SF 500.1300117 MHz
WDW EM
SSB 0
LB 0.30 Hz
GB 0
PC 1.00
Br
O
O
Br
9da
1.0000
0.9911
2.0252
5.1840
1.0170
1.0460
1.0839
8.5
8.0
7.5
7.0
6.5
6.0
5.5
5.0
4.5
4.0
3.5
3.0
ppm

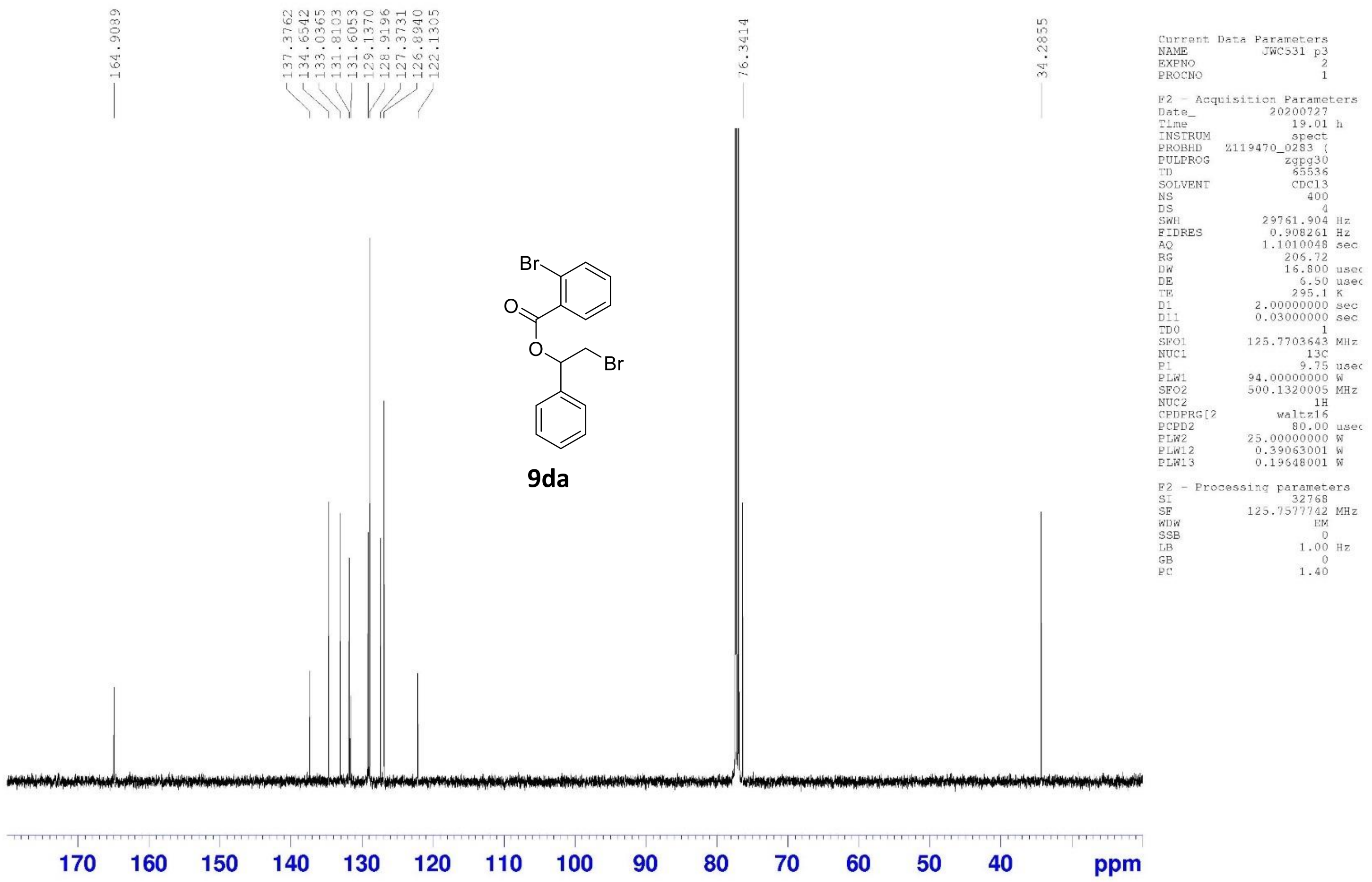
164.9089
137.3762
134.6542
133.0365
131.8103
131.6053
129.1370
128.9196
127.3731
126.8940
122.1305
76.3414
34.2855
Br
O
O
Br
Current Data Parameters
NAME JWC531 p3
EXPNO 2
PROCNO 1
F2 - Acquisition Parameters
Date_ 20200727
Time 19.01 h
INSTRUM spect
PROBHD Z119470_0283 (
PULPROG zgpg30
TD 65536
SOLVENT CDCl3
NS 400
DS 4
SWH 29761.904 Hz
FIDRES 0.908261 Hz
AQ 1.1010048 sec
RG 206.72
DW 16.800 usec
DE 6.50 usec
TE 295.1 K
D1 2.00000000 sec
D11 0.03000000 sec
TD0 1
SFO1 125.7703643 MHz
NUC1 13C
P1 9.75 usec
PLW1 94.00000000 W
SFO2 500.1320005 MHz
NUC2 1H
CPDPRG[2 waltz16
PCPD2 80.00 usec
PLW2 25.00000000 W
PLW12 0.39063001 W
PLW13 0.19648001 W
F2 - Processing parameters
SI 32768
SF 125.7577742 MHz
WDW EM
SSB 0
LB 1.00 Hz
GB 0
PC 1.40
170 160 150 140 130 120 110 100 90 80 70 60 50 40 ppm

9da

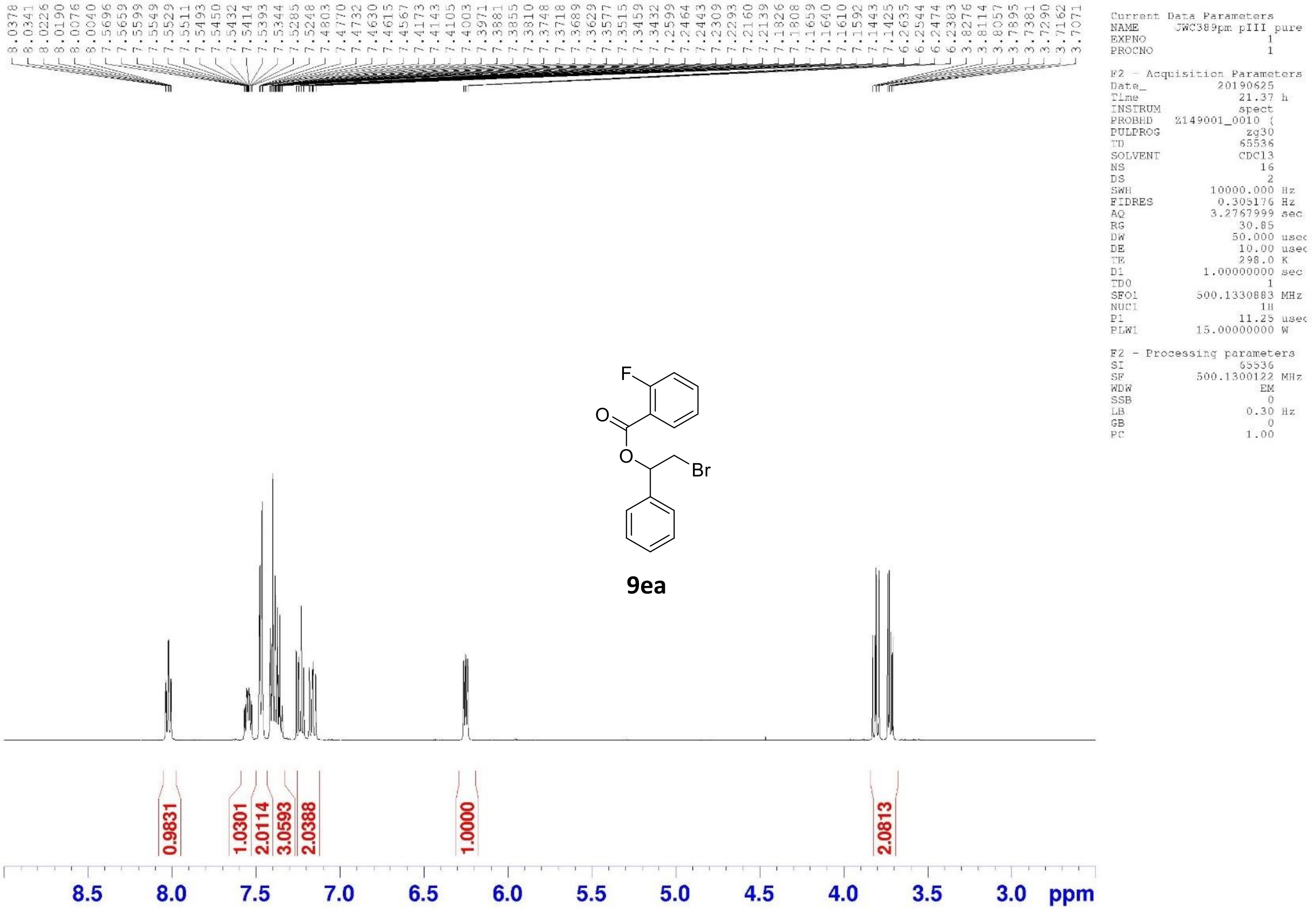
8.0378 8.0341 8.0226 8.0190 8.0076 8.0040 7.5696 7.5659 7.5549 7.5529 7.5511 7.5493 7.5450 7.5432 7.5414 7.5393 7.5344 7.5285 7.5248 7.4803 7.4770 7.4732 7.4630 7.4615 7.4567 7.4173 7.4143 7.4105 7.4003 7.3971 7.3881 7.3855 7.3810 7.3748 7.3718 7.3689 7.3629 7.3577 7.3515 7.3459 7.3432 7.2599 7.2464 7.2443 7.2309 7.2293 7.2160 7.2139 7.1826 7.1808 7.1659 7.1640 7.1610 7.1592 7.1443 7.1425 6.2635 6.2544 6.2474 6.2383 3.8276 3.8114 3.8057 3.7895 3.7381 3.7290 3.7162 3.7071
F
O
O
Br
9ea
0.9831
1.0301
2.0114
3.0593
2.0388
1.0000
2.0813
8.5 8.0 7.5 7.0 6.5 6.0 5.5 5.0 4.5 4.0 3.5 3.0 ppm
Current Data Parameters
NAME JWC389pm pIII pure
EXPNO 1
PROCNO 1
F2 - Acquisition Parameters
Date_ 20190625
Time 21.37 h
INSTRUM spect
PROBHD Z149001_0010 (
PULPROG zg30
TD 65536
SOLVENT CDCl3
NS 16
DS 2
SWH 10000.000 Hz
FIDRES 0.305176 Hz
AQ 3.2767999 sec
RG 30.85
DW 50.000 usec
DE 10.00 usec
TE 298.0 K
D1 1.00000000 sec
TD0 1
SFO1 500.1330883 MHz
NUC1 1H
P1 11.25 usec
PLW1 15.00000000 W
F2 - Processing parameters
SI 65536
SF 500.1300122 MHz
WDW EM
SSB 0
LB 0.30 Hz
GB 0
PC 1.00

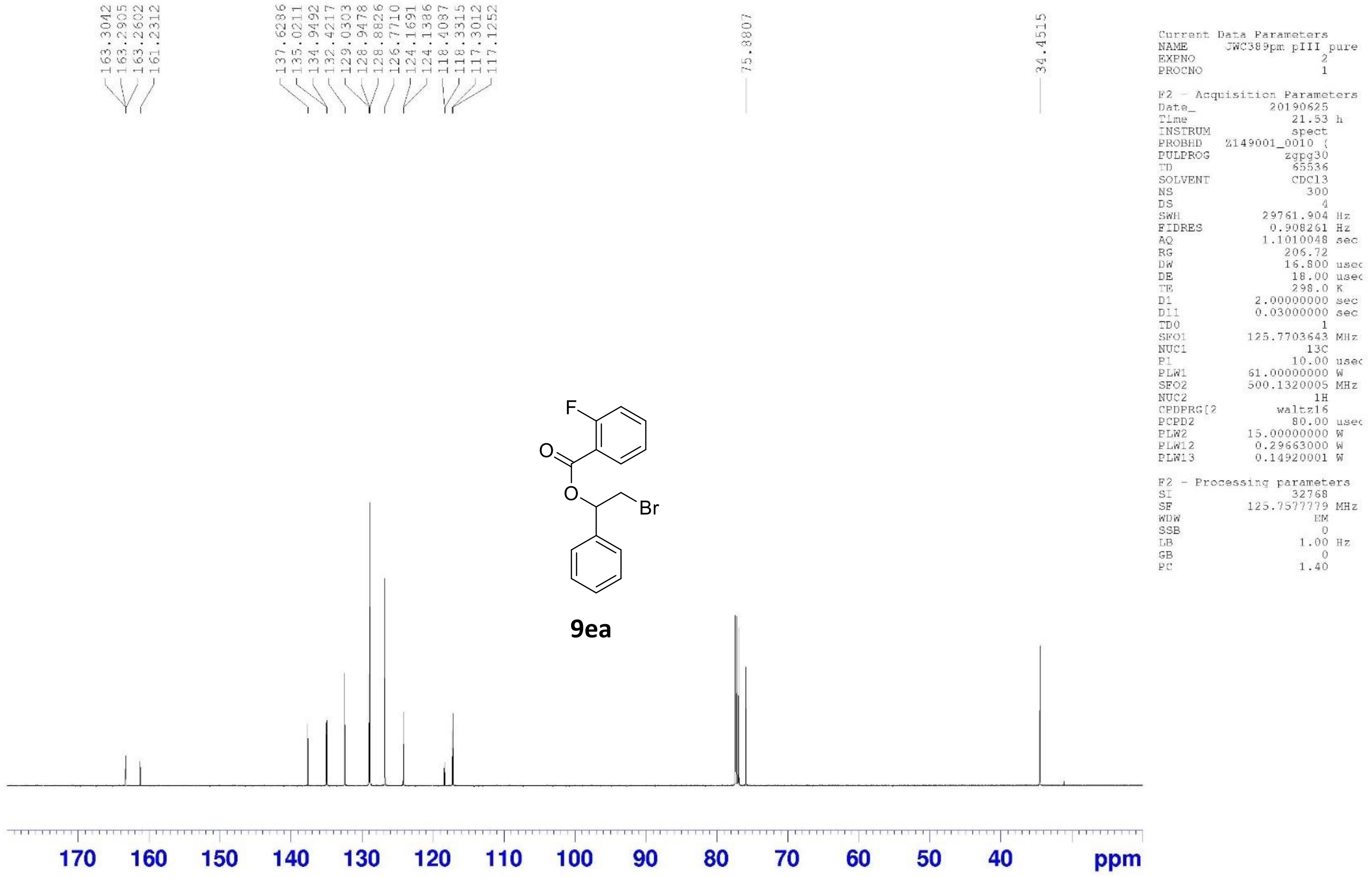
163.3042
163.2905
163.2602
161.2312
137.6286
135.0211
134.9492
132.4217
129.0303
128.9478
128.8826
126.7710
124.1691
124.1386
118.4087
118.3315
117.3012
117.1252
75.8807
34.4515
F
O
O
Br
9ea
170 160 150 140 130 120 110 100 90 80 70 60 50 40 ppm
Current Data Parameters
NAME JWC389pm pIII pure
EXPNO 2
PROCNO 1
F2 - Acquisition Parameters
Date_ 20190625
Time 21.53 h
INSTRUM spect
PROBHD Z149001_0010 (
PULPROG zgpg30
TD 65536
SOLVENT CDCl3
NS 300
DS 4
SWH 29761.904 Hz
FIDRES 0.908261 Hz
AQ 1.1010048 sec
RG 206.72
DW 16.800 usec
DE 18.00 usec
TE 298.0 K
D1 2.00000000 sec
D11 0.03000000 sec
TD0 1
SFO1 125.7703643 MHz
NUC1 13C
P1 10.00 usec
PLW1 61.00000000 W
SFO2 500.1320005 MHz
NUC2 1H
CPDPRG[2 waltz16
PCPD2 80.00 usec
PLW2 15.00000000 W
PLW12 0.29663000 W
PLW13 0.14920001 W
F2 - Processing parameters
SI 32768
SF 125.7577779 MHz
WDW EM
SSB 0
LB 1.00 Hz
GB 0
PC 1.40

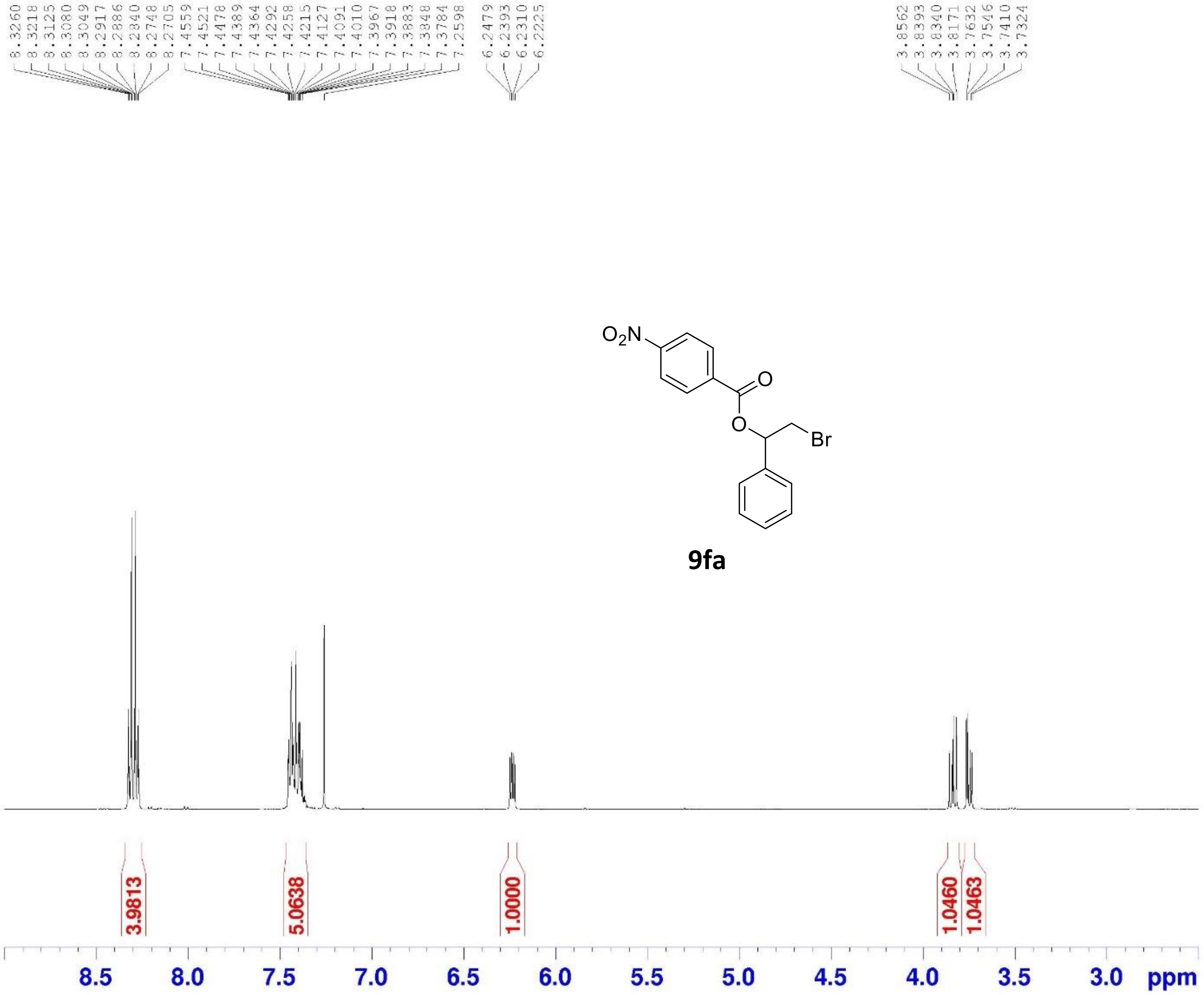

9fa

```
Current Data Parameters
NAME        JWC379pm pure x
EXPNO                     1
PROCNO                    1

F2 - Acquisition Parameters
Date_              20190625
Time                  17.33 h
INSTRUM               spect
PROBHD     Z149001_0010 (
PULPROG                zg30
TD                    65536
SOLVENT               CDCl3
NS                       16
DS                        2
SWH               10000.000 Hz
FIDRES             0.305176 Hz
AQ                3.2767999 sec
RG                    30.85
DW                   50.000 usec
DE                    10.00 usec
TE                    298.0 K
D1               1.00000000 sec
TD0                       1
SFO1            500.1330883 MHz
NUC1                     1H
P1                    11.25 usec
PLW1            15.00000000 W

F2 - Processing parameters
SI                    65536
SF              500.1300125 MHz
WDW                      EM
SSB                       0
LB                     0.30 Hz
GB                        0
PC                     1.00
```

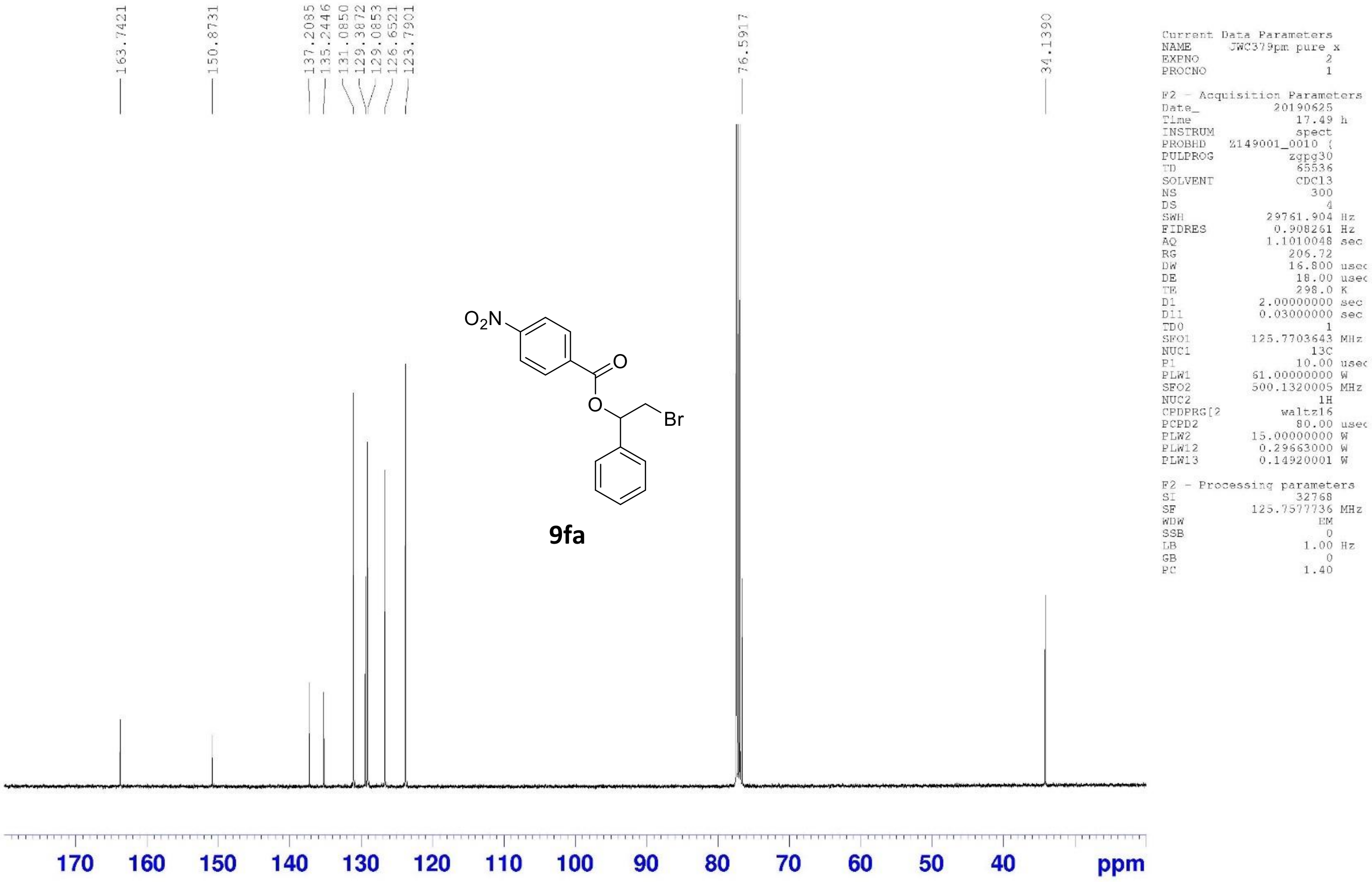
163.7421
150.8731
137.2085
135.2446
131.0850
129.3872
129.0853
126.6521
123.7901
76.5917
34.1390
O2N
O
O
Br
9fa
170 160 150 140 130 120 110 100 90 80 70 60 50 40 ppm
Current Data Parameters
NAME JWC379pm pure x
EXPNO 2
PROCNO 1
F2 - Acquisition Parameters
Date_ 20190625
Time 17.49 h
INSTRUM spect
PROBHD Z149001_0010 (
PULPROG zgpg30
TD 65536
SOLVENT CDCl3
NS 300
DS 4
SWH 29761.904 Hz
FIDRES 0.908261 Hz
AQ 1.1010048 sec
RG 206.72
DW 16.800 usec
DE 18.00 usec
TE 298.0 K
D1 2.00000000 sec
D11 0.03000000 sec
TD0 1
SFO1 125.7703643 MHz
NUC1 13C
P1 10.00 usec
PLW1 61.00000000 W
SFO2 500.1320005 MHz
NUC2 1H
CPDPRG[2 waltz16
PCPD2 80.00 usec
PLW2 15.00000000 W
PLW12 0.29663000 W
PLW13 0.14920001 W
F2 - Processing parameters
SI 32768
SF 125.7577736 MHz
WDW EM
SSB 0
LB 1.00 Hz
GB 0
PC 1.40

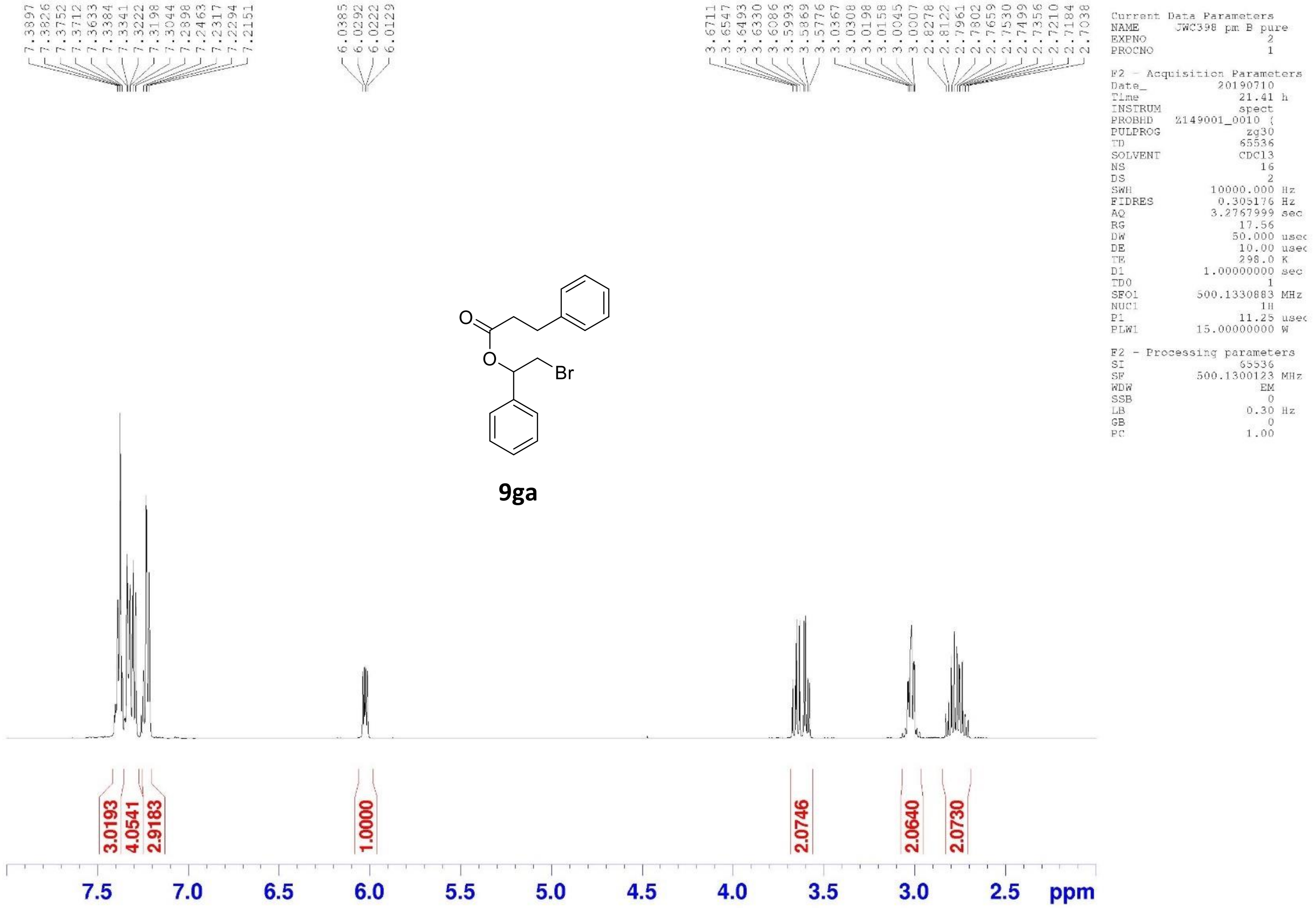
7.3897 7.3826 7.3752 7.3712 7.3633 7.3384 7.3341 7.3222 7.3198 7.3044 7.2898 7.2463 7.2317 7.2294 7.2151
6.0385 6.0292 6.0222 6.0129
3.6711 3.6547 3.6493 3.6330 3.6086 3.5993 3.5869 3.5776 3.0367 3.0308 3.0198 3.0158 3.0045 3.0007 2.8278 2.8122 2.7961 2.7802 2.7659 2.7530 2.7499 2.7356 2.7210 2.7184 2.7038
O
O
Br
9ga
3.0193 4.0541 2.9183
1.0000
2.0746
2.0640
2.0730
7.5 7.0 6.5 6.0 5.5 5.0 4.5 4.0 3.5 3.0 2.5 ppm
Current Data Parameters
NAME JWC398 pm B pure
EXPNO 2
PROCNO 1
F2 - Acquisition Parameters
Date_ 20190710
Time 21.41 h
INSTRUM spect
PROBHD Z149001_0010 (
PULPROG zg30
TD 65536
SOLVENT CDCl3
NS 16
DS 2
SWH 10000.000 Hz
FIDRES 0.305176 Hz
AQ 3.2767999 sec
RG 17.56
DW 50.000 usec
DE 10.00 usec
TE 298.0 K
D1 1.00000000 sec
TD0 1
SFO1 500.1330883 MHz
NUC1 1H
P1 11.25 usec
PLW1 15.00000000 W
F2 - Processing parameters
SI 65536
SF 500.1300123 MHz
WDW EM
SSB 0
LB 0.30 Hz
GB 0
PC 1.00

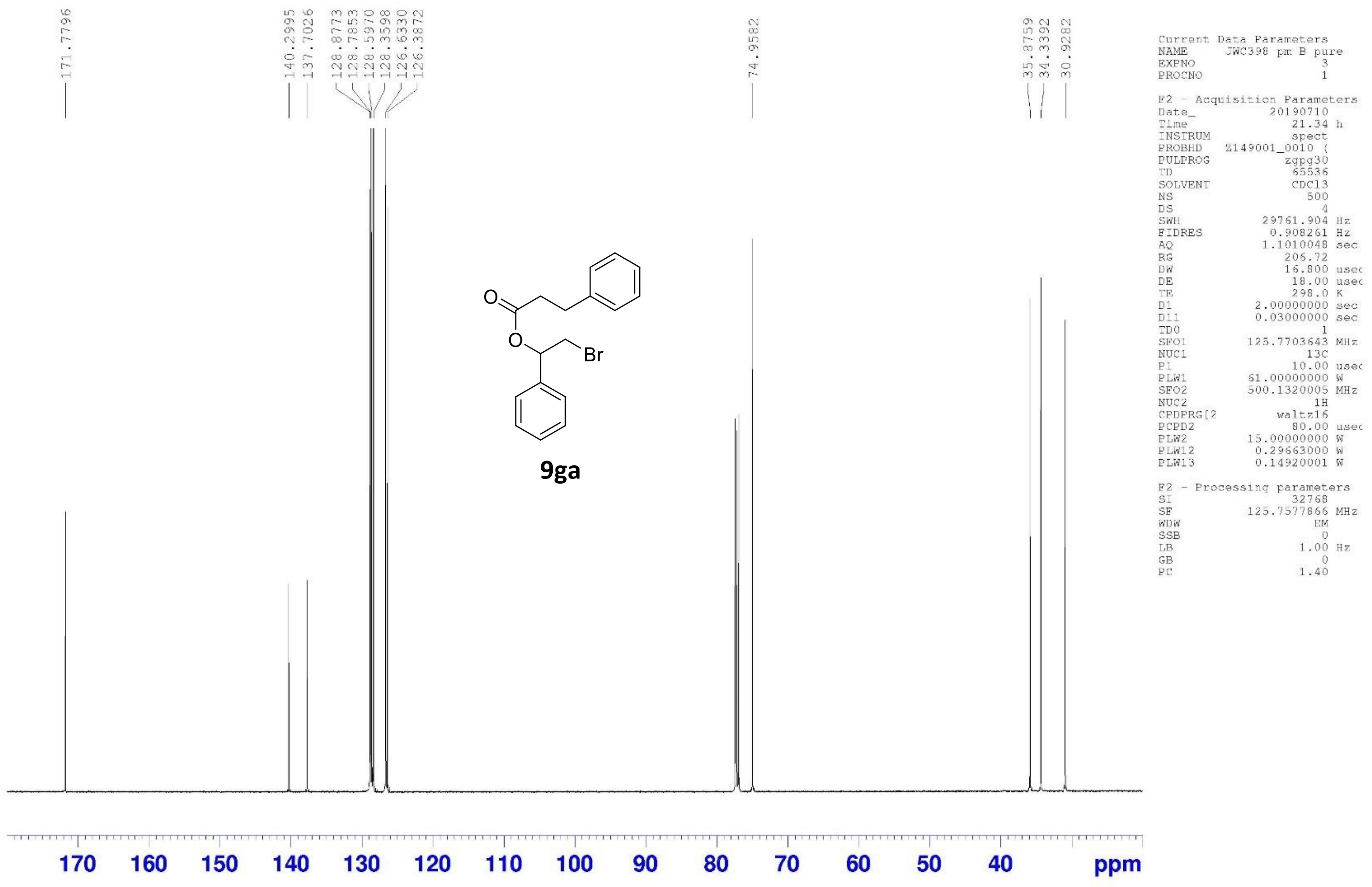
171.7796
140.2995
137.7026
128.8773
128.7853
128.5970
128.3598
126.6330
126.3872
74.9582
35.8759
34.3392
30.9282
O
O
Br
9ga
Current Data Parameters
NAME JWC398 pm B pure
EXPNO 3
PROCNO 1
F2 - Acquisition Parameters
Date_ 20190710
Time 21.34 h
INSTRUM spect
PULPROG zgpg30
TD 65536
SOLVENT CDCl3
NS 500
DS 4
SWH 29761.904 Hz
FIDRES 0.908261 Hz
AQ 1.1010048 sec
RG 206.72
DW 16.800 usec
DE 18.00 usec
TE 298.0 K
D1 2.00000000 sec
D11 0.03000000 sec
TD0 1
SFO1 125.7703643 MHz
NUC1 13C
P1 10.00 usec
PLW1 61.00000000 W
SFO2 500.1320005 MHz
NUC2 1H
CPDPRG[2 waltz16
PCPD2 80.00 usec
PLW2 15.00000000 W
PLW12 0.29663000 W
PLW13 0.14920001 W
F2 - Processing parameters
SI 32768
SF 125.7577866 MHz
WDW EM
SSB 0
LB 1.00 Hz
GB 0
PC 1.40
170
160
150
140
130
120
110
100
90
80
70
60
50
40
ppm

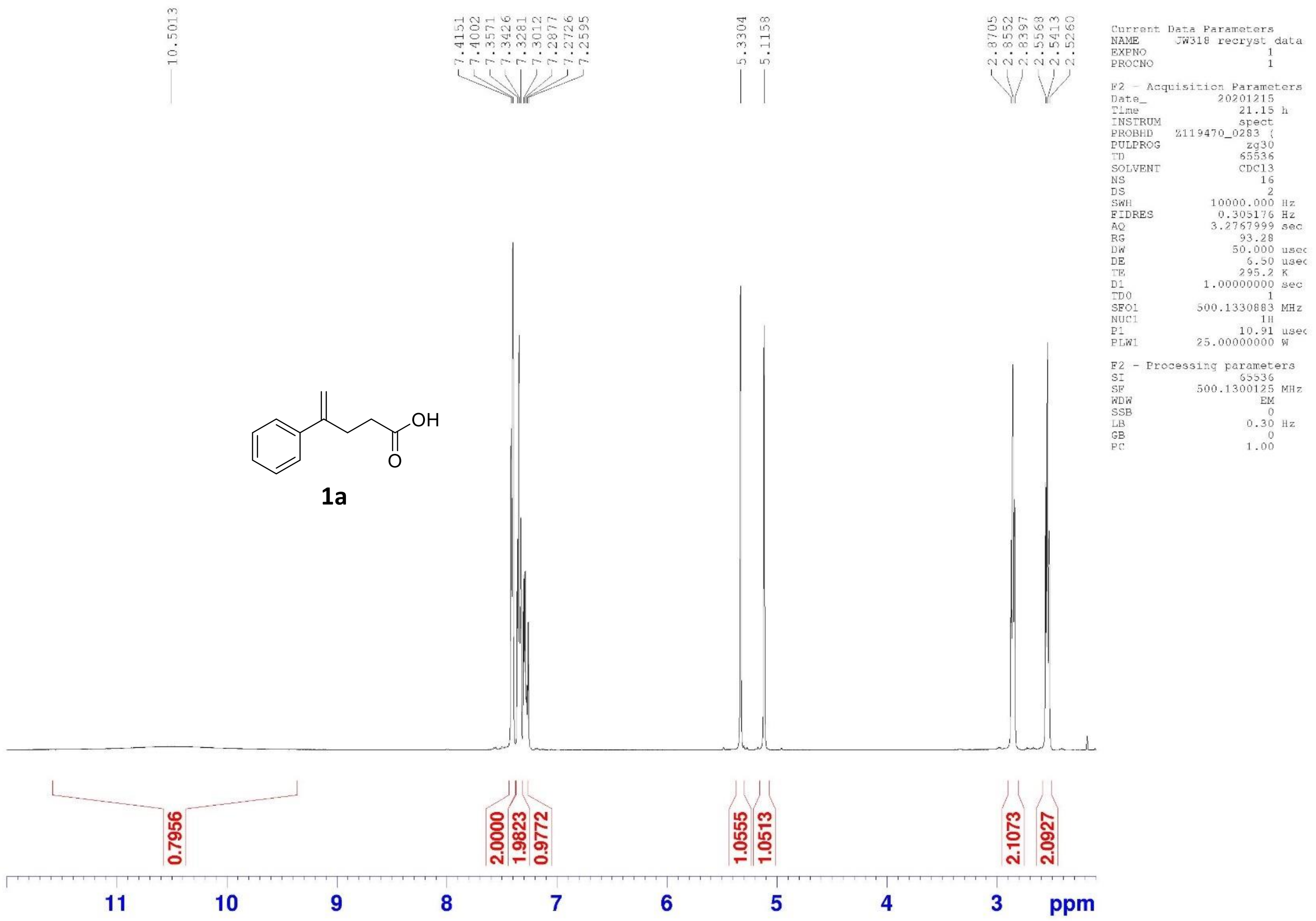
10.5013
7.4151
7.4002
7.3571
7.3426
7.3281
7.3012
7.2877
7.2726
7.2595
5.3304
5.1158
2.8705
2.8552
2.8397
2.5568
2.5413
2.5260
OH
O
1a
0.7956
2.0000
1.9823
0.9772
1.0555
1.0513
2.1073
2.0927
11
10
9
8
7
6
5
4
3
ppm
Current Data Parameters
NAME JW318 recryst data
EXPNO 1
PROCNO 1
F2 - Acquisition Parameters
Date_ 20201215
Time 21.15 h
INSTRUM spect
PROBHD Z119470_0283 (
PULPROG zg30
TD 65536
SOLVENT CDCl3
NS 16
DS 2
SWH 10000.000 Hz
FIDRES 0.305176 Hz
AQ 3.2767999 sec
RG 93.28
DW 50.000 usec
DE 6.50 usec
TE 295.2 K
D1 1.00000000 sec
TD0 1
SFO1 500.1330883 MHz
NUC1 1H
P1 10.91 usec
PLW1 25.00000000 W
F2 - Processing parameters
SI 65536
SF 500.1300125 MHz
WDW EM
SSB 0
LB 0.30 Hz
GB 0
PC 1.00

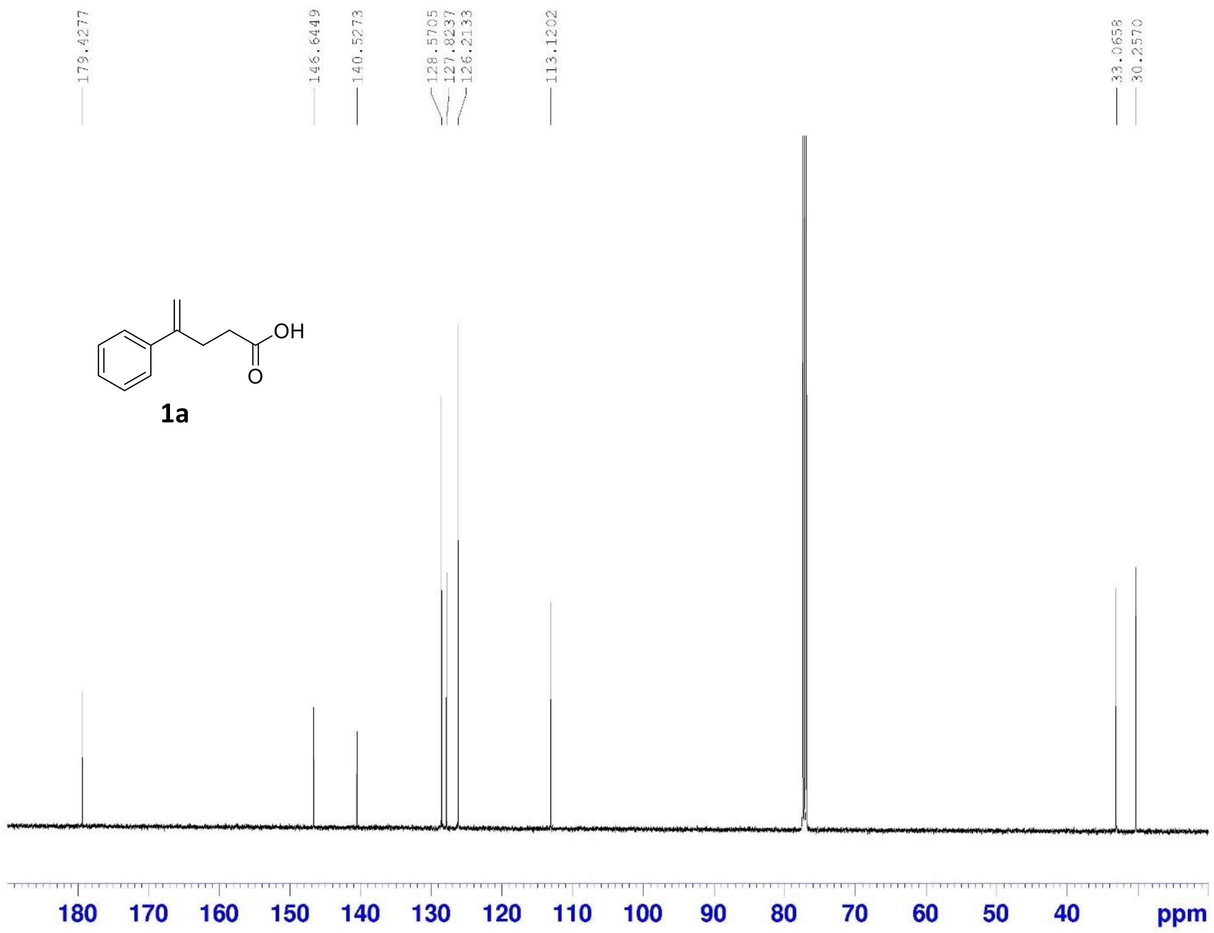

1a

Current Data Parameters		
NAME	JW318 recryst data	
EXPNO	2	
PROCNO	1	
F2 - Acquisition Parameters		
Date_	20201215	
Time	21.52	h
INSTRUM	spect	
PROBHD	Z119470_0283 (	
PULPROG	zgpg30	
TD	65536	
SOLVENT	CDCl3	
NS	700	
DS	4	
SWH	29761.904	Hz
FIDRES	0.908261	Hz
AQ	1.1010048	sec
RG	206.72	
DW	16.800	usec
DE	6.50	usec
TE	295.1	K
D1	2.00000000	sec
D11	0.03000000	sec
TD0	1	
SFO1	125.7703643	MHz
NUC1	13C	
P1	9.75	usec
PLW1	94.00000000	W
SFO2	500.1320005	MHz
NUC2	1H	
CPDPRG[2	waltz16	
PCPD2	80.00	usec
PLW2	25.00000000	W
PLW12	0.46495000	W
PLW13	0.23387000	W
F2 - Processing parameters		
SI	32768	
SF	125.7577740	MHz
WDW	EM	
SSB	0	
LB	1.00	Hz
GB	0	
PC	1.40	

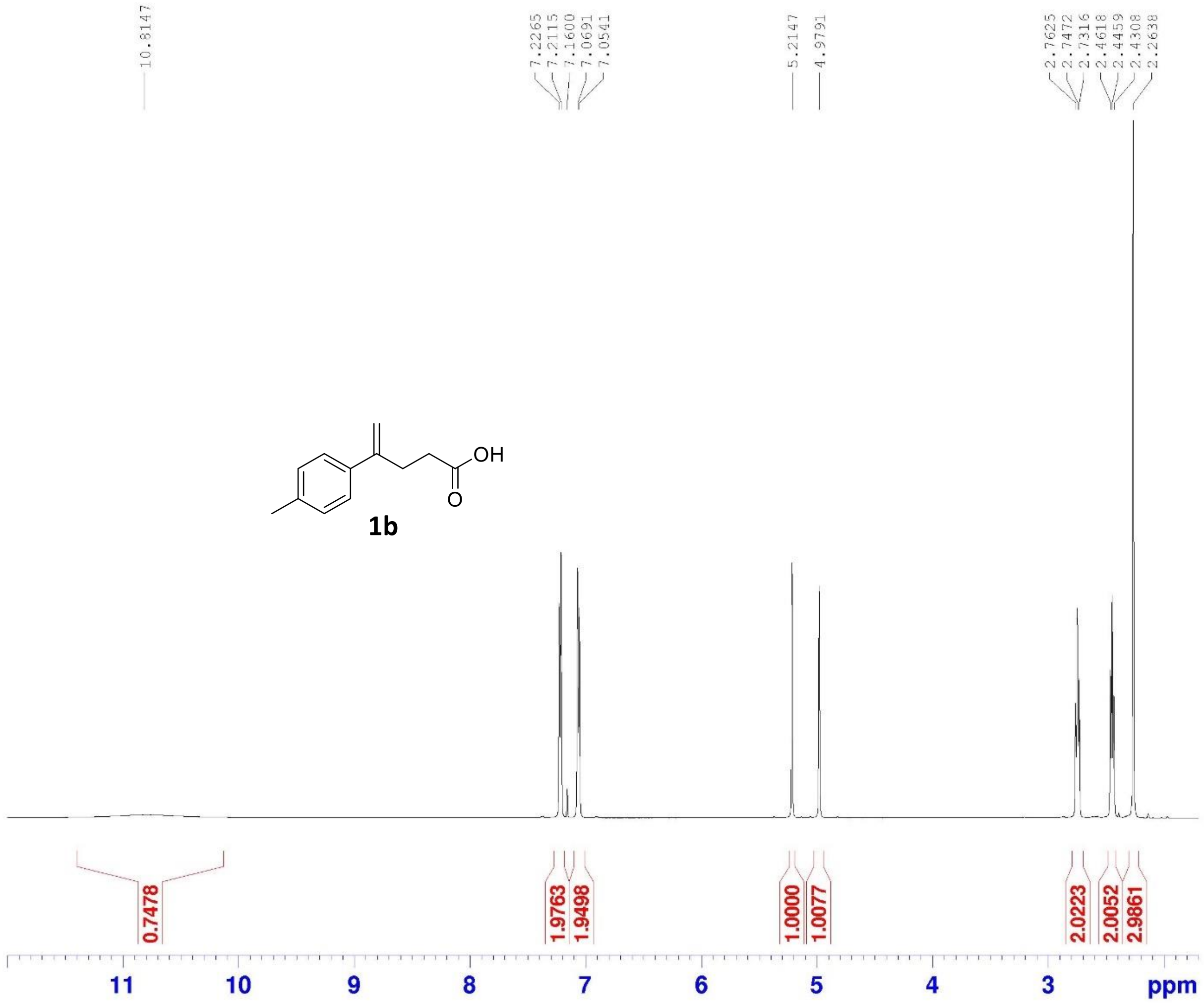

Current Data Parameters
NAME JW344 data
EXPNO 1
PROCNO 1

F2 - Acquisition Parameters
Date_ 20201210
Time 23.37 h
INSTRUM spect
PROBHD Z119470_0283 (
PULPROG zg30
TD 65536
SOLVENT CDCl3
NS 16
DS 2
SWH 10000.000 Hz
FIDRES 0.305176 Hz
AQ 3.2767999 sec
RG 50.6
DW 50.000 usec
DE 6.50 usec
TE 295.2 K
D1 1.00000000 sec
TD0 1
SFO1 500.1330883 MHz
NUC1 1H
P1 10.91 usec
PLW1 25.00000000 W

F2 - Processing parameters
SI 65536
SF 500.1300625 MHz
WDW EM
SSB 0
LB 0.30 Hz
GB 0
PC 1.00

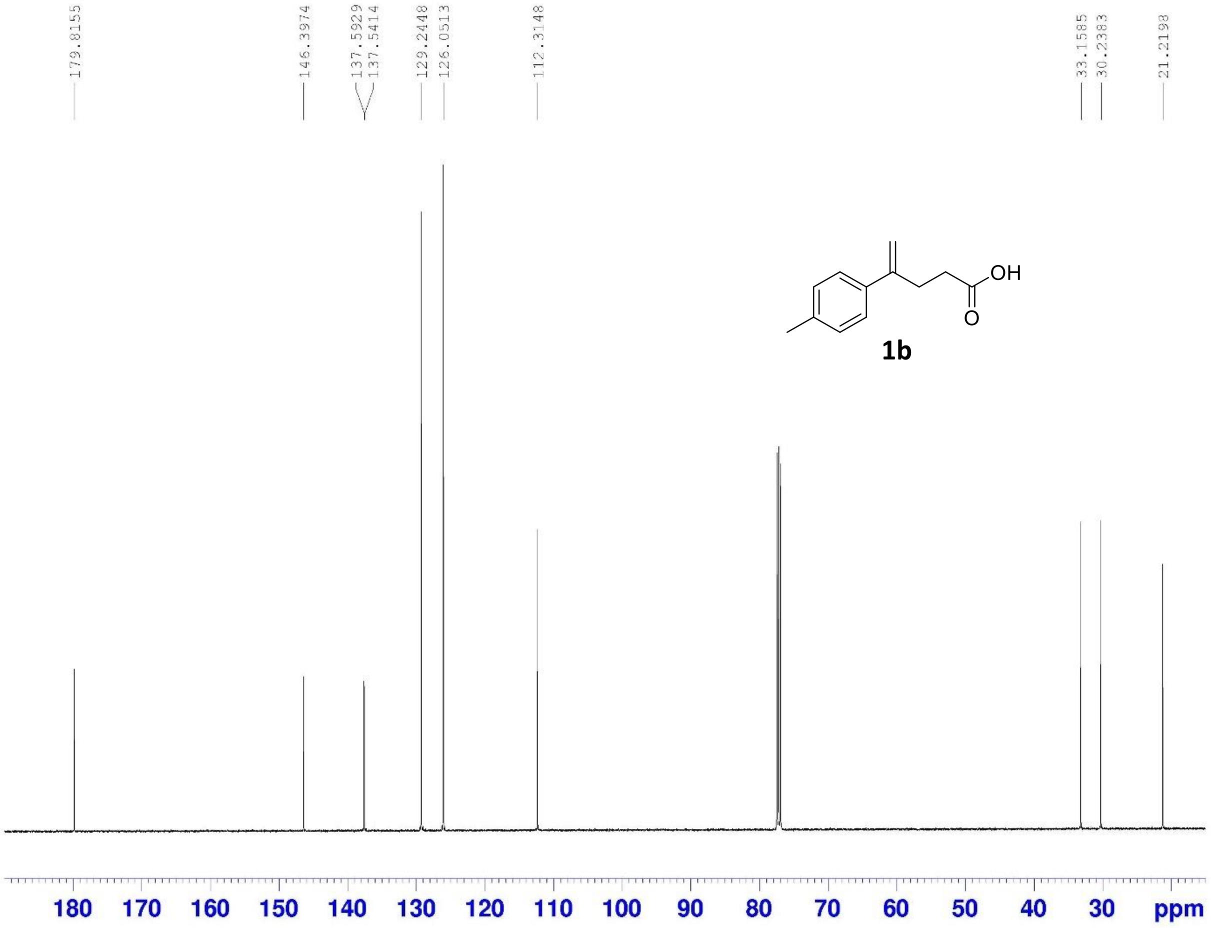

1b

Current Data Parameters		
NAME	JW344 data	
EXPNO	2	
PROCNO	1	
F2 - Acquisition Parameters		
Date_	20201211	
Time	2.31	h
INSTRUM	spect	
PROBHD	Z119470_0283 (	
PULPROG	zgpg30	
TD	65536	
SOLVENT	CDCl3	
NS	1024	
DS	4	
SWH	29761.904	Hz
FIDRES	0.908261	Hz
AQ	1.1010048	sec
RG	206.72	
DW	16.800	usec
DE	6.50	usec
TE	295.2	K
D1	2.00000000	sec
D11	0.03000000	sec
TD0	1	
SFO1	125.7703643	MHz
NUC1	13C	
P1	9.75	usec
PLW1	94.00000000	W
SFO2	500.1320005	MHz
NUC2	1H	
CPDPRG[2	waltz16	
PCPD2	80.00	usec
PLW2	25.00000000	W
PLW12	0.46495000	W
PLW13	0.23387000	W
F2 - Processing parameters		
SI	32768	
SF	125.7577771	MHz
WDW	EM	
SSB	0	
LB	1.00	Hz
GB	0	
PC	1.40	

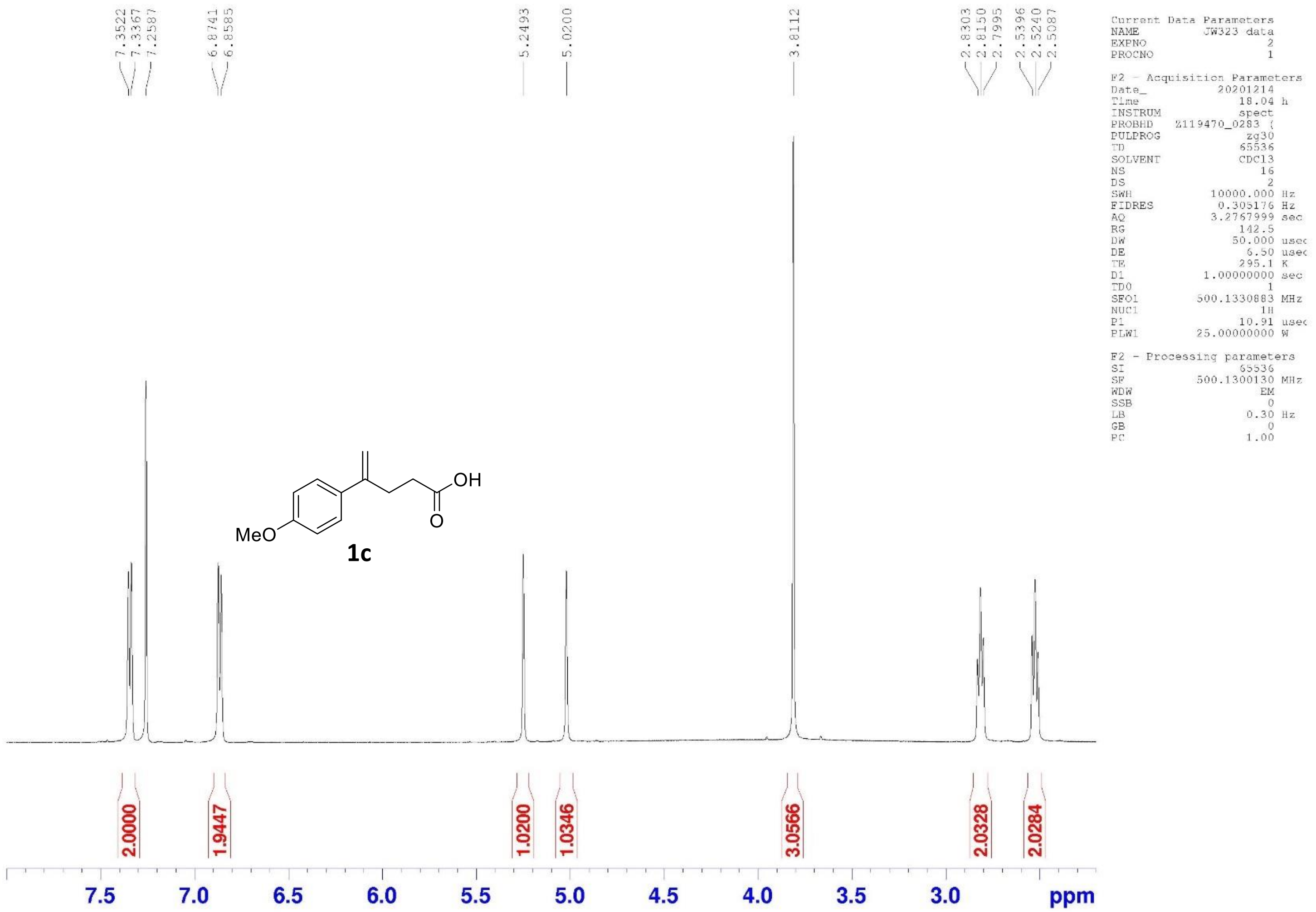
7.3522
7.3367
7.2587
6.8741
6.8585
5.2493
5.0200
3.8112
2.8303
2.8150
2.7995
2.5396
2.5240
2.5087
OH
O
MeO
1c
2.0000
1.9447
1.0200
1.0346
3.0566
2.0328
2.0284
7.5
7.0
6.5
6.0
5.5
5.0
4.5
4.0
3.5
3.0
ppm
Current Data Parameters
NAME JW323 data
EXPNO 2
PROCNO 1
F2 - Acquisition Parameters
Date_ 20201214
Time 18.04 h
INSTRUM spect
PROBHD Z119470_0283 (
PULPROG zg30
TD 65536
SOLVENT CDCl3
NS 16
DS 2
SWH 10000.000 Hz
FIDRES 0.305176 Hz
AQ 3.2767999 sec
RG 142.5
DW 50.000 usec
DE 6.50 usec
TE 295.1 K
D1 1.00000000 sec
TD0 1
SFO1 500.1330883 MHz
NUC1 1H
P1 10.91 usec
PLW1 25.00000000 W
F2 - Processing parameters
SI 65536
SF 500.1300130 MHz
WDW EM
SSB 0
LB 0.30 Hz
GB 0
PC 1.00

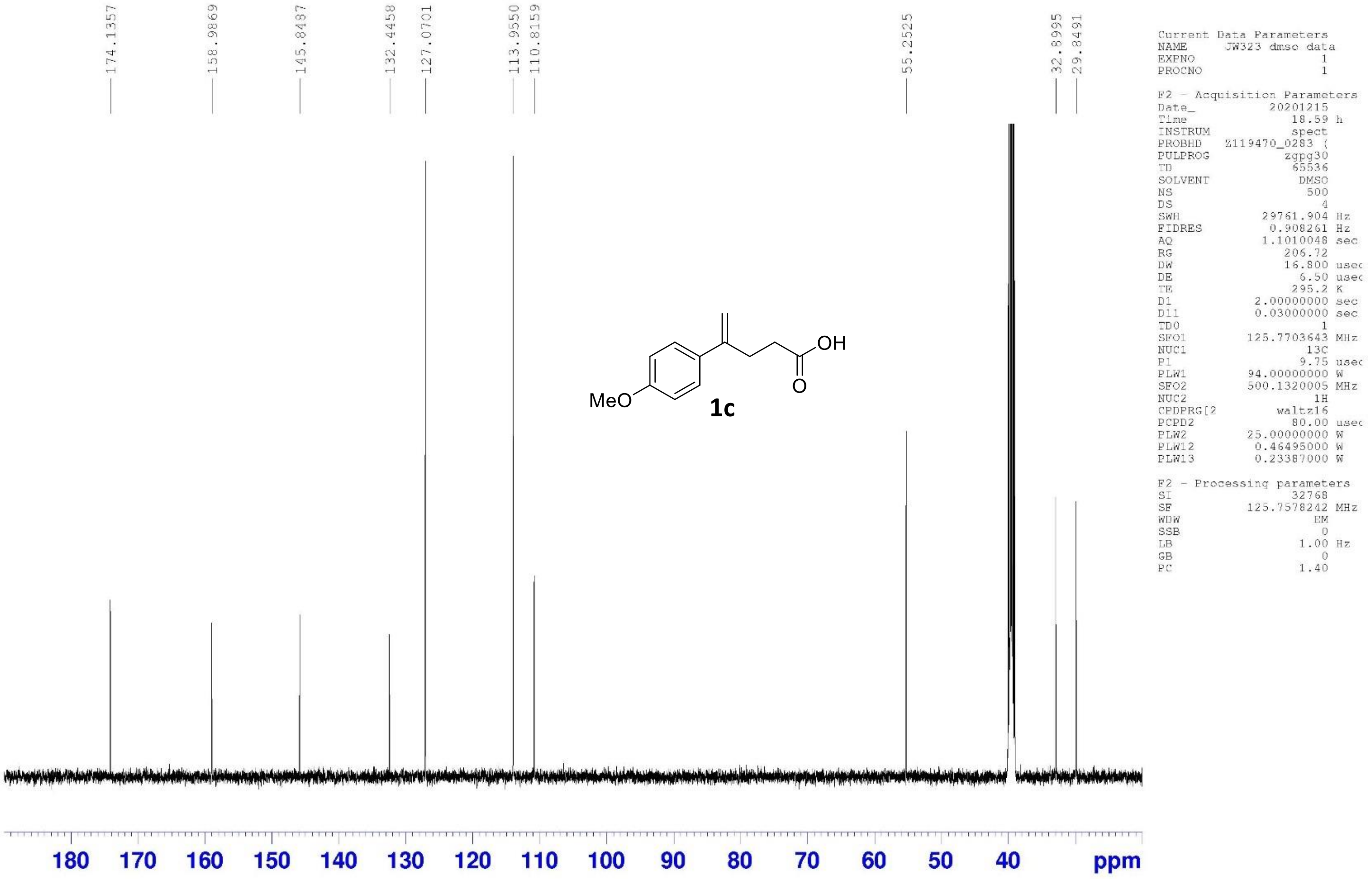

174.1357
158.9869
145.8487
132.4458
127.0701
113.9550
110.8159
55.2525
32.8995
29.8491
MeO
OH
O
1c
180 170 160 150 140 130 120 110 100 90 80 70 60 50 40 ppm
Current Data Parameters
NAME JW323 dmso data
EXPNO 1
PROCNO 1
F2 - Acquisition Parameters
Date_ 20201215
Time 18.59 h
INSTRUM spect
PROBHD Z119470_0283 (
PULPROG zgpg30
TD 65536
SOLVENT DMSO
NS 500
DS 4
SWH 29761.904 Hz
FIDRES 0.908261 Hz
AQ 1.1010048 sec
RG 206.72
DW 16.800 usec
DE 6.50 usec
TE 295.2 K
D1 2.00000000 sec
D11 0.03000000 sec
TD0 1
SFO1 125.7703643 MHz
NUC1 13C
P1 9.75 usec
PLW1 94.00000000 W
SFO2 500.1320005 MHz
NUC2 1H
CPDPRG[2 waltz16
PCPD2 80.00 usec
PLW2 25.00000000 W
PLW12 0.46495000 W
PLW13 0.23387000 W
F2 - Processing parameters
SI 32768
SF 125.7578242 MHz
WDW EM
SSB 0
LB 1.00 Hz
GB 0
PC 1.40

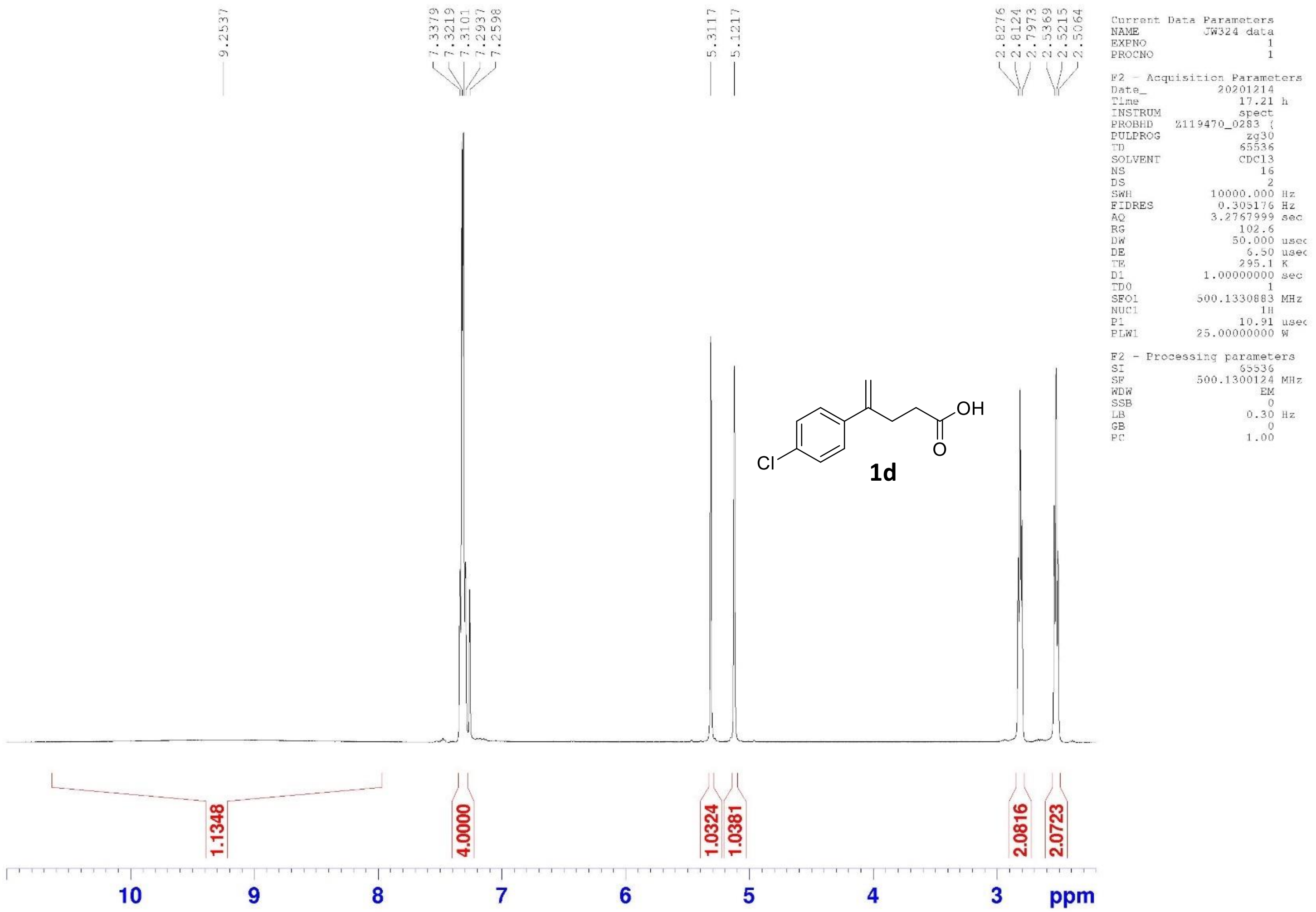
9.2537
7.3379
7.3219
7.3101
7.2937
7.2598
5.3117
5.1217
2.8276
2.8124
2.7973
2.5369
2.5215
2.5064
Current Data Parameters
NAME JW324 data
EXPNO 1
PROCNO 1
F2 - Acquisition Parameters
Date_ 20201214
Time 17.21 h
INSTRUM spect
PROBHD Z119470_0283 (
PULPROG zg30
TD 65536
SOLVENT CDCl3
NS 16
DS 2
SWH 10000.000 Hz
FIDRES 0.305176 Hz
AQ 3.2767999 sec
RG 102.6
DW 50.000 usec
DE 6.50 usec
TE 295.1 K
D1 1.00000000 sec
TD0 1
SFO1 500.1330883 MHz
NUC1 1H
P1 10.91 usec
PLW1 25.00000000 W
F2 - Processing parameters
SI 65536
SF 500.1300124 MHz
WDW EM
SSB 0
LB 0.30 Hz
GB 0
PC 1.00
OH
O
Cl
1d
1.1348
4.0000
1.0324
1.0381
2.0816
2.0723
10
9
8
7
6
5
4
3
ppm

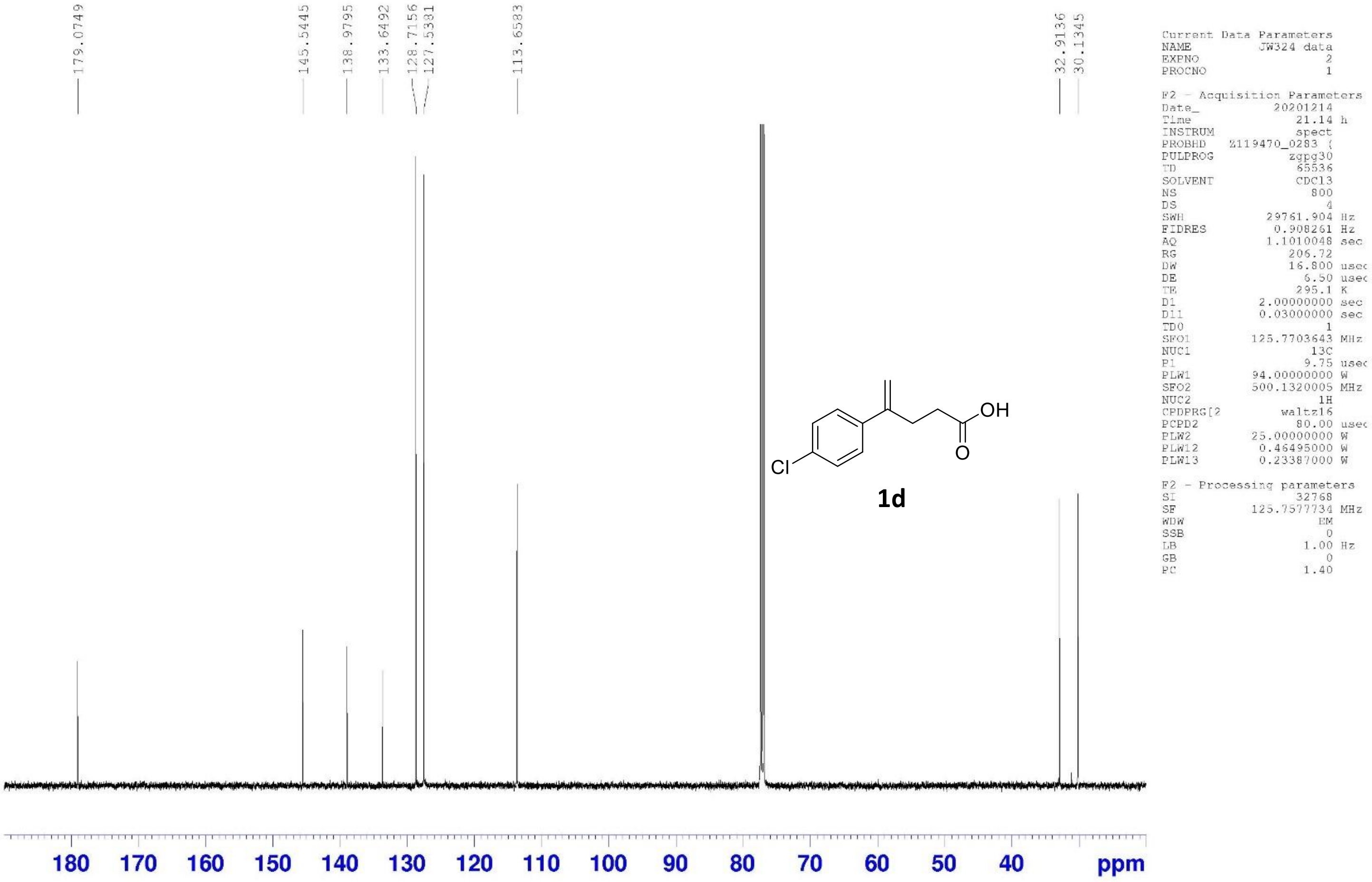
179.0749
145.5445
138.9795
133.6492
128.7156
127.5381
113.6583
32.9136
30.1345
Current Data Parameters
NAME JW324 data
EXPNO 2
PROCNO 1
F2 - Acquisition Parameters
Date_ 20201214
Time 21.14 h
INSTRUM spect
PROBHD Z119470_0283 (
PULPROG zgpg30
TD 65536
SOLVENT CDCl3
NS 800
DS 4
SWH 29761.904 Hz
FIDRES 0.908261 Hz
AQ 1.1010048 sec
RG 206.72
DW 16.800 usec
DE 6.50 usec
TE 295.1 K
D1 2.00000000 sec
D11 0.03000000 sec
TD0 1
SFO1 125.7703643 MHz
NUC1 13C
P1 9.75 usec
PLW1 94.00000000 W
SFO2 500.1320005 MHz
NUC2 1H
CPDPRG[2 waltz16
PCPD2 80.00 usec
PLW2 25.00000000 W
PLW12 0.46495000 W
PLW13 0.23387000 W
F2 - Processing parameters
SI 32768
SF 125.7577734 MHz
WDW EM
SSB 0
LB 1.00 Hz
GB 0
PC 1.40
OH
O
Cl
1d
180 170 160 150 140 130 120 110 100 90 80 70 60 50 40 ppm

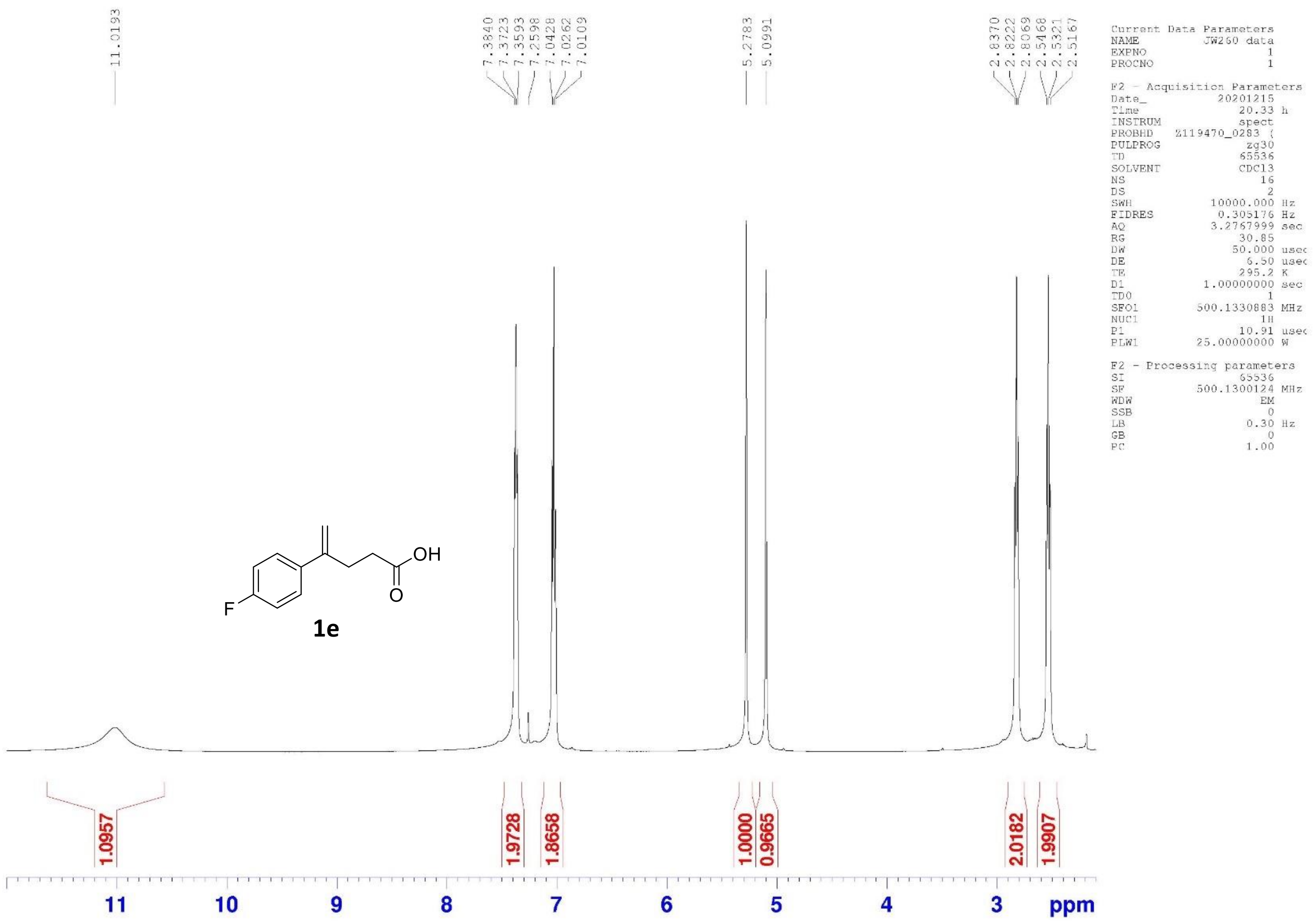
11.0193
7.3840
7.3723
7.3593
7.2598
7.0428
7.0262
7.0109
5.2783
5.0991
2.8370
2.8222
2.8069
2.5468
2.5321
2.5167
Current Data Parameters
NAME JW260 data
EXPNO 1
PROCNO 1
F2 - Acquisition Parameters
Date_ 20201215
Time 20.33 h
INSTRUM spect
PROBHD Z119470_0283 (
PULPROG zg30
TD 65536
SOLVENT CDCl3
NS 16
DS 2
SWH 10000.000 Hz
FIDRES 0.305176 Hz
AQ 3.2767999 sec
RG 30.85
DW 50.000 usec
DE 6.50 usec
TE 295.2 K
D1 1.00000000 sec
TD0 1
SFO1 500.1330883 MHz
NUC1 1H
P1 10.91 usec
PLW1 25.00000000 W
F2 - Processing parameters
SI 65536
SF 500.1300124 MHz
WDW EM
SSB 0
LB 0.30 Hz
GB 0
PC 1.00
OH
O
F
1e
1.0957
1.9728
1.8658
1.0000
0.9665
2.0182
1.9907
11
10
9
8
7
6
5
4
3
ppm

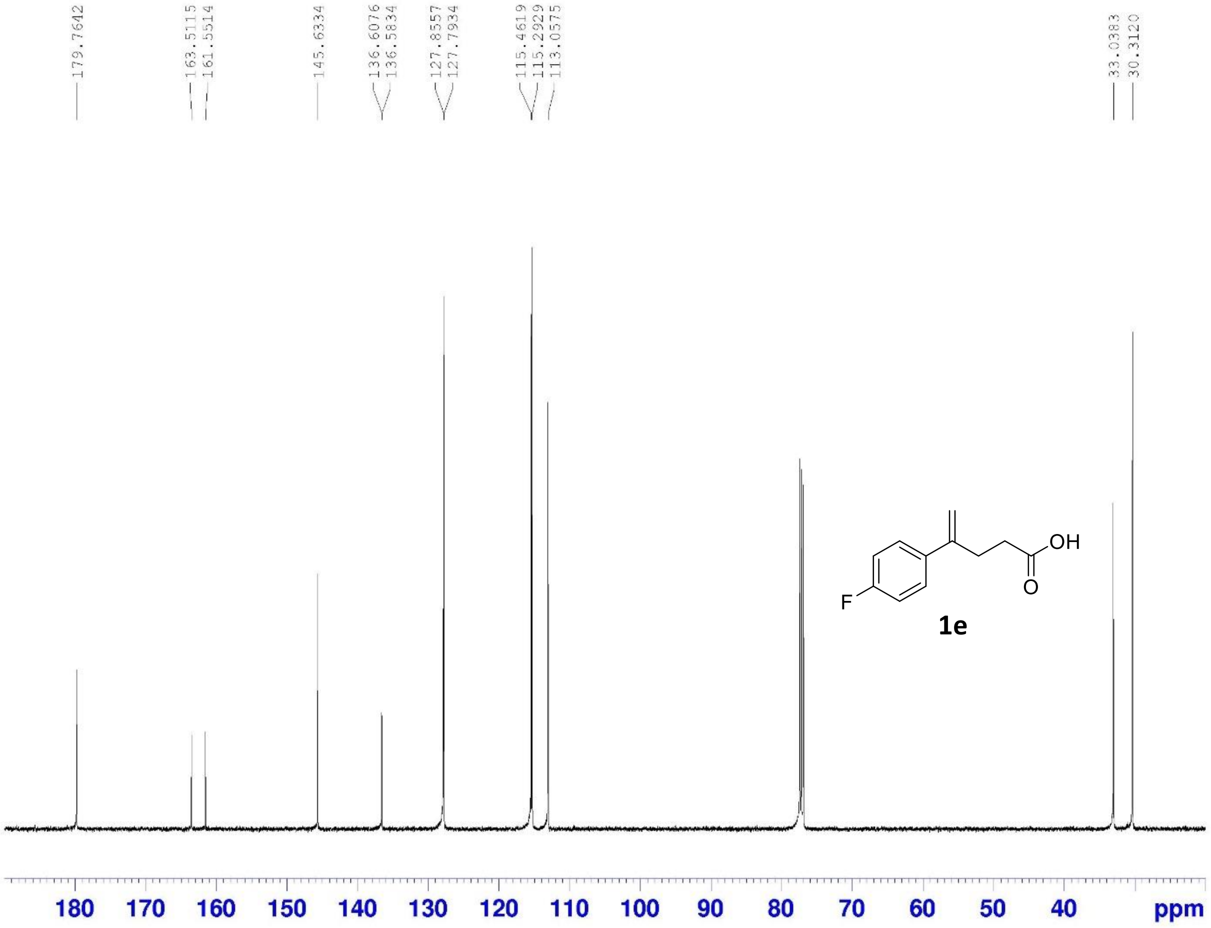


```
Current Data Parameters
NAME          JW260 data
EXPNO                  2
PROCNO                 1

F2 - Acquisition Parameters
Date_           20201215
Time               21.11 h
INSTRUM            spect
PROBHD    Z119470_0283 (
PULPROG           zgpg30
TD                 65536
SOLVENT            CDCl3
NS                   700
DS                     4
SWH            29761.904 Hz
FIDRES          0.908261 Hz
AQ             1.1010048 sec
RG                206.72
DW                16.800 usec
DE                  6.50 usec
TE                 295.2 K
D1            2.00000000 sec
D11           0.03000000 sec
TD0                    1
SFO1         125.7703643 MHz
NUC1                 13C
P1                  9.75 usec
PLW1         94.00000000 W
SFO2         500.1320005 MHz
NUC2                  1H
CPDPRG[2         waltz16
PCPD2              80.00 usec
PLW2         25.00000000 W
PLW12         0.46495000 W
PLW13         0.23387000 W

F2 - Processing parameters
SI                 32768
SF           125.7577767 MHz
WDW                   EM
SSB                    0
LB                  1.00 Hz
GB                     0
PC                  1.40
```

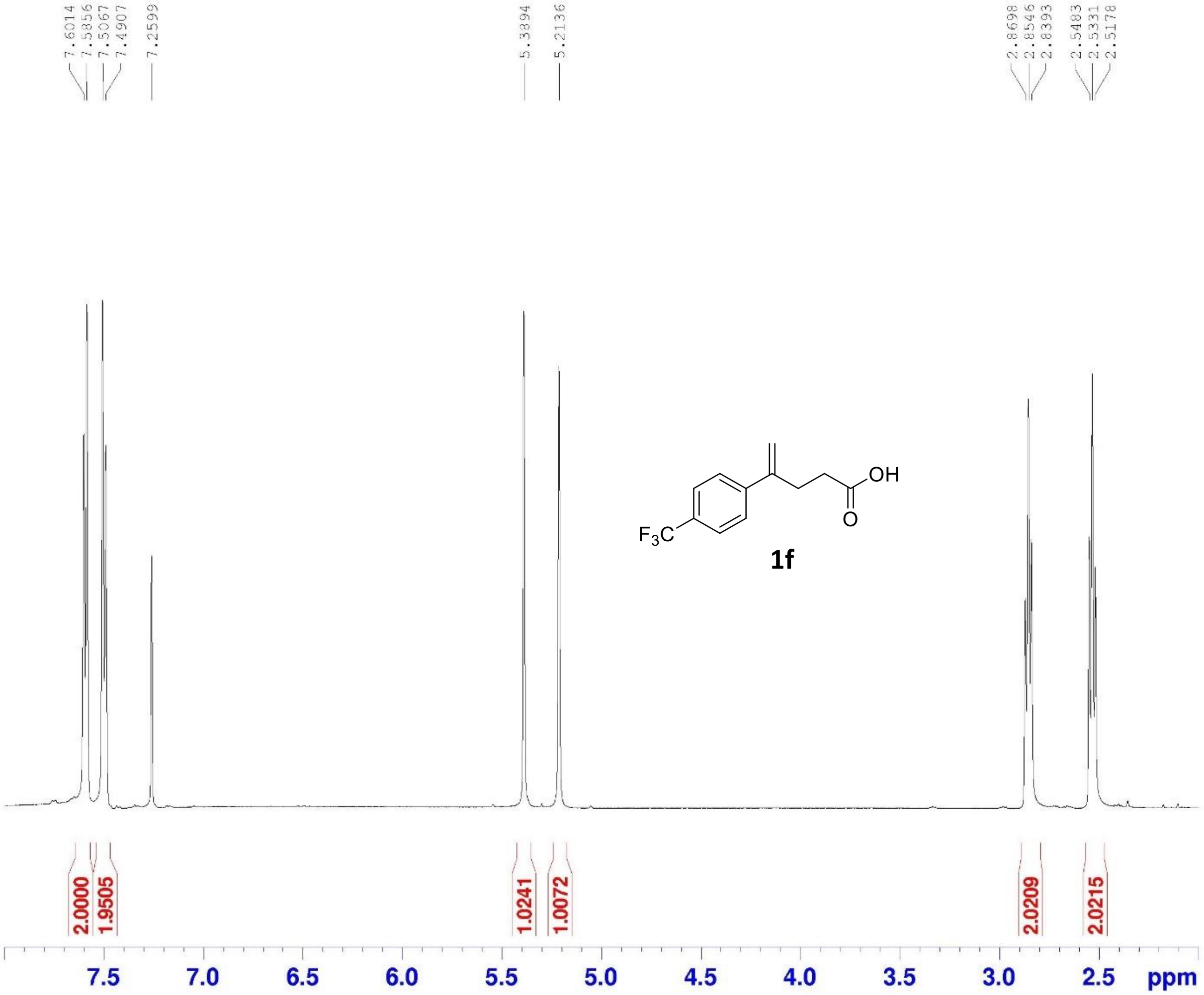


```
Current Data Parameters
NAME       JW287 ptlc data
EXPNO                    2
PROCNO                   1

F2 - Acquisition Parameters
Date_             20201223
Time                 22.29 h
INSTRUM              spect
PROBHD   Z119470_0283 (
PULPROG               zg30
TD                   65536
SOLVENT              CDCl3
NS                      16
DS                       2
SWH              10000.000 Hz
FIDRES            0.305176 Hz
AQ               3.2767999 sec
RG                   142.5
DW                  50.000 usec
DE                    6.50 usec
TE                   295.2 K
D1              1.00000000 sec
TD0                      1
SFO1           500.1330883 MHz
NUC1                    1H
P1                   10.91 usec
PLW1           25.00000000 W

F2 - Processing parameters
SI                   65536
SF             500.1300121 MHz
WDW                     EM
SSB                      0
LB                    0.30 Hz
GB                       0
PC                    1.00
```

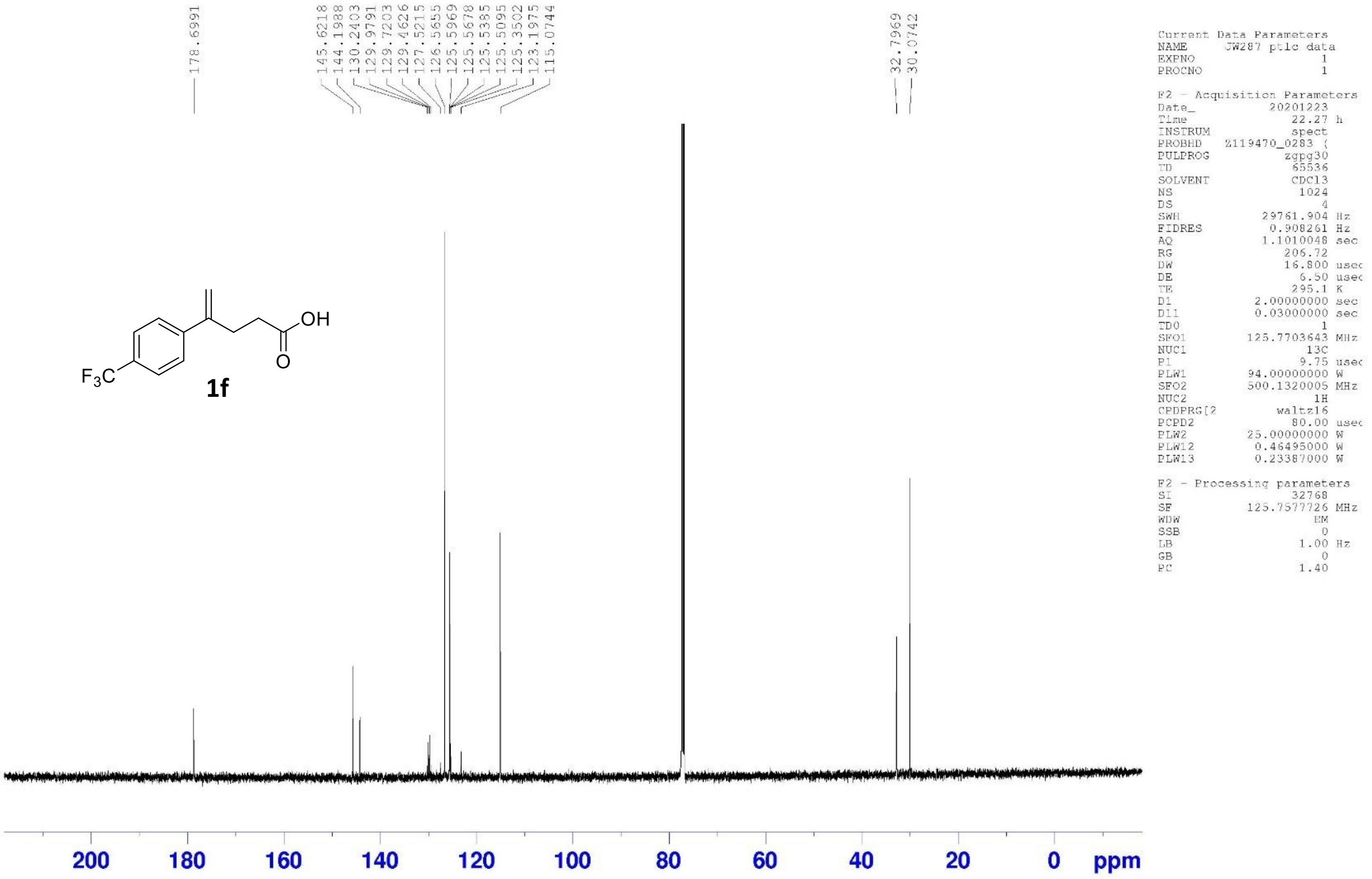
178.6991
145.6218
144.1988
130.2403
129.9791
129.7203
129.4626
127.5215
126.5655
125.5969
125.5678
125.5385
125.5095
125.3502
123.1975
115.0744
32.7969
30.0742
OH
O
F3C
1f
Current Data Parameters
NAME JW287 ptlc data
EXPNO 1
PROCNO 1
F2 - Acquisition Parameters
Date_ 20201223
Time 22.27 h
INSTRUM spect
PROBHD Z119470_0283 (
PULPROG zgpg30
TD 65536
SOLVENT CDCl3
NS 1024
DS 4
SWH 29761.904 Hz
FIDRES 0.908261 Hz
AQ 1.1010048 sec
RG 206.72
DW 16.800 usec
DE 6.50 usec
TE 295.1 K
D1 2.00000000 sec
D11 0.03000000 sec
TD0 1
SFO1 125.7703643 MHz
NUC1 13C
P1 9.75 usec
PLW1 94.00000000 W
SFO2 500.1320005 MHz
NUC2 1H
CPDPRG[2 waltz16
PCPD2 80.00 usec
PLW2 25.00000000 W
PLW12 0.46495000 W
PLW13 0.23387000 W
F2 - Processing parameters
SI 32768
SF 125.7577726 MHz
WDW EM
SSB 0
LB 1.00 Hz
GB 0
PC 1.40
200
180
160
140
120
100
80
60
40
20
0
ppm

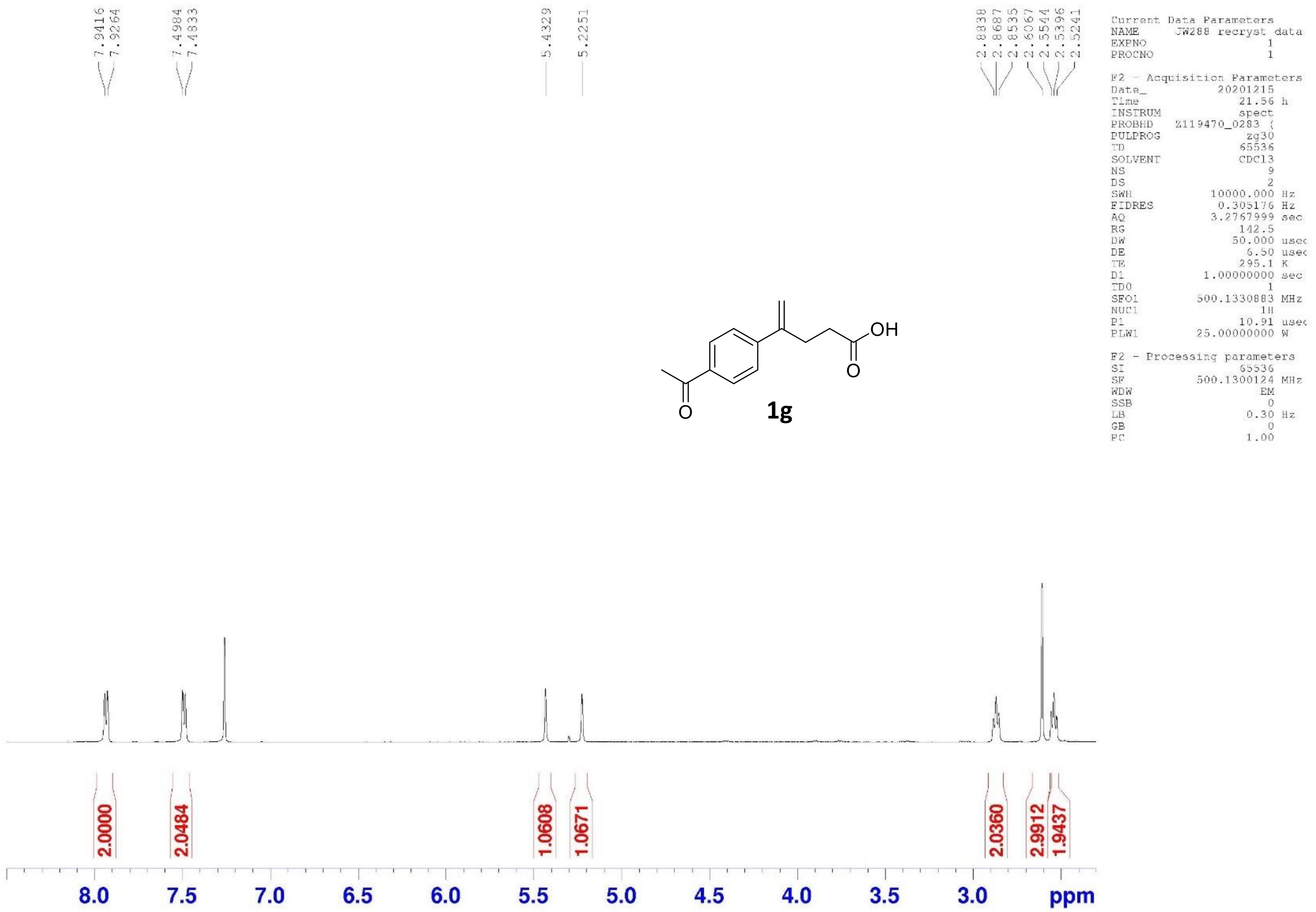
7.9416
7.9264
7.4984
7.4833
5.4329
5.2251
2.8838
2.8687
2.8535
2.6067
2.5544
2.5396
2.5241
Current Data Parameters
NAME JW288 recryst data
EXPNO 1
PROCNO 1
F2 - Acquisition Parameters
Date_ 20201215
Time 21.56 h
INSTRUM spect
PROBHD Z119470_0283 (
PULPROG zg30
TD 65536
SOLVENT CDCl3
NS 9
DS 2
SWH 10000.000 Hz
FIDRES 0.305176 Hz
AQ 3.2767999 sec
RG 142.5
DW 50.000 usec
DE 6.50 usec
TE 295.1 K
D1 1.00000000 sec
TD0 1
SFO1 500.1330883 MHz
NUC1 1H
P1 10.91 usec
PLW1 25.00000000 W
F2 - Processing parameters
SI 65536
SF 500.1300124 MHz
WDW EM
SSB 0
LB 0.30 Hz
GB 0
PC 1.00
OH
O
O
1g
2.0000
2.0484
1.0608
1.0671
2.0360
2.9912
1.9437
8.0
7.5
7.0
6.5
6.0
5.5
5.0
4.5
4.0
3.5
3.0
ppm

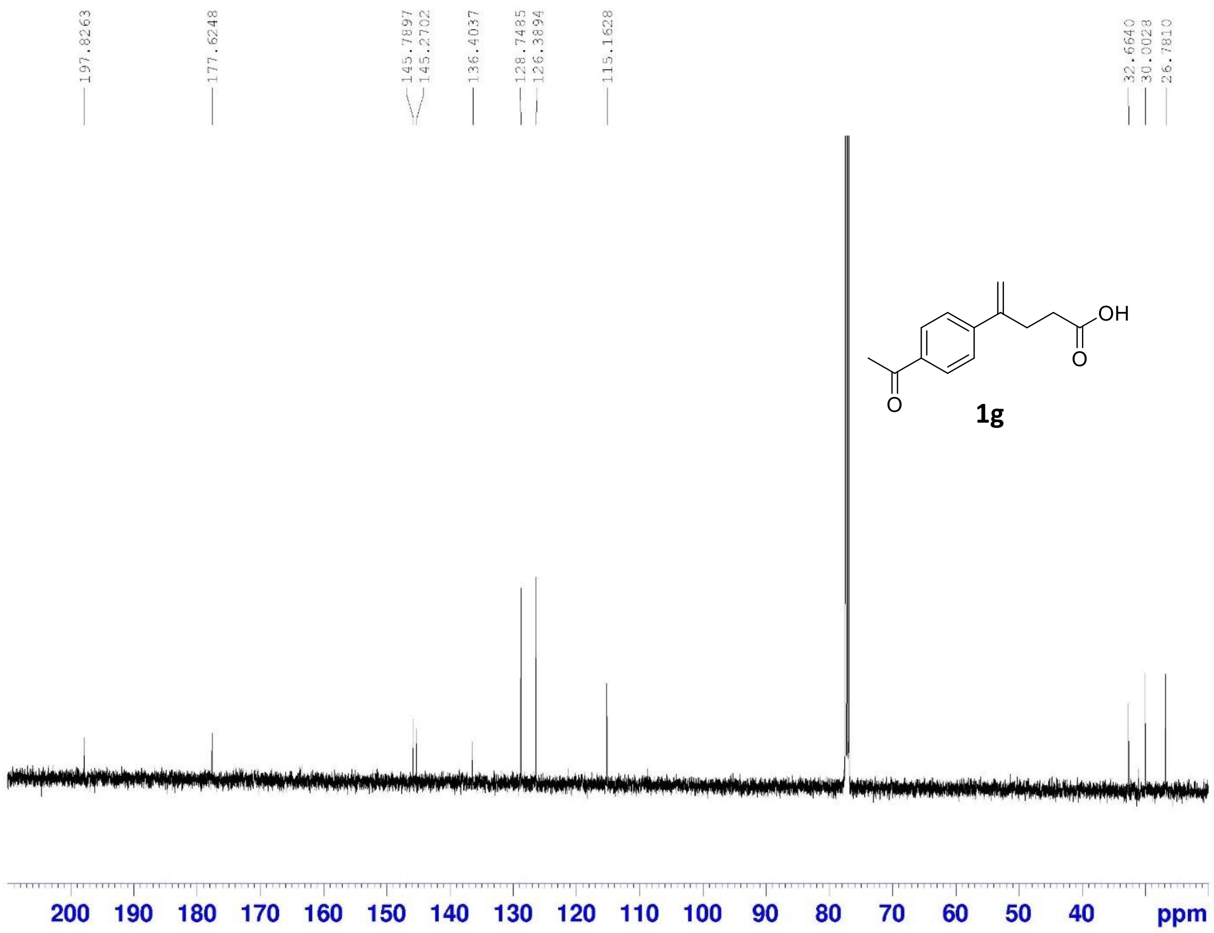

Current Data Parameters
NAME JW288 recryst data
EXPNO 2
PROCNO 1

F2 - Acquisition Parameters
Date_ 20201215
Time 22.33 h
INSTRUM spect
PROBHD Z119470_0283 (
PULPROG zgpg30
TD 65536
SOLVENT CDCl3
NS 700
DS 4
SWH 29761.904 Hz
FIDRES 0.908261 Hz
AQ 1.1010048 sec
RG 206.72
DW 16.800 usec
DE 6.50 usec
TE 295.2 K
D1 2.00000000 sec
D11 0.03000000 sec
TD0 1
SFO1 125.7703643 MHz
NUC1 13C
P1 9.75 usec
PLW1 94.00000000 W
SFO2 500.1320005 MHz
NUC2 1H
CPDPRG[2 waltz16
PCPD2 80.00 usec
PLW2 25.00000000 W
PLW12 0.46495000 W
PLW13 0.23387000 W

F2 - Processing parameters
SI 32768
SF 125.7577726 MHz
WDW EM
SSB 0
LB 1.00 Hz
GB 0
PC 1.40

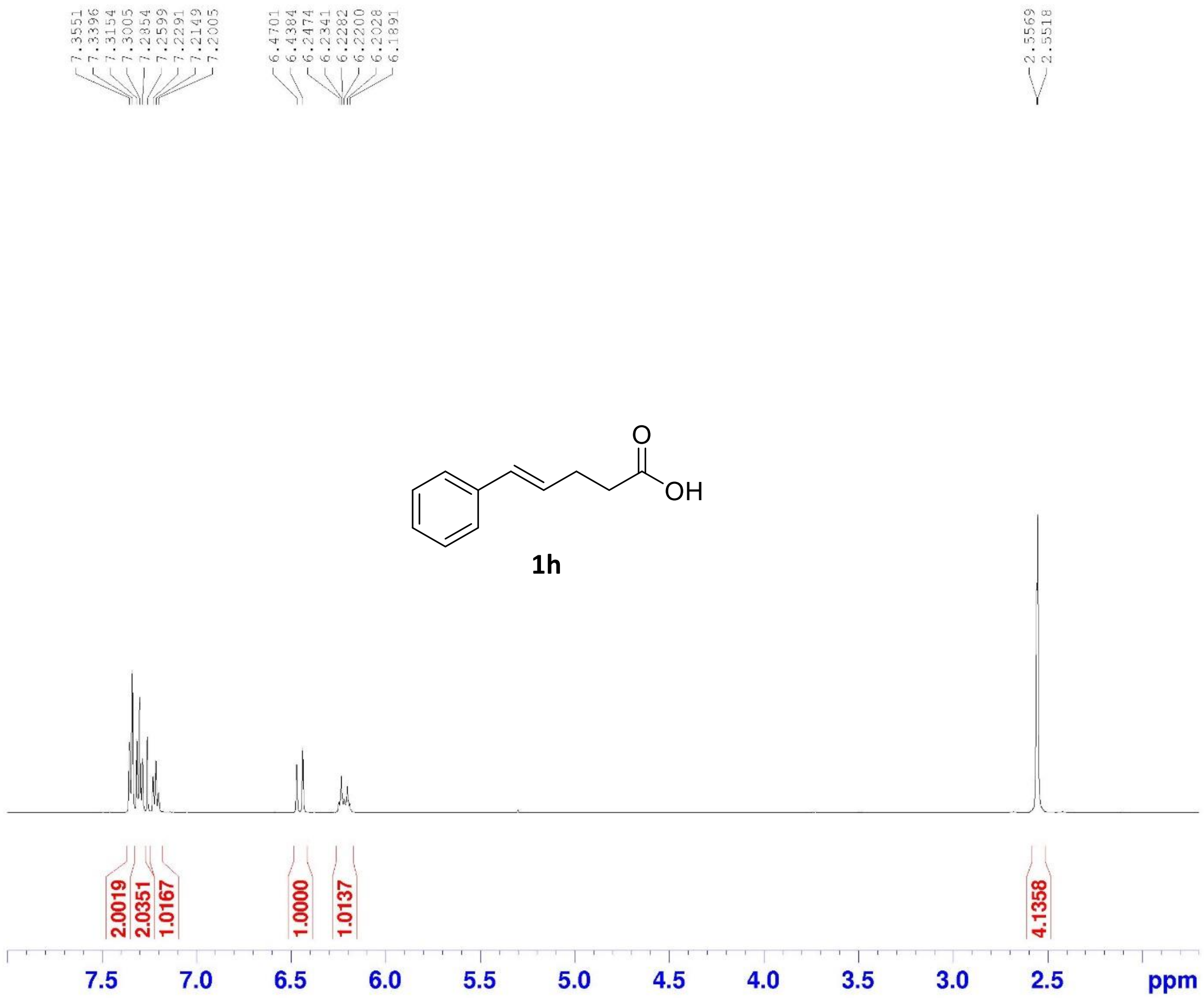

Current Data Parameters
NAME JW312 data
EXPNO 2
PROCNO 1

F2 - Acquisition Parameters
Date_ 20210113
Time 21.33 h
INSTRUM spect
PROBHD Z119470_0283 (
PULPROG zg30
TD 65536
SOLVENT CDCl3
NS 16
DS 2
SWH 10000.000 Hz
FIDRES 0.305176 Hz
AQ 3.2767999 sec
RG 102.6
DW 50.000 usec
DE 6.50 usec
TE 295.2 K
D1 1.00000000 sec
TD0 1
SFO1 500.1330883 MHz
NUC1 1H
P1 10.91 usec
PLW1 25.00000000 W

F2 - Processing parameters
SI 65536
SF 500.1300121 MHz
WDW EM
SSB 0
LB 0.30 Hz
GB 0
PC 1.00

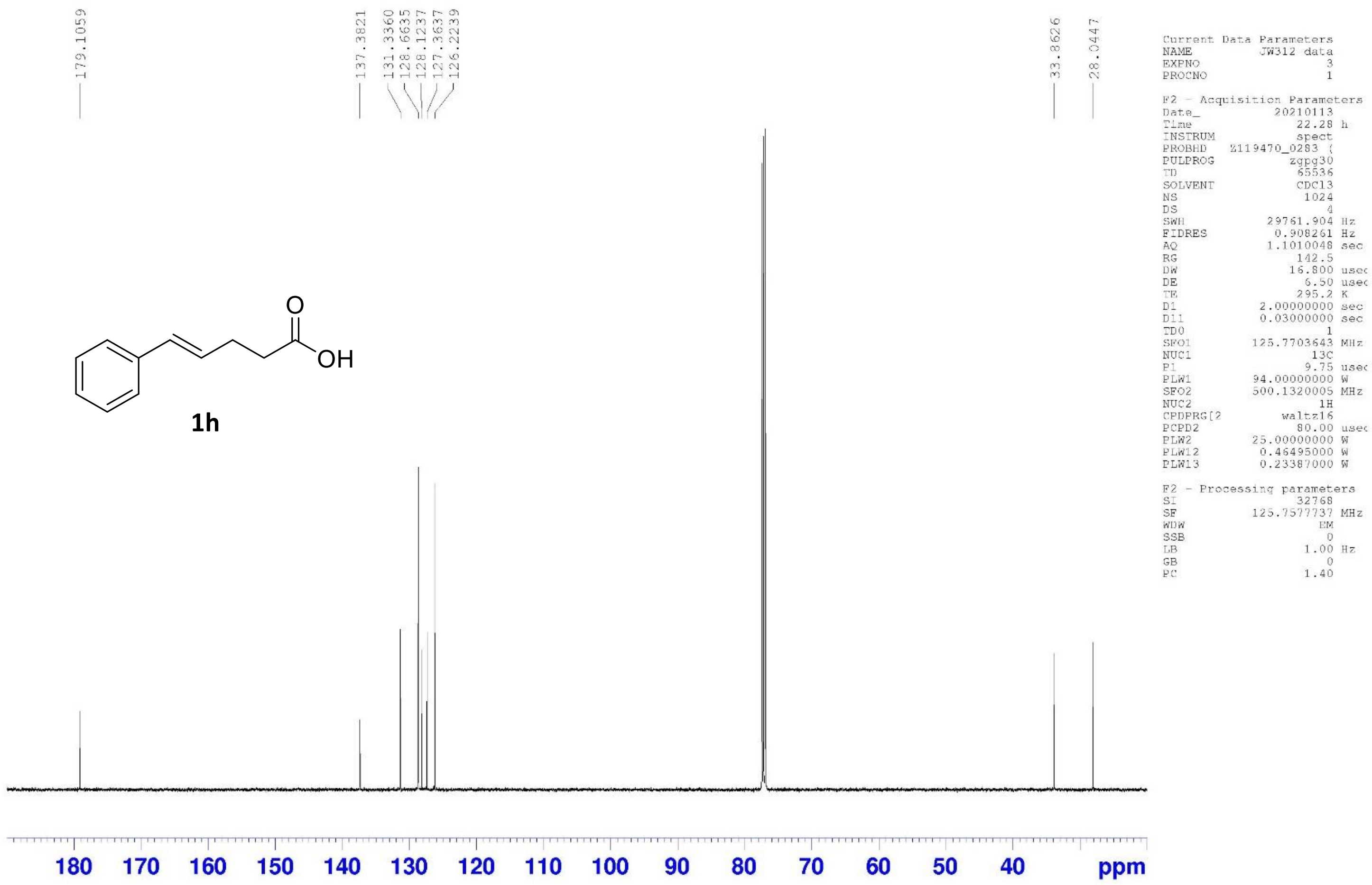

179.1059
137.3821
131.3360
128.6635
128.1237
127.3637
126.2239
33.8626
28.0447
OH
O
1h
180 170 160 150 140 130 120 110 100 90 80 70 60 50 40 ppm
Current Data Parameters
NAME JW312 data
EXPNO 3
PROCNO 1
F2 - Acquisition Parameters
Date_ 20210113
Time 22.28 h
INSTRUM spect
PROBHD Z119470_0283 (
PULPROG zgpg30
TD 65536
SOLVENT CDCl3
NS 1024
DS 4
SWH 29761.904 Hz
FIDRES 0.908261 Hz
AQ 1.1010048 sec
RG 142.5
DW 16.800 usec
DE 6.50 usec
TE 295.2 K
D1 2.00000000 sec
D11 0.03000000 sec
TD0 1
SFO1 125.7703643 MHz
NUC1 13C
P1 9.75 usec
PLW1 94.00000000 W
SFO2 500.1320005 MHz
NUC2 1H
CPDPRG[2 waltz16
PCPD2 80.00 usec
PLW2 25.00000000 W
PLW12 0.46495000 W
PLW13 0.23387000 W
F2 - Processing parameters
SI 32768
SF 125.7577737 MHz
WDW EM
SSB 0
LB 1.00 Hz
GB 0
PC 1.40

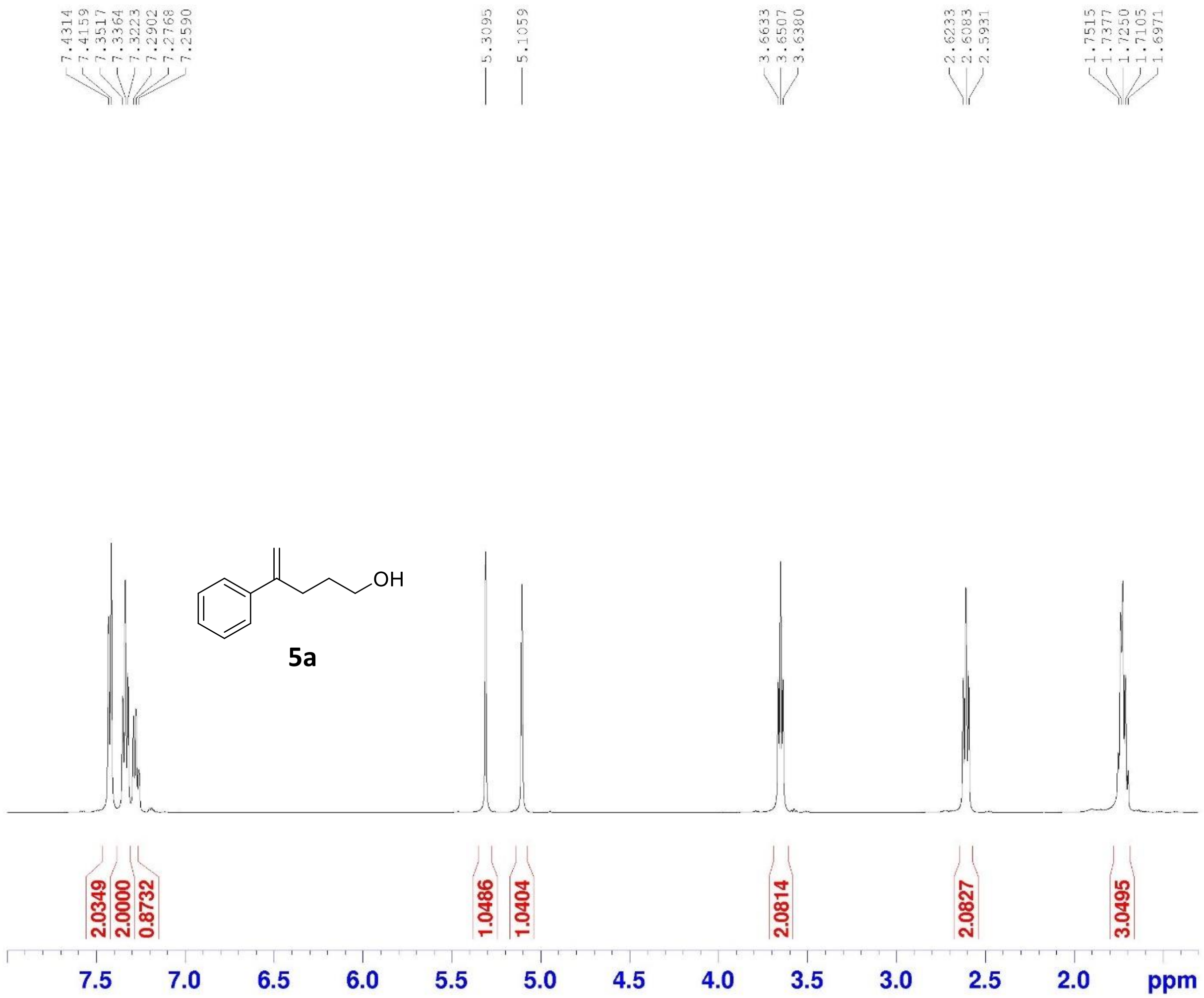


```
Current Data Parameters
NAME          JW340 data
EXPNO                  1
PROCNO                 1

F2 - Acquisition Parameters
Date_           20201202
Time               20.34 h
INSTRUM            spect
PROBHD   Z119470_0283 (
PULPROG             zg30
TD                 65536
SOLVENT            CDCl3
NS                    16
DS                     2
SWH            10000.000 Hz
FIDRES          0.305176 Hz
AQ             3.2767999 sec
RG                 30.85
DW                50.000 usec
DE                  6.50 usec
TE                 295.2 K
D1            1.00000000 sec
TD0                    1
SFO1         500.1330883 MHz
NUC1                  1H
P1                 10.91 usec
PLW1         25.00000000 W

F2 - Processing parameters
SI                 65536
SF           500.1300147 MHz
WDW                   EM
SSB                    0
LB                  0.30 Hz
GB                     0
PC                  1.00
```

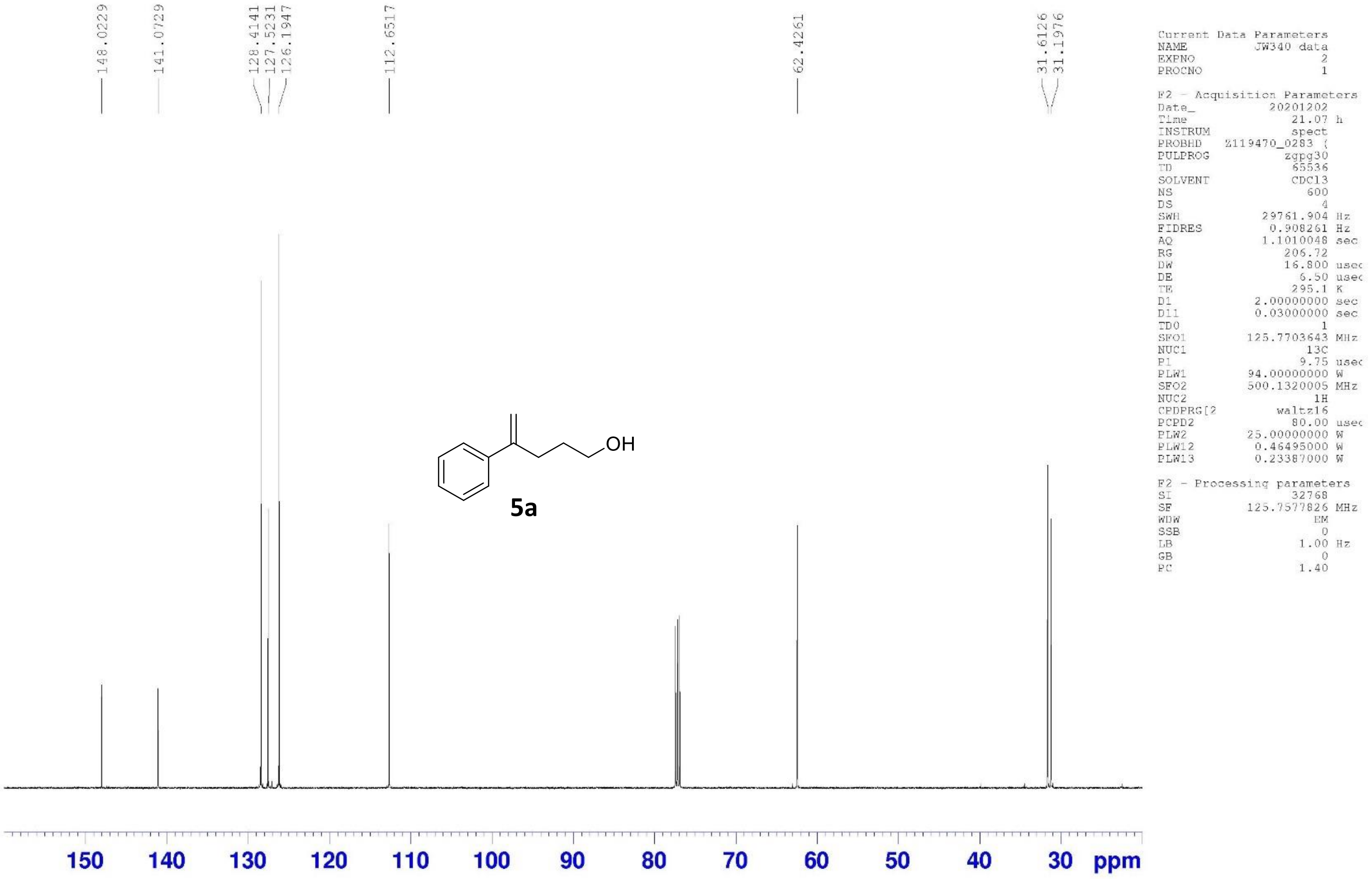
148.0229
141.0729
128.4141
127.5231
126.1947
112.6517
62.4261
31.6126
31.1976
OH
5a
150 140 130 120 110 100 90 80 70 60 50 40 30 ppm
Current Data Parameters
NAME JW340 data
EXPNO 2
PROCNO 1
F2 - Acquisition Parameters
Date_ 20201202
Time 21.07 h
INSTRUM spect
PROBHD Z119470_0283 (
PULPROG zgpg30
TD 65536
SOLVENT CDCl3
NS 600
DS 4
SWH 29761.904 Hz
FIDRES 0.908261 Hz
AQ 1.1010048 sec
RG 206.72
DW 16.800 usec
DE 6.50 usec
TE 295.1 K
D1 2.00000000 sec
D11 0.03000000 sec
TD0 1
SFO1 125.7703643 MHz
NUC1 13C
P1 9.75 usec
PLW1 94.00000000 W
SFO2 500.1320005 MHz
NUC2 1H
CPDPRG[2 waltz16
PCPD2 80.00 usec
PLW2 25.00000000 W
PLW12 0.46495000 W
PLW13 0.23387000 W
F2 - Processing parameters
SI 32768
SF 125.7577826 MHz
WDW EM
SSB 0
LB 1.00 Hz
GB 0
PC 1.40

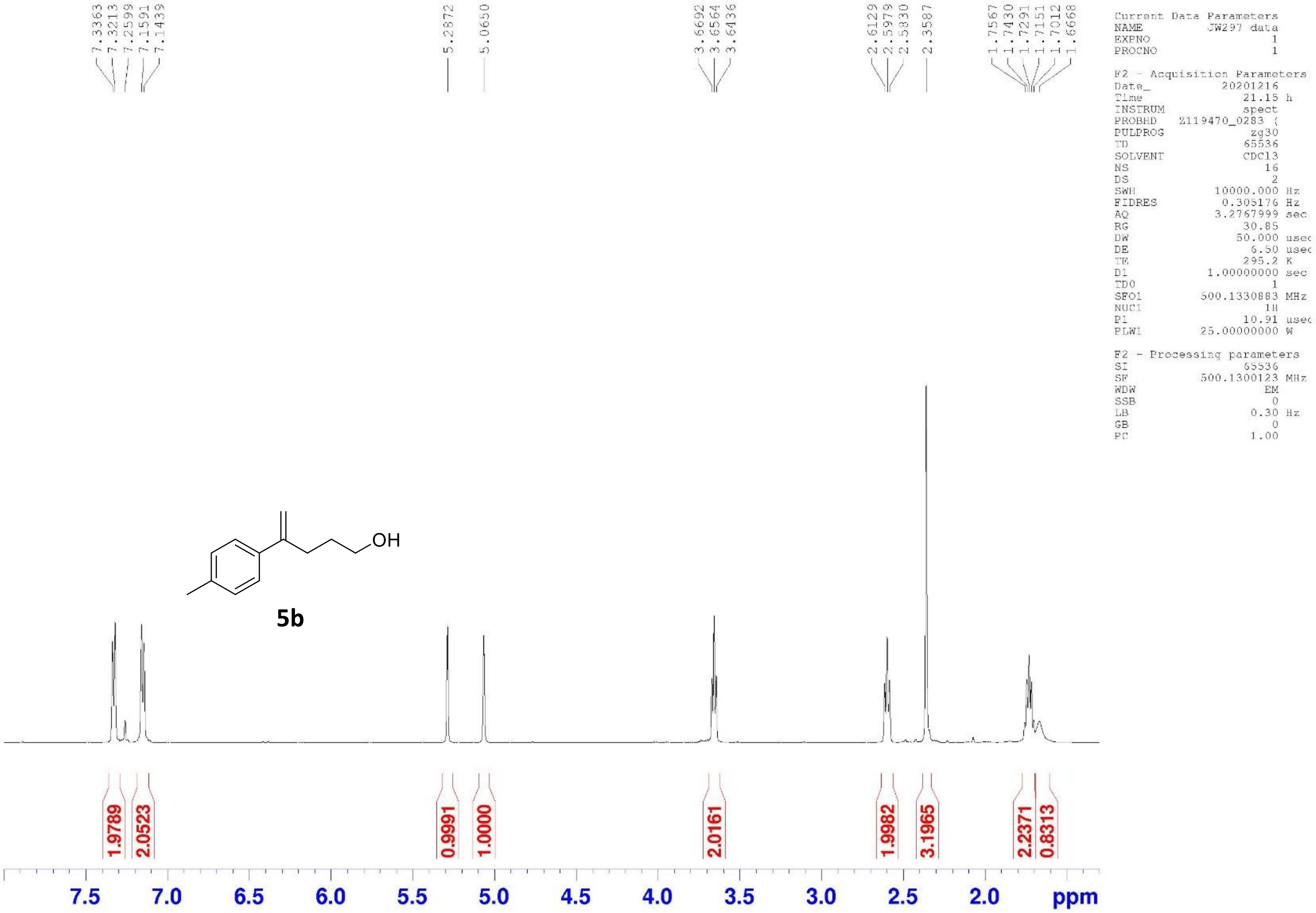
7.3363
7.3213
7.2599
7.1591
7.1439
5.2872
5.0650
3.6692
3.6564
3.6436
2.6129
2.5979
2.5830
2.3587
1.7567
1.7430
1.7291
1.7151
1.7012
1.6668
Current Data Parameters
NAME JW297 data
EXPNO 1
PROCNO 1
F2 - Acquisition Parameters
Date_ 20201216
Time 21.15 h
INSTRUM spect
PROBHD Z119470_0283 (
PULPROG zg30
TD 65536
SOLVENT CDCl3
NS 16
DS 2
SWH 10000.000 Hz
FIDRES 0.305176 Hz
AQ 3.2767999 sec
RG 30.85
DW 50.000 usec
DE 6.50 usec
TE 295.2 K
D1 1.00000000 sec
TD0 1
SFO1 500.1330883 MHz
NUC1 1H
P1 10.91 usec
PLW1 25.00000000 W
F2 - Processing parameters
SI 65536
SF 500.1300123 MHz
WDW EM
SSB 0
LB 0.30 Hz
GB 0
PC 1.00
OH
5b
1.9789
2.0523
0.9991
1.0000
2.0161
1.9982
3.1965
2.2371
0.8313
7.5
7.0
6.5
6.0
5.5
5.0
4.5
4.0
3.5
3.0
2.5
2.0
ppm

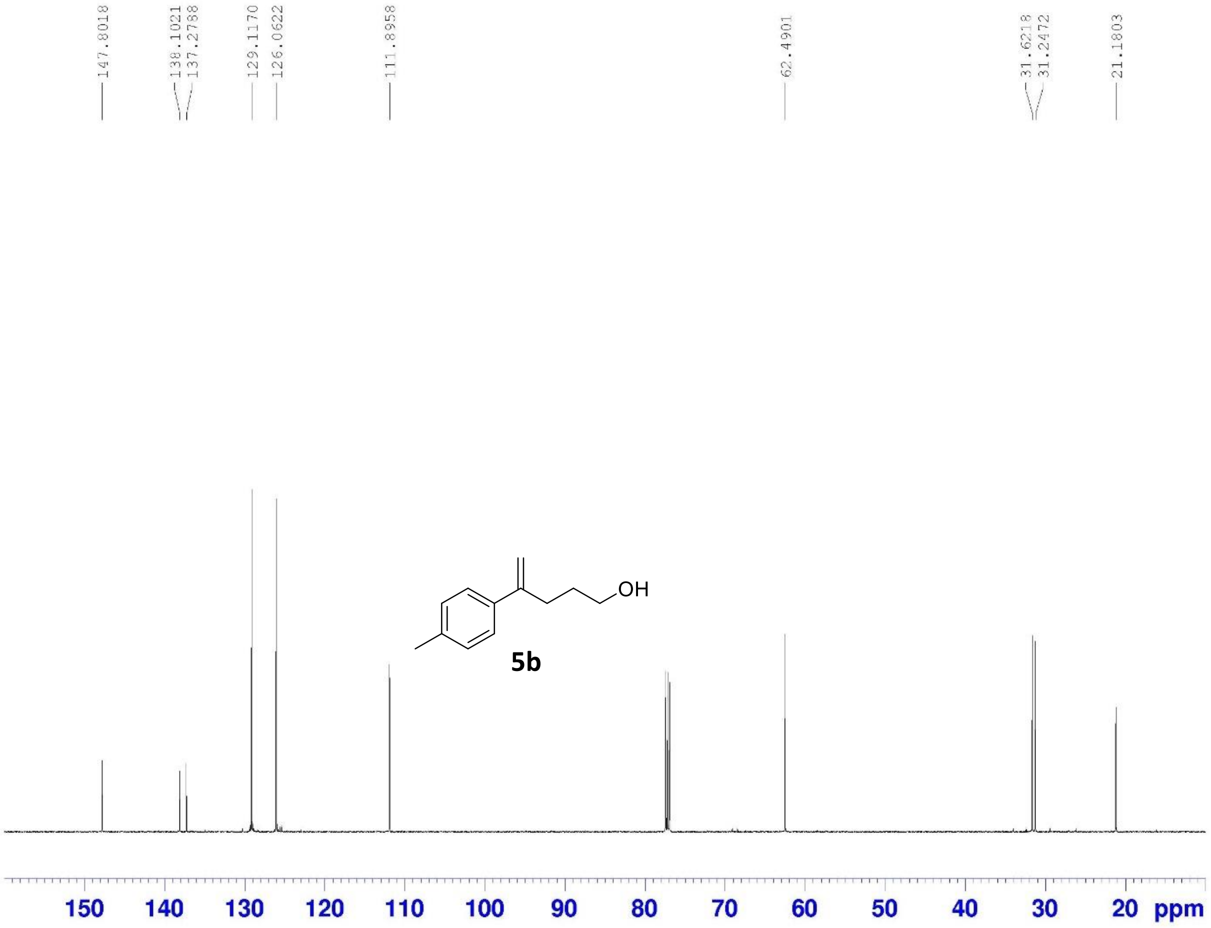

Current Data Parameters		
NAME	JW297 data	
EXPNO	2	
PROCNO	1	
F2 - Acquisition Parameters		
Date_	20201216	
Time	21.52	h
INSTRUM	spect	
PROBHD	Z119470_0283 (	
PULPROG	zgpg30	
TD	65536	
SOLVENT	CDCl3	
NS	700	
DS	4	
SWH	29761.904	Hz
FIDRES	0.908261	Hz
AQ	1.1010048	sec
RG	206.72	
DW	16.800	usec
DE	6.50	usec
TE	295.2	K
D1	2.00000000	sec
D11	0.03000000	sec
TD0	1	
SFO1	125.7703643	MHz
NUC1	13C	
P1	9.75	usec
PLW1	94.00000000	W
SFO2	500.1320005	MHz
NUC2	1H	
CPDPRG[2	waltz16	
PCPD2	80.00	usec
PLW2	25.00000000	W
PLW12	0.46495000	W
PLW13	0.23387000	W
F2 - Processing parameters		
SI	32768	
SF	125.7577814	MHz
WDW	EM	
SSB	0	
LB	1.00	Hz
GB	0	
PC	1.40	

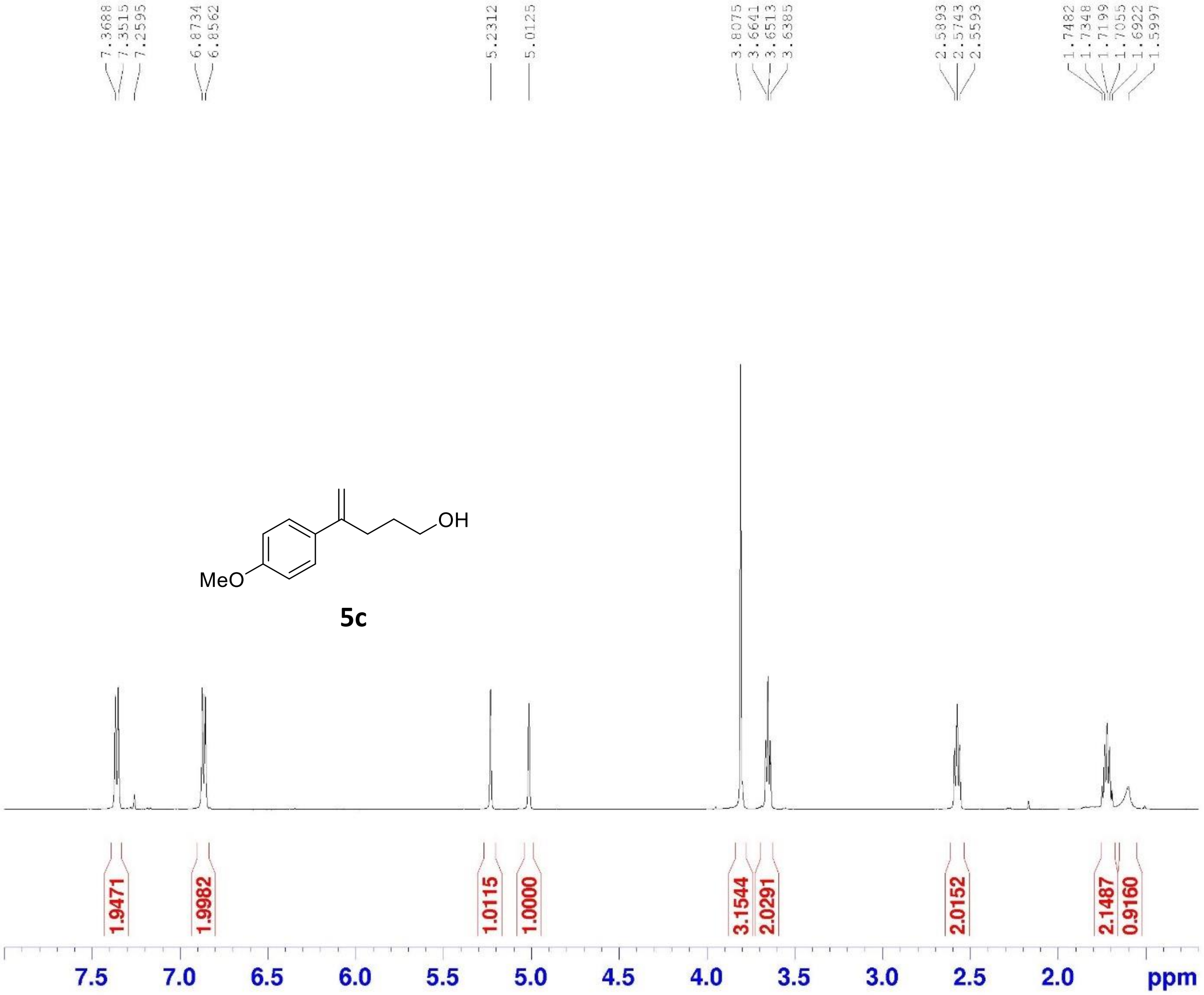


```
Current Data Parameters
NAME          JW294 data
EXPNO                  1
PROCNO                 1

F2 - Acquisition Parameters
Date_           20201211
Time                0.39 h
INSTRUM            spect
PROBHD   Z119470_0283 (
PULPROG             zg30
TD                 65536
SOLVENT            CDCl3
NS                    16
DS                     2
SWH            10000.000 Hz
FIDRES          0.305176 Hz
AQ             3.2767999 sec
RG                 30.85
DW                50.000 usec
DE                  6.50 usec
TE                 295.1 K
D1            1.00000000 sec
TD0                    1
SFO1         500.1330883 MHz
NUC1                  1H
P1                 10.91 usec
PLW1         25.00000000 W

F2 - Processing parameters
SI                 65536
SF           500.1300128 MHz
WDW                   EM
SSB                    0
LB                  0.30 Hz
GB                     0
PC                  1.00
```

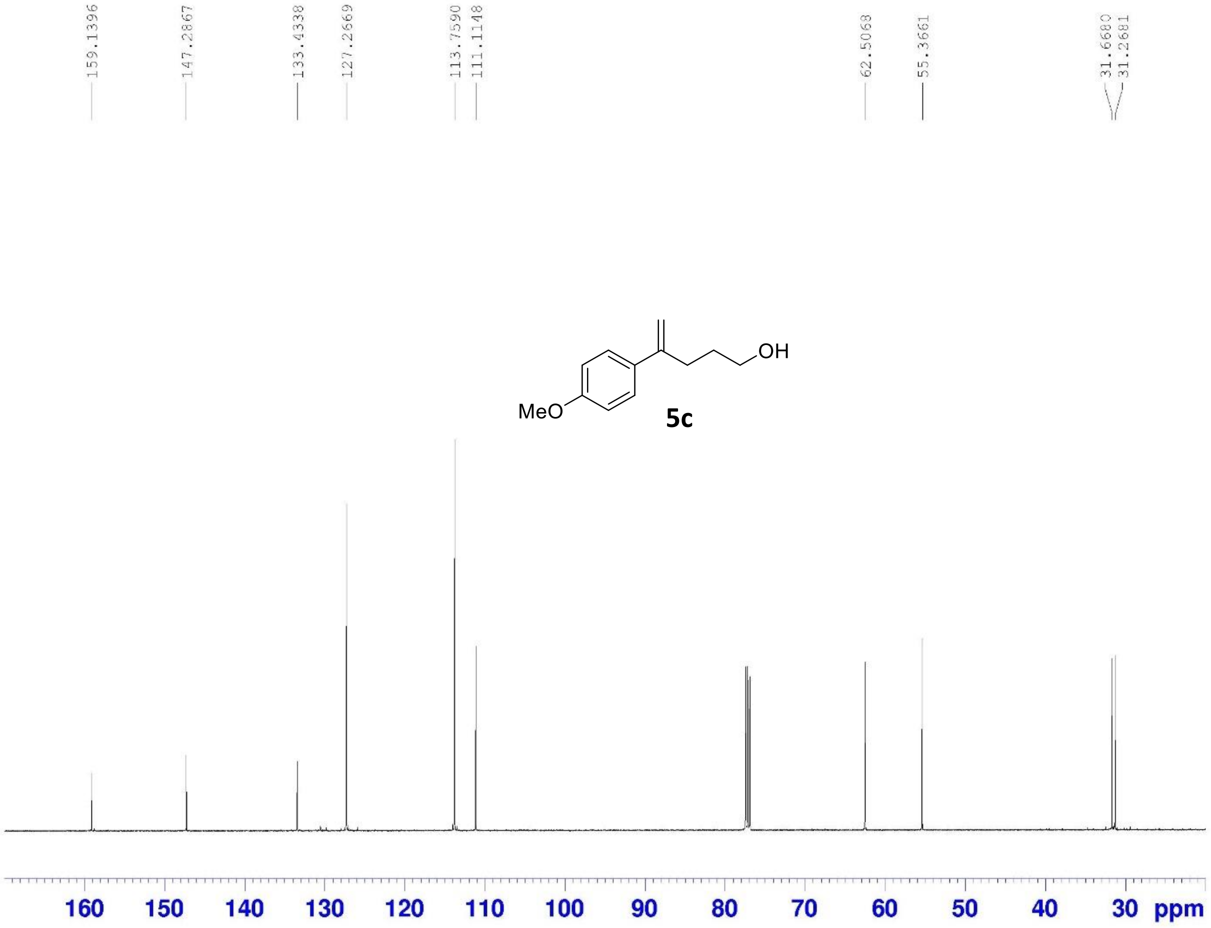

Current Data Parameters
NAME JW294 data
EXPNO 2
PROCNO 1

F2 - Acquisition Parameters
Date_ 20201211
Time 1.33 h
INSTRUM spect
PROBHD Z119470_0283 (
PULPROG zgpg30
TD 65536
SOLVENT CDCl3
NS 1024
DS 4
SWH 29761.904 Hz
FIDRES 0.908261 Hz
AQ 1.1010048 sec
RG 206.72
DW 16.800 usec
DE 6.50 usec
TE 295.2 K
D1 2.00000000 sec
D11 0.03000000 sec
TD0 1
SFO1 125.7703643 MHz
NUC1 13C
P1 9.75 usec
PLW1 94.00000000 W
SFO2 500.1320005 MHz
NUC2 1H
CPDPRG[2 waltz16
PCPD2 80.00 usec
PLW2 25.00000000 W
PLW12 0.46495000 W
PLW13 0.23387000 W

F2 - Processing parameters
SI 32768
SF 125.7577798 MHz
WDW EM
SSB 0
LB 1.00 Hz
GB 0
PC 1.40

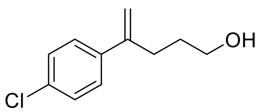

Cl
OH

5d

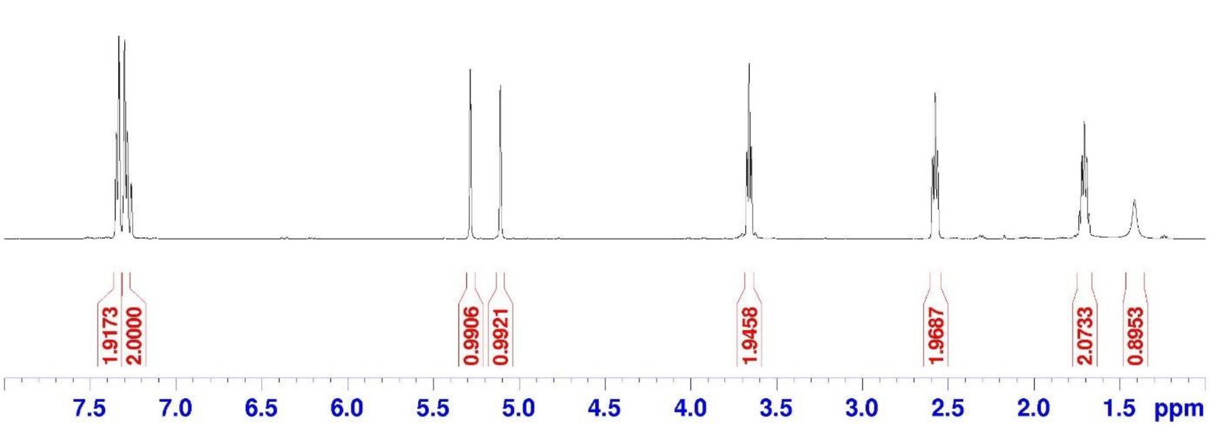

7.3480
7.3325
7.2994
7.2833
7.2598
5.2843
5.1091
3.6720
3.6594
3.6467
2.5907
2.5756
2.5606
1.7335
1.7196
1.7056
1.6917
1.6782
1.4127
Current Data Parameters
NAME JW298 data
EXPNO 1
PROCNO 1
F2 - Acquisition Parameters
Date_ 20201216
Time 21.56 h
INSTRUM spect
PROBHD Z119470_0283 (
PULPROG zg30
TD 65536
SOLVENT CDCl3
NS 16
DS 2
SWH 10000.000 Hz
FIDRES 0.305176 Hz
AQ 3.2767999 sec
RG 71.76
DW 50.000 usec
DE 6.50 usec
TE 295.2 K
D1 1.00000000 sec
TD0 1
SFO1 500.1330883 MHz
NUC1 1H
P1 10.91 usec
PLW1 25.00000000 W
F2 - Processing parameters
SI 65536
SF 500.1300114 MHz
WDW EM
SSB 0
LB 0.30 Hz
GB 0
PC 1.00
1.9173
2.0000
0.9906
0.9921
1.9458
1.9687
2.0733
0.8953
7.5
7.0
6.5
6.0
5.5
5.0
4.5
4.0
3.5
3.0
2.5
2.0
1.5
ppm

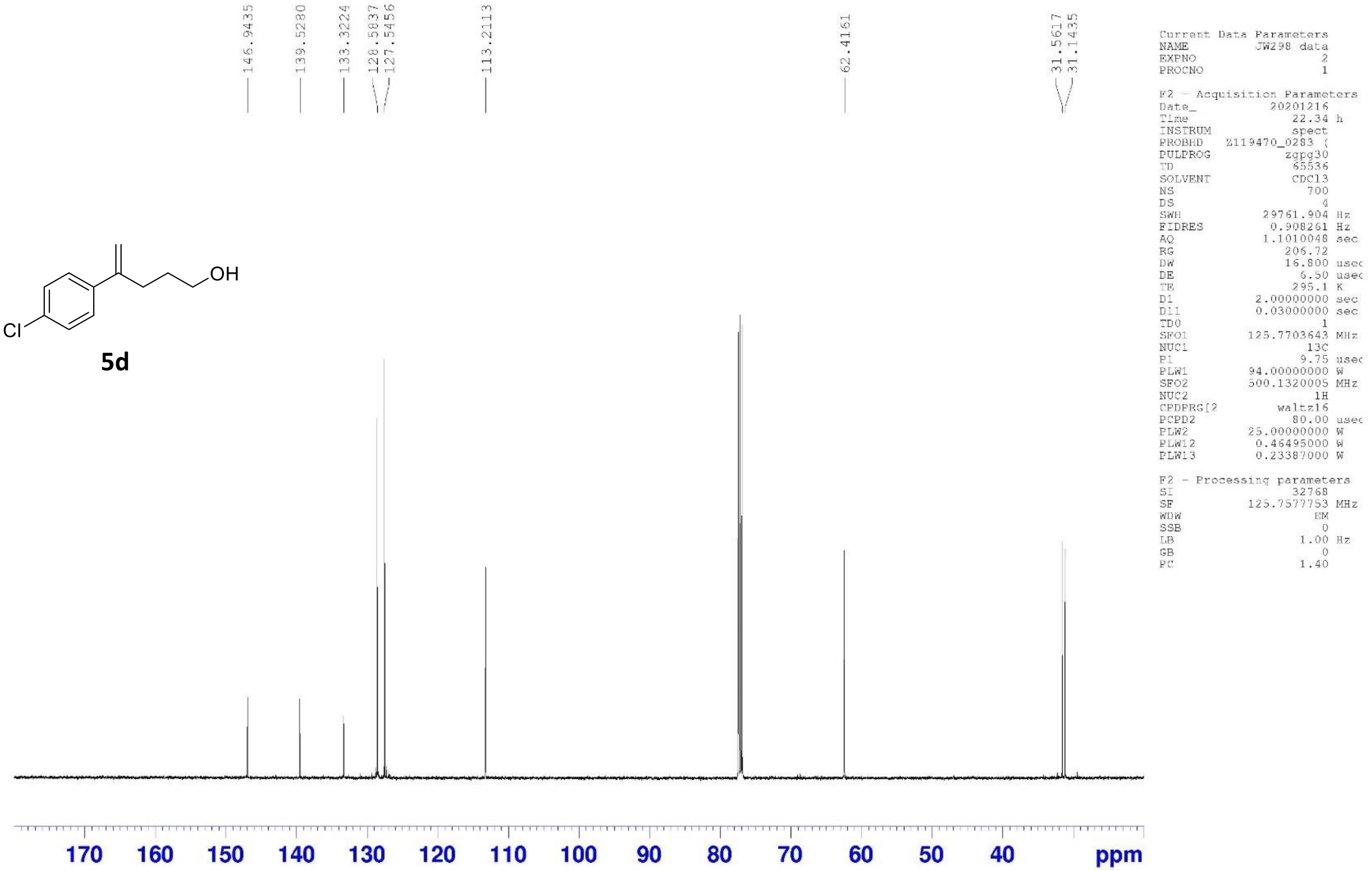
OH
Cl
146.9435
139.5280
133.3224
128.5837
127.5456
113.2113
62.4161
31.5617
31.1435
170 160 150 140 130 120 110 100 90 80 70 60 50 40 ppm
Current Data Parameters
NAME JW298 data
EXPNO 2
PROCNO 1
F2 - Acquisition Parameters
Date_ 20201216
Time 22.34 h
INSTRUM spect
PROBHD Z119470_0283 (
PULPROG zgpg30
TD 65536
SOLVENT CDCl3
NS 700
DS 4
SWH 29761.904 Hz
FIDRES 0.908261 Hz
AQ 1.1010048 sec
RG 206.72
DW 16.800 usec
DE 6.50 usec
TE 295.1 K
D1 2.00000000 sec
D11 0.03000000 sec
TD0 1
SFO1 125.7703643 MHz
NUC1 13C
P1 9.75 usec
PLW1 94.00000000 W
SFO2 500.1320005 MHz
NUC2 1H
CPDPRG[2 waltz16
PCPD2 80.00 usec
PLW2 25.00000000 W
PLW12 0.46495000 W
PLW13 0.23387000 W
F2 - Processing parameters
SI 32768
SF 125.7577753 MHz
WDW EM
SSB 0
LB 1.00 Hz
GB 0
PC 1.40

5d

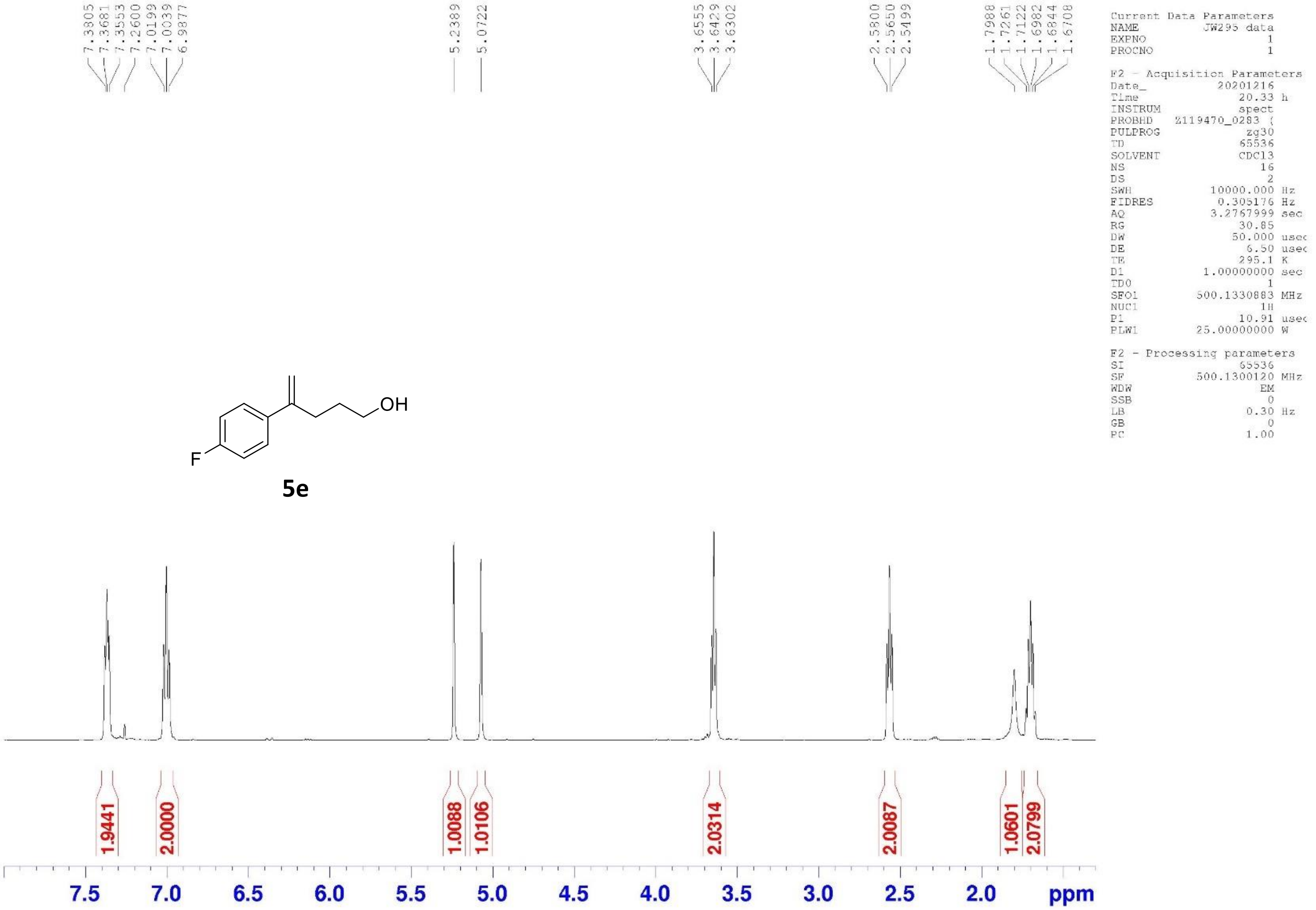
7.3805
7.3681
7.3553
7.2600
7.0199
7.0039
6.9877
5.2389
5.0722
3.6555
3.6429
3.6302
2.5800
2.5650
2.5499
1.7988
1.7261
1.7122
1.6982
1.6844
1.6708
Current Data Parameters
NAME JW295 data
EXPNO 1
PROCNO 1
F2 - Acquisition Parameters
Date_ 20201216
Time 20.33 h
INSTRUM spect
PROBHD Z119470_0283 (
PULPROG zg30
TD 65536
SOLVENT CDCl3
NS 16
DS 2
SWH 10000.000 Hz
FIDRES 0.305176 Hz
AQ 3.2767999 sec
RG 30.85
DW 50.000 usec
DE 6.50 usec
TE 295.1 K
D1 1.00000000 sec
TD0 1
SFO1 500.1330883 MHz
NUC1 1H
P1 10.91 usec
PLW1 25.00000000 W
F2 - Processing parameters
SI 65536
SF 500.1300120 MHz
WDW EM
SSB 0
LB 0.30 Hz
GB 0
PC 1.00
OH
F
1.9441
2.0000
1.0088
1.0106
2.0314
2.0087
1.0601
2.0799
7.5
7.0
6.5
6.0
5.5
5.0
4.5
4.0
3.5
3.0
2.5
2.0
ppm

5e

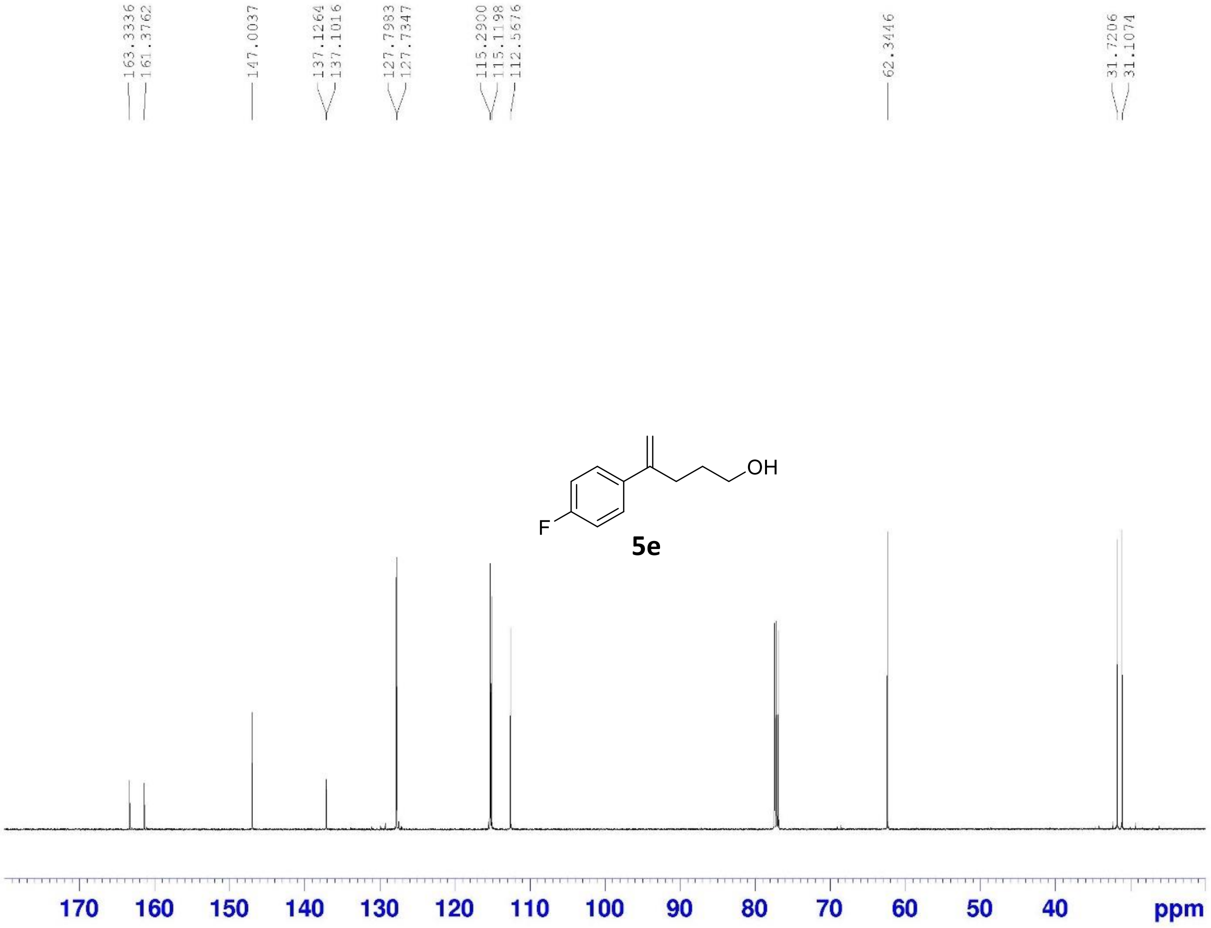

Current Data Parameters		
NAME	JW295 data	
EXPNO	2	
PROCNO	1	
F2 - Acquisition Parameters		
Date_	20201216	
Time	21.11	h
INSTRUM	spect	
PROBHD	Z119470_0283 (	
PULPROG	zgpg30	
TD	65536	
SOLVENT	CDCl3	
NS	700	
DS	4	
SWH	29761.904	Hz
FIDRES	0.908261	Hz
AQ	1.1010048	sec
RG	206.72	
DW	16.800	usec
DE	6.50	usec
TE	295.2	K
D1	2.00000000	sec
D11	0.03000000	sec
TD0	1	
SFO1	125.7703643	MHz
NUC1	13C	
P1	9.75	usec
PLW1	94.00000000	W
SFO2	500.1320005	MHz
NUC2	1H	
CPDPRG[2	waltz16	
PCPD2	80.00	usec
PLW2	25.00000000	W
PLW12	0.46495000	W
PLW13	0.23387000	W
F2 - Processing parameters		
SI	32768	
SF	125.7577798	MHz
WDW	EM	
SSB	0	
LB	1.00	Hz
GB	0	
PC	1.40	

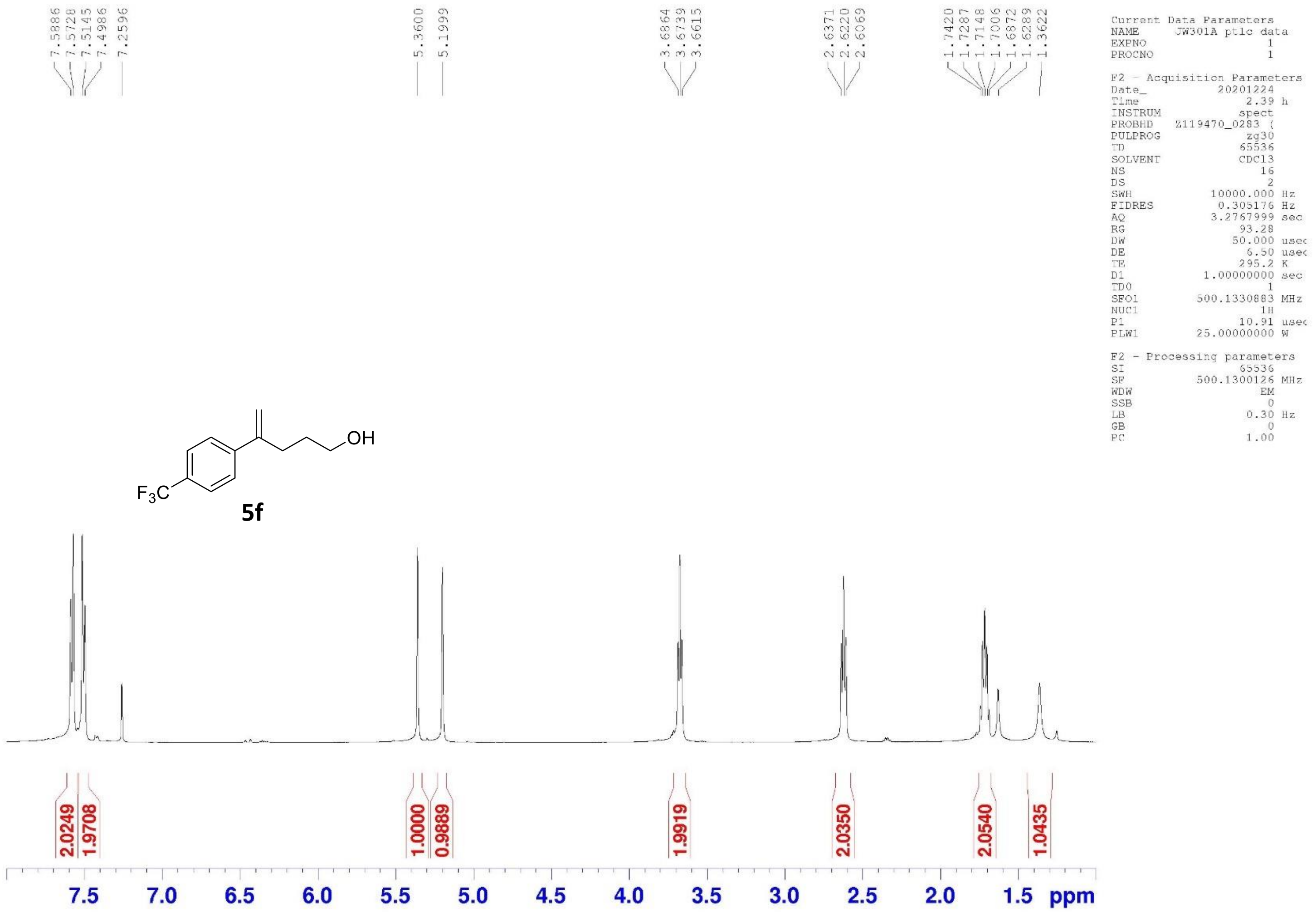
7.5886
7.5728
7.5145
7.4986
7.2596
5.3600
5.1999
3.6864
3.6739
3.6615
2.6371
2.6220
2.6069
1.7420
1.7287
1.7148
1.7006
1.6872
1.6289
1.3622
Current Data Parameters
NAME JW301A ptlc data
EXPNO 1
PROCNO 1
F2 - Acquisition Parameters
Date_ 20201224
Time 2.39 h
INSTRUM spect
PROBHD Z119470_0283 (
PULPROG zg30
TD 65536
SOLVENT CDCl3
NS 16
DS 2
SWH 10000.000 Hz
FIDRES 0.305176 Hz
AQ 3.2767999 sec
RG 93.28
DW 50.000 usec
DE 6.50 usec
TE 295.2 K
D1 1.00000000 sec
TD0 1
SFO1 500.1330883 MHz
NUC1 1H
P1 10.91 usec
PLW1 25.00000000 W
F2 - Processing parameters
SI 65536
SF 500.1300126 MHz
WDW EM
SSB 0
LB 0.30 Hz
GB 0
PC 1.00
OH
F3C
5f
2.0249
1.9708
1.0000
0.9889
1.9919
2.0350
2.0540
1.0435
7.5
7.0
6.5
6.0
5.5
5.0
4.5
4.0
3.5
3.0
2.5
2.0
1.5
ppm

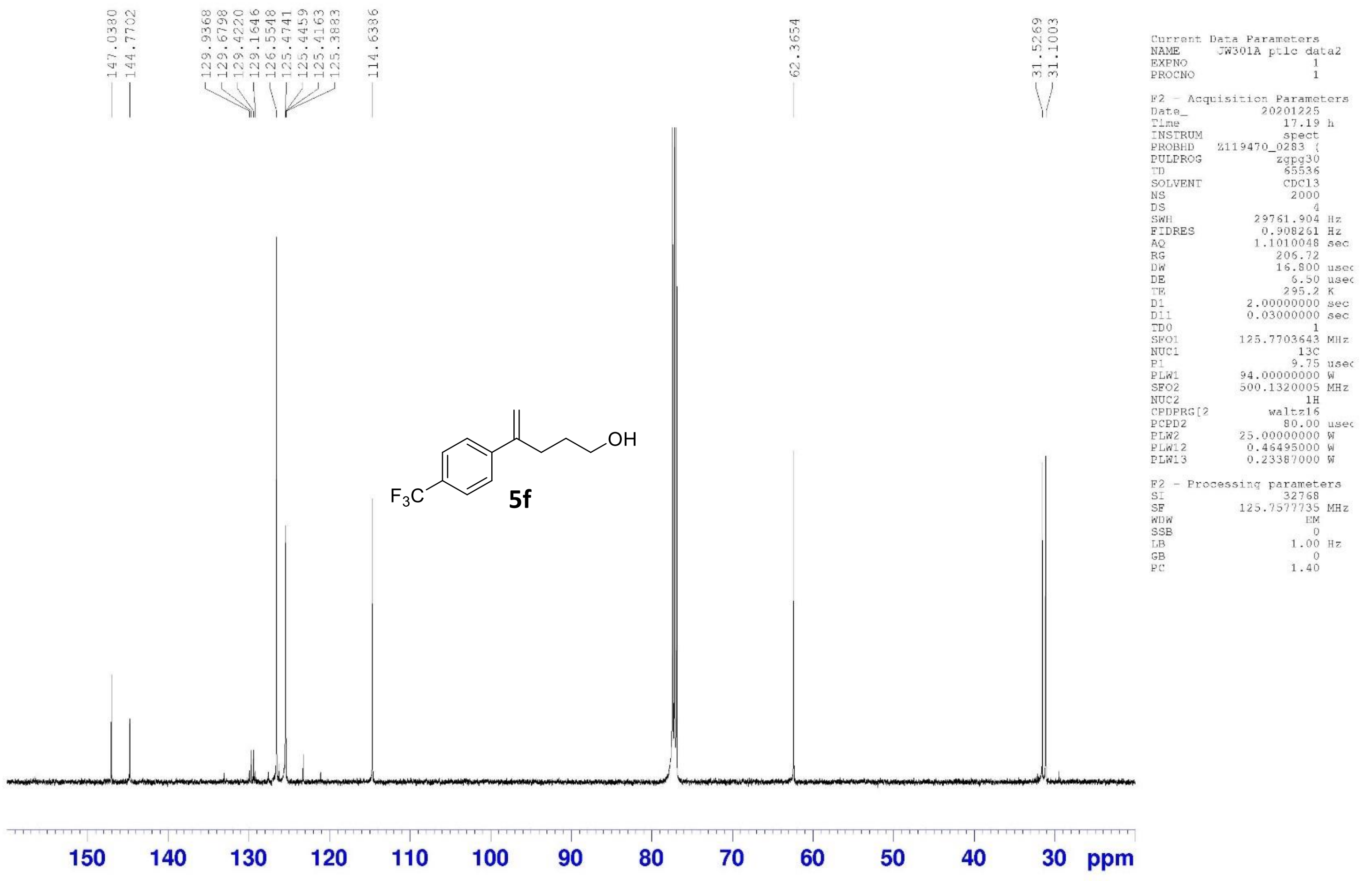

147.0380
144.7702
129.9368
129.6798
129.4220
129.1646
126.5548
125.4741
125.4459
125.4163
125.3883
114.6386
62.3654
31.5269
31.1003
OH
F3C
5f
150
140
130
120
110
100
90
80
70
60
50
40
30
ppm

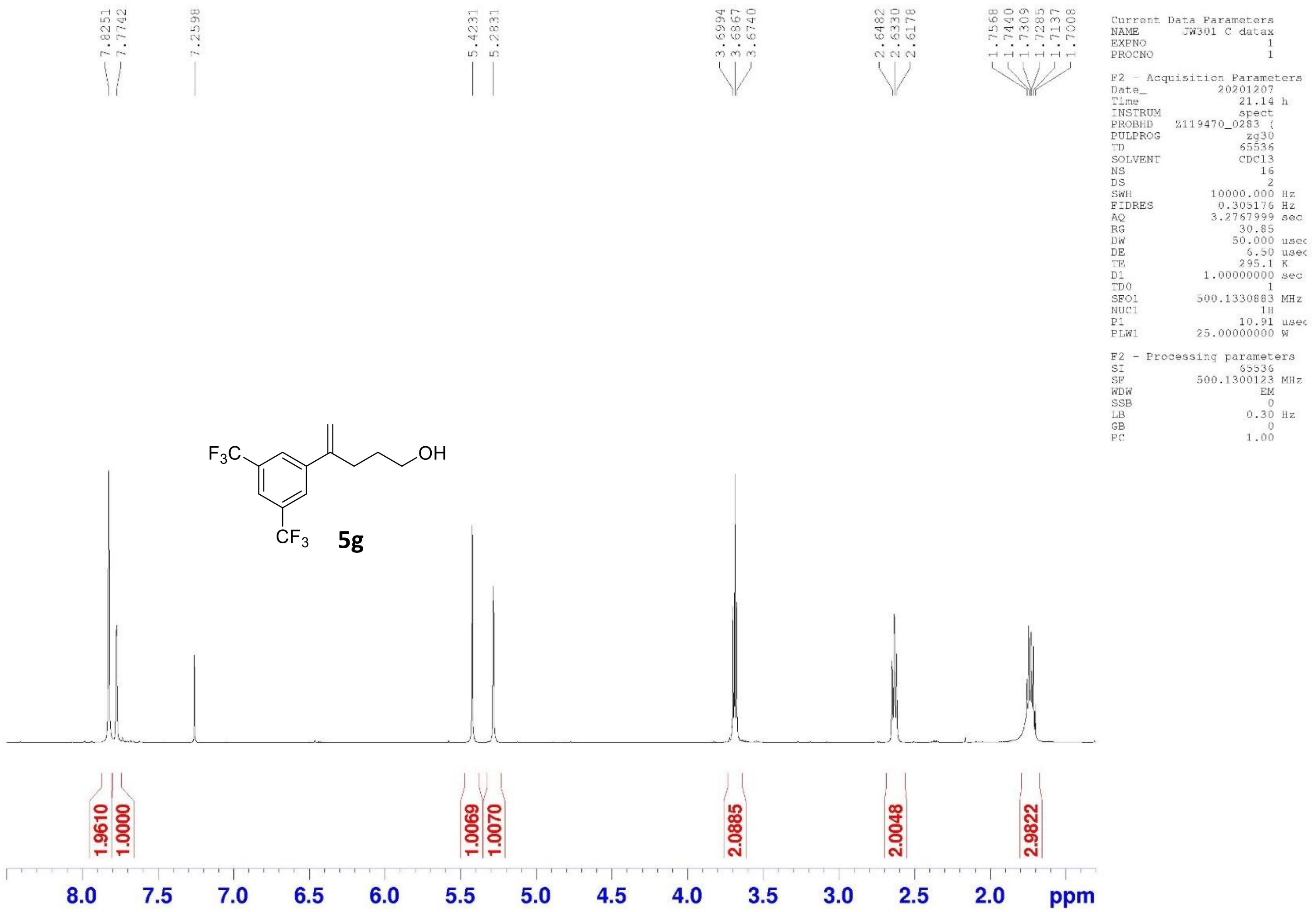

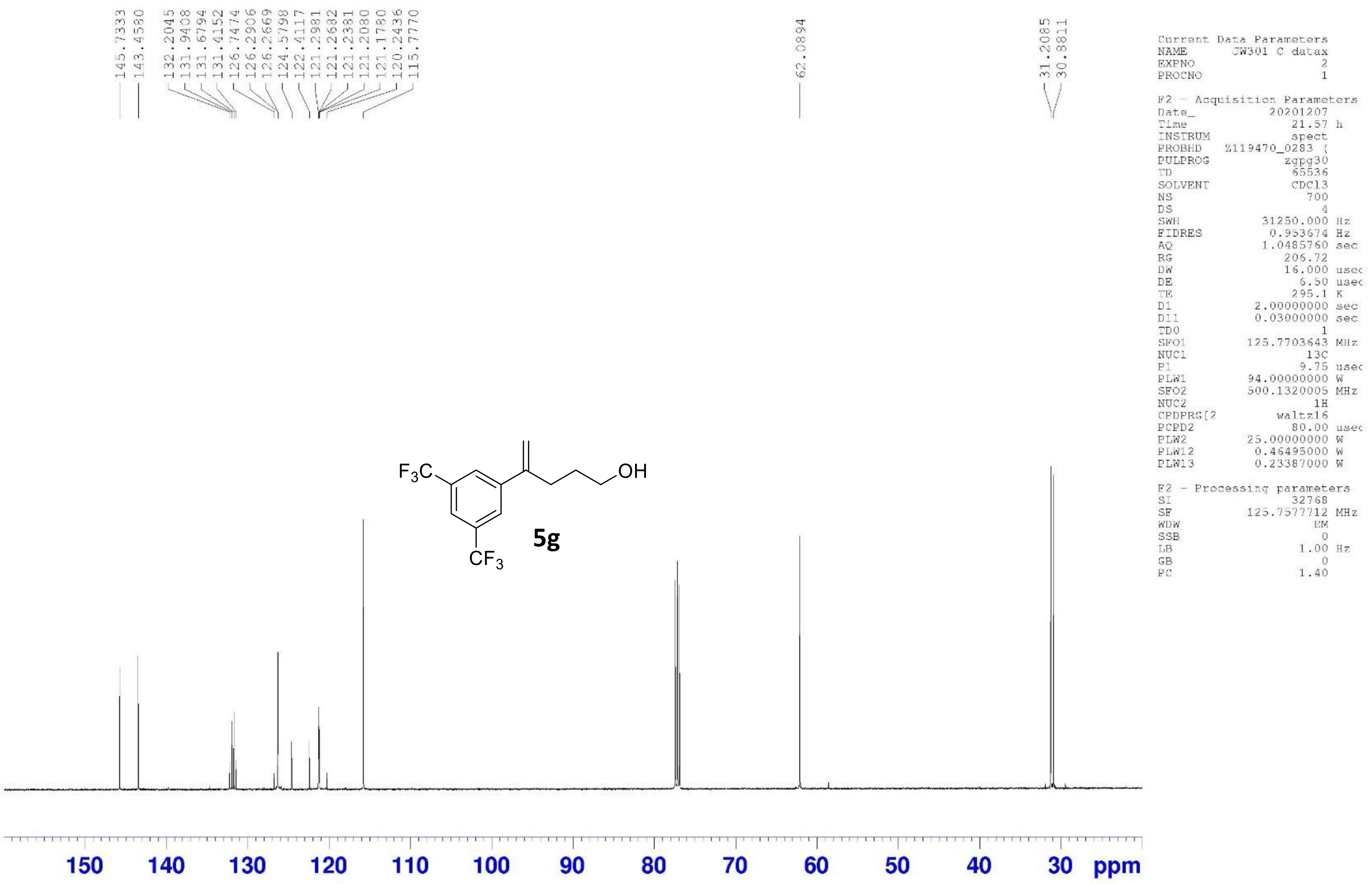
145.7333
143.4580
132.2045
131.9408
131.6794
131.4152
126.7474
126.2906
126.2669
124.5798
122.4117
121.2981
121.2682
121.2381
121.2080
121.1780
120.2436
115.7770
62.0894
31.2085
30.8811
F_3C
OH
CF_3
5g
150
140
130
120
110
100
90
80
70
60
50
40
30
ppm
Current Data Parameters
NAME JW301 C datax
EXPNO 2
PROCNO 1
F2 - Acquisition Parameters
Date_ 20201207
Time 21.57 h
INSTRUM spect
PROBHD Z119470_0283 (
PULPROG zgpg30
TD 65536
SOLVENT CDCl3
NS 700
DS 4
SWH 31250.000 Hz
FIDRES 0.953674 Hz
AQ 1.0485760 sec
RG 206.72
DW 16.000 usec
DE 6.50 usec
TE 295.1 K
D1 2.00000000 sec
D11 0.03000000 sec
TD0 1
SFO1 125.7703643 MHz
NUC1 13C
P1 9.75 usec
PLW1 94.00000000 W
SFO2 500.1320005 MHz
NUC2 1H
CPDPRG[2 waltz16
PCPD2 80.00 usec
PLW2 25.00000000 W
PLW12 0.46495000 W
PLW13 0.23387000 W
F2 - Processing parameters
SI 32768
SF 125.7577712 MHz
WDW EM
SSB 0
LB 1.00 Hz
GB 0
PC 1.40
